# Evaluating a Guideline-Integrated Clinical Interaction Framework Vs a Standard Large Language Model Interaction for Dietary Recommendations in Recurrent Urolithiasis: In Silico Study

**DOI:** 10.2196/95162

**Published:** 2026-09-04

**Authors:** Xiaofeng Wang, Jun Li, Yudong Hu, Yujie Chen, Yong Zhong, Faming Zhu, Ye Yuan, Fan Yang, Jin Ye

**Affiliations:** 1 Department of Urology, The Thirteenth People’s Hospital, No. 16, Railway New Village, Huangjueping Subdistrict, Jiulongpo District, Chongqing, China, 86 15808005558

**Keywords:** urolithiasis, kidney stones, large language models, AI, dietary counseling, metabolic evaluation, 24-hour urine analysis, synthetic clinical vignettes, in silico evaluation

## Abstract

**Background:**

Personalized dietary counseling is central to recurrence prevention in patients with urolithiasis, particularly after a 24-hour urine metabolic evaluation. However, translating quantitative metabolic abnormalities into patient-facing, guideline-concordant, and safe dietary recommendations can be challenging in routine clinical practice. Large language models (LLMs) may assist with this task, but unguided responses may overlook key metabolic priorities or case-specific safety constraints.

**Objective:**

This study evaluated whether a guideline-integrated, safety-aware, LLM-based clinical interaction framework (StoneAgent) could generate higher-quality, personalized dietary recommendations than a standard LLM configuration for recurrent urolithiasis. We also assessed whether any performance advantage persisted when the same clinical scenarios were presented as patient query–style inputs.

**Methods:**

We conducted an in silico comparative study using 30 synthetic clinical vignettes representing common, mixed, and safety-relevant metabolic stone scenarios. For the primary experiment, StoneAgent and a standard LLM configuration were compared using structured vignette inputs. For the robustness experiment, each vignette was reformulated into 2 patient query–style variants (query A and query B), preserving the same clinical content in more natural conversational language. A vignette-specific expert reference standard was developed from guideline-informed specialist consensus. Three independent reviewers blindly rated outputs on a 5-point Likert scale for metabolic specificity, guideline adherence, and actionability; safety was assessed as a binary outcome. For the patient query–style experiment, query A and query B were aggregated at the vignette level for paired comparison.

**Results:**

In the structured-input experiment, StoneAgent achieved higher performance than the standard LLM across metabolic specificity, guideline adherence, and actionability, with median case-level scores of 5.00 (IQR 5.00‐5.00) vs 3.00 (IQR 2.75‐3.92) for metabolic specificity, 5.00 (IQR 5.00‐5.00) vs 3.67 (IQR 3.08‐4.00) for guideline adherence, and 5.00 (IQR 5.00‐5.00) vs 3.00 (IQR 2.67‐3.33) for actionability (all *P*<.001). Safety pass rates were 100% (30/30) for StoneAgent and 83.3% (25/30) for the standard LLM (exact McNemar *P*=.06). In the patient query–style robustness experiment, StoneAgent retained a directional advantage after case-level aggregation, with mean scores of 4.44 vs 3.62 for metabolic specificity, 4.51 vs 3.63 for guideline adherence, and 4.11 vs 3.40 for actionability. Safety pass rates were 100% (30/30) for StoneAgent and 90% (27/30) for the standard LLM (exact McNemar *P*=.25). The performance gap was more conservative under patient query–style inputs compared with structured inputs, but the overall pattern remained consistent across domains.

**Conclusions:**

In this in silico study, a guideline-integrated, safety-aware clinical interaction framework generated higher-quality dietary recommendations for recurrent urolithiasis than a standard LLM condition with structured vignette inputs. This advantage was retained with patient query–style inputs. These findings suggest that explicit clinical framing, guideline grounding, and safety-oriented response scaffolding may improve the reliability of specialty counseling tasks involving metabolic stone prevention. Further validation is needed using real patient-authored queries and prospective clinical workflows.

## Introduction

Urolithiasis is a common condition worldwide, affecting roughly 7% to 13% of adults in North America and Europe [1]. Nearly half of patients will develop another stone within 10 years of the initial diagnosis [2]. Modern surgical techniques, including flexible ureteroscopy, have made the treatment of acute stone episodes far more effective. However, surgery primarily removes existing stones and does not correct the metabolic factors responsible for stone formation [3]. For many patients, this means that the cycle of stone formation and intervention continues over time. Recurrent procedures are therefore common and are associated with both reduced quality of life and increased health care costs [4]. Because of this, long-term management now places growing emphasis on prevention rather than repeated surgical treatment alone [5].

Clinical practice guidelines from the European Association of Urology (EAU) [6] and the Canadian Urological Association (CUA) [7] recommend metabolic evaluation for patients with recurrent or high-risk stones. In most cases, this involves 24-hour urine testing to detect metabolic abnormalities linked to stone formation, such as hypercalciuria, hypocitraturia, hyperoxaluria, and abnormal urinary pH [8,9]. These metabolic findings can guide individualized dietary and lifestyle recommendations aimed at reducing recurrence [10]. In reality, however, applying these recommendations consistently in routine practice is not straightforward. Interpreting metabolic profiles requires expertise, and detailed dietary counseling can be difficult to provide in busy clinical settings [7]. As a result, many patients receive only general advice, for example, increasing fluid intake without recommendations that specifically target their metabolic risk factors [11].

Large language models (LLMs), including systems such as OpenAI’s ChatGPT, have shown promise in medical communication and decision support, including patient education and the summarization of complex clinical information [12,13]. However, the task addressed in recurrent urolithiasis is not simply to generate general dietary advice; it requires the model to interpret quantitative metabolic findings, prioritize the dominant stone-risk pattern, and translate those findings into recommendations that remain consistent with guideline principles and clinically safe in the presence of comorbid conditions. In this setting, unconstrained LLM responses may appear plausible while omitting key metabolic targets, misprioritizing the main preventive strategy, or overlooking case-specific safety constraints [14-16].

An additional challenge is that real-world counseling requests are rarely presented as neatly structured case summaries. Patients typically ask questions in conversational language, with variable ordering of information and uneven emphasis on laboratory findings, symptoms, and concerns. As a result, performance observed under standardized vignette inputs may overestimate how reliably an LLM-based counseling framework can generalize to more naturalistic consultation scenarios. Evaluating robustness to patient query–style inputs is therefore important when assessing the practical usefulness of guideline-integrated LLM-based counseling approaches.

In this study, we evaluated a guideline-integrated, safety-aware, LLM-based clinical interaction framework (StoneAgent) for generating personalized dietary recommendations in recurrent urolithiasis. We first compared StoneAgent with a standard LLM configuration using structured synthetic clinical vignettes designed to reflect common and safety-relevant metabolic stone scenarios. We then performed a robustness experiment using patient query–style variants derived from the same vignettes to assess whether any performance advantage was retained under more naturalistic conversational inputs. We hypothesized that, compared with a standard unguided LLM interaction, StoneAgent would produce recommendations that were more metabolically specific, more concordant with guideline-based care, more actionable for patients, and safer in clinically constrained scenarios.

## Methods

### Study Design and Dataset

#### Study Design

This in silico comparative study consisted of 2 linked experimental phases. In the primary phase, StoneAgent and a standard LLM configuration (GPT-5.1) were compared using structured synthetic clinical vignettes containing standardized clinical context and 24-hour urine metabolic data. In the second phase, each vignette was reformulated into 2 patient query–style variants to evaluate whether the relative performance of the 2 frameworks was preserved under more natural conversational inputs. The same vignette-specific expert reference standard was used as the scoring anchor for both phases.

**Figure 1.**
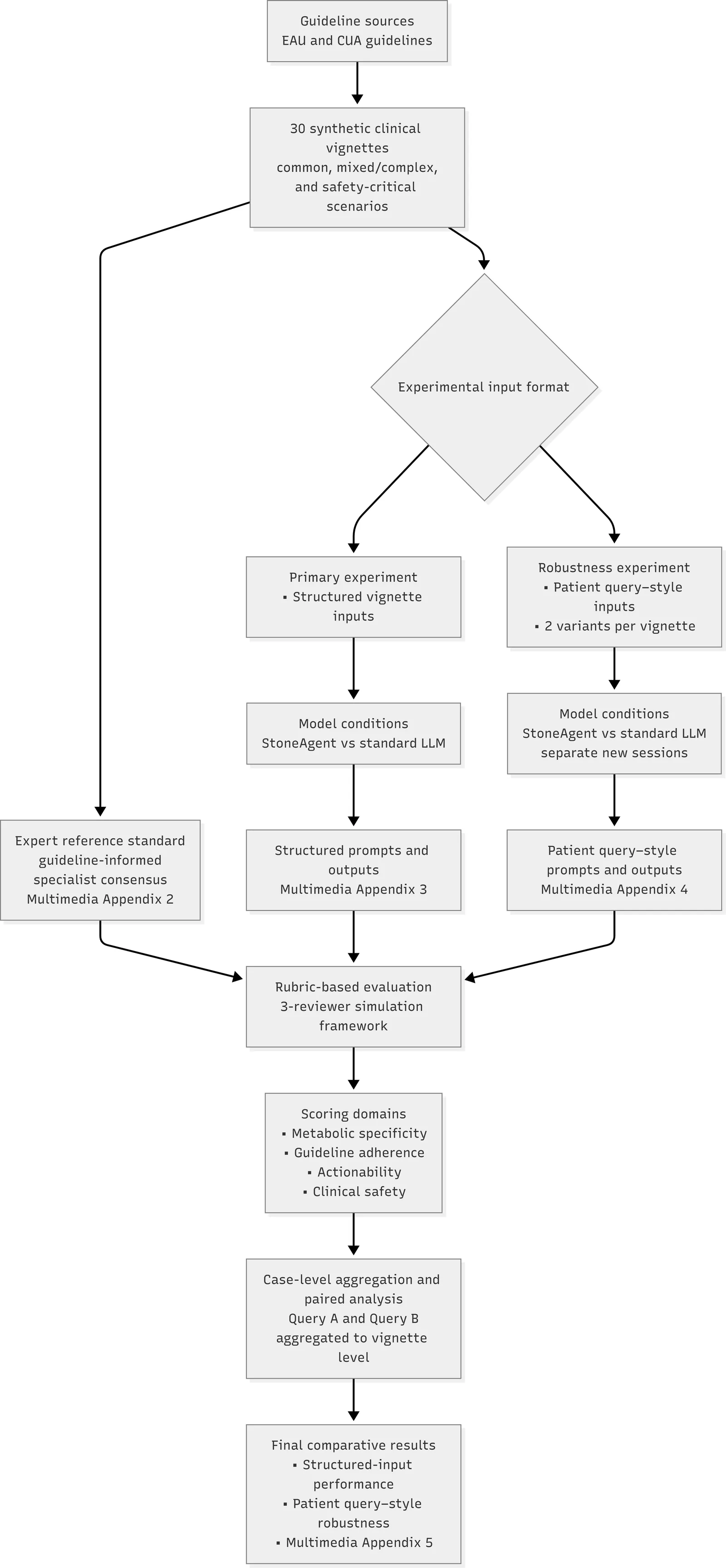
Study workflow diagram. CUA: Canadian Urological Association; EAU: European Association of Urology; LLM: large language model.

Thirty synthetic clinical vignettes were purposively constructed based on EAU and CUA guideline-relevant metabolic stone scenarios [6,7] and stratified into categories A to C. In both phases, outputs from StoneAgent and the standard LLM were evaluated by 3 blinded reviewers for metabolic specificity, guideline adherence, and actionability using a 5-point Likert scale, while safety was evaluated separately as a binary outcome. In the query-style experiment, the 2 patient-style variants derived from the same vignette were aggregated at the vignette level for the primary paired comparison. The overall workflow is summarized in Figure 1.

#### Ethical Considerations

This study used synthetic clinical vignettes generated specifically for research purposes and did not involve real patients, identifiable health information, clinical records, or interactions with human participants. Institutional review board approval was not required because the study did not involve human participants or access to clinical data. Informed consent was not applicable because no human participant data were collected or analyzed.

#### Dataset

We constructed a dataset of 30 synthetic clinical vignettes to cover a broad range of metabolic patterns described in the EAU and CUA guidelines for urolithiasis [6,7]. Before analysis, the vignettes were reviewed for content validity by a senior urologist (>15 y of experience) who did not participate in scoring. The reviewer checked that (1) each vignette reflected plausible clinical presentations and (2) the metabolic profiles were physiologically consistent. Ambiguous or unrealistic elements were revised accordingly. The full vignette dataset is provided in Multimedia Appendix 1.

Each vignette included 3 layers of information:

1. Patient characteristics: age, sex, BMI, and comorbidities (eg, hypertension, type 2 diabetes, and chronic kidney disease)
2. Stone history: recurrence pattern, reported stone composition (eg, calcium oxalate, uric acid, and struvite), and prior procedures
3. A 24-hour urine profile includes urine volume and quantitative parameters, including calcium, sodium, oxalate, citrate, uric acid, magnesium, and urine pH

#### Cohort Stratification

To reflect differences in clinical complexity, the 30 vignettes were stratified into 3 prespecified categories: (1) single metabolic abnormality, (2) mixed or complex metabolic abnormalities, and (3) safety-critical scenarios in which otherwise standard dietary recommendations could become inappropriate because of patient-specific clinical factors. The comorbid conditions included in the safety-critical category were selected based on their potential to alter routine dietary counseling in recurrent stone prevention, including chronic kidney disease, heart failure, pregnancy, infection-related stones, primary hyperparathyroidism, and age-related functional limitations affecting hydration advice. These conditions were chosen because they represent clinically relevant situations in which generic dietary recommendations may require modification, additional caution, or prioritization of alternative management strategies. We focused on representative high-impact scenarios rather than attempting to reproduce the full spectrum of multimorbidity encountered in clinical practice. The distribution and key features of these vignettes are summarized in Table 1.

**Table 1.** Characteristics and stratification of the 30 synthetic clinical vignettes used in the study (N=30)^a^.

Category and clinical phenotype	Defining features	Number of cases (n)	Example cases
Single metabolic abnormality (n=12)			
Sodium-dependent hypercalciuria	Urine Ca (>8.0 mmol/d) driven by high Na (>200 mmol/d); normal oxalate	4	Case ID: C01, C12, C26
Dietary hyperoxaluria	Urine oxalate >0.5 mmol/d; high intake of oxalate-rich foods (eg, spinach, nuts)	3	Case ID: C04, C29
Hypocitraturia	Urine citrate <1.5 mmol/d; often associated with low fruit/veg intake or high acid load	3	Case ID: C03, C13, C17
Uric acid stones	Persistently low urine pH (<5.5); hyperuricosuria; gout history	2	Case ID: C05, C28
Mixed and complex abnormalities (n=10)			
Mixed CaOx + uric acid	Hypercalciuria combined with hyperuricosuria and/or low pH	3	Case ID: C07, C20
Infection stones (struvite)	High urine pH (>7.5); high ammonium; recurrent UTIs^b^	2	Case ID: C09
Enteric hyperoxaluria	Severe hyperoxaluria (>1.0 mmol/d) due to malabsorption (eg, Crohn, IBD^c^)	2	Case ID: C08
Renal tubular acidosis	High urine pH (>6.8); severe hypocitraturia; calcium phosphate stones	2	Case ID: C10, C18
Cystinuria	Genetic defect; positive urinary cystine	1	Case ID: C11
Safety-critical scenarios (n=8)			
Congestive heart failure	Fluid volume sensitivity; potential harm from sodium bicarbonate load	2	Case ID: C21
Chronic kidney disease	Stage 3b-4 (GFR^d^<45); risk of hyperkalemia and fluid overload	2	Case ID: C22
Pregnancy	Physiological hypercalciuria; restrictions on pharmacotherapy	2	Case ID: C23
Primary hyperparathyroidism	Hypercalcemia; surgical indication rather than dietary alone	1	Case ID: C16
Urinary incontinence	Older adults; risk of worsening symptoms with aggressive hydration	1	Case ID: C24

a The number of representative cases listed for each phenotype does not always equal the total case count because only selected examples are provided to illustrate the clinical category. All 30 synthetic clinical vignettes were included in the evaluation dataset.

b UTI: urinary tract infection.

c IBD: inflammatory bowel disease.

d GFR: glomerular filtration rate.

#### Reference Standard

For each vignette, a guideline-concordant dietary plan was established as the reference standard by 2 senior endourologists (>15 y of experience in stone management) using EAU and CUA recommendations [6,7]. Discrepancies were resolved by consensus. The complete set of reference standard recommendations is provided in Multimedia Appendix 2.

### Development of the StoneAgent Framework

#### Underlying Model and Configuration

The recommendations evaluated in this study were generated using an LLM accessed through the GPT-5.1 web interface. The experiments were conducted between December 2025 and January 2026 using the model version available on the platform at that time. Both the StoneAgent framework and the standard LLM condition used the same underlying model; the only difference between conditions was the interaction framework and the prompt structure applied to the model.

Because the web interface does not provide direct control over generation parameters such as temperature, top-p sampling, or fixed model snapshot identifiers, all outputs were produced using the default platform configuration. Accordingly, this study should be interpreted as an operational comparative evaluation under real-world usage conditions rather than a fully parameter-controlled API benchmark. To minimize context carryover, each clinical vignette or query variant was evaluated in a separate, newly initiated conversation session. No custom instructions, memory functions, or external tools were used during generation.

Two framework conditions were evaluated. The first, StoneAgent, represented a guideline-integrated prompting framework designed to support the interpretation of metabolic evaluation results and the generation of personalized dietary recommendations. The second represented a standard, general-purpose LLM condition, in which the same clinical content was presented without the structured clinical reasoning scaffold. To enhance transparency and reproducibility, the full prompt templates were prespecified and are reported in Multimedia Appendices 3 and 4, and the complete model outputs were retained and shared in the supplementary materials.

#### Prompt Engineering Strategy

The StoneAgent framework was developed to address the specific requirements of metabolic stone prevention counseling rather than to improve general conversational performance. The framework design was informed by the clinical workflow used by specialists when interpreting 24-hour urine metabolic evaluations and translating findings into dietary recommendations. Specifically, the prompt structure was organized into 5 sequential components: (1) clinical role orientation, which established a specialist perspective for metabolic stone management; (2) task definition, which instructed the model to interpret the clinical scenario and generate personalized dietary guidance; (3) metabolic reasoning structure, which required identification of dominant urinary abnormalities and their potential dietary drivers; (4) safety constraints, which prompted consideration of comorbid conditions and situations requiring modification of standard advice; and (5) output organization, which emphasized prioritized, patient-usable recommendations. This structure was designed to mirror key steps in clinical dietary counseling while remaining compatible with a general-purpose LLM.

StoneAgent was designed as a guideline-integrated response framework for the interpretation of metabolic stone evaluations. Rather than relying on the model’s default conversational behavior, the framework imposed a structured clinical task orientation: identifying the dominant metabolic abnormalities, linking those abnormalities to likely dietary drivers, prioritizing preventive recommendations, and checking whether standard advice required modification because of comorbid conditions or other safety constraints. The output was structured to emphasize patient-usable dietary counseling while preserving fidelity to established clinical guidance.

Within this framework, the model was instructed to interpret the clinical vignette from the perspective of a clinician familiar with metabolic stone disease and current guideline recommendations. The instructions emphasized alignment with the principles outlined in the EAU and CUA guidelines [6,7] and highlighted the importance of considering patient safety when comorbid conditions were present.

The prompt structure also encouraged the model to review the metabolic profile before generating recommendations. Urinary abnormalities, such as hypercalciuria, hypocitraturia, hyperoxaluria, and low urine volume, were expected to be interpreted in relation to established metabolic risk patterns.

The generated output focused on practical dietary guidance that could reasonably be communicated to patients during clinical counseling. Recommendations were, therefore, constrained to clear and actionable lifestyle measures rather than broad or nonspecific advice.

The complete prompt templates used in the StoneAgent configuration, together with the full set of model-generated responses for all clinical vignettes, are provided in Multimedia Appendix 3.

#### Agent Reasoning Framework

The StoneAgent framework was designed to approximate the reasoning process commonly used by clinicians when interpreting metabolic evaluations for kidney stone prevention. When a clinical vignette was provided as input, the model first examined the clinical context, including patient demographics, comorbidities, and prior stone history. These elements were considered to ensure that any dietary recommendations would remain appropriate for the patient’s overall clinical condition.

The model then analyzed the 24-hour urine metabolic profile to identify abnormalities associated with stone formation. Identified abnormalities were subsequently interpreted in relation to potential dietary or metabolic etiologies, allowing the system to link laboratory findings with modifiable lifestyle factors relevant to stone prevention. Parameters such as urinary calcium, citrate, oxalate, sodium, and urinary pH were interpreted in relation to established metabolic risk patterns described in clinical guidelines [6,7,17].

Based on this interpretation, the model generated dietary recommendations tailored to the metabolic findings and clinical context. The output focused on modifiable dietary and lifestyle factors commonly addressed in stone prevention, including fluid intake, sodium restriction, calcium consumption, oxalate exposure, and citrate supplementation, where appropriate.

This reasoning structure was intended to reflect guideline-based clinical decision-making and to reduce the likelihood that the model would generate generic or inconsistent recommendations.

### Experimental Setup and Comparison Groups

#### Study Conditions and Reference Standard

To evaluate the contribution of structured clinical framing rather than differences in underlying model capability, we compared 2 interaction frameworks implemented on the same base LLM against a vignette-specific expert reference standard. The 2 conditions intentionally used different prompt structures because the objective was to evaluate whether a guideline-integrated clinical interaction framework could improve the reliability and usefulness of a general-purpose LLM for a specialty counseling task. In the StoneAgent condition, the model received the vignette within the guideline-integrated framework described above, which incorporated structured metabolic interpretation, safety considerations, and patient-oriented output organization. In the standard LLM condition, the same clinical content was presented without the additional reasoning scaffold, allowing the model to respond using its default general-purpose conversational behavior. Therefore, the observed differences should be interpreted as the contribution of framework-level design elements, including clinical framing, guideline grounding, and safety-oriented scaffolding, rather than as differences in the intrinsic capability of the underlying language model.

The expert reference standard was developed for each vignette by 2 senior endourologists with experience in metabolic stone prevention. Each expert independently reviewed the vignette and drafted guideline-concordant dietary recommendations based on current EAU and CUA guidance [6,7]. Differences were resolved through discussion to create a single consensus reference response for scoring. Full prompt templates and complete model outputs for the structured-input and patient query–style robustness experiments are provided in Multimedia Appendices 3 and 4.

#### Query-Style Input Robustness Experiment

To assess robustness under more naturalistic input conditions, each structured vignette was reformulated into 2 patient query–style variants (query A and query B). These variants preserved the same underlying clinical scenario and 24-hour urine findings, but presented the information in a conversational format with realistic differences in wording, emphasis, and information order. No new clinical facts were introduced beyond those contained in the original vignette.

For each query variant, StoneAgent and the standard LLM configuration generated responses in separate new conversation sessions to minimize carryover effects from prior prompts or outputs. This design allowed us to examine whether the relative performance of the 2 systems was preserved when the same clinical content was expressed in a more patient-like manner. Because query A and query B represented 2 phrasings of the same clinical case, rather than independent cases, the primary robustness analysis was performed at the vignette level after aggregating the 2 query variants for each case.

### Outcome Measures and Evaluation Procedure

Outputs from the 2 model conditions were evaluated using a structured scoring framework anchored to the vignette-specific expert reference standard. For each case, scoring focused on whether the response correctly identified the dominant metabolic pattern, prioritized recommendations consistent with guideline-based care, translated the findings into practical patient-facing advice, and avoided clinically inappropriate suggestions.

Three independent reviewers, blinded to study arm allocation, evaluated all outputs using the same predefined rubric. To reduce the likelihood of recognition bias, outputs were deidentified and scored according to content rather than response source. The evaluation framework was applied to both the structured-input experiment and the patient query–style robustness experiment. The detailed scoring rubric is summarized in Table 2, and the final reviewer-scoring dataset is provided in Multimedia Appendix 5.

**Table 2.** Standardized scoring rubric used by expert reviewers to evaluate AI responses.

Score	Metabolic specificity	Guideline adherence	Actionability
1 (Very poor)	Fails to identify relevant metabolic abnormalities; provides inaccurate or irrelevant recommendations	Direct contradiction of established guideline recommendations	Recommendations are unclear, impractical, or potentially misleading
2 (Poor)	Identifies abnormalities but does not link them to specific etiologies or provides largely generic advice	Partially inconsistent with guidelines or omits key management targets	Advice lacks clarity or clinical applicability
3 (Fair)	Correctly identifies major abnormalities but provides limited explanation of their clinical implications	Generally consistent with guidelines but lacks specificity or nuance	Recommendations are understandable but insufficiently prioritized
4 (Good)	Identifies specific metabolic drivers and links them to appropriate dietary modifications	Consistent with guideline-based targets and appropriate for the clinical context	Recommendations are clear, logically organized, and clinically useful
5 (Excellent)	Demonstrates comprehensive interpretation of metabolic findings, including interaction among abnormalities when present	Fully concordant with guideline recommendations, including context-specific considerations	Recommendations are clear, prioritized according to clinical importance, and readily applicable in practice

Three ordinal domains were assessed: metabolic specificity, guideline adherence, and actionability. Each domain was scored on a 5-point Likert scale, with higher scores indicating better performance. For each output, scores from the 3 reviewers were averaged to generate a composite score for each ordinal domain.

Metabolic specificity reflected the extent to which the response was tailored to the metabolic abnormalities and the clinical scenario presented in the case. Higher scores were assigned when the output correctly prioritized the dominant stone-risk pattern and linked specific abnormalities, such as hypercalciuria, hypocitraturia, hyperoxaluria, low urine volume, or urine pH abnormalities, to appropriate dietary recommendations.

Guideline adherence was evaluated by the degree to which the recommendations were consistent with the expert reference standard and current guideline principles. Higher scores were awarded when the response captured the key recommended measures without introducing advice that conflicted with established dietary management strategies for recurrent stone prevention.

Actionability assessed whether responses translated the metabolic interpretation into clear, feasible, and patient-usable dietary guidance. Responses received higher scores when they provided specific instructions, practical priorities, and language that could reasonably support clinical counseling or patient follow-up.

In addition to these ordinal domains, safety was assessed as a binary outcome. A response was classified as unsafe if it omitted or contradicted an important case-specific safety constraint, particularly in scenarios in which otherwise standard dietary advice required modification because of comorbid disease, pregnancy, advanced age, infection-related stones, or other clinically relevant conditions. For the primary safety analysis, consensus failure was defined a priori as a fail judgment assigned by at least 2 of the 3 reviewers.

### Statistical Analysis

The primary unit of analysis was the clinical vignette. For the structured-input experiment, paired comparisons were performed between StoneAgent and the standard LLM for each vignette. For the patient query–style robustness experiment, query A and query B represented 2 phrasings of the same underlying case rather than independent observations; therefore, reviewer-averaged scores for the 2 query variants were first aggregated at the vignette level before paired comparison.

For the ordinal domains of metabolic specificity, guideline adherence, and actionability, scores from the 3 independent reviewers were averaged to obtain a composite score for each output. These ordinal outcomes are summarized as medians with IQRs, and means are also reported for descriptive completeness where appropriate. Paired comparisons between StoneAgent and the standard LLM were performed using the Wilcoxon signed-rank test.

Safety was analyzed as a paired binary outcome. For each output, consensus failure was defined a priori as a fail judgment assigned by at least 2 of the 3 reviewers. Paired comparisons of safety pass rates between study arms were evaluated using the exact McNemar test.

Query-level results are presented descriptively to illustrate within-case consistency across the 2 patient-style phrasings, whereas vignette-level aggregation was used for the primary robustness comparison. Interrater reliability for the ordinal rubric domains was assessed using the intraclass correlation coefficient (ICC), based on a 2-way mixed-effects model for absolute agreement [18]. Because reviewer scores were averaged to generate composite vignette-level scores, average-measures ICCs were used for the primary interpretation, with single-measure ICCs calculated for reference. Agreement for the binary safety assessment was summarized, where reviewer-level data were available, using percent agreement and Fleiss κ. All tests were 2-sided, and *P*<.05 was considered statistically significant. Given the exploratory nature of this in silico comparative study, secondary domain-level comparisons were interpreted as supportive rather than strictly confirmatory. All analyses were performed using SPSS, version 29.0 (IBM Corp).

### Reporting Guideline

This study was reported with consideration of the relevant recommendations for evaluating AI-based clinical interaction and decision support approaches. The completed checklist is provided as a supplementary file.

## Results

### Overall Performance

A total of 30 synthetic clinical vignettes were evaluated in the structured-input experiment, and each vignette was also reformulated into 2 patient query–style variants for the robustness experiment. Across both phases, StoneAgent generally outperformed the standard LLM in metabolic specificity, guideline adherence, and actionability, while also demonstrating a more favorable safety profile. The full prompt and response sets are provided in Multimedia Appendices 3 and 4, and the reviewer scoring dataset is provided in Multimedia Appendix 5.

In the structured-input experiment, StoneAgent showed consistently higher paired ratings than the standard LLM across all 3 ordinal domains. Median scores reached 5.00 across metabolic specificity, guideline adherence, and actionability, whereas the standard LLM showed lower and more variable performance. Safety pass rates were numerically higher for StoneAgent (30/30 vs 25/30), although this difference did not reach statistical significance in exact paired testing.

In the patient query–style experiment, the absolute differences between frameworks were more conservative after vignette-level aggregation of query A and query B, but the directional advantage of StoneAgent was retained across the 3 ordinal domains, with numerically higher safety pass rates as well. Taken together, these findings indicate that the observed advantage of StoneAgent was not limited to idealized structured inputs and remained detectable when the same cases were presented in more natural conversational language.

### Interrater Reliability

Interrater reliability across the 3 ordinal rubric domains was excellent in both experimental phases. In the structured-input experiment, the average-measures ICC(A,3) was 0.991 for metabolic specificity, 0.961 for guideline adherence, and 0.967 for actionability. In the patient query–style robustness experiment, after vignette-level aggregation of query A and query B, ICC(A,3) values were 0.978 for metabolic specificity, 0.988 for guideline adherence, and 0.974 for actionability. These findings support the use of reviewer-averaged composite scores for vignette-level comparison.

For the binary safety assessment, reviewer-level ratings were available in the query-level dataset, where agreement was also high (Fleiss κ=0.944; overall agreement 99.4%). For the structured-input experiment, only the final consensus safety classification was retained in the exported appendix workbook.

A summary of the paired case-level results for the structured-input experiment is presented in Table 3.

**Table 3.** Case-level performance of StoneAgent vs a standard large language model (LLM) in the structured-input experiment^a^.

Domain	StoneAgent	Standard LLM	*P* value
Metabolic specificity, median (IQR)	5.00 (5.00‐5.00)	3.00 (2.75‐3.92)	<.001
Guideline adherence, median (IQR)	5.00 (5.00‐5.00)	3.67 (3.08‐4.00)	<.001
Actionability, median (IQR)	5.00 (5.00‐5.00)	3.00 (2.67‐3.33)	<.001
Clinical safety, n/N (%)	30/30 (100)	25/30 (83.3)	.06 (exact McNemar)

a Values are presented as median (IQR). Paired comparisons for ordinal domains were performed using the Wilcoxon signed-rank test. Safety outcomes were compared using the exact McNemar test.

### Metabolic Specificity

#### Quantitative and Qualitative Findings

In the structured-input experiment, StoneAgent received higher metabolic specificity scores than the standard LLM, indicating more consistent recognition and prioritization of the dominant metabolic abnormalities within each vignette (median 5.00, IQR 5.00‐5.00 vs median 3.00, IQR 2.75‐3.92; Wilcoxon signed-rank *P*<.001). In the patient query–style robustness experiment, this advantage was retained after aggregation of query A and query B at the vignette level (median 4.67, IQR 4.00‐5.00 vs median 4.00, IQR 3.00‐4.00; *P*<.001), suggesting that StoneAgent remained better able to map patient-style input back to the underlying metabolic-risk pattern.

Qualitatively, StoneAgent’s responses more often linked specific abnormalities, such as hypercalciuria, hyperoxaluria, hypocitraturia, urine pH abnormalities, or low urine volume, to corresponding dietary targets. By contrast, the standard LLM more often defaulted to broadly appropriate but less case-prioritized advice, particularly in scenarios requiring identification of a dominant metabolic driver rather than generic stone-prevention counseling.

**Figure 2.**
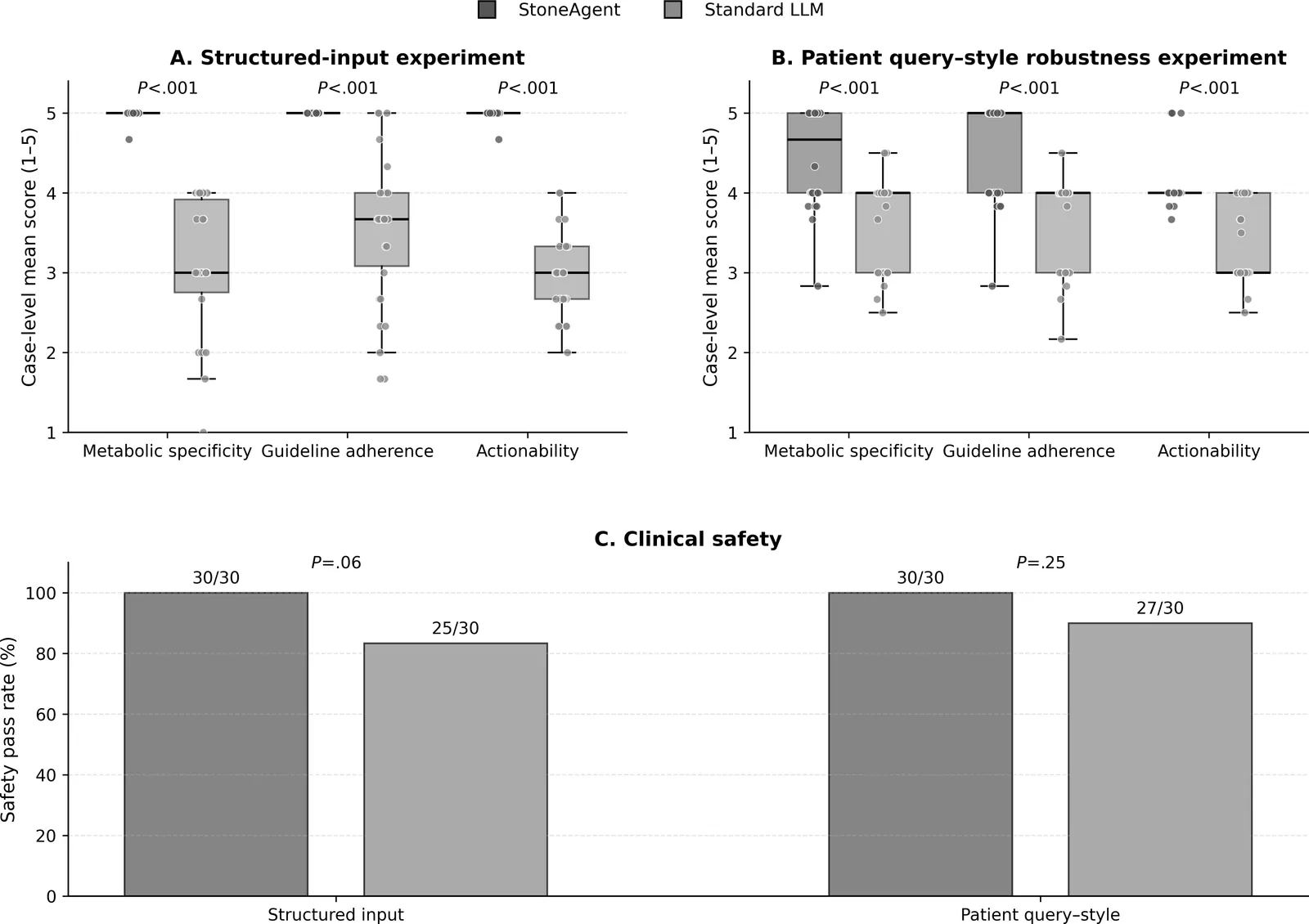
Distribution of case-level evaluation scores and safety outcomes for StoneAgent vs a standard large language model (LLM) across the structured-input and patient query–style experiments. Panels A and B show the distributions of case-level mean scores for metabolic specificity, guideline adherence, and actionability. Each point represents 1 vignette; boxplots indicate the median and IQR. For the patient query–style robustness experiment, query A and query B were aggregated at the vignette level before analysis. Panel C shows safety pass rates for each model condition in both experimental phases. *P* values for ordinal domains were calculated using the Wilcoxon signed-rank test, and safety was compared using the exact McNemar test.

The tabulated results in Tables 3 and 4 are complemented by the score distributions shown in Figure 2.

**Table 4.** Case-level performance of StoneAgent vs a standard large language model (LLM) in the patient query–style robustness experiment^a^.

Domain	StoneAgent	Standard LLM	*P* value	Mean difference
Metabolic specificity, median (IQR)	4.67 (4.00‐5.00)	4.00 (3.00‐4.00)	<.001	+0.82
Guideline adherence, median (IQR)	5.00 (4.00‐5.00)	4.00 (3.00‐4.00)	<.001	+0.88
Actionability, median (IQR)	4.00 (4.00‐4.00)	3.00 (3.00‐4.00)	<.001	+0.71
Clinical safety, n/N (%)	30/30 (100)	27/30 (90)	.25 (exact McNemar)	+10 percentage points

a Ordinal domain scores represent vignette-level composite ratings from 3 blinded reviewers after aggregation of query A and query B. Paired comparisons were performed using the Wilcoxon signed-rank test and are summarized as medians (IQRs); mean differences are shown for descriptive comparison. Clinical safety was analyzed separately as a binary outcome using the exact McNemar test.

#### Guideline Adherence

StoneAgent also showed higher guideline adherence than the standard LLM in the structured-input experiment (median 5.00, IQR 5.00‐5.00 vs median 3.67, IQR 3.08‐4.00; *P*<.001). In the patient query–style robustness experiment, the same directional advantage remained present after vignette-level aggregation (median 5.00, IQR 4.00‐5.00 vs median 4.00, IQR 3.00‐4.00; *P*<.001).

The difference was most apparent in cases in which guideline-concordant counseling required prioritization rather than simple completeness. These included scenarios in which standard stone-prevention advice needed to be modified because of infection-related stones, primary hyperparathyroidism, chronic kidney disease, heart failure, pregnancy, or other clinically relevant constraints. In such cases, StoneAgent more often preserved the central management priority and avoided advice that was superficially plausible but incompletely aligned with the reference standard.

#### Actionability

StoneAgent responses were also rated more actionable than those generated by the standard LLM. In the structured-input experiment, StoneAgent more often translated metabolic findings into clear, patient-usable priorities, such as sodium restriction targets, appropriate calcium intake strategies, fluid goals, oxalate-related counseling, or citrate-focused dietary measures (median 5.00, IQR 5.00‐5.00 vs median 3.00, IQR 2.67‐3.33; *P*<.001). In the patient query–style experiment, this advantage remained present, although the difference was more modest than that observed under structured inputs (median 4.00, IQR 4.00‐4.00 vs median 3.00, IQR 3.00‐4.00; *P*<.001).

The reported scores for metabolic specificity, guideline adherence, and actionability in the patient query–style robustness experiment represent vignette-level composite scores derived from ratings by 3 blinded reviewers. For this phase, each vignette was reformulated into 2 patient-style prompts (query A and query B), and reviewer-averaged scores were aggregated at the vignette level to avoid treating 2 phrasings of the same case as independent observations. Safety was analyzed separately as a binary outcome. A summary of the aggregated patient query–style comparison is presented in Table 4.

### Clinical Safety

StoneAgent showed a numerically more favorable safety profile than the standard LLM across both experimental phases (Tables 3 and 4). In the structured-input experiment, StoneAgent produced no consensus-fail responses, whereas 5 of 30 responses in the standard LLM group were classified as unsafe or insufficiently constrained (30/30 vs 25/30; exact McNemar *P*=.06). In the patient query–style robustness experiment, the same directional pattern persisted after vignette-level aggregation (30/30 vs 27/30; exact McNemar *P*=.25). These findings suggest a potential safety advantage associated with the StoneAgent framework, although the paired comparisons for safety did not reach conventional statistical significance in this sample.

The safety difference was most evident in vignettes in which standard stone-prevention recommendations required explicit modification because of comorbid conditions or competing clinical priorities. These included cases involving advanced chronic kidney disease, heart failure, pregnancy, primary hyperparathyroidism, infection-related stones, and older adults with functional constraints affecting hydration counseling.

### Illustrative Cases

Representative cases illustrated how framework-level differences translated into reviewer scoring. In case C09, an infection-related stone scenario, StoneAgent more consistently prioritized infection-focused management and stone clearance before routine dietary prevention advice, whereas the standard LLM was more likely to default to generic recurrence-prevention counseling [6,7,17]. In case C22, a chronic kidney disease scenario, StoneAgent more often modified otherwise standard recommendations related to fluid intake, alkalinization, or electrolyte-related advice in light of renal safety considerations, whereas the standard LLM was more prone to provide broadly reasonable but insufficiently constrained recommendations [6,7,19,20]. These examples highlight that the observed differences were not limited to response detail alone but also involved prioritization of the dominant clinical problem and preservation of case-specific safety logic.

## Discussion

### Principal Findings

This study evaluated whether a guideline-integrated, safety-aware clinical interaction framework could improve the quality of personalized dietary counseling outputs for recurrent urolithiasis when implemented on the same underlying LLM as the standard LLM interaction condition. In the structured-input experiment, StoneAgent generated recommendations that were more metabolically specific, more closely aligned with guideline-based management, and more actionable than those produced by the standard LLM configuration. Importantly, this performance advantage was not confined to standardized vignette inputs. When the same cases were reformulated as patient query–style prompts, StoneAgent retained a directional advantage across the main evaluation domains after vignette-level aggregation.

The observed difference was not simply a matter of producing longer or more detailed answers. Rather, StoneAgent more consistently identified the dominant metabolic problem, prioritized the most relevant preventive strategy, and modified standard dietary advice when case-specific safety constraints were present. The contrast with the standard LLM was most evident in vignettes involving comorbidity-sensitive counseling, mixed metabolic patterns, or scenarios in which broadly reasonable stone advice could still be clinically misprioritized.

Taken together, these findings support the view that specialty counseling tasks may benefit not only from general language competence but also from explicit clinical framing, guideline grounding, and safety-oriented response scaffolding.

It is important to interpret guideline adherence in the context of the framework design. Because explicit guideline integration was a predefined component of the StoneAgent framework, this outcome reflects the degree to which the framework successfully operationalized guideline-based counseling principles rather than representing an isolated measure of general reasoning ability. Therefore, the comparison evaluates the clinical use of a structured, guideline-oriented interaction approach relative to an unguided LLM interaction.

### Comparison With Previous Work

Prior studies on LLM use in urology have largely focused on general health information, patient education, or broad question-answering tasks [15,16,21-23]. By contrast, the present study addressed a narrower and more clinically constrained task: translating 24-hour urine metabolic findings into personalized dietary recommendations for recurrent stone prevention. This distinction is important because the quality of the response in this setting depends not only on fluency or factual recall but also on correctly prioritizing metabolic abnormalities, preserving guideline-consistent management logic, and respecting case-specific safety constraints.

Our findings also extend the literature by evaluating performance under both structured vignette inputs and patient query–style inputs. This dual design allowed us to assess not only whether a framework could perform well under standardized conditions but also whether its advantage was retained when the same cases were expressed in more natural conversational language. In that sense, the study evaluates whether a clinically structured, guideline-integrated interaction framework can improve task reliability across different input formats when compared with a standard LLM interaction.

More broadly, as general-purpose foundation models continue to improve, the key question may shift from whether they can respond to specialized counseling tasks at all to how they should be structured and governed for reliable use. Our findings suggest that explicit clinical framing and safety-aware scaffolding may still add value even when a general-purpose model is already capable of producing broadly plausible responses [12,14,15].

### Clinical Implications

These findings have potential implications for metabolic stone counseling, particularly in settings where clinicians must convert laboratory data into patient-facing advice under time constraints. A framework such as StoneAgent may support dietary counseling by organizing metabolic interpretation into more consistent and practical recommendations. Its potential value may be greatest not in replacing specialist judgment but in assisting with the translation of metabolic findings into structured counseling points that are easier to communicate during follow-up visits or preventive care discussions.

From an implementation perspective, the potential value of such a framework will depend not only on clinical performance but also on development, maintenance, and integration costs. This study did not perform a health economic evaluation and therefore cannot determine whether framework-assisted counseling would reduce health care expenditures. However, recurrent urolithiasis is associated with substantial downstream costs related to repeated procedures, emergency visits, and long-term management. Future studies should evaluate the cost-effectiveness of guideline-integrated LLM-based counseling tools by considering implementation costs alongside potential reductions in preventable recurrence-related health care service use.

However, higher actionability scores should not necessarily be interpreted as requiring increasingly detailed or prescriptive recommendations. In dietary counseling for recurrent urolithiasis, clinically useful advice must balance specificity with appropriate individualization and safety considerations. Particularly in cases involving comorbidities or competing clinical priorities, a cautious and appropriately constrained recommendation may be preferable to a more extensive but potentially unsuitable dietary plan.

At the same time, the results should not be interpreted as support for fully autonomous dietary management. The outputs evaluated in this study were generated in a simulated setting and should be viewed as decision support candidates rather than stand-alone clinical advice.

### Safety Considerations

Safety is a particularly important dimension in dietary counseling for recurrent urolithiasis because recommendations that appear broadly reasonable in general stone prevention may become inappropriate when important clinical modifiers are present, such as chronic kidney disease, pregnancy, infection-related stones, advanced age, or cardiovascular comorbidity. In this context, the apparent advantage of StoneAgent is less about generating longer or more detailed responses and more about preserving case-specific clinical constraints when translating metabolic findings into advice. Although the safety comparisons in this study did not reach conventional statistical significance, the directional pattern across both experimental phases suggests that explicit safety-oriented scaffolding may be valuable in specialty counseling tasks where otherwise standard recommendations require contextual modification [6,7,19,20,24-26].

The potential risk of unguided LLM use should also be considered. Although general-purpose LLMs can provide broadly appropriate health information, responses generated without clinical constraints may fail to prioritize the dominant metabolic abnormality, provide overly generalized dietary advice, or recommend modifications that are inappropriate for patients with specific comorbidities. In recurrent stone disease, such limitations could theoretically contribute to ineffective prevention strategies, unnecessary dietary restriction, or delayed recognition of conditions requiring specialist evaluation. Therefore, future clinical applications of LLMs in specialty counseling should incorporate explicit safety frameworks, domain-specific validation, and appropriate human oversight.

### Limitations

Several limitations should be considered. First, this study was based on synthetic clinical vignettes rather than real patient encounters. Although the cases were designed to reflect clinically plausible and guideline-relevant metabolic stone scenarios, they cannot capture the full heterogeneity, ambiguity, and communication variability of real-world practice. Second, the patient query–style inputs were derived from the structured vignettes rather than authored by patients themselves and therefore represent a pragmatic approximation of real consultation language rather than a true external validation set. Third, the study was designed as an exploratory in silico benchmarking study using purposively constructed cases to maximize coverage of guideline-relevant and safety-sensitive scenarios, rather than as a power-calculated confirmatory trial. Fourth, the study compared 2 interaction frameworks implemented through the same publicly available web interface of the underlying LLM. Because that interface does not provide API-level control over generation parameters or fixed model snapshot identifiers, the findings should be interpreted as an operational comparative evaluation under real-world usage conditions rather than as a fully parameter-controlled benchmark. Fifth, the study focused specifically on dietary counseling and did not evaluate broader management decisions such as pharmacologic prevention, imaging follow-up, or procedural planning. Finally, response quality was assessed against expert-derived standards rather than real patient outcomes, and the study evaluated isolated response generation rather than longitudinal patient-model interactions. Therefore, the findings do not establish clinical effectiveness, adherence, recurrence reduction, conversational adaptation, or long-term clinical integration.

Additionally, because the intervention being evaluated was a structured prompting framework, the observed performance improvement may partly reflect the contribution of prompt design and task-specific scaffolding rather than the language model alone. Future studies using controlled prompt-ablation designs could help clarify the relative contribution of individual framework components.

### Future Directions

Future work should evaluate this type of framework using real patient-authored queries, external clinician reviewers, and prospective clinical workflows. Future evaluations should also examine interactive use cases rather than single-turn responses alone, including how guideline-integrated frameworks respond to additional patient questions, clarification requests, newly available laboratory information, or changes in clinical context over time. Future evaluations should also include patients or simulated cases with multiple concurrent comorbidities to determine whether the framework can appropriately reconcile competing or interacting safety constraints when generating dietary recommendations. Future development could also distinguish between clinician-facing and patient-facing implementations of guideline-integrated LLM frameworks. A clinician-facing version could support interpretation of metabolic findings and preparation of individualized counseling plans within clinical workflows, with subsequent evaluation against patient outcomes. A patient-facing version could support longitudinal reinforcement of clinician-provided recommendations, respond to follow-up questions, and adapt counseling as symptoms, laboratory findings, or clinical circumstances change between formal health care encounters. These complementary use cases warrant separate evaluation of usability, safety, and clinical effectiveness. Qualitative studies involving patients and clinicians, such as interviews or focus groups with individuals who have experience with recurrent stone disease, may provide important insights into usability, trust, communication quality, and barriers to adoption. Comparative evaluation across newer foundation models and alternative guideline-based frameworks would also help clarify which components of the observed benefit are attributable to framework design and which may diminish as baseline model performance improves.

### Conclusions

In this in silico comparative study, a guideline-integrated, safety-aware clinical interaction framework generated higher-quality dietary recommendations for recurrent urolithiasis than a standard general-purpose LLM interaction when both were implemented on the same underlying model. This advantage was retained, although in a more conservative form, when the same cases were presented as patient query–style inputs. These findings support the value of explicit clinical framing, guideline grounding, and safety-oriented scaffolding for specialty counseling tasks involving metabolic stone prevention. Further evaluation is needed using real patient-authored queries, external reviewers, and prospective clinical settings.

## Supplementary material

10.2196/95162 Multimedia Appendix 1 Synthetic clinical vignette dataset.

10.2196/95162 Multimedia Appendix 2 Expert reference standards for the synthetic clinical vignettes.

10.2196/95162 Multimedia Appendix 3 Full prompts and model outputs for the structured-input experiment.

10.2196/95162 Multimedia Appendix 4 Patient query–style input variants and corresponding model outputs.

10.2196/95162 Multimedia Appendix 5 Reviewer scoring dataset and scoring rubric.

## Abbreviations

CUA: Canadian Urological Association
EAU: European Association of Urology
ICC: intraclass correlation coefficient
LLM: large language model
