# Supplementary material for: Evaluating a Guideline-Integrated Clinical Interaction Framework Vs a Standard Large Language Model Interaction for Dietary Recommendations in Recurrent Urolithiasis: In Silico Study
Source: J Med Internet Res. 2026 Sep 4;28:e95162. doi: 10.2196/95162 (PMC13544699; doi:10.2196/95162)
Supplement: Supplementary file 2 [file jmir-v28-e95162-s003.docx]

**Agent Prompt：**

\# Role Definition

You are an expert Academic Endourologist and Renal Dietitian specializing in the metabolic management of urolithiasis. Your goal is to provide highly personalized, evidence-based dietary prevention strategies based on the EAU (European Association of Urology) and CUA (Canadian Urological Association) Guidelines.

\# Task

I will provide you with a patient's profile, including stone history, comorbidities, and 24-hour urine metabolic evaluation data.

You must interpret this data to identify the specific metabolic abnormalities (e.g., Hypercalciuria, Hypocitraturia, Hyperuricosuria, etc.).

\# Analysis Logic (Chain of Thought)

1. Risk Identification: Analyze the 24h urine values. Flag any values outside the normal reference range.

2. Etiology Link: Connect the abnormal urine parameter to dietary habits (e.g., High Urine Calcium is often caused by High Sodium intake, not just dietary calcium).

3. Safety Check: Review comorbidities. (e.g., If the patient has CKD or Heart Failure, do NOT recommend excessive fluid intake or high-potassium foods without a warning).

4. Actionable Advice: Convert the medical findings into specific food choices.

\# Output Format

Please structure your response exactly as follows:

1. Metabolic Diagnosis: (e.g., "The patient has absorptive hypercalciuria driven by high sodium intake.")

2. Key Dietary Modifications: (List 3 top priorities. Be specific. Do not just say "eat healthy".)

3. Sample Menu Suggestions: (Give examples of specific foods to eat and foods to avoid based on the specific metabolic profile).

4. Safety Warning: (If applicable based on comorbidities).

\# Constraint

Do NOT recommend restricting dietary calcium for Calcium Oxalate stone formers unless there is specific evidence, as this increases oxalate absorption. Focus on Sodium and Protein restrictions first for hypercalciuria.

**General-purpose LLM:**

"I have kidney stones and I want to prevent recurrence. Here is my medical information: [Insert Vignette Data]. What should I eat? Please give me advice."

**Case ID: C01**

**Output (Agent):**

1. Metabolic Diagnosis:

Recurrent calcium oxalate stone former with hypercalciuria likely sodium-driven (renal “salt leak” physiology) plus low urine volume increasing CaOx supersaturation risk. Marked hypernatriuria strongly supports dietary sodium as a major driver of the elevated urine calcium.

2. Key Dietary Modifications:

1) Lower sodium aggressively (primary lever for urine calcium):

- Target ≤100 mmol sodium/day (about 2.3 g sodium/day ≈ 5–6 g salt/day). Current urine sodium 260 mmol/day is very high.

- Practical: avoid adding salt; choose “no added salt/low sodium” products; avoid restaurant/fast food most days.

- Rule of thumb: aim for <600 mg sodium/meal and <200 mg per snack .

2) Increase fluids to raise urine volume:

- Aim for urine volume ≥2.5 L/day (typically requires ~ 3.0 L/day fluid intake, more with sweating).

- Distribute: big glass on waking, with each meal, mid‑afternoon, and one before bed (stones often form overnight).

- Prefer water; citrus beverages can help (see menu).

3) Moderate animal protein (helps calciuria and CaOx risk without cutting calcium):

- Target about 0.8–1.0 g/kg/day protein, with a shift toward plant proteins.

- Limit large portions of meat/fish/poultry (e.g., keep to ~4–6 oz / 120–170 g per day total), and avoid “double protein” meals.

3. Sample Menu Suggestions:

Eat / choose more often (low sodium, CaOx-friendly):

- Breakfast: plain oats cooked with milk or calcium-fortified beverage (no added salt) + berries; coffee/tea; water.

- Lunch: homemade salad bowl with olive oil + vinegar (no bottled salty dressing), chickpeas or grilled chicken (unsalted), plenty of vegetables; fruit; water.

- Dinner: baked salmon or tofu with no-salt seasoning (lemon, garlic, pepper, herbs) + rice/quinoa + roasted vegetables; water.

- Stone-preventive beverages: water; lemon/lime juice in water (unsweetened).

- Dairy/calcium: keep normal dietary calcium (e.g., 2–3 servings/day) with meals—milk, yogurt, cheese in modest portions—this helps bind oxalate in the gut (do not restrict calcium).

Avoid / limit (major sodium sources driving hypercalciuria and worsening HTN):

- Processed meats: bacon, deli meats, sausage, jerky.

- Restaurant/fast food, pizza, ramen, instant noodles.

- Packaged salty foods: chips, salted nuts, crackers.

- High-sodium condiments: soy sauce, teriyaki, ketchup-heavy use, pickles, bouillon, seasoning salts.

- “Sports drinks” and electrolyte mixes unless medically indicated (often high sodium).

4. Safety Warning:

With hypertension, sodium reduction is especially important and usually safe. Fluid increase is appropriate unless there is unmentioned heart failure, advanced CKD, or hyponatremia risk —if any of those exist, fluid targets should be individualized with the treating clinician. Also, if using thiazide diuretics for HTN (common), sodium restriction enhances their stone-preventive effect but electrolytes should be monitored by your doctor.

**Output (Control):**

Got it — thanks for sharing the details. I’ll keep this practical and tailored to *calcium oxalate stone prevention* and your labs, not generic fluff.

**Big picture (based on your data)**

- **Low urine volume (1.8 L/day)** → stones concentrate more easily
- **Very high urine sodium (260 mmol/day)** → drives calcium into urine
- **Recurrent CaOx stones + HTN** → diet can help *both* problems at once

The goals are:

1. **Dilute your urine**
2. **Lower urine calcium**
3. **Reduce oxalate absorption**
4. **Protect BP and kidneys long-term**

**1. Fluids: your #1 priority 💧**

You want **urine output ≥ 2.5–3.0 L/day**

**What that means practically**

- Drink **~3–3.5 L fluid/day** (more if sweating/exercising)
- Spread intake across the day
- One glass before bed helps prevent overnight concentration

**Best choices**

- Water (still or sparkling)
- Water with lemon or lime (citrate helps inhibit stones)
- Unsweetened tea (black tea is okay *in moderation*)

**Limit**

- Sugar-sweetened drinks
- Large amounts of cola (phosphoric acid)
- Excess grapefruit juice

**2. Sodium: this is hurting you the most 🧂**

Your urine sodium is **very high** → this increases urinary calcium.

**Target**

- **≤ 100 mmol/day sodium**
  ≈ **2–2.3 g sodium/day**
  ≈ **5–6 g salt/day**

**What to cut hard**

- Processed foods (deli meats, sausages, bacon)
- Restaurant food / fast food
- Chips, crackers, instant noodles
- Sauces: soy sauce, teriyaki, BBQ, salad dressings

**What to do instead**

- Cook at home more
- Use herbs, garlic, pepper, lemon instead of salt
- Choose “no added salt” or low-sodium options

➡️ This will **lower stone risk AND help your hypertension**

**3. Calcium: do NOT restrict it 🥛**

This surprises many people.

**Target**

- **Normal dietary calcium: 1,000–1,200 mg/day**
- From **food**, not supplements (unless your doctor says otherwise)

**Why**

- Dietary calcium binds oxalate in the gut → *less oxalate absorbed*
- Low calcium diets actually **increase CaOx stones**

**Best sources**

- Milk, yogurt, kefir
- Cheese (watch sodium)
- Calcium-fortified plant milks (check labels)

**Timing tip**

- Eat calcium-containing foods **with meals that include oxalate**

**4. Oxalate: reduce, don’t eliminate 🥬**

You don’t need a zero-oxalate diet — just be strategic.

**High-oxalate foods to limit**

- Spinach
- Beets
- Rhubarb
- Almonds & almond butter
- Cashews
- Dark chocolate
- Wheat bran

**Lower-oxalate alternatives**

- Kale, cabbage, bok choy
- Broccoli, cauliflower
- Peanuts instead of almonds
- Rice or oats instead of bran cereals

**Key rule**

Oxalate foods are safer when eaten **with calcium**

**5. Protein: moderate, not high 🍗**

Excess animal protein increases calcium and lowers citrate.

**Target**

- ~**0.8–1.0 g/kg/day**
- For you: roughly **65–80 g/day**

**Better choices**

- Fish
- Poultry
- Plant proteins (beans, lentils — moderate portions)

**Limit**

- Large portions of red meat
- Very high-protein / keto-style diets

**6. Potassium & citrate-rich foods 🍋**

These help protect against stones.

**Eat more**

- Citrus fruits (lemon, lime, orange)
- Bananas
- Avocados
- Potatoes
- Beans and lentils

If stones keep recurring, doctors sometimes prescribe **potassium citrate**, but food is a great first step.

**7. Weight & lifestyle**

- BMI 27 → gradual weight loss helps stone risk
- Exercise is good, just **replace fluids**
- Avoid dehydration during workouts or hot weather

**Example day (stone-friendly)**

**Breakfast**

- Yogurt with oats and berries
- Water + lemon

**Lunch**

- Grilled chicken salad (kale, cucumber, olive oil & lemon)
- Low-salt whole grain bread
- Water

**Snack**

- Banana or apple
- Cheese stick (low sodium)

**Dinner**

- Baked salmon
- Rice
- Steamed broccoli
- Sparkling water with lime

**Evening**

- Glass of water before bed

**The 3 take-home rules**

1. **Drink enough to pee clear**
2. **Slash salt**
3. **Normal calcium with meals**

**Case ID: C02**

**Output (Agent):**

1. Metabolic Diagnosis:

Recurrent calcium oxalate stone former with hypercalciuria (urine calcium 8.5 mmol/day ≈ 340 mg/day). Urine sodium is not markedly elevated (120 mmol/day), so the hypercalciuria is less likely to be primarily sodium-driven and may reflect idiopathic/renal leak hypercalciuria and/or high animal protein acid load. Osteopenia increases concern for bone calcium loss, so prevention should prioritize normal calcium intake, sodium moderation, and protein/alkali optimization, not calcium restriction.

2. Key Dietary Modifications:

1) Maintain normal dietary calcium (bone + stone benefit):

- Aim 1,000–1,200 mg/day calcium from food, spread across meals (e.g., dairy or calcium-fortified options).

- Avoid low-calcium dieting; it can raise oxalate absorption and worsen CaOx risk.

2) Moderate animal protein and increase alkali (supports lower calciuria and bone):

- Target about 0.8–1.0 g/kg/day total protein, emphasizing plant proteins and limiting large meat portions.

- Add fruits/vegetables at each meal (citrate/alkali support; also bone-friendly).

3) Keep sodium modest (don’t “chase” it lower, but avoid creep):

- Current sodium 120 mmol/day is reasonable; aim ~65–100 mmol/day (about 1.5–2.3 g sodium/day) and avoid high-salt processed foods that could push calciuria higher.

3. Sample Menu Suggestions:

Eat / choose more often (CaOx + osteopenia supportive):

- Breakfast: Greek yogurt or milk + oatmeal + berries; or fortified soy milk smoothie with fruit.

- Lunch: lentil/bean soup made low-sodium + whole grain bread; salad with olive oil + vinegar.

- Dinner: tofu/beans or a modest portion of fish/chicken + quinoa/brown rice + roasted vegetables.

- Calcium-with-meals options (stone-smart): milk with meals, yogurt snack, small cheese portion with lunch; calcium-fortified plant milks if dairy intolerant.

- Flavoring instead of salt: lemon, herbs, garlic, pepper, vinegar, salt-free spice blends.

Avoid / limit (tends to worsen hypercalciuria and bone loss):

- Large animal-protein portions (e.g., big steaks, high-protein diets/keto-style plans).

- High-sodium processed foods (even though sodium isn’t extreme now): deli meats, instant noodles, salty snacks.

- Colas (phosphoric acid) if heavily consumed; choose water/seltzer instead.

4. Safety Warning:

With osteopenia, do not restrict dietary calcium. If calcium supplements are used, they should generally be taken with meals (not on an empty stomach) and the need/dose should be reviewed with her clinician given stone history. Also ensure vitamin D and secondary causes of bone loss are evaluated by her treating team (I don’t have her serum labs here).

**Output (Control):**

Thanks for sharing the details — that really helps tailor the advice. I’ll focus on **dietary steps that reduce calcium oxalate (CaOx) stone recurrence** *and* protect your bones, given the osteopenia.

**Big picture goals (for *you* specifically)**

- **Lower urine calcium** (your 8.5 mmol/day is on the high side)
- **Reduce oxalate absorption**
- **Increase urine volume & citrate**
- **Protect bone density** (very important — no low-calcium diets)

**1. Calcium: *Do not* restrict it 🚫🥛**

This is crucial with CaOx stones *and* osteopenia.

**Target:**
👉 **1,000–1,200 mg/day of calcium from food**, spread across meals

**Why:**

- Adequate dietary calcium **binds oxalate in the gut**, lowering stone risk
- Low calcium intake actually **increases stones** *and* worsens bone loss

**Best sources (with meals):**

- Milk, yogurt, kefir
- Cheese (moderate portions)
- Calcium-fortified soy/almond milk
- Tofu set with calcium

⚠️ **Avoid calcium supplements unless your clinician specifically advises them** — especially if taken between meals.

**2. Sodium: this is a major issue for you 🧂⬇️**

Your urine sodium (120 mmol/day) suggests **high salt intake**, which **drives calcium into urine**.

**Goal:**
👉 **< 2 g sodium/day** (~5 g salt)

**Practical tips:**

- Avoid processed foods, fast food, packaged snacks
- Skip “hidden salt”: breads, sauces, soups, deli meats
- Cook at home; use herbs, lemon, vinegar instead of salt
- Check labels: aim for **<140 mg sodium/serving**

📉 Reducing sodium can **significantly lower urine calcium** — often more effective than cutting calcium.

**3. Oxalate: reduce (not eliminate) 🥬**

You don’t need an extreme low-oxalate diet — just be strategic.

**Limit high-oxalate foods:**

- Spinach, Swiss chard, beet greens
- Almonds, cashews
- Rhubarb
- Beets
- Dark chocolate, cocoa
- Wheat bran

**Smart strategy:**
👉 If you eat oxalate-containing foods, **pair them with calcium at the same meal** (e.g., yogurt + berries instead of berries alone).

**4. Fluids: this is non-negotiable 💧**

**Target urine volume:**
👉 **≥ 2.5 liters/day** (usually means ~3 L fluid intake)

**Best choices:**

- Water (mainstay)
- Citrus beverages (lemon/lime water)

🍋 **Citrate helps prevent CaOx stones** — aim for:

- Lemon or lime juice in water (½ cup/day total, diluted)
- Or oranges (whole fruit > juice)

Avoid:

- Sugary drinks
- Excess cola (phosphoric acid)

**5. Protein: moderate, not high 🍗**

Excess animal protein:

- Increases urine calcium
- Lowers citrate

**Aim for:**

- ~0.8–1.0 g/kg/day
- Plant proteins encouraged (lentils, beans — eaten with calcium!)

Limit:

- Large portions of red meat
- Protein supplements unless prescribed

**6. Vitamin C & supplements ⚠️**

- **Avoid high-dose vitamin C** (>500–1000 mg/day) → converts to oxalate
- Be cautious with “bone” or “wellness” supplements that include calcium + vitamin C combos

**7. Bone health synergy 🦴**

What helps stones also helps bones:

- Adequate dietary calcium
- Lower sodium
- Normal protein intake
- Weight-bearing exercise
- Adequate vitamin D (check levels; supplement if low)

**A simple “stone-smart” day example**

- **Breakfast:** Yogurt + berries + oats
- **Lunch:** Lentil salad with feta, olive oil, lemon
- **Snack:** Fruit + cheese
- **Dinner:** Grilled fish, rice, roasted vegetables
- **Fluids:** Water throughout + lemon water

**One last clinical note (not diet, but important)**

With **recurrent CaOx stones + hypercalciuria + osteopenia**, many patients benefit from **thiazide diuretics** — they lower urine calcium *and* protect bone. Worth discussing with your nephrologist/urologist.

If you want, I can:

- Build you a **low-sodium, bone-friendly meal plan**
- Help you **decode food labels**
- Tailor this further if you have urine oxalate or citrate values

You’re asking exactly the right questions — prevention here is very doable 💪🧠

**Case ID: C03**

**Output (Agent):**

1. Metabolic Diagnosis:

Mixed calcium oxalate/calcium phosphate stone former with severe hypocitraturia (urine citrate 0.8 mmol/day is profoundly low). With urine pH 6.0, there is not a strong alkalinization signal yet, but given the CaP component, any strategy that raises urine pH too much could increase calcium phosphate supersaturation. Metabolic syndrome also increases stone risk via diet patterns (high animal protein/sodium, low fruit/veg) and often low citrate.

2. Key Dietary Modifications:

1) Raise citrate primarily through food (alkali) while avoiding excessive pH rise:

- Increase fruits and vegetables daily (citrate/alkali sources).

- Use citrus in food/drinks (lemon/lime/orange), preferably unsweetened.

- Avoid “alkali loading” with large bicarbonate doses unless supervised, because CaP stones can worsen if urine pH climbs too high.

2) Reduce dietary acid load (helps citrate and Ca balance):

- Moderate animal protein (avoid very high-protein/low-carb patterns).

- Shift protein mix toward plant proteins (beans, lentils, soy, nuts in reasonable portions).

3) Metabolic-syndrome focused changes that also reduce stone risk:

- Cut sugar-sweetened beverages and refined carbs (also helps weight/insulin resistance).

- Choose high-fiber carbs (vegetables, legumes, intact whole grains) and healthy fats (olive oil, nuts).

3. Sample Menu Suggestions:

Eat / choose more often (to raise citrate without sugary load):

- Morning: water + lemon/lime juice (unsweetened) or plain seltzer with citrus.

- Breakfast: eggs or tofu scramble + vegetables; or plain Greek yogurt + berries + nuts.

- Lunch: big salad with olive oil + vinegar, grilled chicken or chickpeas; add citrus segments or a squeeze of lemon.

- Dinner: baked fish or tofu + roasted vegetables + a small portion of brown rice/quinoa.

- Snacks: fruit (citrus, melon), vegetables with hummus, unsalted nuts (portion-controlled).

Avoid / limit (worsens citrate/MetS and can push CaP risk indirectly):

- Sugar-sweetened sodas/juices, sweet tea, energy drinks.

- Very high animal-protein meals (large steaks, frequent processed meats).

- Highly processed salty foods (often paired with refined carbs).

4. Safety Warning:

Because he forms calcium phosphate stones, be cautious with interventions that significantly raise urine pH (for example, large amounts of alkali supplements). Food-based citrate strategies are generally safer, but if pharmacologic citrate (e.g., potassium citrate) is considered, it should be monitored with repeat 24‑hour urine (especially pH and CaP supersaturation) to avoid shifting risk toward more CaP stone growth. Metabolic syndrome also raises cardiovascular risk, so avoid high-sugar “citrate sources” (like sweetened lemonade).

**Output (Control):**

Got it — thanks for sharing the details. I’ll tailor this to **mixed calcium oxalate (CaOx) + calcium phosphate (CaP) stones**, **low urine citrate**, **urine pH ~6.0**, and **metabolic syndrome**.

Below is **evidence-based, practical nutrition advice** to reduce recurrence. Think of this as a *stone-prevention eating pattern*, not a short-term diet.

**1. Hydration (this is non-negotiable 🚰)**

**Goal:** urine volume **≥ 2.5 L/day**
That usually means **3–3.5 L of fluids daily**

**Best choices**

- Water (still or sparkling)
- Water with lemon or lime (natural citrate)
- Unsweetened herbal tea

**Limit / avoid**

- Sugar-sweetened beverages (soda, juice)
- Cola drinks (phosphoric acid ↑ CaP risk)

💡 Your urine should be **very pale yellow** most of the day.

**2. Boost Citrate (yours is low)**

Low citrate removes a key natural stone inhibitor.

**Eat more alkali-producing foods**

- **Citrus**: lemon, lime, orange (whole fruit > juice)
- Melons
- Avocado
- Vegetables (especially leafy greens)

**Practical trick**

- **½–1 cup lemon juice/day** diluted in water
  (or multiple lemons squeezed across the day)

⚠️ Avoid potassium supplements unless prescribed — food sources are preferred.

**3. Calcium: Don’t Cut It — Use It Strategically 🦴**

**Target:** ~1,000–1,200 mg/day **from food**

**Best sources (take WITH meals)**

- Low-fat milk, yogurt
- Cheese (moderate portions)
- Calcium-fortified unsweetened plant milks

Why?

- Dietary calcium **binds oxalate in the gut**, reducing CaOx stones
- Low calcium diets **increase stone risk**

🚫 Avoid calcium supplements unless your doctor insists — they raise stone risk if taken incorrectly.

**4. Oxalate: Moderate, Not Zero**

Since your stones include CaOx:

**Limit (especially without calcium present)**

- Spinach
- Beets
- Almonds
- Rhubarb
- Swiss chard
- Large amounts of dark chocolate

**Tips**

- Pair oxalate foods with calcium (e.g., yogurt + berries)
- Boiling vegetables lowers oxalate content

You do **not** need a zero-oxalate diet — that’s unnecessary and miserable.

**5. Sodium: This Is Huge for You 🧂**

High sodium increases urinary calcium.

**Goal:**
👉 **< 2,000 mg sodium/day**
(ideal: ~1,500 mg given metabolic syndrome)

**Reduce**

- Processed foods
- Restaurant meals
- Cured meats
- Packaged snacks

**Flavor instead with**

- Herbs
- Lemon
- Vinegar
- Garlic

**6. Protein: Moderate, Lean, Balanced 🍗**

Excess animal protein:

- ↑ urinary calcium
- ↓ citrate
- ↑ acid load

**Aim for**

- ~0.8–1.0 g/kg/day
- More plant protein, less red meat

**Better choices**

- Fish
- Poultry
- Beans & lentils (moderate portions)
- Tofu

**Limit**

- Red meat
- Processed meat
- Very high-protein diets (keto, carnivore = 🚫)

**7. Sugar & Refined Carbs (Metabolic Syndrome tie-in)**

Insulin resistance worsens stone risk.

**Minimize**

- Sugary drinks
- White bread, pastries
- Large juice portions

**Choose**

- Whole grains (moderate)
- Vegetables
- Berries
- Healthy fats (olive oil, nuts in moderation)

This helps **stones, weight, BP, and diabetes risk** all at once.

**8. Watch the Urine pH Balance ⚖️**

Your pH (~6.0) is *borderline*:

- Too acidic → CaOx risk
- Too alkaline (>6.5) → CaP risk

That’s why we:

- Increase **citrate via food**
- Avoid aggressive alkalinization unless prescribed

(Your clinician may still consider potassium citrate, but diet comes first.)

**A Sample “Stone-Smart” Day 🍽️**

**Breakfast**

- Greek yogurt + berries
- Oatmeal
- Lemon water

**Lunch**

- Grilled chicken salad
- Olive oil + lemon dressing
- Whole-grain bread (small portion)

**Snack**

- Orange or melon

**Dinner**

- Salmon
- Steamed broccoli
- Brown rice (small portion)

**Fluids**

- Water throughout the day
- Lemon water in afternoon/evening

**Big Picture Summary**

**Most important levers for you**

1. High urine volume
2. Lower sodium
3. Normal dietary calcium
4. Higher citrate intake
5. Moderate animal protein
6. Metabolic-friendly eating

If you want, I can:

- Build a **7-day stone-prevention meal plan**
- Help interpret a **24-hour urine panel**
- Adjust advice if you’re considering meds like potassium citrate

Just tell me 👍

**Case ID: C04**

**Output (Agent):**

1. Metabolic Diagnosis:

Calcium oxalate stone former with hyperoxaluria (urine oxalate 0.9 mmol/day ≈ 80 mg/day, clearly elevated) with normocalciuria. Pattern is most consistent with dietary hyperoxaluria and/or inadequate calcium with meals (reduced intestinal oxalate binding).

2. Key Dietary Modifications:

1) Lower high-oxalate foods (targeted restriction, not “everything”):

- Strongly limit/avoid: spinach, beet greens, rhubarb, almonds/almond flour, cashews, peanuts, sesame/tahini, bran, buckwheat, dark chocolate/cocoa, and large portions of beets.

- Keep “moderate oxalate” foods to reasonable portions rather than daily large servings.

2) Ensure normal calcium intake and pair calcium with oxalate-containing meals:

- Aim 1,000–1,200 mg/day dietary calcium, spread with meals.

- Practical pairing: if eating salad/veg/grains that contain oxalate, include milk/yogurt/cheese (or calcium-fortified alternatives) with that meal to bind oxalate in the gut.

3) Hydration to dilute oxalate load:

- Aim for urine volume ≥2.5 L/day (often ~3 L/day fluid intake depending on activity/sweat).

- Water is best; avoid high-dose vitamin C drinks/supplements.

3. Sample Menu Suggestions:

Eat / choose more often (low-oxalate pattern with calcium at meals):

- Breakfast: oatmeal made with milk + berries; or eggs + low-oxalate vegetables (mushrooms, peppers) + yogurt.

- Lunch: turkey/chicken sandwich with cheese; side of cucumber/tomato salad (no spinach) + fruit.

- Dinner: rice or pasta + fish/chicken + cauliflower/zucchini/green beans; add a dairy serving if the meal includes legumes/grains.

- Snacks: yogurt, cheese with crackers, fresh fruit; pumpkin seeds or walnuts/pecans (generally lower than almonds/cashews) in modest portions.

Avoid / limit (common hyperoxaluria drivers):

- Spinach smoothies / “green juices” with spinach/beet greens.

- Almond milk as a staple beverage, almond flour baked goods, large handfuls of almonds/cashews.

- Dark chocolate/cocoa-heavy foods daily.

- Vitamin C supplements (especially ≥500–1000 mg/day), which can increase oxalate.

4. Safety Warning:

No comorbidity-specific restrictions noted. Main caution: do not restrict dietary calcium to “lower stones”—that can worsen hyperoxaluria and CaOx risk. If he has chronic diarrhea, bowel disease, or prior bariatric surgery (not reported), the approach would shift toward enteric hyperoxaluria strategies.

**Output (Control):**

Got it — thanks for the clear details. I’ll tailor this specifically to **calcium oxalate (CaOx) stone prevention** with **elevated urine oxalate** and **normal urine calcium**. You’re in a good position to prevent recurrence 👍

**Big Picture (what matters most)**

For CaOx stones, the highest-impact levers are:

1. **High fluid intake**
2. **Lower oxalate exposure**
3. **Adequate (not low!) dietary calcium**
4. **Smart pairing of foods**
5. **Moderate sodium & animal protein**

I’ll translate that into exactly *what to eat and avoid*.

**1. Fluids (non-negotiable)**

**Goal:** produce **≥2.5 L urine/day**
That usually means drinking **~3 L (100 oz) fluid/day**

**Best choices**

- Water (still or sparkling)
- Water with lemon or lime 🍋 (citrate helps inhibit stones)
- Unsweetened herbal teas (not black tea)

**Limit**

- Sugary drinks
- Cola (phosphoric acid)
- Excess black tea or iced tea (high oxalate)

**2. Oxalate: lower the load (key for you)**

Your urine oxalate is elevated, so this is crucial.

**High-oxalate foods → avoid or strictly limit**

- Spinach (biggest offender 🚨)
- Beets
- Rhubarb
- Swiss chard
- Almonds, cashews
- Peanuts, peanut butter
- Dark chocolate, cocoa
- Black tea
- Wheat bran
- Sweet potatoes

You don’t have to be zero-oxalate — just **strategic and consistent**.

**Lower-oxalate alternatives (swap these in)**

- Greens: **kale, romaine, iceberg, arugula**
- Veg: broccoli, cauliflower, zucchini, cucumbers
- Fruit: bananas, apples, pears, berries (moderate)
- Nuts: **pistachios, walnuts** (small amounts)

**3. Calcium: don’t cut it out ❗**

This is a common mistake.

**Goal:** ~**1,000–1,200 mg/day from food**

Calcium binds oxalate **in the gut**, preventing absorption.

**Best calcium sources (with meals)**

- Milk or lactose-free milk
- Yogurt (plain/Greek)
- Cheese
- Calcium-fortified plant milks (check labels)

**Timing matters:**
👉 Eat calcium **with oxalate-containing meals**, not separately.

🚫 Avoid calcium supplements unless your doctor specifically advises them.

**4. Sodium: keep it moderate**

High sodium increases urinary calcium (even if labs are normal now).

**Target:**

- **<2,300 mg/day** (ideally closer to 1,800)

**Limit**

- Fast food
- Processed meats
- Chips, salty snacks
- Restaurant sauces

**5. Protein: quality over quantity**

You don’t need to be low-protein, just **not excessive**.

**Aim:** ~0.8–1.0 g/kg/day

**Better choices**

- Fish
- Poultry
- Eggs
- Plant proteins (lentils, tofu — moderate oxalate but okay in balance)

**Limit**

- Large daily portions of red meat
- Protein mega-supplements

**6. Vitamin C & supplements**

- **Avoid high-dose vitamin C (>500 mg/day)**
  → converts to oxalate
- Magnesium supplements may help some people, but food first

**A simple day of eating (example)**

**Breakfast**

- Greek yogurt with berries
- Oatmeal (not wheat bran)
- Water + lemon

**Lunch**

- Grilled chicken salad (romaine, cucumber, olive oil)
- Cheese or yogurt on the side

**Snack**

- Apple + small handful walnuts

**Dinner**

- Salmon
- Rice or quinoa
- Steamed broccoli
- Glass of milk or fortified alternative

**Extra habits that help**

- Spread protein across the day
- Eat oxalate foods *with* calcium, not alone
- Don’t restrict calcium to “protect kidneys” — it backfires

If you want, I can:

- Build a **7-day kidney-stone-safe meal plan**
- Give you a **“safe vs risky foods” cheat sheet**
- Help you fine-tune this for eating out or travel

You’re doing the right thing tackling this early — recurrence prevention works when you’re this targeted 💪🫗

**Case ID: C05**

**Output (Agent):**

1. Metabolic Diagnosis:

Recurrent uric acid stone former with persistently acidic urine (pH 5.1, major driver of uric acid crystallization). Urine uric acid 5.8 mmol/day (≈ 980 mg/day) is at least borderline-high, and with gout suggests a high purine load and/or insulin resistance physiology, but the dominant correctable abnormality for uric acid stones is the low urine pH.

2. Key Dietary Modifications:

1) Alkali strategy to raise urine pH (diet-first, consistent daily):

- Increase fruits and vegetables (alkali load) at every meal.

- Use citrus (lemon/lime/orange) in water/food; keep it unsweetened.

- Goal conceptually is to bring urine pH up into a safer range for uric acid (often ~6.0–6.5 clinically), but with diet alone, focus on daily consistency.

2) Lower purine load (helps gout and may reduce uric acid excretion):

- Limit organ meats (liver), anchovies/sardines, mussels/scallops, and large portions of red meat.

- Keep meat/fish portions modest; shift toward plant proteins and low-fat dairy (dairy is generally favorable for urate).

3) Hydration (reduce uric acid concentration):

- Target urine volume ≥2.5 L/day (often ~3 L/day fluid intake; more if sweating).

- Prefer water; avoid sugar-sweetened beverages.

3. Sample Menu Suggestions:

Eat / choose more often (raise pH + gout-friendly):

- Breakfast: low-fat yogurt with berries + oats; water with lemon.

- Lunch: large salad/vegetable bowl with olive oil + vinegar; add tofu/beans or a small portion of chicken; fruit.

- Dinner: stir-fry vegetables + tofu, or baked fish (moderate portion) + potatoes/rice + vegetables.

- Beverages: water, seltzer, unsweetened coffee/tea; citrus-infused water.

- Dairy: milk/yogurt as tolerated (often helpful for urate compared with meat-based proteins).

Avoid / limit (uric acid + gout triggers):

- Beer and spirits-heavy drinking patterns (beer is particularly purine-rich and gout-provoking).

- Fructose-heavy drinks/foods: regular soda, sweetened juices, sweet tea, many energy drinks.

- High-purine items: organ meats, anchovies/sardines, shellfish; frequent large red-meat portions.

4. Safety Warning:

With gout, aggressive weight-loss crash diets and very high-protein / “keto” patterns can worsen uric acid and stone risk; aim for gradual, sustainable changes. If pharmacologic urine alkalinization (commonly potassium citrate) is used, it should be coordinated with his clinician—especially if there is any unreported CKD or use of ACE inhibitors/ARBs or potassium-sparing diuretics, which can increase hyperkalemia risk.

**Output (Control):**

Thanks for sharing the details—that really helps. Given your history (recurrent **uric acid stones**, low **urine pH 5.1**, and **gout**), prevention is very doable with the right food pattern and habits. I’ll keep this practical.

**Big Picture (what matters most for uric acid stones)**

Uric acid stones form mainly because:

1. **Urine is too acidic**
2. **Uric acid load is high**
3. **Urine volume is low**

So the goals are to:

- **Alkalinize your urine** (target pH ~6.0–6.5)
- **Lower dietary purines**
- **Increase urine volume**

Diet plays a *huge* role in all three.

**What to Eat More Of ✅**

**🥦 Plant-Forward Foods (very important)**

These help raise urine pH and reduce uric acid:

- Vegetables (especially leafy greens, broccoli, zucchini, peppers, cucumbers)
- Potatoes, sweet potatoes
- Fruits: cherries, oranges, lemons, limes, melons, berries
- Whole grains (oats, brown rice, quinoa)

👉 **Citrus fruits** are especially helpful—lemon and lime juice alkalinize urine (even though they taste acidic).

**🥛 Low-Fat Dairy**

- Skim or 1% milk
- Yogurt
- Low-fat cheese

These are associated with **lower stone risk** and are safe for gout.

**💧 Fluids (non-negotiable)**

- Aim for **at least 2.5–3 liters/day** unless your doctor limits fluids
- Best choices:
  - Water
  - Lemon water (½ cup lemon juice spread through the day)
  - Unsweetened herbal teas

Your urine should be **pale yellow to clear** most of the day.

**What to Limit or Avoid ⚠️**

**🥩 High-Purine Animal Proteins (key trigger)**

These raise uric acid and acidify urine:

- Red meat (beef, lamb, pork)
- Organ meats (liver, kidney)
- Game meats
- Sardines, anchovies, shellfish
- Meat gravies, broths, stock cubes

**Target:**

- Keep animal protein to **~0.8 g/kg/day**
- Prefer **plant protein** (beans, lentils, tofu) instead

**🍺 Alcohol (especially beer)**

- Beer is one of the **worst offenders** for uric acid
- Spirits and wine are better than beer, but still increase risk

If you drink:

- Avoid beer
- Keep intake minimal and well-hydrated

**🧃 Sugar & Fructose**

Fructose raises uric acid:

- Soda (even fruit juice)
- Sweetened drinks
- Candy, pastries, syrups

Whole fruit is fine—**juice is not**.

**Special Notes for Gout + Stones**

- **Coffee** (1–2 cups/day) is actually associated with *lower* uric acid—okay unless contraindicated for you.
- **Vitamin C from food** is helpful; avoid high-dose supplements unless advised.
- Don’t severely restrict protein—just shift the *source*.

**Sample Day (simple & realistic)**

**Breakfast**

- Oatmeal with berries and low-fat milk
- Coffee or tea
- Water with lemon

**Lunch**

- Large salad with olive oil & lemon dressing
- Grilled tofu or chickpeas
- Whole-grain bread

**Snack**

- Yogurt + fruit
- Handful of nuts

**Dinner**

- Baked salmon *or* lentil stew (fish okay 1–2×/week)
- Roasted vegetables
- Brown rice or quinoa

**All day:** water, water, water 💧

**Medical Therapy (worth discussing with your doctor)**

Diet helps a lot, but with urine pH 5.1:

- **Urine alkalinization (e.g., potassium citrate)** is often the *most effective* prevention
- **Allopurinol** may be indicated given gout + uric acid stones

Diet + meds together = best protection.

If you want, I can:

- Build a **1-week meal plan**
- Help you track **urine pH with food choices**
- Translate this into a **“kidney stone grocery list”**

Just say the word.

**Case ID: C06**

**Output (Agent):**

1. Metabolic Diagnosis:

Recurrent calcium oxalate stone former with severe low urine volume (0.9 L/day) causing high urinary concentration and increased CaOx supersaturation. No other metabolic parameters provided, but this alone is a major, actionable driver.

2. Key Dietary Modifications:

1) Increase fluid intake to achieve protective urine volume:

- Target urine volume ≥2.5 L/day (often requires ~3.0 L/day fluid intake, more with heat/exercise).

- Structure it: ~500 mL on waking, ~500 mL mid‑morning, ~500 mL afternoon, ~500 mL with dinner, ~250–500 mL before bed (adjust to lifestyle).

2) Choose fluids that reduce stone risk (and avoid sugar load):

- Best: water and unsweetened sparkling water.

- Helpful option: citrus in water (lemon/lime) to support citrate, ideally without sugar.

- Avoid routine sugar-sweetened beverages (they can worsen stone risk and weight).

3) Pair hydration with sodium awareness (prevents “thirsty but salty” cycle):

- Keep processed salty foods lower so increased drinking doesn’t come with increased sodium intake (which can raise urine calcium).

3. Sample Menu Suggestions:

Eat / drink more often:

- Carry a 1 L bottle: finish 1 bottle by lunch and 1 by dinner + an additional glass evening/bedtime.

- Water or seltzer with lemon/lime; unsweetened tea/coffee in moderation.

- High-water foods to support totals: soups (not salty), cucumbers, watermelon, oranges.

Avoid / limit:

- Skipping fluids for long stretches (common cause of very low urine volume).

- Sugar-sweetened soda/juice “to meet fluid goals.”

- Very salty snacks/fast food that drive thirst and can worsen calciuria.

4. Safety Warning:

No comorbidities listed that would usually limit fluids. If she has unreported heart failure, advanced kidney disease, or hyponatremia history, fluid goals should be individualized with her clinician.

**Output (Control):**

Got it — thanks for sharing the details. I’ll tailor this specifically to **recurrent calcium oxalate (CaOx) stones** and your labs.

The **single biggest risk factor** jumping out is your **very low urine volume (0.9 L/day)**, so we’ll start there, then fine-tune diet to reduce calcium oxalate formation.

**1️⃣ Fluids (this is the #1 priority)**

Your goal is **urine output ≥ 2.5 L/day**.

**What that means practically**

- Drink **~3 L (100 oz) fluids daily** (more if sweating)
- Spread intake evenly throughout the day
- Include a glass **before bed** and if you wake overnight

**Best choices**

- Water (still or sparkling)
- Lemon or lime water 🍋 (natural citrate helps prevent stones)
- Unsweetened iced tea *in moderation*

**Limit / avoid**

- Sugary drinks (increase stone risk)
- Grapefruit juice (linked to higher stone risk)
- Excess cola (phosphoric acid)

👉 If you do nothing else but increase fluids, you’ll dramatically reduce recurrence risk.

**2️⃣ Calcium: Do NOT restrict**

This is a common mistake.

**Target:**

- **1,000–1,200 mg/day from food**

**Why:**
Dietary calcium **binds oxalate in the gut**, preventing absorption.

**Best sources**

- Milk, yogurt, kefir
- Cheese (watch sodium)
- Calcium-fortified plant milks (check labels)

⚠️ Avoid calcium supplements unless specifically prescribed — they can increase stone risk if taken without meals.

**3️⃣ Oxalate: Reduce *high* sources (not zero)**

You don’t need an oxalate-free diet — just be strategic.

**High-oxalate foods to limit**

- Spinach 🚫
- Beets
- Rhubarb
- Almonds & almond flour
- Cashews
- Dark chocolate
- Wheat bran
- Sweet potatoes

**Better swaps**

- Kale, arugula, romaine
- Broccoli, cauliflower
- White potatoes
- Peanuts (lower oxalate than tree nuts)

💡 Tip: **Eat oxalate foods WITH calcium-containing foods** to block absorption.

**4️⃣ Sodium: Keep it low**

High sodium increases urinary calcium.

**Goal:**

- **< 2,000 mg sodium/day**

**Watch out for**

- Restaurant food
- Processed meats
- Packaged snacks
- Sauces & dressings

Lower sodium = lower calcium in urine = fewer stones.

**5️⃣ Protein: Moderate, not high**

Excess animal protein increases calcium and lowers citrate.

**Target**

- ~0.8–1.0 g/kg/day (not keto / high-protein)

**Best choices**

- Fish
- Poultry
- Eggs
- Plant proteins (lentils, beans — in moderation)

Try to limit:

- Large portions of red meat
- Protein powders unless advised

**6️⃣ Citrate: A natural stone inhibitor**

Low citrate is common in CaOx stone formers.

**Boost citrate naturally**

- Lemon or lime juice (½ cup daily diluted)
- Or 2–3 tbsp lemon juice in water twice daily
- Fruits: oranges, melon

(Some patients need potassium citrate medication — your doctor would decide based on 24-hr urine.)

**7️⃣ Weight & insulin resistance (important but gradual)**

BMI 35 is associated with:

- Lower urine pH
- Higher stone risk

**Focus on**

- Slow, sustainable weight loss
- Mediterranean-style eating
- Avoid crash dieting or keto (stone-promoting)

**8️⃣ What a “stone-smart” day could look like**

**Breakfast**

- Greek yogurt with berries
- Water with lemon

**Lunch**

- Grilled chicken salad (romaine, cucumber, olive oil)
- Whole-grain roll
- Water

**Snack**

- Cheese + apple

**Dinner**

- Baked salmon
- Rice
- Roasted broccoli
- Sparkling water with lime

**Evening**

- Glass of water before bed

**Bottom line (key takeaways)**

If you remember only 4 things:

1. **Drink enough to pee >2.5 L/day**
2. **Eat normal calcium with meals**
3. **Lower sodium**
4. **Limit high-oxalate foods (especially spinach & almonds)**

If you want, I can:

- Build a **7-day meal plan**
- Help you track **fluid goals**
- Translate this into a **grocery list**
- Or tailor it to vegetarian, lactose-free, or cultural preferences

Just say the word 💧🪨

**Case ID: C07**

**Output (Agent):**

1. Metabolic Diagnosis:

Mixed calcium oxalate and uric acid stone former with mild–moderate hypercalciuria (urine calcium 7.8 mmol/day ≈ 310 mg/day) and borderline hyperuricosuria (uric acid 5.2 mmol/day ≈ 875 mg/day). This profile suggests a combined risk from calcium load in urine (often amplified by sodium and animal-protein intake) plus higher purine/uric acid production. Urine pH and volume are not provided, but for mixed CaOx/uric acid stones, low urine pH and low volume are common contributors.

2. Key Dietary Modifications:

1) Reduce sodium (first-line lever for hypercalciuria):

- Target ≤100 mmol/day sodium (≈ 2.3 g sodium/day).

- Prioritize cutting processed foods/restaurant meals; avoid salty condiments and snacks.

2) Moderate animal protein and purines (helps both calcium and uric acid risks):

- Aim 0.8–1.0 g/kg/day protein, shift toward plant proteins and low-fat dairy.

- Limit high-purine items (organ meats, anchovies/sardines, many shellfish) and keep meat portions modest.

3) Hydration + sugar avoidance (covers both stone types):

- Aim for urine volume ≥2.5 L/day (often ~3 L/day intake).

- Avoid sugar-sweetened drinks (raise uric acid and stone risk).

3. Sample Menu Suggestions:

Eat / choose more often (covers hypercalciuria + uric acid risk):

- Breakfast: oatmeal with milk or yogurt + fruit; water/coffee.

- Lunch: lentil/bean bowl or salad with olive oil + vinegar; add a small portion of chicken/fish if desired; fruit.

- Dinner: tofu/vegetable stir-fry or baked fish + vegetables + rice/quinoa; water with lemon/lime.

- Snacks: yogurt, fruit, unsalted nuts (portion-controlled), vegetables with hummus.

- Calcium: keep normal dietary calcium (about 1,000–1,200 mg/day from food, with meals), not restricted.

Avoid / limit:

- Processed salty foods: deli meats, pizza, fast food, chips, instant noodles.

- High-purine foods: organ meats; frequent sardines/anchovies; shellfish-heavy meals.

- Sugar-sweetened beverages and frequent fructose-heavy snacks.

4. Safety Warning:

No comorbidities listed that restrict these recommendations. Because urine pH and volume (and urine sodium) weren’t provided, the most important missing discriminator is whether he also has low urine pH (key for uric acid stones). If pH is low, diet may help, but many patients require clinician-guided alkalinization; repeat 24-hour urine testing is important to avoid undertreating uric acid risk.

**Output (Control):**

Got it. With **mixed calcium oxalate + uric acid stones**, the goal is a diet that **dilutes urine, lowers calcium/uric acid load, reduces oxalate absorption, and raises urine citrate/pH**. Here’s a practical, evidence-based eating plan tailored to your numbers and profile.

**1️⃣ Fluids (this is the #1 intervention)**

**Target:** enough to make **≥2.5–3.0 L of urine/day**
➡️ Usually means **~3 L (100 oz) fluid intake daily**, spread out

**Best choices**

- Water (still or sparkling)
- Lemon or lime water (natural citrate ↑)
- Unsweetened herbal tea

**Limit / avoid**

- Sugar-sweetened beverages
- Cola (phosphoric acid)
- Excess fruit juice (esp. apple, grape)

📌 Tip: Add **½ cup lemon juice/day** diluted in water if tolerated.

**2️⃣ Calcium: Don’t cut it — eat it *right***

Your urine calcium is **moderately elevated**, but **low calcium diets increase stone risk**.

**Target:** **1,000–1,200 mg/day from food**

**Best sources (with meals)**

- Yogurt, milk, kefir
- Cheese (moderate amounts)
- Calcium-fortified plant milks

🚫 Avoid calcium supplements unless prescribed
✔️ Calcium with meals binds oxalate in the gut → **less stone formation**

**3️⃣ Oxalate: Reduce high-load foods (not everything!)**

Focus on **cutting the big offenders**, not total elimination.

**Limit or avoid**

- Spinach, Swiss chard
- Beets
- Rhubarb
- Almonds, cashews
- Dark chocolate
- Wheat bran
- Sweet potatoes

**Lower-oxalate alternatives**

- Kale, bok choy, broccoli
- Rice, oats
- Peanuts (moderate)
- Berries, apples, bananas

📌 Eat oxalate foods **with calcium-containing foods**.

**4️⃣ Sodium: This directly raises urine calcium**

**Target:** **≤2,000 mg sodium/day** (ideal: 1,500)

**Cut back on**

- Processed foods
- Restaurant meals
- Deli meats, chips, soups

**Flavor with**

- Herbs, spices, citrus
- Garlic, onion, vinegar

📌 Every 100 mmol sodium ↑ → urine calcium ↑ significantly.

**5️⃣ Protein: Moderate, especially animal protein**

Important for **both calcium and uric acid stones**.

**Target:** ~**0.8–1.0 g/kg/day**
➡️ ~65–80 g/day for most men your size

**Limit**

- Red meat
- Organ meats
- Sardines, anchovies
- Shellfish

**Prefer**

- Poultry (moderate)
- Fish (2–3x/week)
- Plant proteins (beans, lentils in moderation)

📌 Animal protein ↑ uric acid, ↓ urine pH, ↓ citrate.

**6️⃣ Purines (for uric acid stones)**

Your uric acid excretion is **elevated**, so this matters.

**Avoid / minimize**

- Organ meats
- Game meat
- Anchovies, sardines
- Excess beer and spirits

**Generally safe**

- Dairy (protective!)
- Eggs
- Most vegetables (even “high-purine” veg are fine)

**7️⃣ Raise urine citrate & pH (key for both stone types)**

**Eat more**

- Citrus fruits
- Melons
- Avocados
- Vegetables in general

**Limit**

- High-sugar diets
- Excess refined carbs

📌 If stones recur, your doctor may consider **potassium citrate**, but diet comes first.

**8️⃣ Weight & lifestyle**

- BMI 26 → small reduction helps
- Regular exercise improves urine chemistry
- Avoid crash dieting or ketogenic diets (increase stone risk)

**A simple daily template**

**Breakfast**

- Yogurt with berries
- Oatmeal
- Water + lemon

**Lunch**

- Grilled chicken or tofu
- Rice or quinoa
- Steamed vegetables

**Snack**

- Fruit + cheese

**Dinner**

- Fish or legumes
- Vegetables
- Whole grains

**When to re-check**

- Repeat **24-hour urine** after 8–12 weeks on diet
- Goal: higher volume, lower calcium & uric acid, higher citrate, pH ~6.0–6.5

If you want, I can:

- Build a **1-week meal plan**
- Calculate your **exact protein & calcium targets**
- Help you interpret future urine results

Just tell me 👍

**Case ID: C08**

**Output (Agent):**

1. Metabolic Diagnosis:

Recurrent calcium oxalate stone former with severe hyperoxaluria (urine oxalate 1.5 mmol/day ≈ 130 mg/day) and low urine volume (1.2 L/day). With Crohn’s disease, this pattern is highly suggestive of enteric hyperoxaluria (fat malabsorption → less calcium available to bind oxalate in the gut → increased oxalate absorption), often worsened by diarrhea and low fluid intake.

2. Key Dietary Modifications:

1) Bind oxalate in the gut by pairing calcium with meals (do not restrict calcium):

- Aim 1,000–1,200 mg/day calcium from food, split across meals/snacks.

- Specifically take calcium with higher-oxalate meals (this is the key enteric hyperoxaluria tactic). Examples: yogurt with lunch; milk/cheese with dinner.

- If dietary intake is insufficient, a clinician may use calcium citrate with meals for binding (medical supervision recommended).

2) Low-oxalate diet focused on the biggest contributors:

- Strongly avoid/limit: spinach, beet greens, rhubarb, almonds/almond flour, cashews, peanuts, sesame/tahini, bran, buckwheat, dark chocolate/cocoa, and frequent large portions of beets .

- Avoid “green smoothies” that concentrate oxalate (spinach-based smoothies are a common cause of extreme oxalate).

3) Increase fluids to raise urine volume despite diarrhea risk:

- Target urine volume ≥2.5 L/day if feasible; practically this often means frequent small drinks through the day.

- Use oral rehydration-type fluids when diarrhea is active (rather than plain water only), but choose lower-sugar options; avoid high-dose vitamin C drinks/supplements.

3. Sample Menu Suggestions:

Eat / choose more often (enteric hyperoxaluria-friendly):

- Breakfast: lactose-free milk or yogurt (if tolerated) + low-oxalate fruit (banana, melon) + oats.

- Lunch: chicken/turkey + rice/potatoes + low-oxalate vegetables (zucchini, cauliflower, mushrooms, peppers). Add yogurt or cheese with the meal.

- Dinner: fish/tofu + pasta/rice + green beans/carrots. Include a calcium-containing food with the meal.

- Snacks: yogurt, cheese and crackers, low-oxalate fruit; electrolyte solution during flares.

Avoid / limit (high oxalate and common triggers):

- Spinach salads, spinach smoothies, beet greens; nuts / “nut milks” as staples (especially almond milk).

- Dark chocolate/cocoa-heavy foods daily.

- High-dose vitamin C supplements (can convert to oxalate).

- If fat malabsorption is present: very high-fat meals may worsen enteric oxalate absorption (coordinate fat goals with her IBD plan).

4. Safety Warning:

With Crohn’s disease, recommendations must account for diarrhea, malabsorption, and flare-specific tolerances. Pushing fluids is important for stones but may be limited by GI symptoms; prioritize small frequent fluids and consider oral rehydration solutions during flares. Also be cautious with high-potassium “alkali” strategies or supplements if she has any unreported kidney impairment or is on medications affecting potassium. Coordination with her gastroenterologist is important, especially if she has had ileal disease/resection, which increases enteric hyperoxaluria risk.

**Output (Control):**

Thanks for sharing the details — that helps a lot. I’ll keep this practical and tailored to **recurrent calcium oxalate stones + Crohn’s disease**, which is a *very specific* (and common) combo.

Big picture from your data:

- **Low urine volume (1.2 L)** → *major* stone risk
- **High urine oxalate (1.5 mmol/day)** → consistent with **enteric hyperoxaluria** from Crohn’s
- **Normal BMI** → restriction needs to be smart, not extreme

**The 4 biggest goals for *you***

1. **Dilute urine** (most important)
2. **Reduce oxalate absorption from the gut**
3. **Bind oxalate with calcium at meals**
4. **Avoid triggers that worsen Crohn’s-related oxalate load**

**What to EAT (prioritize these)**

**1. Fluids (non-negotiable)**

Target: **≥2.5–3 L urine/day**
This usually means **3+ liters of fluid intake daily**

Best choices:

- Water 💧
- Water with lemon or lime (natural citrate)
- Diluted electrolyte drinks (low sugar)
- Herbal teas

Avoid relying on:

- Soda
- Sweetened drinks
- Excess coffee (>2 cups/day)

Tip: Spread fluids evenly, including **before bed**.

**2. Calcium — WITH meals**

This is crucial for Crohn’s-related stones.

Target: **1,000–1,200 mg/day**, **from food or supplements taken with meals**

Best sources:

- Milk, yogurt, kefir
- Cheese (moderate portions)
- Calcium-fortified plant milks (check label)

Why this helps:

Calcium **binds oxalate in the gut**, preventing absorption → *lower urine oxalate*

⚠️ Do **not** take calcium supplements *between meals*.

**3. Low-oxalate vegetables & fruits**

Safe choices:

- Lettuce
- Cucumber
- Zucchini
- Mushrooms
- Cauliflower
- Cabbage
- Bell peppers
- Apples
- Pears
- Melons
- Mango
- Grapes

You *can* eat vegetables — just choose wisely.

**4. Citrate-boosting foods**

Citrate protects against CaOx stones.

Good options:

- Lemon/lime juice
- Oranges (whole fruit > juice)
- Avocado
- Bananas

**5. Moderate, balanced protein**

Aim for **normal intake**, not high-protein.

Best choices:

- Fish
- Poultry
- Eggs
- Tofu (moderate)

**What to LIMIT or AVOID**

**1. High-oxalate foods (very important)**

Avoid or strictly limit:

- Spinach ❌
- Rhubarb
- Beets
- Swiss chard
- Almonds & almond milk
- Cashews
- Peanuts
- Sesame/tahini
- Chocolate/cocoa
- Black tea
- Wheat bran

Even “healthy” smoothies can be stone bombs if spinach/almond milk are involved.

**2. Excess fat (especially if Crohn’s not well controlled)**

Why: Fat malabsorption → more free oxalate absorbed.

Limit:

- Fried foods
- Heavy cream
- Large amounts of oils
- Fatty meats

**3. Salt (sodium)**

Target: **<2 g sodium/day**

High sodium → more urinary calcium.

Watch out for:

- Processed foods
- Restaurant meals
- Packaged snacks

**4. Vitamin C supplements**

Avoid >500 mg/day.

Vitamin C → oxalate conversion.

**Crohn’s-specific tips (important)**

- Eat **calcium at the same time as oxalate-containing foods**
- Smaller, more frequent meals can reduce fat malabsorption
- If diarrhea is active → fluid + electrolyte replacement is critical
- Probiotics may help gut oxalate metabolism (evidence is mixed, but reasonable)

**A sample day (stone-safe)**

**Breakfast**

- Yogurt with banana
- White toast with butter
- Water with lemon

**Lunch**

- Grilled chicken
- Rice
- Steamed zucchini
- Calcium-fortified drink

**Snack**

- Apple
- Cheese

**Dinner**

- Baked fish
- Potatoes
- Green beans
- Salad (lettuce + cucumber)

**Throughout the day**

- Water, water, water 💧

**When to go further**

Given your **recurrent stones + Crohn’s**, you’d likely benefit from:

- Repeat **24-hour urine after diet changes**
- Consider **potassium citrate** *if urine citrate is low*
- Coordinated care between **GI + nephrology/urology**

If you want, I can:

- Build you a **low-oxalate Crohn’s-friendly meal plan**
- Help decode a **24-hour urine report**
- Adjust advice based on **active vs quiescent Crohn’s**

Just say the word 💙

**Case ID: C09**

**Output (Agent):**

1. Metabolic Diagnosis:

Infection stone disease consistent with struvite (magnesium ammonium phosphate) stones, characterized by markedly alkaline urine (pH 7.8) and high urinary ammonium, in the setting of recurrent UTIs —most consistent with urease-producing organisms. This is not primarily a diet-driven metabolic stone; it is an infection-driven stone problem.

2. Key Dietary Modifications:

1) Hydration to reduce crystallization and help UTI prevention:

- Aim for urine output ≥2.0–2.5 L/day if medically appropriate.

- Spread fluids through the day; include a glass before bed (nighttime stasis increases risk).

2) Avoid urine-alkalinizing supplements/diets (already very alkaline):

- Do not use alkalinizing agents (e.g., bicarbonate “alkali” products) intended for uric acid stones.

- Avoid making alkaline water a routine habit.

3) UTI-focused dietary habits (adjunct, not definitive):

- Regular fluid intake; consider unsweetened cranberry products if tolerated (evidence is mixed but may reduce recurrence in some; avoid high-sugar versions).

- Avoid high-sugar beverages which can worsen UTI risk in predisposed patients.

3. Sample Menu Suggestions:

Eat / choose more often:

- Water/seltzer as main beverages; unsweetened tea/coffee in moderation.

- High-water foods: soups (moderate sodium), cucumbers, melon, oranges.

- Balanced meals with lean protein, vegetables, whole grains (general health support).

Avoid / limit:

- “Alkalinizing” supplements (baking soda, alkaline powders) and routine alkaline water.

- Sugar-sweetened drinks (soda/juice cocktails), especially if UTIs are frequent.

4. Safety Warning:

Diet alone will not prevent struvite stones. The cornerstone is complete stone clearance plus eradication/suppression of urease-producing infection and careful follow-up, because residual fragments can perpetuate infection and rapid regrowth. Any fever, flank pain, or signs of systemic infection require urgent medical evaluation due to risk of obstructive pyelonephritis/sepsis. If considering urinary acidification strategies or medications (e.g., acetohydroxamic acid in select cases), this must be managed by a urologist/infectious disease team due to side effects and monitoring needs.

**Output (Control):**

Thanks for sharing the details—that really helps. With **struvite stones**, the big picture is a little different from calcium or uric-acid stones:

**Struvite stones are driven mainly by infection**, not diet alone.

Your high urine pH (7.8) and high ammonium fit that pattern, especially with recurrent UTIs.

That said, **what you eat and drink still matters**, mainly to support urine chemistry and reduce infection risk.

**1. Hydration (non-negotiable)**

This is the single most important step.

- **Goal:** enough fluids to make **≥2–2.5 liters of urine/day**
- Practically: ~2.5–3 liters (85–100 oz) of fluid daily unless restricted by your doctor
- Water is best; spread it evenly through the day
- A glass before bed helps (struvite stones can grow fast)

**2. Diet goals for struvite stones**

The strategy is to **avoid further urine alkalinization** and **support infection prevention**.

**✅ Foods to emphasize**

These tend to **not raise urine pH** and support overall kidney health:

- **Lean proteins** (moderate portions)
  - Fish, chicken, turkey, eggs
- **Whole grains**
  - Rice, oats, barley, quinoa
- **Most vegetables**
  - Especially non-starchy: zucchini, green beans, carrots, cauliflower
- **Fruits in moderation**
  - Apples, berries, pears, grapes
- **Healthy fats**
  - Olive oil, avocado (reasonable amounts)

**⚠️ Foods to limit (not eliminate)**

These can **increase urine alkalinity**, which struvite stones like:

- Large amounts of:
  - Spinach
  - Kale
  - Beets
  - Swiss chard
- Excessive **plant-based alkalizing diets**
- Overuse of alkaline mineral waters

You don’t need to avoid vegetables—just don’t push heavily alkaline patterns.

**3. Protein: moderate, not high**

- Aim for **normal intake**, not high-protein diets
- Rough target: ~0.8–1.0 g/kg/day
- Avoid chronic high-dose protein shakes unless advised

Too much protein can worsen ammonium production.

**4. Dairy & calcium**

- **Normal dietary calcium is OK**
- Avoid excessive calcium supplements unless prescribed
- Dairy does *not* cause struvite stones—but don’t overdo it

**5. Sodium: keep it reasonable**

- High sodium increases urinary minerals
- Target: **<2,300 mg/day**
- Watch processed foods, canned soups, deli meats

**6. Cranberry & urine acidification (may help)**

Because your urine is quite alkaline:

- **Cranberry products** (juice with low sugar or capsules) may:
  - Slightly lower urine pH
  - Reduce bacterial adherence in UTIs
- Some patients are prescribed **urine-acidifying agents** (like methionine)—that’s a doctor decision, not dietary alone

**7. What diet *cannot* fix (important)**

Since these are **infection stones**:

- Diet alone will **not** prevent recurrence if UTIs persist
- Key medical steps include:
  - Complete stone clearance
  - Aggressive UTI prevention and treatment
  - Periodic urine cultures
  - Sometimes long-term suppressive antibiotics (urology-directed)

Your food choices support the plan—but **infection control is the cornerstone**.

**A simple daily pattern (example)**

- **Breakfast:** Oatmeal with berries, egg, water
- **Lunch:** Grilled chicken, rice, green beans
- **Snack:** Apple, yogurt
- **Dinner:** Fish, quinoa, roasted vegetables
- **Fluids:** Water throughout + optional cranberry supplement

If you want, I can:

- Translate this into a **1-week meal plan**
- Help you choose **cranberry supplements vs juice**
- Tailor it further if you have kidney function limits, gout, or diabetes

You’re asking exactly the right question—struvite stones are tough, but recurrence *can* be reduced with the right combo of medical + dietary steps.

**Case ID: C10**

**Output (Agent):**

1. Metabolic Diagnosis:

Calcium phosphate brushite stone former with alkaline urine (pH 6.8) and hypercalciuria (calcium 9.0 mmol/day ≈ 360 mg/day). This combination strongly favors CaP/brushite supersaturation. Key risk drivers are high urine pH (promotes CaP) plus elevated urine calcium (often amplified by sodium and animal-protein intake). Unlike uric acid stones, raising pH further is harmful here.

2. Key Dietary Modifications:

1) Lower sodium (first-line for hypercalciuria):

- Target ≤100 mmol/day sodium (≈ 2.3 g sodium/day).

- Avoid processed/restaurant foods, salty snacks, and high-sodium condiments; cook mostly at home.

2) Moderate animal protein (reduce calciuria and CaP risk):

- Aim about 0.8–1.0 g/kg/day total protein; keep meat/fish/poultry portions modest and add plant-protein meals.

3) Avoid urine-alkalinizing strategies; maintain normal calcium intake with meals:

- Do not use alkalinizing supplements (e.g., bicarbonate) or high-dose citrate therapy without monitoring, because higher pH can worsen brushite risk.

- Keep normal dietary calcium (about 1,000–1,200 mg/day from food, with meals). Do not restrict calcium.

3. Sample Menu Suggestions:

Eat / choose more often (low sodium, moderate animal protein):

- Breakfast: plain yogurt with fruit + oats; or eggs with vegetables; choose low-sodium options.

- Lunch: homemade grain/bean bowl (lentils, quinoa, vegetables) with olive oil + vinegar; or salad with unsalted chicken.

- Dinner: baked fish/tofu + rice/potatoes + vegetables; season with herbs, garlic, pepper, lemon (not salt).

- Calcium foods (normal amounts): milk, yogurt, modest cheese portions with meals.

Avoid / limit (brushite-focused):

- High-salt foods: deli meats, fast food, pizza, instant noodles, salty snacks.

- Very high-protein diets (keto/carnivore-style).

- “Alkalinizing” products: alkaline water as a routine, baking soda, and unmonitored alkali supplements intended for uric acid stones.

4. Safety Warning:

No comorbidities limiting these recommendations are listed. Because brushite stones can be aggressive and recur , dietary measures are important but often not sufficient alone; she typically needs close urology follow-up and repeat 24-hour urine testing. If potassium citrate is ever prescribed for low citrate, it should be used cautiously with CaP/brushite, with monitoring of urine pH and CaP supersaturation to avoid worsening risk.

**Output (Control):**

Thanks for sharing the details — that helps a lot. Brushite (calcium phosphate, CaP) stones behave *very* differently from the more common calcium oxalate stones, so prevention is a bit more nuanced. I’ll tailor this specifically to **CaP/brushite + high urine pH (6.8)**.

**Big picture goals for *your* stone type**

For calcium phosphate stones, we want to:

1. **Lower urine supersaturation of calcium phosphate**
2. **Avoid further raising urine pH** (this is key — CaP stones love alkaline urine)
3. **Reduce urinary calcium**
4. **Maintain high urine volume**

**1️⃣ Fluids (non-negotiable)**

**Target:** at least **2.5–3.0 L urine/day**
(Usually means drinking ~3–3.5 L/day)

**Best choices**

- Plain water
- Water with lemon *sparingly* (not heavily alkalinizing)
- **Cranberry juice (unsweetened)** → mildly acidifies urine (helpful for CaP)
- Diluted fruit juices in moderation

**Avoid / limit**

- **Alkaline water**
- Excessive mineral water (high calcium/bicarbonate)
- Large amounts of vegetable juices

**2️⃣ Calcium: don’t restrict it (but do it right)**

This surprises many people.

**Target intake:** **1,000–1,200 mg/day**, from food
🚫 **Do NOT follow a low-calcium diet**

**Best sources (with meals)**

- Milk, yogurt, kefir
- Cheese (moderate portions)
- Calcium-fortified foods

💡 Getting calcium **with meals** helps bind phosphate in the gut and *reduces urinary calcium spikes*.

**Avoid:**

- Calcium supplements unless your stone specialist specifically recommends them

**3️⃣ Sodium: very important for you**

High sodium = high urine calcium.

**Goal:** **<2,000 mg sodium/day** (ideally 1,500 mg)

**Cut back on**

- Processed foods
- Restaurant meals
- Canned soups
- Deli meats
- Chips, sauces, fast food

This alone can significantly lower calcium excretion.

**4️⃣ Protein: moderate, not high**

Animal protein:

- ↑ urine calcium
- ↑ acid load (which *can* lower pH — helpful)
- But excess still raises stone risk

**Aim for:** ~0.8–1.0 g/kg/day
(roughly 55–70 g/day for many women)

**Prefer**

- Fish
- Poultry
- Plant proteins (beans, lentils) in moderation

🚫 Avoid high-protein / keto / carnivore diets — they’re terrible for brushite stones.

**5️⃣ Fruits & vegetables: selective approach**

This is where CaP stones differ from oxalate stones.

Many fruits/vegetables → **alkalinize urine** → bad for CaP.

**Better options**

- Berries
- Apples
- Pears
- Grapes
- Cranberries
- Lettuce, cucumber, zucchini

**Limit (don’t eliminate)**

- Spinach
- Kale
- Swiss chard
- Beets
- Sweet potatoes
- Avocado

You don’t need to avoid vegetables — just don’t push heavy “alkaline diet” patterns.

**6️⃣ Citrate supplements: usually not recommended**

Potassium citrate:

- Raises urine citrate (good)
- **Raises urine pH (bad for CaP)**

Most brushite stone formers **should avoid citrate supplements** unless a specialist is carefully monitoring labs.

**7️⃣ Phosphate & additives**

- Avoid foods with **phosphate additives** (cola, processed foods)
- Natural phosphorus from food is okay

**Sample day of eating (CaP-friendly)**

**Breakfast**

- Greek yogurt with berries
- Oatmeal (low sodium)
- Water or diluted cranberry juice

**Lunch**

- Grilled chicken salad (lettuce, cucumber, olive oil)
- Whole-grain bread (low sodium)
- Apple

**Snack**

- Cheese stick + pear

**Dinner**

- Baked salmon
- Rice or quinoa
- Zucchini or green beans
- Water

**Extra (non-diet but important)**

Many patients with brushite stones benefit from:

- **Thiazide diuretics** (reduce urine calcium)
- Careful follow-up with 24-hour urine testing

If you want, I can:

- Translate this into a **personalized meal plan**
- Review a **24-hour urine panel** in detail
- Help you figure out **which foods are secretly raising your urine pH**

You’re asking the right question early — that alone makes recurrence prevention much more achievable 💙

**Case ID: C11**

**Output (Agent):**

1. Metabolic Diagnosis:

Cystinuria with cystine stone disease, with acidic urine (pH 5.5) increasing cystine insolubility and crystallization risk. Primary metabolic issues are high urinary cystine concentration (implied by positive cystine) and insufficient urine alkalinization/dilution.

2. Key Dietary Modifications:

1) Very high fluid intake distributed day and night (core therapy):

- Goal is typically urine volume ≥3.0 L/day for cystinuria (often requires ~ 3.5–4+ L/day fluid intake depending on losses).

- Include late evening and overnight fluids if feasible (cystine precipitates during overnight concentration).

2) Lower sodium strongly (reduces cystine excretion):

- Target ≤100 mmol/day sodium (≈ 2.3 g sodium/day, and many cystinuria programs aim even lower).

- Avoid processed foods, fast food, salty snacks, and high-sodium sauces.

3) Reduce methionine/animal protein load and raise urine pH with alkali foods (and usually medication):

- Moderate animal protein; emphasize plant proteins.

- Dietary alkali (fruits/vegetables) helps, but most cystinuria patients need clinician-guided alkalinization to keep urine pH in a more soluble range (often ~7.0–7.5), balancing against CaP risk if relevant.

3. Sample Menu Suggestions:

Eat / choose more often:

- Fluids: water/seltzer as default; set “scheduled drinking” (on waking, every 1–2 hours, with meals, before bed).

- Low-sodium meals:

- Breakfast: oats + fruit; yogurt; unsalted eggs.

- Lunch: homemade lentil/bean salad; low-sodium soup; fruit.

- Dinner: tofu/vegetable stir-fry with no-salt spices; rice/pasta; salad with olive oil + vinegar.

- Snacks: fruit, unsalted nuts (portion-controlled), yogurt, low-sodium crackers.

Avoid / limit:

- High-sodium foods: deli meats, pizza, fast food, instant noodles, chips, salted nuts.

- Very high animal-protein patterns (large meat portions, protein shakes as staples), which increase acid load and can make alkalinization harder.

4. Safety Warning:

Cystinuria usually requires medical therapy in addition to diet (urine alkalinization with agents like potassium citrate and sometimes cystine-binding thiol drugs). Any alkalinization plan should be supervised, especially if there is any unreported kidney impairment or medications that raise potassium. Also, the recommended high fluid targets should be individualized if he has any unreported heart/kidney conditions, but in an otherwise healthy 22-year-old they are typically appropriate for cystinuria.

**Output (Control):**

Got it — thanks for sharing the details. I’ll keep this practical and focused on **preventing cystine stone recurrence**.
*(This is general education, not a replacement for your urologist/nephrologist’s plan.)*

**Big picture (what matters most for cystinuria)**

Cystine stones form when **urinary cystine concentration is high** and **urine is too acidic**. So your diet goals are:

1. **Dilute the urine** (very high fluid intake)
2. **Lower cystine excretion** (mainly by reducing sodium and excess animal protein)
3. **Raise urine pH** (alkalinization)

**1️⃣ Fluids: the #1 intervention**

This is non-negotiable.

**Goal**

- Produce **≥3–4 liters of urine/day**
- That usually means **4–5 liters (135–170 oz) of fluids daily**

**Tips**

- Spread fluids evenly throughout the day
- Drink **before bed** and consider one nighttime drink
- Water is best; you can include:
  - Lemon water (citrate helps)
  - Diluted citrus juices (not sugar-heavy)

🚫 Avoid dehydration at all costs — exercise, heat, alcohol all increase risk.

**2️⃣ Sodium restriction (VERY important for cystinuria)**

High sodium = higher cystine excretion.

**Goal**

- **<2,000 mg sodium/day** (ideally 1,500 mg)

**Limit or avoid**

- Processed foods
- Fast food
- Chips, pretzels
- Cured meats (bacon, sausage, deli meats)
- Instant noodles, frozen meals
- Restaurant food (huge sodium source)

**Choose**

- Fresh, home-cooked meals
- Herbs, spices, lemon, vinegar instead of salt
- Read labels aggressively

This single change can significantly reduce cystine levels.

**3️⃣ Protein: moderate, not high**

Animal protein increases cystine and acidifies urine.

**Goal**

- **0.8–1.0 g protein/kg/day**
- For most 22-yo men: ~55–75 g/day

**Limit**

- Red meat
- Poultry
- Fish
- Eggs (okay occasionally, not daily in excess)

**Prefer**

- Plant-based proteins:
  - Beans (moderate amounts)
  - Lentils
  - Tofu
  - Nuts/seeds (not excessive)
- Dairy in moderation

🚫 Avoid high-protein diets (keto, carnivore, “bulking” diets).

**4️⃣ Alkalinize your urine (target pH ≥ 7.0)**

Your urine pH is **5.5**, which strongly favors cystine stone formation.

**Diet helps, but meds are often needed.**

**Foods that help raise urine pH**

- Fruits (especially citrus)
- Vegetables (leafy greens, squash, potatoes)
- Plant-forward meals

**Foods that acidify urine (limit)**

- Large amounts of meat
- Cheese-heavy meals
- Processed grains

⚠️ **Diet alone is often insufficient** — many cystinuria patients need:

- **Potassium citrate** or other alkalinizing agents
- Urine pH monitoring at home

(Ask your doctor if you aren’t already on this.)

**5️⃣ Cystine-specific notes (important nuance)**

- Cystine is **not significantly reduced by lowering dietary cystine**
- The real drivers are:
  - **Sodium**
  - **Total protein**
  - **Urine volume**
  - **Urine pH**

So don’t stress about individual amino acids — focus on the big levers above.

**6️⃣ What a “good day” of eating looks like**

**Breakfast**

- Oatmeal with fruit
- Low-fat milk or plant milk
- Water or lemon water

**Lunch**

- Rice or quinoa bowl
- Roasted vegetables
- Tofu or small portion of chicken
- Olive oil + herbs (no salty sauces)

**Snack**

- Fruit
- Yogurt
- Unsalted nuts (small portion)

**Dinner**

- Vegetables + whole grains
- Legumes or moderate animal protein
- Big glass of water

**Before bed**

- Another glass of water

**7️⃣ Things to avoid or be careful with**

🚫 High-sodium sports drinks
🚫 Protein supplements
🚫 Dehydration from alcohol or stimulants
🚫 “Clean eating” that’s secretly high in salt

**TL;DR (printable version)**

- **Fluids:** enough to pee clear, ≥3–4 L urine/day
- **Sodium:** <2 g/day
- **Protein:** moderate, avoid high-protein diets
- **Urine pH:** aim ≥7.0 (diet + meds)
- **Plant-forward meals** help

If you want, I can:

- Help you **build a weekly meal plan**
- Translate this into **college / gym / busy life eating**
- Explain **when meds like tiopronin or potassium citrate are needed**

Just tell me where you want to go next.

**Case ID: C12**

**Output (Agent):**

1. Metabolic Diagnosis:

Recurrent calcium oxalate stone former with significant hypercalciuria (urine calcium 10 mmol/day ≈ 400 mg/day) that is very likely sodium-driven given marked hypernatriuria (urine sodium 300 mmol/day, extremely high). This pattern strongly supports dietary salt as the main modifiable driver of urine calcium and CaOx supersaturation.

2. Key Dietary Modifications:

1) Aggressively reduce sodium (top priority to lower urine calcium):

- Target ≤100 mmol/day sodium (≈ 2.3 g sodium/day, ~5–6 g salt/day). From 300 mmol/day, this is a major but achievable shift with meal prep.

- Tactics: stop adding salt; avoid processed/restaurant foods; choose “no added salt/low sodium” items; use herbs, garlic, lemon, vinegar, pepper.

2) Moderate animal protein (supports lower calciuria and CaOx risk):

- Aim 0.8–1.0 g/kg/day protein; avoid high-protein diets and “double-protein” meals.

- Prefer plant proteins and low-fat dairy over frequent large meat portions.

3) Maintain normal dietary calcium with meals (do not restrict):

- Aim 1,000–1,200 mg/day calcium from food, spaced through meals (milk, yogurt, cheese in modest portions).

- This helps reduce intestinal oxalate absorption and is not the driver here—salt is.

3. Sample Menu Suggestions:

Eat / choose more often (low sodium, CaOx-friendly):

- Breakfast: plain oats cooked with milk + fruit; or eggs with vegetables (no salty breakfast meats).

- Lunch: homemade salad/grain bowl with olive oil + vinegar; unsalted chicken/tofu/beans; fruit.

- Dinner: baked fish/chicken/tofu with rice/potatoes + vegetables; season with herbs/garlic/lemon (no seasoning salt).

- Snacks: yogurt, fruit, unsalted nuts, veggies with hummus (check sodium).

Avoid / limit (biggest sodium/calciuria drivers):

- Deli meats, bacon/sausage, jerky, canned soups, instant noodles/ramen.

- Fast food, pizza, restaurant sauces.

- Salty snacks (chips, pretzels) and high-sodium condiments (soy sauce, teriyaki, bouillon, seasoning salts).

4. Safety Warning:

No comorbidities listed that would limit sodium reduction or these dietary changes. The main caution is practical: a rapid large sodium drop can change blood pressure—if he’s on any antihypertensives (not listed), BP should be monitored as diet improves. Recheck a 24-hour urine after sustained sodium reduction to confirm urine calcium falls as expected.

**Output (Control):**

I can give **evidence-based dietary guidance** tailored to **recurrent calcium oxalate (CaOx) stones**, using the information you shared. This is preventive education, not a substitute for care from your urologist or nephrologist.

**Key Issues from Your Data**

- **Recurrent CaOx stones**
- **High urine calcium** (10 mmol/day ≈ hypercalciuria)
- **Very high urine sodium** (300 mmol/day ≈ ~7 g sodium ≈ 17 g salt/day)
- BMI slightly overweight, otherwise healthy

👉 The **single most important modifiable factor for you is sodium intake**, followed by fluid intake and oxalate management.

**1. Fluids (Most Important)**

**Goal:**

- **Urine output ≥ 2.5 L/day**
- Usually requires **~3 L fluid intake/day**

**Best choices**

- Water (primary)
- Citrus beverages (lemon/lime water, unsweetened)
  - Citrate inhibits stone formation

**Limit / avoid**

- Sugar-sweetened sodas
- Excess cola (phosphoric acid)
- Large amounts of grapefruit juice (stone risk signal in some studies)

📌 Tip: Pale yellow urine throughout the day = good hydration.

**2. Sodium (CRITICAL for You)**

High sodium → **more urinary calcium → more stones**

**Target sodium intake**

- **≤ 100 mmol/day**
- ≈ **2.3 g sodium**
- ≈ **5–6 g salt/day**

Your current level (300 mmol/day) is **triple the goal**.

**Reduce / avoid**

- Processed meats (ham, bacon, sausages)
- Fast food
- Packaged snacks (chips, crackers)
- Instant noodles, canned soups
- Sauces: soy sauce, ketchup, salad dressings
- Cheese in large amounts

**Better choices**

- Fresh meats, fish, eggs
- Home-cooked meals
- Herbs, spices, lemon instead of salt
- Low-sodium labeled foods

📌 Lowering sodium alone can **significantly reduce urinary calcium** even without medications.

**3. Calcium (Do NOT Restrict)**

This is counter-intuitive but essential.

**Target**

- **Normal dietary calcium: 1,000–1,200 mg/day**
- From **food**, not supplements (unless prescribed)

**Good sources**

- Milk, yogurt, cheese (moderate portions)
- Calcium-fortified plant milks
- Sardines with bones

**Why this matters**

- Dietary calcium **binds oxalate in the gut**, preventing absorption
- Low-calcium diets **increase stone risk**

⚠️ Avoid calcium supplements unless specifically advised.

**4. Oxalate (Moderate, Not Zero)**

Avoid **large quantities** of high-oxalate foods, especially **without calcium**.

**High-oxalate foods to limit**

- Spinach
- Rhubarb
- Beets
- Swiss chard
- Nuts (especially almonds)
- Dark chocolate
- Wheat bran
- Tea (large amounts)

**Smart strategy**

- Eat oxalate foods **with calcium-containing meals**
  - Example: spinach with yogurt or cheese

**5. Animal Protein (Moderate)**

Excess animal protein:

- Increases calcium excretion
- Lowers citrate
- Raises uric acid

**Target**

- ~0.8–1.0 g/kg/day
- For you: ~65–80 g/day protein

**Limit**

- Large portions of red meat
- Organ meats
- Excess protein powders

**Prefer**

- Fish
- Poultry
- Plant proteins (beans, lentils — balanced with calcium)

**6. Increase Citrate Naturally**

Citrate inhibits stone formation.

**Helpful foods**

- Lemon, lime (fresh juice)
- Oranges
- Melons

📌 Practical tip:

- Lemon water (½ cup lemon juice diluted over the day)

**7. Weight & Lifestyle**

- Maintain healthy weight
- Regular physical activity
- Avoid dehydration (exercise, hot weather, travel)

**Sample Daily Eating Pattern**

**Breakfast**

- Yogurt with fruit
- Whole-grain toast (low sodium)

**Lunch**

- Grilled chicken or fish
- Rice or potatoes
- Vegetables (non-spinach)
- Fruit

**Dinner**

- Home-cooked meal
- Moderate cheese or dairy
- Minimal added salt

**Snacks**

- Fruit
- Low-salt options

**Summary: Your Top 5 Priorities**

1. **Drink enough to produce ≥2.5 L urine/day**
2. **Cut sodium aggressively** (this is key for you)
3. **Maintain normal dietary calcium**
4. **Moderate oxalate, pair with calcium**
5. **Moderate animal protein**

If you want, I can:

- Translate this into a **one-page food list**
- Build a **weekly meal plan**
- Explain how thiazide diuretics or potassium citrate fit in (if your doctor considers meds)

Just tell me 👍

**Case ID: C13**

**Output (Agent):**

1. Metabolic Diagnosis:

Calcium oxalate stone former with hypocitraturia (citrate 1.0 mmol/day, low) and hypomagnesuria (magnesium 1.2 mmol/day, low). Both citrate and magnesium are natural inhibitors of CaOx crystallization, so low levels increase stone risk.

2. Key Dietary Modifications:

1) Increase dietary alkali to raise citrate (food-first):

- Add fruits and vegetables at every meal, with emphasis on citrus (lemon/lime/orange) and other produce.

- Use unsweetened citrus water (e.g., lemon/lime in water) rather than sugary lemonade.

2) Increase magnesium intake from foods (and avoid low-carb patterns that cut these foods):

- Emphasize legumes (beans, lentils), whole grains (oats, brown rice), and nuts/seeds in sensible portions.

- Choose magnesium-containing vegetables (e.g., broccoli) and consider mineral waters that contain magnesium if tolerated.

3) Avoid dietary patterns that lower citrate:

- Moderate animal protein (high acid load can suppress citrate).

- Avoid frequent sugar-sweetened beverages; prioritize water/seltzer.

3. Sample Menu Suggestions:

Eat / choose more often (raise citrate + magnesium):

- Breakfast: oatmeal topped with fruit + yogurt; or whole-grain toast + avocado + fruit.

- Lunch: lentil soup or chickpea salad; add lemon dressing; side fruit.

- Dinner: salmon/tofu + brown rice/quinoa + broccoli/vegetable mix; squeeze lemon on vegetables/fish.

- Snacks: fruit, hummus with vegetables; a small portion of nuts/seeds (be mindful if she has known high oxalate—otherwise portions are fine).

- Beverages: water or seltzer with lemon/lime; unsweetened tea.

Avoid / limit:

- Very high animal-protein meals (can worsen hypocitraturia).

- Sugary drinks (soda, sweet tea, sweetened juices) in place of water.

- Highly processed diets low in fruits/vegetables/whole foods (often low in both citrate precursors and magnesium).

4. Safety Warning:

No comorbidities listed that restrict increasing fruits/vegetables or magnesium-rich foods. If she ever uses magnesium supplements, they can cause diarrhea and should be discussed with her clinician (food sources are usually safest). If future labs show CKD, potassium-containing alkalinizing therapies would require caution, but nothing here suggests that.

**Output (Control):**

I can give **evidence-based dietary guidance** tailored to your history of **calcium oxalate (CaOx) stones** and your urine results (**low citrate and low magnesium**), which are both important, modifiable risk factors.

**Your Key Risk Factors (from your data)**

- **Calcium oxalate stones**
- **Low urine citrate (1.0 mmol/day)** → citrate normally *prevents* stones
- **Low urine magnesium (1.2 mmol/day)** → magnesium binds oxalate and lowers stone risk
- BMI normal, no major comorbidities → diet can be very effective for prevention

**1. Fluids: Your #1 Prevention Tool**

**Goal:** Produce ≥ **2.5 liters of urine/day**

**What to drink**

- Water (mainstay)
- Citrus beverages (see citrate section below)
- Herbal teas (no added sugar)

**Limit / avoid**

- Sugary drinks
- Cola (phosphoric acid)
- Excess alcohol

📌 *Tip:* Spread fluids evenly throughout the day and include a glass before bed.

**2. Increase Citrate Intake (Very Important for You)**

Low citrate strongly increases CaOx stone risk.

**Best Citrate-Rich Foods**

- **Lemon & lime juice** (highest citrate)
  - ½ cup lemon juice/day diluted in water (can be split)
- Oranges, mandarins
- Grapefruit (check if interacting with any meds)
- Kiwi

⚠️ Avoid relying on sweetened lemonade—use fresh juice or no-sugar options.

**3. Increase Magnesium Intake**

Your urine magnesium is low, which favors stone formation.

**Magnesium-Rich Foods**

- Dark leafy greens (spinach in *moderation*—see oxalate note below)
- Avocado
- Legumes (lentils, chickpeas, black beans)
- Nuts & seeds (pumpkin seeds, almonds—small portions)
- Whole grains (brown rice, oats)

📌 Magnesium supplements **may help**, but discuss with your physician first (especially kidney function).

**4. Maintain NORMAL Calcium Intake (Do NOT restrict)**

**Goal:** ~**1,000–1,200 mg/day from food**

Adequate calcium **reduces oxalate absorption** in the gut.

**Good Calcium Sources**

- Milk, yogurt, kefir
- Cheese (moderate sodium)
- Calcium-fortified plant milks
- Calcium-set tofu

⚠️ Avoid calcium supplements unless advised—food sources are preferred.

**5. Moderate Oxalate Intake (Not Zero)**

You don’t need to eliminate oxalate—just be strategic.

**High-Oxalate Foods to Limit**

- Spinach
- Beets
- Rhubarb
- Almonds
- Dark chocolate
- Wheat bran

📌 *Key strategy:* Eat oxalate foods **with calcium-containing foods** (e.g., spinach + yogurt).

**6. Reduce Sodium (Critical)**

High sodium increases urinary calcium.

**Goal:** < **2,000 mg sodium/day**

Avoid:

- Processed foods
- Fast food
- Cured meats
- Salty snacks

**7. Moderate Animal Protein**

Excess animal protein lowers citrate and raises calcium.

**Better choices**

- Fish
- Poultry (moderate portions)
- Plant proteins (beans, lentils)

Limit:

- Red meat
- Organ meats

**8. Vitamin C: Avoid High Doses**

- Avoid supplements > **500 mg/day**
- High vitamin C → converts to oxalate

**Sample Day (Stone-Preventive)**

**Breakfast:**

- Yogurt with oats + berries
- Lemon water

**Lunch:**

- Lentil salad with olive oil & vegetables
- Whole-grain bread

**Snack:**

- Orange or kiwi

**Dinner:**

- Grilled fish
- Brown rice
- Steamed vegetables

**Throughout the day:**

- Water + diluted lemon juice

**When to Follow Up**

- Repeat **24-hour urine** after 8–12 weeks of diet changes
- Ask your clinician whether **potassium citrate** or **magnesium supplementation** is appropriate if diet alone is insufficient

If you’d like, I can:

- Create a **personalized weekly meal plan**
- Review **specific foods you enjoy**
- Help interpret future urine test results

Just tell me 👍

**Case ID: C14**

**Output (Agent):**

1. Metabolic Diagnosis:

No identifiable metabolic abnormality on 24-hour urine evaluation (normocalciuria, normo-oxaluria, normal citrate, sodium, uric acid, pH, and volume as reported). This is a low–intermediate recurrence risk calcium oxalate stone former with a single event and a normal metabolic profile.

2. Key Dietary Modifications:

1) Maintain high urine volume (most important universal prevention step):

- Aim for urine volume ≥2.0–2.5 L/day (usually ~2.5–3.0 L/day fluid intake; more with heat/exercise).

2) Keep sodium moderate to avoid inducing hypercalciuria over time:

- Aim ≤100 mmol/day sodium (~2.3 g/day) and minimize processed/restaurant foods.

3) Normal calcium intake with meals + balanced protein:

- Keep 1,000–1,200 mg/day dietary calcium (do not restrict).

- Avoid very high animal-protein diets; keep protein moderate and include plant proteins.

3. Sample Menu Suggestions:

Eat / choose more often:

- Water as default beverage; add lemon/lime if desired (unsweetened).

- Meals built around vegetables/whole grains/legumes with moderate portions of lean protein.

- Regular calcium foods with meals: milk, yogurt, modest cheese; calcium-fortified alternatives if needed.

Avoid / limit:

- Dehydration patterns (long periods without drinking; heavy exercise without replacing fluids).

- High-salt patterns (fast food, deli meats, salty snacks).

- Extreme diets that skew risk (very high-protein or very low-calcium approaches).

4. Safety Warning:

None based on the information provided. If he develops recurrent stones, obtains a stone composition different from CaOx, or has changes in health/medications, repeat metabolic evaluation is appropriate.

**Output (Control):**

Given your profile (30-year-old male, BMI 25, **single calcium oxalate stone**, normal labs, no comorbidities), the goal is **dietary prevention** rather than medication. The advice below is evidence-based and appropriate for first-time CaOx stone formers.

**1. Fluids (Most Important)**

**Target:** produce **≥2.0–2.5 L of urine/day**
→ usually means drinking **2.5–3.0 L of fluids daily**

**Best choices**

- Water (mainstay)
- Citrus beverages (lemon/lime water, orange juice in moderation)

**Limit / avoid**

- Sugary sodas
- Cola (phosphoric acid)
- Excess alcohol

👉 Add lemon juice (½ cup/day) if possible — citrate helps prevent stones.

**2. Calcium Intake (Do NOT restrict)**

**Target:** **1,000–1,200 mg/day from food**

✔️ **Why:** Dietary calcium binds oxalate in the gut, reducing absorption.

**Best sources**

- Milk, yogurt, cheese
- Calcium-fortified plant milks
- Tofu (calcium-set)

⚠️ Avoid calcium supplements unless prescribed — food is better.

**3. Oxalate Management (Reduce, Don’t Eliminate)**

Avoid **large amounts** of high-oxalate foods, especially **without calcium**.

**High-oxalate foods to limit**

- Spinach
- Rhubarb
- Beets
- Nuts (especially almonds)
- Chocolate, cocoa
- Wheat bran
- Sweet potatoes
- Black tea

✔️ Tip: Eat oxalate foods **with calcium-containing meals**.

**4. Sodium (Salt) Restriction**

**Target:** **<2,300 mg sodium/day** (ideally ~1,500 mg)

High sodium → ↑ urinary calcium → ↑ stone risk.

**Reduce**

- Processed foods
- Fast food
- Packaged snacks
- Restaurant meals

**5. Protein Intake (Moderate)**

**Target:** ~**0.8–1.0 g/kg/day**

Limit **excess animal protein**, which increases calcium and uric acid excretion.

**Prefer**

- Fish
- Poultry (moderate portions)
- Plant proteins (beans, lentils)

Avoid very high-protein diets (e.g., keto, carnivore).

**6. Fruits & Vegetables (Encouraged)**

They increase urinary citrate and alkalinize urine.

**Best**

- Citrus fruits
- Bananas
- Melons
- Avocado
- Most vegetables (except very high-oxalate ones)

**7. Vitamin & Supplement Cautions**

- ❌ **Vitamin C supplements >500 mg/day** (increase oxalate)
- ✔️ Normal dietary vitamin C is fine
- Avoid unnecessary supplements unless directed

**8. Practical Daily Eating Pattern**

**Breakfast**

- Yogurt + fruit
- Oatmeal with milk

**Lunch**

- Grilled chicken or fish
- Rice/quinoa
- Vegetables

**Dinner**

- Tofu or lean meat
- Vegetables
- Dairy side

**Snacks**

- Fruit
- Cheese
- Low-salt options

**When to Re-evaluate**

Consider a **24-hour urine study** if:

- Stones recur
- Strong family history
- Stones occurred at young age again

**Bottom Line**

Your prevention strategy is:
**Hydration + normal calcium + low sodium + moderate protein + smart oxalate control**

If you’d like, I can:

- Create a **1-week meal plan**
- Give you a **high- vs low-oxalate food chart**
- Tailor advice to a specific diet (vegetarian, gym-focused, etc.)

**Case ID: C15**

**Output (Agent):**

1. Metabolic Diagnosis:

- Mild hypercalciuria (or high-normal calciuria) in a CaOx stone former: urine calcium 8.0 mmol/day ≈ 320 mg/day (conversion: 1 mmol Ca = 40 mg), which is at least borderline-high for many labs and clinically relevant in CaOx risk when combined with other factors.

- High acid load from diet (hyperacidogenic diet) suggested by high urinary sulfate, most commonly reflecting high animal-protein intake (sulfur-containing amino acids). This pattern increases stone risk by raising urine calcium and lowering urine citrate (even if citrate wasn’t measured here).

2. Key Dietary Modifications:

1) Lower sodium to drive urine calcium down

- Target <2,000 mg sodium/day (≈ <87 mmol/day) and avoid “hidden salt.”

- Practical swaps: choose no-salt-added canned goods, limit sauces/condiments, avoid processed meats, instant foods, salty snacks.

2) Reduce animal protein portions and shift protein sources

- Aim for moderate protein: roughly 0.8–1.0 g/kg/day (for many men ~70–100 g/day depending on lean body weight), with smaller portions per meal.

- Replace some animal protein with plant proteins (beans/lentils/tofu) and lower-purine/lean options (fish/chicken in modest portions) to reduce sulfate/acid load.

3) Maintain normal dietary calcium with meals (do not restrict)

- Keep 1,000–1,200 mg/day calcium from food, ideally with higher-oxalate meals to bind oxalate in the gut.

- Use foods like milk, yogurt, kefir, calcium-fortified alternatives, and cheese in moderate portions. Avoid “calcium avoidance,” which can raise oxalate absorption.

3. Sample Menu Suggestions:

- Breakfast (stone-friendly)

- Plain Greek yogurt + berries + oats

- Or calcium-fortified soy milk smoothie (no added salt)

- Avoid: salty breakfast sandwiches, processed meats (bacon/sausage)

- Lunch

- Big salad + chickpeas/lentils + olive oil/lemon dressing; whole-grain bread (low-sodium)

- Or turkey/chicken 3–4 oz with rice/quinoa + vegetables (no salty seasoning blends)

- Avoid: deli meats, canned soups, fast food (very high sodium)

- Dinner

- Tofu/bean chili (no-salt-added tomatoes/beans) or salmon 3–4 oz + roasted vegetables + potatoes

- Add a calcium source if the meal contains oxalate-rich items (e.g., a glass of milk or yogurt)

- Avoid: large steak portions, heavily salted marinades, boxed/instant meals

- Snacks

- Fruit, unsalted nuts (moderate), carrots/hummus (watch sodium), yogurt

- Avoid: chips, salted nuts, jerky

- Fluids (general for CaOx)

- Spread intake through the day; choose water as default. (A precise target depends on your 24h urine volume, which wasn’t provided.)

4. Safety Warning:

- With the limited comorbidity info (obesity only), there’s no specific contraindication to standard stone-prevention diet changes.

- If you have unreported CKD, heart failure, or are on potassium-raising meds (ACE inhibitor/ARB, spironolactone), be cautious with potassium citrate or very high-potassium diets —but I can’t assess that here without creatinine/eGFR and meds.

- Because we don’t have urine sodium, citrate, oxalate, volume, pH, this plan targets the two clear signals (calcium and sulfate). Adding those values would allow much tighter personalization (e.g., whether hypocitraturia or hyperoxaluria is also present).

**Output (Control):**

I can give **evidence-based dietary guidance** tailored to your profile to help **reduce recurrence of calcium oxalate (CaOx) kidney stones**. This is general medical education—not a substitute for care from your nephrologist/urologist.

**Your key risk factors (from the info you shared)**

- **Calcium oxalate stones (CaOx)**
- **High urine calcium (hypercalciuria)** – 8.0 mmol/day
- **High urine sulfate** → usually reflects **high animal protein intake**
- **Obesity (BMI 32)** – increases stone risk via insulin resistance, low urine pH, higher calcium/oxalate excretion

Your diet should aim to:

1. **Lower urine calcium**
2. **Reduce oxalate absorption**
3. **Lower animal protein load**
4. **Increase urine volume**
5. **Maintain normal (not low) dietary calcium**

**What to EAT (Recommended)**

**1. Fluids – MOST IMPORTANT**

- **Goal:** ≥ **2.5–3.0 L urine/day**
  → usually **3–3.5 L fluid intake/day**
- Best choices:
  - Water
  - Water with lemon or lime 🍋 (natural citrate)
  - Unsweetened herbal tea
- Spread intake evenly throughout the day

**2. Normal Dietary Calcium (NOT low!)**

Low calcium diets **increase oxalate absorption**.

- **Target:** 1,000–1,200 mg/day from food
- Best sources:
  - Milk, yogurt, kefir
  - Cheese (moderate amounts)
  - Calcium-fortified plant milks
- **Take calcium with meals** (especially meals containing oxalate)

❌ Avoid calcium supplements unless specifically prescribed

**3. Low-Oxalate Vegetables & Fruits**

Choose these freely:

- Broccoli, cauliflower, cabbage
- Zucchini, cucumber
- Mushrooms
- Peas
- Apples, pears
- Bananas
- Melons
- Grapes

**4. Citrate-Rich Foods (stone-protective)**

Citrate binds calcium and prevents crystal formation.

- Lemons, limes
- Oranges
- Kiwi
- Low-sugar citrus juices (not grapefruit if on meds)

**5. Whole Grains & Fiber**

- Oats
- Brown rice
- Barley
- Whole-grain bread (moderate portions)

**What to LIMIT or AVOID**

**1. Animal Protein (VERY IMPORTANT for you)**

Your **high sulfate** strongly suggests excess animal protein.

- **Limit to:** ~0.8–1.0 g protein/kg/day
  (~65–80 g/day for most men)
- Reduce:
  - Red meat
  - Poultry
  - Fish/seafood
  - Eggs
- Favor:
  - Plant proteins (lentils, beans – moderate portions)
  - Tofu, tempeh

Why:

- Increases urine calcium
- Lowers citrate
- Raises acid load → stone formation

**2. High-Oxalate Foods**

Avoid or strictly limit:

- Spinach ❌
- Rhubarb
- Beets
- Swiss chard
- Almonds, cashews
- Peanuts
- Dark chocolate
- Black tea
- Soy protein isolate

If eaten → **always with calcium-containing food**

**3. Salt (Sodium)**

Salt drives calcium into the urine.

- **Target:** < **2 g sodium/day** (~5 g salt)
- Avoid:
  - Processed foods
  - Canned soups
  - Deli meats
  - Chips, fast food
- Cook at home when possible

**4. Sugar & Fructose**

- Avoid sugary drinks
- Limit sweets and desserts
- High fructose → ↑ calcium & oxalate excretion

**Weight & Lifestyle**

- Gradual **weight loss** improves urine chemistry
- Avoid crash dieting or high-protein diets (keto, carnivore)
- Regular physical activity helps insulin sensitivity

**Example One-Day Kidney-Stone-Friendly Menu**

**Breakfast**

- Oatmeal with milk
- Banana
- Water with lemon

**Lunch**

- Grilled vegetables (broccoli, zucchini)
- Quinoa or brown rice
- Small portion of tofu
- Yogurt

**Snack**

- Apple
- Handful of grapes

**Dinner**

- Lentil stew
- Side salad (no spinach)
- Whole-grain bread
- Water or citrus-infused water

**Ask your doctor about (medical management)**

- **24-hour urine follow-up**
- **Thiazide diuretics** (often used for high urine calcium)
- **Potassium citrate** if citrate is low

**Key Takeaway**

**Drink more, eat normal calcium, cut salt and animal protein, avoid high-oxalate foods, and aim for weight loss.**

If you’d like, I can:

- Customize a **7-day meal plan**
- Translate this into a **shopping list**
- Explain how to read food labels for oxalate/sodium
- Help interpret future urine test results

**Case ID: C16**

**Output (Agent):**

1. Metabolic Diagnosis:

- Marked hypercalciuria: urine calcium 12 mmol/day ≈ 480 mg/day, strongly lithogenic for calcium phosphate (CaP) stones.

- Hypercalcemia due to Primary Hyperparathyroidism (Primary HPT) with associated parathyroid-driven hypercalciuria (increased calcium release from bone + increased filtered load of calcium).

- In recurrent CaP stone formers, this pattern is highly consistent with systemic (endocrine) hypercalciuria, not simply dietary excess.

2. Key Dietary Modifications:

1) Strict sodium reduction to lower urine calcium

- Target <2,000 mg sodium/day (≈ <87 mmol/day). Sodium restriction is one of the most effective dietary levers to reduce calciuria.

- Avoid: processed foods, restaurant meals, canned soups, salty cheeses, sauces (soy sauce, teriyaki), cured meats.

2) Keep calcium intake normal (do not restrict food calcium), avoid calcium supplements unless prescribed

- Aim ~1,000–1,200 mg/day calcium from food, spread with meals.

- Avoid calcium supplements unless her endocrine/urology team explicitly prescribes them (in Primary HPT + stones + hypercalciuria, supplements can worsen urinary calcium in some patients). Food calcium is preferred.

3) Moderate animal protein and increase alkali from fruits/vegetables (but avoid “alkalinizing” strategies that push urine pH higher without oversight)

- Keep animal protein portions modest (helps reduce calciuria and acid load).

- Emphasize fruits/vegetables for overall diet quality and citrate support, but in CaP stone formers avoid aggressively “alkalinizing” (e.g., large bicarbonate loads) without urine pH data, because higher urine pH can increase CaP supersaturation.

3. Sample Menu Suggestions:

- Breakfast

- Oatmeal made with milk (or calcium-fortified alternative) + fruit

- Or yogurt + berries + unsalted seeds

- Avoid: salty breakfast items, processed meats, high-sodium breads

- Lunch

- Low-sodium grain bowl: quinoa/rice + vegetables + olive oil + lemon + beans or small portion chicken/fish

- Side: fruit or yogurt

- Avoid: deli meats, pickles, salty soups, fast food

- Dinner

- Baked fish or tofu + roasted vegetables + potato/rice (use herbs, citrus, vinegar, garlic instead of salt)

- Include a normal-calcium food with the meal (e.g., yogurt) rather than supplements

- Avoid: large portions of red meat + salty seasoning blends

- Beverages

- Water as default; avoid high-sodium mineral waters.

- (A precise fluid target depends on 24h urine volume, not provided.)

4. Safety Warning:

- Primary HPT with high serum calcium is a high-risk, medical/surgical issue, not just dietary. This patient should be managed with endocrinology/urology; parathyroidectomy is often definitive for stone formers who meet guideline criteria (stones themselves commonly qualify). Dietary measures help, but will not reliably overcome parathyroid-driven hypercalciuria.

- Avoid starting high-dose vitamin D or calcium supplements without the treating clinician’s direction, as both can raise calcium/urine calcium in this setting.

- If potassium citrate is considered for stone prevention, it should be done with urine pH and CaP supersaturation monitoring, because raising urine pH can worsen CaP stone risk in some patients.

**Output (Control):**

Thanks for sharing your details. Given your history of **recurrent calcium phosphate (CaP) stones** and **primary hyperparathyroidism (HPT)** with **high serum and urine calcium**, prevention focuses on **lowering urinary calcium, avoiding excess urine alkalinity, and maintaining overall stone-protective habits**.

Below is **dietary advice tailored to your situation**. This is general guidance and should complement care from your nephrologist/endocrinologist.

**1. Fluids (Most Important)**

- **Goal:** produce **≥2.5–3.0 L of urine per day**
- This usually means **drinking ~3 L/day**, more in heat or exercise
- Best choices:
  - Plain water
  - Water with a splash of lemon (small amounts are OK)
- Avoid:
  - Large amounts of **alkaline mineral water**
  - Sugary drinks and sodas

👉 Adequate hydration dilutes calcium and phosphate in urine.

**2. Calcium Intake (Do NOT restrict too much)**

This is especially important in CaP stones and HPT.

- **Target:** ~**1,000 mg/day from food**
- **Do NOT follow a low-calcium diet**
- Best sources:
  - Milk, yogurt, cheese (moderate portions)
  - Calcium-containing foods **with meals**
- **Avoid calcium supplements** unless your doctor specifically prescribes them

👉 Very low calcium intake increases stone risk and bone loss.

**3. Sodium (Salt) – Strongly Limit**

High sodium drives calcium into urine.

- **Target:** < **2,000 mg sodium/day**
- Avoid:
  - Processed foods
  - Canned soups
  - Chips, fast food
  - Pickles, sauces, soy sauce
- Choose:
  - Fresh, home-cooked meals
  - Herbs and spices instead of salt

👉 This is one of the **most effective dietary changes** for you.

**4. Protein – Moderate, Not High**

Excess animal protein increases calcium excretion.

- **Target:** ~**0.8–1.0 g/kg/day**
- Limit:
  - Red meat
  - Large portions of poultry
  - Processed meats
- Prefer:
  - Smaller portions of fish or chicken
  - Plant proteins (beans, lentils) in moderation

**5. Fruits, Vegetables & Alkali (Important for CaP Stones)**

Unlike calcium oxalate stones, **CaP stones form more easily in alkaline urine**.

- Eat fruits and vegetables **in moderation**, not excessive amounts
- Avoid:
  - Large quantities of alkaline supplements
  - **Potassium citrate** unless specifically prescribed
  - Very alkaline diets or “alkaline waters”

👉 Balance is key — not elimination.

**6. Phosphate & Colas**

- Limit **cola beverages** (contain phosphoric acid)
- Avoid excessive processed foods with phosphate additives

**7. Vitamins & Supplements**

- **Avoid high-dose vitamin D** unless supervised (important in HPT)
- Avoid:
  - Calcium supplements
  - Alkalinizing supplements
- A standard multivitamin is usually fine

**8. Weight & Lifestyle**

- Your BMI is excellent — maintain it
- Stay physically active
- Avoid dehydration (especially during illness or travel)

**Very Important Medical Note**

Because you have **primary hyperparathyroidism**, **diet alone is not enough** to fully prevent recurrence.

👉 **Definitive treatment of primary HPT (often parathyroid surgery)** significantly reduces:

- High urine calcium
- Stone recurrence
- Bone loss

You should continue close follow-up with **endocrinology and nephrology/urology**.

**Summary: What Helps You Most**

✔ High fluid intake
✔ Normal dietary calcium (food, not supplements)
✔ Low salt
✔ Moderate protein
✔ Avoid excess alkalinization
✔ Treat underlying primary HPT

If you’d like, I can also:

- Review a **sample daily meal plan**
- Explain **which foods are high vs low sodium**
- Help interpret a **24-hour urine stone profile**

**Case ID: C17**

**Output (Agent):**

1. Metabolic Diagnosis:

- Severe hypocitraturia: urine citrate 1.2 mmol/day (very low; typical targets for CaOx prevention are roughly ≥2.5–3.0 mmol/day or higher depending on lab units/ranges).

- Low urinary potassium (25 mmol/day) consistent with low dietary potassium intake and/or systemic potassium depletion.

- Given the comorbidity of hypokalemia, this pattern fits whole-body potassium deficit leading to reduced urinary citrate (hypokalemia promotes renal citrate reabsorption → low urine citrate), increasing CaOx risk.

2. Key Dietary Modifications:

1) Increase potassium-rich, citrate-producing foods daily

- Prioritize fruits and vegetables that raise potassium intake and support citrate (alkali load):

- Citrus (orange, lemon, lime), melon, banana, avocado, kiwi

- Potatoes/sweet potatoes, tomatoes, beans/lentils, leafy greens

- This targets both hypokalemia and hypocitraturia.

2) Use citrate strategically: “citrate with fluids,” not sugar

- Use lemon/lime juice in water (unsweetened) as a practical citrate source.

- Avoid high-sugar lemonade/juice cocktails; sugar can increase stone risk via metabolic effects and excess calories.

3) Moderate high acid-load eating patterns that suppress citrate

- Keep animal protein portions moderate and avoid very high-protein/low-carb patterns that can lower citrate.

- Include plant-based meals regularly (beans/tofu/vegetables) to reduce acid load.

3. Sample Menu Suggestions:

- Breakfast

- Greek yogurt + berries + banana

- Or oatmeal + kiwi + nuts (unsalted)

- Lunch

- Lentil/bean bowl with tomatoes, greens, olive oil

- Add fruit: orange or melon

- Dinner

- Salmon or chicken (moderate portion) + baked potato + salad

- Or tofu/vegetable stir-fry (use low-sodium sauces)

- Fluids

- Water with lemon/lime juice (e.g., squeeze into each bottle/glass).

- Avoid: sugar-sweetened beverages; “sports drinks” unless specifically needed for medical potassium replacement.

4. Safety Warning:

- Because he has hypokalemia, potassium repletion should be coordinated with his clinician, especially if considering potassium citrate (often appropriate for hypocitraturia, but dosing and monitoring matter).

- If he has kidney disease, is on ACE inhibitors/ARBs, spironolactone/eplerenone, or has heart rhythm issues, increasing potassium (dietary or supplements) can be risky. Without those details, increase potassium foods gradually and ensure follow-up labs.

- If hypokalemia is from ongoing losses (e.g., diarrhea, vomiting, diuretics), diet alone may not correct it—treat the cause to normalize urine citrate and reduce stone risk.

**Output (Control):**

Thanks for sharing the details — they’re very helpful. I’ll tailor this to **calcium oxalate (CaOx) stones**, **low urine citrate**, and **hypokalemia**, while keeping things practical and safe.

**Big Picture Goals for You**

1. **Increase urine citrate** (your level is low at 1.2 mmol/day)
2. **Correct low potassium**
3. **Reduce calcium oxalate crystallization**
4. **Maintain good hydration and urine chemistry**

Diet plays a *major* role in all four.

**1. Fluids (Most Important)**

**Target:** Enough fluid to produce **≥2.5 L of urine/day**
Usually means **~3 L (100 oz) of fluids daily**

**Best choices**

- Water (mainstay)
- **Citrate-containing fluids**:
  - Lemon or lime juice (fresh or bottled, unsweetened)
  - Lemon water (½ cup lemon juice/day diluted)
- Herbal teas

**Limit / avoid**

- Sugar-sweetened beverages
- Excess cola (phosphoric acid)
- Large amounts of grapefruit juice (can raise oxalate)

**2. Increase Citrate & Potassium (Key for You)**

Low citrate + hypokalemia strongly suggest benefit from **alkali-rich, potassium-rich foods**.

**Strongly recommended foods**

**Fruits (daily):**

- Oranges
- Lemons, limes
- Bananas
- Melons
- Kiwi
- Mango

**Vegetables (daily):**

- Spinach ❌ (high oxalate – avoid)
- Instead choose:
  - Broccoli
  - Cauliflower
  - Zucchini
  - Cabbage
  - Bell peppers
  - Sweet potatoes

**Legumes & plant foods:**

- Lentils
- Chickpeas
- White beans (moderate oxalate but acceptable in normal portions)

💡 These foods **raise urine citrate**, **increase potassium**, and **reduce stone risk**.

Many patients with your labs ultimately need **potassium citrate medication**, but diet is the foundation and sometimes enough.

**3. Maintain Normal Calcium Intake (Do NOT Restrict)**

**Target:** ~1,000–1,200 mg/day from food

Why: Dietary calcium **binds oxalate in the gut**, preventing absorption.

**Best calcium sources**

- Milk, yogurt, kefir
- Cheese (moderate portions)
- Calcium-fortified plant milks

🚫 Avoid calcium supplements unless directed by your doctor
✅ Get calcium **with meals**, especially meals containing oxalate

**4. Reduce Oxalate Intake (But Don’t Go Extreme)**

High oxalate foods increase CaOx stones **especially when citrate is low**.

**Avoid or strictly limit**

- Spinach
- Rhubarb
- Beets
- Almonds
- Cashews
- Peanuts
- Chocolate
- Wheat bran
- Black tea (large amounts)

**Moderate (okay occasionally, with calcium-containing meals)**

- Berries
- Soy products
- Sweet potatoes

**5. Reduce Sodium (Very Important)**

**Target:** <2,000 mg sodium/day

High sodium:

- Increases urine calcium
- Worsens potassium loss
- Raises stone risk

**Tips**

- Avoid processed foods
- Avoid salty snacks
- Read labels
- Don’t add salt at the table

**6. Moderate Animal Protein**

**Target:** ~0.8–1.0 g/kg/day

Excess animal protein:

- Lowers citrate
- Raises calcium and uric acid

**Better choices**

- Fish
- Poultry (moderate)
- Plant proteins (beans, tofu in moderation)

Avoid very high–protein diets (keto, carnivore).

**7. Weight & Lifestyle**

- BMI 28 → gradual weight loss helps stone risk
- Regular physical activity improves calcium balance
- Avoid dehydration during exercise or hot weather

**Summary: What Your Plate Should Look Like**

✅ High fluids
✅ Fruits & vegetables daily (citrate + potassium)
✅ Normal calcium **with meals**
✅ Low sodium
✅ Moderate animal protein
❌ High-oxalate foods
❌ Excess salt & sugar

**Important Medical Note**

Given **low urine citrate + hypokalemia**, many patients benefit from **potassium citrate therapy**, which:

- Raises urine citrate
- Corrects potassium
- Significantly lowers recurrence risk

This should be discussed with your physician or nephrologist.

If you want, I can:

- Build a **1-day or 7-day meal plan**
- Translate this into a **shopping list**
- Explain **potassium citrate vs diet** in more detail

**Case ID: C18**

**Output (Agent):**

1. Metabolic Diagnosis:

- Distal (Type 1) renal tubular acidosis pattern with inappropriately high urine pH (7.2) and marked hypocitraturia, creating high supersaturation for calcium phosphate (CaP) stones.

- In Type 1 RTA, chronic metabolic acidosis drives renal citrate wasting (low urine citrate) and often bone buffering/hypercalciuria, while the kidney cannot acidify urine, so urine stays alkaline —a classic CaP stone phenotype.

2. Key Dietary Modifications:

1) Alkali replacement should be citrate-based and medically supervised

- For Type 1 RTA with CaP stones, the priority is correcting systemic acidosis and raising citrate, typically with potassium citrate (or alkali therapy chosen by her clinician). Food alone is usually insufficient.

- Dietary role: support the prescription by avoiding high acid-load diets that worsen acidosis.

2) Moderate sodium strictly to reduce calciuria and CaP supersaturation

- Target <2,000 mg sodium/day.

- Avoid: processed foods, restaurant meals, salty cheeses, canned soups, sauces, cured meats.

3) Maintain normal dietary calcium with meals; avoid excessive animal protein

- Keep 1,000–1,200 mg/day dietary calcium (do not restrict), taken with meals.

- Keep animal protein moderate (large animal-protein loads increase acid burden and can worsen hypocitraturia and calciuria).

3. Sample Menu Suggestions:

- Foods to emphasize

- Low-sodium whole foods: fresh poultry/fish, eggs, unsalted nuts (moderate), grains, vegetables.

- Citrate-containing fruits in reasonable portions: lemon/lime, oranges (these can help citrate intake, though urine pH is already high so this is not a substitute for medical alkali dosing).

- Normal-calcium foods with meals: milk, yogurt, kefir, calcium-fortified alternatives.

- Example day

- Breakfast: oatmeal made with milk + berries; unsalted nuts

- Lunch: quinoa bowl with vegetables + chickpeas; fruit (orange)

- Dinner: baked fish + roasted vegetables + rice; yogurt

- Fluids: water throughout the day

- Foods to limit/avoid

- High-sodium items (major driver of urinary calcium): deli meats, pizza, fast food, salty snacks, packaged soups.

- Very high animal-protein patterns (e.g., steak-heavy, keto/carnivore style diets).

4. Safety Warning:

- In Type 1 RTA, the key therapy is medical alkali replacement and monitoring (serum bicarbonate, potassium, and ideally 24h urine including calcium/citrate and CaP supersaturation). Diet changes alone rarely normalize citrate.

- Because urine pH is already very alkaline (7.2), any citrate/alkali therapy should be titrated with follow-up urine studies to avoid further increasing CaP supersaturation. Clinicians often still use potassium citrate in RTA because the benefit of correcting systemic acidosis and raising citrate can outweigh the pH effect, but it needs monitoring.

- If she has reduced kidney function or is on potassium-retaining drugs, potassium-containing alkali requires caution and lab monitoring.

**Output (Control):**

I’m glad you’re thinking about prevention—your stone type and labs give us very specific targets. I’ll tailor this to **calcium phosphate (CaP) stones**, **alkaline urine (pH 7.2)**, **very low citrate**, and **distal (type 1) RTA**.

**Big picture (what we’re trying to fix)**

Your main stone drivers are:

1. **High urine pH** → favors calcium phosphate stones
2. **Very low citrate** → citrate normally inhibits stone formation
3. **RTA type 1** → promotes alkaline urine, low citrate, calcium loss from bone

Diet can help, but **medical therapy (often potassium citrate ± thiazide)** is usually essential in RTA. Diet works best as a *support*, not a replacement.

**1. Fluids (most important)**

**Goal:** Urine output ≥ **2.5 L/day**

**What to drink**

- Water (primary)
- Lemon or lime juice diluted in water (see citrate note below)
- Coffee or tea (moderate amounts are fine)

**What to limit**

- Sugar-sweetened beverages
- Cola (phosphoric acid)
- Alkaline mineral waters (can push urine pH higher)

**Practical tip:**
Aim for pale-yellow urine all day, including evening.

**2. Calcium intake (do NOT restrict too much)**

**Target:** ~**1,000–1,200 mg/day from food**

✔ Good sources:

- Milk, yogurt, cheese (normal portions)
- Calcium-fortified foods

✘ Avoid:

- Calcium supplements **unless prescribed** (they can raise urine calcium)

**Why:**
Low calcium intake increases bone loss and kidney calcium release—especially risky in RTA.

**3. Sodium (very important for CaP stones)**

**Goal:** ≤ **2,000 mg sodium/day** (ideally 1,500 mg)

High sodium → high urine calcium → more stones

**Limit:**

- Processed foods
- Canned soups
- Fast food
- Deli meats
- Chips, crackers

**Choose:**

- Fresh foods
- Herbs instead of salt
- “No salt added” products

**4. Protein: moderate, not high**

**Goal:** ~**0.8–1.0 g/kg/day**

Excess animal protein:

- Raises urine calcium
- Lowers citrate
- Worsens bone loss

**Prefer:**

- Plant proteins (beans, lentils)
- Fish and poultry in moderate portions

**Limit:**

- Red meat
- Large portions of animal protein

**5. Citrate: tricky but important in CaP stones**

Your citrate is **very low**, which strongly increases stone risk.

**Food sources of citrate**

- Lemon juice
- Lime juice
- Oranges (moderate)
- Melons

⚠ **Important nuance:**
Dietary citrate can help **without raising urine pH as much as alkali supplements**, but with RTA, diet alone is usually not enough.

**Practical approach:**

- ½ cup (120 mL) lemon juice per day diluted in water (split doses)
- Avoid sweetened lemonade

👉 Your nephrologist may still prescribe **potassium citrate**, but at **carefully titrated doses**, with urine pH monitoring to avoid pushing pH higher than ~6.8–7.0.

**6. Fruits & vegetables (with balance)**

✔ Encourage:

- Most vegetables
- Berries
- Apples
- Pears

⚠ Moderate (not excessive):

- Large volumes of alkaline foods (e.g., massive green smoothies)

**Why:**
They help citrate and potassium, but excessive alkali load may further raise urine pH in CaP stone formers.

**7. Oxalate: not your main issue**

Since you form **calcium phosphate**, oxalate restriction is **not a priority** unless you also have high urine oxalate.

**8. Vitamins & supplements**

Avoid:

- **Vitamin C supplements >500 mg/day** (↑ stone risk)
- Over-the-counter “alkalizing” supplements

Use only if prescribed:

- Calcium
- Vitamin D (important in RTA but must be monitored)

**Sample day (stone-friendly)**

**Breakfast**

- Greek yogurt with berries
- Oatmeal
- Water or coffee

**Lunch**

- Grilled chicken or lentils
- Brown rice or quinoa
- Steamed vegetables
- Water with lemon

**Snack**

- Apple or pear
- Unsalted nuts (small portion)

**Dinner**

- Fish or tofu
- Roasted vegetables
- Salad with olive oil (no salty dressing)

**Throughout day**

- Water consistently
- Lemon water split into 2–3 doses

**Very important medical note**

Because you have **Type 1 RTA**, diet alone is **not enough** to prevent stones and protect bone health. Standard care usually includes:

- **Alkali therapy (often potassium citrate)**
- **Possibly a thiazide diuretic** if urine calcium is high
- Regular **24-hour urine monitoring**, especially urine pH

If you want, I can:

- Translate this into a **1-page diet checklist**
- Help you **balance potassium citrate dosing with CaP risk**
- Review a **24-hour urine panel** if you have one

Just tell me 👍

**Case ID: C19**

**Output (Agent):**

1. Metabolic Diagnosis:

- Hyperoxaluria: urine oxalate 0.6 mmol/day. Using oxalate MW ≈ 88 mg/mmol, this is ~53 mg/day, which is above common CaOx risk thresholds (often >40–45 mg/day, lab-dependent).

- Likely iatrogenic hyperoxaluria from high-dose vitamin C (ascorbic acid) 1000 mg/day, which can be metabolized to oxalate and raise urinary oxalate, increasing CaOx supersaturation.

2. Key Dietary Modifications:

1) Stop high-dose vitamin C supplementation

- Discontinue 1,000 mg/day vitamin C unless there is a compelling medical indication and the prescribing clinician insists.

- If supplementation is needed, use only the lowest effective dose and avoid routine “megadoses” in CaOx stone formers.

2) Keep normal dietary calcium with meals to bind oxalate

- Target 1,000–1,200 mg/day calcium from food, and specifically pair calcium with meals that contain oxalate (this reduces intestinal oxalate absorption).

- Examples: milk/yogurt with meals, calcium-fortified milk alternatives, cheese in modest portions.

3) Reduce high-oxalate foods and avoid “oxalate stacking”

- Limit the biggest oxalate contributors (especially when eaten frequently or in large portions): spinach, rhubarb, beets, almonds, cashews, peanut products, bran, dark chocolate/cocoa, many “green” smoothies, and large amounts of black tea.

- Don’t combine multiple high-oxalate items in the same day unless calcium is co-ingested.

3. Sample Menu Suggestions:

- Eat more

- Breakfast: yogurt + berries + oats (instead of spinach smoothie); coffee or herbal tea

- Lunch: chicken/turkey or tofu wrap with lettuce/cucumber/peppers + cheese; fruit

- Dinner: salmon + rice/potatoes + broccoli; glass of milk or yogurt

- Snacks: apples, grapes, carrots; low-salt crackers with cheese

- Avoid/limit

- Spinach salads and spinach smoothies (top priority)

- Almond-flour/keto baking, big handfuls of nuts daily

- Dark chocolate/cocoa-heavy foods

- High-dose vitamin C tablets/powders; “immune megadose” drinks

4. Safety Warning:

- No special safety restriction from the info provided, but if he has CKD or a history of malabsorption/bariatric surgery/IBD, hyperoxaluria can be more severe and needs tighter management—diet plus targeted therapy.

- After stopping vitamin C and adjusting diet, a repeat 24-hour urine is important to confirm oxalate normalizes and to check for other drivers (low urine volume, low citrate, high sodium/calcium) that weren’t provided here.

**Output (Control):**

Given your history of **recurrent calcium oxalate (CaOx) stones** and **elevated urine oxalate (0.6 mmol/day)**, your diet can make a *big* difference in preventing recurrence. Below is **targeted, evidence-based advice tailored to your profile**.

**1. Most important changes for *you***

**Stop high-dose vitamin C**

- **Strong recommendation:** Discontinue **vitamin C 1000 mg/day**
- Vitamin C is metabolized to oxalate and **raises urinary oxalate**, especially at doses ≥500 mg/day
- If needed, keep total vitamin C **≤100 mg/day** from food alone

**Increase fluid intake (non-negotiable)**

- Goal urine output: **≥2.5 L/day**
- Practical target: drink **3 L/day**
- Best choices:
  - Water
  - Lemon or lime water (natural citrate)
- Limit:
  - Sugar-sweetened beverages
  - Cola drinks (phosphate load)

**2. Calcium: eat MORE (but correctly)**

Many patients mistakenly restrict calcium—this **increases oxalate absorption**.

**Recommended intake**

- **1000–1200 mg/day from food**
- Spread across meals

**Good calcium sources**

- Milk, yogurt, kefir
- Cheese (moderate portions)
- Calcium-fortified plant milks

⚠️ Avoid calcium supplements **unless prescribed**, and if used, **take with meals**.

**3. Reduce oxalate load (very relevant for you)**

**High-oxalate foods to LIMIT or AVOID**

- Spinach
- Beets
- Rhubarb
- Swiss chard
- Almonds, peanuts
- Dark chocolate, cocoa
- Wheat bran
- Black tea (large amounts)

**Lower-oxalate alternatives**

- Kale, bok choy, lettuce
- Broccoli, cauliflower
- Rice, oats
- Apples, bananas, berries
- Dairy-based snacks instead of nuts

**Key rule:** If you eat oxalate-containing foods, **eat them with calcium**.

**4. Sodium: keep it low**

High sodium → increased urinary calcium.

- Target: **<2,000 mg sodium/day**
- Avoid:
  - Processed foods
  - Fast food
  - Cured meats
- Use herbs/spices instead of salt

**5. Protein: moderate, not high**

- Excess animal protein increases calcium and lowers citrate
- Aim for **0.8–1.0 g/kg/day**

**Prefer:**

- Fish
- Poultry
- Plant proteins (lentils, tofu — moderate oxalate)

Limit:

- Red meat
- Processed meats

**6. Increase urinary citrate (natural stone inhibitor)**

**Best dietary sources**

- Lemon juice
- Lime juice
- Oranges (moderate)
- Vegetables

Tip:
**½ cup lemon juice/day** (diluted) ≈ therapeutic citrate dose

**7. Weight & lifestyle**

- BMI 27 → mild overweight
- Gradual weight loss helps reduce stone risk
- Avoid dehydration during exercise or heat exposure

**8. Summary: your personal stone-prevention checklist**

✅ Stop vitamin C supplements
✅ Drink ≥3 L fluids/day
✅ Eat normal calcium with meals
✅ Limit high-oxalate foods
✅ Reduce salt
✅ Moderate animal protein
✅ Increase citrate (lemon/lime)

If you’d like, I can:

- Create a **1-day or 7-day meal plan**
- Review your **24-hour urine results in detail**
- Help decide if **potassium citrate** therapy may be useful

Just tell me 👍

**Case ID: C20**

**Output (Agent):**

1. Metabolic Diagnosis:

- Low urine volume: 1.5 L/day, increasing CaOx supersaturation (concentration-driven stone risk).

- Borderline hypercalciuria / high-normal calciuria: urine calcium 7.0 mmol/day ≈ 280 mg/day, which can be clinically relevant for CaOx risk, especially when urine volume is low.

2. Key Dietary Modifications:

1) Increase fluid intake to raise urine volume

- Goal: ≥2.5 L urine/day (often requires ~ 3.0 L/day fluid, more with sweating).

- Tactics: 500 mL on waking, 500 mL mid-morning, 500 mL mid-afternoon, 500 mL evening; add an extra glass with each meal.

2) Lower sodium to reduce urine calcium

- Target <2,000 mg sodium/day.

- Biggest wins: cut restaurant/fast food, processed meats, salty snacks, canned soups; choose “no-salt-added” products.

3) Maintain normal dietary calcium with meals; moderate animal protein

- Keep 1,000–1,200 mg/day dietary calcium (do not restrict), spread across meals.

- Keep animal protein portions moderate (helps limit calciuria and supports citrate).

3. Sample Menu Suggestions:

- Fluids (daily examples)

- Water as default; unsweetened sparkling water is fine.

- Add lemon/lime to water if desired.

- Avoid: sugar-sweetened drinks; go easy on high-sodium tomato/vegetable juices.

- Meals

- Breakfast: oatmeal + milk (or yogurt) + fruit; water

- Lunch: low-sodium turkey/chicken or hummus sandwich; salad (non-spinach-based) + yogurt; water

- Dinner: fish/chicken/tofu + rice/potatoes + vegetables; water

- Snacks: fruit, yogurt, unsalted nuts (moderate)

- Foods to avoid/limit (for sodium/calcium control)

- Deli meats, pizza, fast food, packaged soups, salty snack foods, heavy sauces/seasoning blends.

4. Safety Warning:

- With no comorbidities provided, increasing fluids to achieve ≥2.5 L urine/day is generally safe. If there is any history of heart failure, advanced CKD, or hyponatremia, fluid targets would need medical tailoring.

- If urine volume remains low despite effort, consider practical barriers (work schedule, nocturia) and use timed drinking rather than “chugging” late at night.

**Output (Control):**

I can give you **evidence-based dietary guidance** tailored to your profile and stone type. This is educational advice and should complement (not replace) care from your clinician or nephrologist.

**Your Risk Profile (What Matters Most)**

- **Stone type:** Calcium oxalate (CaOx)
- **Urine volume:** 1.5 L/day → **too low** (major risk factor)
- **Urine calcium:** 7.0 mmol/day → **elevated (hypercalciuria)**
- **BMI 29:** Overweight (associated with higher stone risk)
- **No major comorbidities:** Good — lifestyle changes can be very effective

**What You Should Eat (and Drink)**

**1. Fluids – Most Important Intervention**

**Goal:**
➡️ **Urine output ≥ 2.5 L/day**
➡️ This usually requires **3.0–3.5 L fluid intake/day**

**Best choices**

- Water (primary)
- Lemon or lime water (natural citrate)
- Diluted citrus juices (no added sugar)

**Limit / avoid**

- Sugary drinks
- Cola (phosphoric acid)
- Excess alcohol

📌 Tip: Spread fluids evenly through the day and include a glass before bed.

**2. Calcium – Do NOT Restrict**

This is counterintuitive but critical.

**Target intake:**
➡️ **1,000–1,200 mg/day from food**

**Best sources (with meals)**

- Milk, yogurt, cheese
- Calcium-fortified alternatives
- Sardines with bones

🚫 **Avoid calcium supplements** unless prescribed
✔️ Dietary calcium binds oxalate in the gut → **reduces stone risk**

**3. Reduce Sodium (Very Important for You)**

High sodium increases urinary calcium.

**Goal:**
➡️ **< 2,000 mg sodium/day** (ideally 1,500 mg)

**Limit**

- Processed foods
- Fast food
- Canned soups
- Deli meats
- Chips, crackers

**Choose**

- Fresh foods
- Herbs/spices instead of salt

📌 Lower sodium = lower urine calcium

**4. Moderate Animal Protein**

Excess animal protein increases calcium and lowers citrate.

**Limit to**

- ~0.8–1.0 g/kg/day protein
- Moderate portions of:
  - Red meat
  - Poultry
  - Fish

**Prefer**

- Plant proteins (beans, lentils — in moderation)
- Balance meals with vegetables

**5. Oxalate – Reduce High Sources (Not Eliminate All)**

Especially important for CaOx stones.

**High-oxalate foods to limit**

- Spinach
- Beets
- Rhubarb
- Almonds
- Cashews
- Peanuts
- Dark chocolate
- Black tea

**Safer alternatives**

- Kale
- Broccoli
- Cauliflower
- Apples
- Bananas
- White rice

📌 Eat oxalate-containing foods **with calcium-rich meals**

**6. Increase Citrate (Natural Stone Inhibitor)**

Citrate prevents calcium crystal formation.

**Good sources**

- Lemon juice (½ cup/day diluted)
- Lime
- Oranges
- Melons

📌 Lemon water daily is strongly recommended

**7. Weight & Metabolic Health**

- Aim for **gradual weight loss**
- Avoid crash dieting or high-protein diets (e.g., keto)

**Example Day of Eating (Stone-Friendly)**

**Breakfast**

- Oatmeal with milk
- Banana
- Water with lemon

**Lunch**

- Grilled chicken (moderate portion)
- Brown rice
- Steamed broccoli
- Yogurt

**Snack**

- Apple
- Water

**Dinner**

- Baked fish
- Roasted vegetables
- Small serving of cheese

**Fluids throughout day**

- Water spaced evenly
- Lemon water once or twice

**Key Takeaways (Your Priority List)**

1. **Increase fluids** → biggest impact
2. **Lower sodium**
3. **Normal dietary calcium**
4. **Moderate animal protein**
5. **Limit high-oxalate foods**
6. **Increase citrate (lemon/lime)**

If you’d like, I can:

- Create a **7-day meal plan**
- Translate this into a **shopping list**
- Explain how medications (like thiazides or potassium citrate) fit into prevention
- Review another urine test (e.g., oxalate, citrate, sodium)

Just tell me.

**Case ID: C21**

**Output (Agent):**

1. Metabolic Diagnosis:

- Unduly acidic urine (low urine pH 5.0) consistent with uric acid stone formation (uric acid precipitates in acidic urine).

- Hyperuricosuria (“UA high”), which increases uric acid supersaturation and stone risk.

- High-risk constraint: concomitant CHF limits aggressive fluid-loading strategies.

2. Key Dietary Modifications:

1) Reduce purine load and animal protein

- Cut back on high-purine meats and seafood: organ meats, anchovies/sardines, mussels/scallops, game meats, large portions of red meat.

- Choose lower-purine proteins in modest portions: eggs, low-fat dairy, tofu, beans/lentils (generally acceptable), and smaller portions of poultry/fish.

2) Avoid fructose and sugar-sweetened beverages

- Eliminate/avoid regular soda, sweetened iced tea, energy drinks, and large amounts of juice. Fructose increases uric acid production and can worsen hyperuricosuria.

- Prefer water (within CHF plan), coffee/tea without sugar, and low/zero-calorie beverages.

3) Urine alkalinization via diet plus medical therapy discussion

- The main preventive lever for uric acid stones is raising urine pH (often target ~6.0–6.5, clinician-directed). Diet can help, but many patients require alkali therapy (often potassium citrate or sodium bicarbonate).

- Dietary support: more vegetables and fruits (alkali-producing foods) and less animal protein.

- Because CHF patients often need to limit sodium, avoid self-starting sodium bicarbonate without cardiology/urology input.

3. Sample Menu Suggestions:

- Eat more

- Breakfast: oatmeal with low-fat milk/yogurt + berries; coffee/tea (unsweetened)

- Lunch: lentil/bean soup made low-sodium + salad + fruit

- Dinner: tofu/vegetable stir-fry or small portion chicken + vegetables + rice/potato

- Snacks: fruit, yogurt, unsalted nuts (moderate)

- Avoid/limit

- Organ meats; large red-meat portions; high-purine seafood (anchovies/sardines/shellfish)

- Alcohol, especially beer/spirits (raises uric acid; beer is particularly purine-rich)

- Sugar-sweetened beverages and high-fructose products

4. Safety Warning:

- With CHF, do not follow generic “drink as much as possible” stone advice. Fluid targets should match the cardiology-recommended fluid and sodium limits (often fluid restriction is necessary). Work with cardiology/urology to find the safest urine-volume goal.

- If urine alkalinization medication is used:

- Sodium bicarbonate can worsen fluid retention and blood pressure in CHF due to sodium load—use only if specifically directed.

- Potassium citrate may be preferable from a sodium standpoint, but requires monitoring, especially if there is any CKD or use of ACEi/ARB/MRA (spironolactone/eplerenone), due to hyperkalemia risk.

**Output (Control):**

Given your history of **uric acid kidney stones**, **acidic urine (pH 5.0)**, **high uric acid**, and **heart failure (CHF)**, the goal is to **reduce uric acid production** and **raise urine pH**—*while staying safe for your heart*.

Below is **diet-focused, evidence-based advice**, tailored to your situation.

**Key Principles for Uric Acid Stone Prevention**

1. **Alkalinize the urine** (target urine pH ≈ **6.0–6.5**)
2. **Lower uric acid production**
3. **Maintain adequate—but CHF-appropriate—hydration**
4. **Avoid sodium excess** (important for both stones and heart failure)

**What You SHOULD Eat More Of**

**🥬 Fruits & Vegetables (Very Important)**

These help **raise urine pH naturally**.

- Citrus fruits: **lemons, limes, oranges**
  (lemon juice in water is especially helpful)
- Melons, bananas, apples, pears
- Leafy greens, broccoli, cucumbers, zucchini, potatoes

✔ These foods are **alkalinizing** and **low in purines**

**🍚 Whole Grains & Plant Proteins**

- Brown rice, oats, barley
- Lentils, chickpeas, beans (moderate portions)
- Tofu, soy products

✔ Prefer **plant protein over animal protein**

**🥛 Low-Fat Dairy**

- Low-fat milk, yogurt, kefir

✔ Associated with **lower stone risk**

**💧 Fluids (With CHF Caution)**

- Aim for **steady hydration throughout the day**
- Water with lemon is ideal
- Spread fluids evenly (avoid large boluses)

⚠️ **Important:**
Because of CHF, **do NOT exceed your cardiologist’s fluid limit**. Even modest dehydration increases uric acid stone risk.

**What You Should LIMIT or AVOID**

**🥩 High-Purine Foods (Major Trigger for Uric Acid Stones)**

Avoid or keep very limited:

- Red meat (beef, pork, lamb)
- Organ meats (liver, kidney)
- Game meats
- Anchovies, sardines, herring, mackerel
- Shellfish

✔ Choose **small portions** of poultry or fish instead (not daily)

**🍺 Alcohol (Especially Beer)**

- Beer is **very high in purines**
- Spirits and wine can also raise uric acid

👉 Best to **avoid**, or keep to **rare, small amounts**

**🧂 Salt (Critical for CHF + Stones)**

- Avoid processed foods, canned soups, fast food
- Do not add salt at the table

✔ High sodium increases **calcium & uric acid excretion** and worsens CHF

**🥤 Sugary Drinks & Fructose**

Avoid:

- Soda
- Sweetened juices
- High-fructose corn syrup

✔ Fructose **raises uric acid production**

**Protein Guidance (Very Important)**

- Aim for **moderate total protein**
- Prefer:
  - Plant protein
  - Low-fat dairy
- Limit animal protein to **small portions (≤1 serving/day)**

**Weight & Metabolism**

- BMI 28 → gradual weight loss helps lower uric acid
- Avoid crash diets or high-protein diets (keto, carnivore ❌)

**Summary: Best Diet Pattern for You**

**Mediterranean-style, plant-forward, low-salt diet**

- High fruits & vegetables
- Moderate whole grains
- Limited animal protein
- Low sugar & low sodium

This approach helps:
✔ Raise urine pH
✔ Lower uric acid
✔ Support heart failure management

**One Important Follow-Up Question**

Do you currently have a **daily fluid restriction** from your cardiologist (for example, 1.5–2.0 liters/day)?
That detail helps fine-tune hydration advice safely.

If you’d like, I can also:

- Help design a **1-day or 7-day meal plan**
- Suggest **CHF-safe lemon water strategies**
- Explain **medications (like urine alkalinization) at a high level** without prescribing

Just tell me.

**Case ID: C22**

**Output (Agent):**

1. Metabolic Diagnosis:

- Hyperoxaluria: urine oxalate 0.7 mmol/day. Using MW ≈ 88 mg/mmol, this is ~62 mg/day, a clear risk factor for CaOx stones.

- High-risk comorbidity: CKD stage 4 (advanced CKD). This changes prevention priorities because:

- Potassium handling may be impaired (risk with high-potassium diets and potassium citrate).

- Fluid goals may need individualization if there is edema/heart disease, though CKD alone doesn’t always mandate restriction.

2. Key Dietary Modifications:

1) Lower oxalate intake by targeting the highest-oxalate foods

- Avoid/strictly limit: spinach, rhubarb, beet greens, almonds/cashews, peanut products, bran, dark chocolate/cocoa, and large “green smoothie” intakes.

- Choose lower-oxalate greens: romaine, iceberg, arugula, kale (moderate), cabbage (oxalate varies; spinach is the big offender).

2) Maintain normal dietary calcium with meals (food-based) to bind oxalate

- For CaOx, do not restrict dietary calcium. Aim roughly 1,000–1,200 mg/day from food, spread across meals and specifically with oxalate-containing foods.

- Use low-sodium dairy (milk, yogurt) or clinician-approved calcium sources with meals.

- If he uses calcium supplements, they should be discussed with nephrology (CKD-mineral bone disease considerations), but binding oxalate with meal-time calcium is often helpful when supervised.

3) Reduce intestinal oxalate absorption triggers

- Avoid high-dose vitamin C supplements (can increase oxalate).

- Keep dietary fat reasonable (very high fat can increase oxalate absorption in malabsorption states; if he has diarrhea, bariatric surgery, or steatorrhea, this becomes even more important).

- Prioritize whole foods over processed items (often high sodium; sodium can increase calciuria and worsen stones, though urine calcium wasn’t provided).

3. Sample Menu Suggestions:

- Eat more (generally lower-oxalate choices)

- Breakfast: oatmeal made with milk; berries; eggs

- Lunch: chicken/turkey or tofu with rice + cucumber/lettuce salad; yogurt

- Dinner: fish + potatoes + cauliflower/green beans; milk or yogurt with the meal

- Snacks: apples, grapes, pears; crackers + cheese (watch sodium); carrots

- Avoid/limit (high oxalate)

- Spinach salads/smoothies, beet greens, rhubarb

- Almond milk/almond flour heavy use; cashews; peanut butter daily

- Dark chocolate/cocoa, bran cereals

- High-dose vitamin C tablets/powders

4. Safety Warning:

- CKD stage 4: do not aggressively increase potassium-rich foods (e.g., large amounts of citrus/banana/potato/tomato) or use potassium citrate without nephrology oversight due to hyperkalemia risk.

- Fluid targets should be individualized to kidney function and volume status; avoid blanket “high fluid” prescriptions if there is any tendency to fluid overload.

- Given advanced CKD and significant hyperoxaluria, this patient warrants evaluation for secondary causes (malabsorption, bariatric surgery, chronic diarrhea, excessive vitamin C, very high oxalate diet). Dietary oxalate restriction plus supervised calcium-with-meals is usually first-line, but coordination with nephrology is essential.

**Output (Control):**

I’m glad you’re thinking about prevention—**calcium oxalate (CaOx) stone prevention in someone with Stage 4 CKD needs a careful, kidney-safe approach**. Below is **general dietary guidance**, but because of your advanced CKD, **individualization with your nephrologist/renal dietitian is essential**.

**Big Picture Goals (for CaOx stones + CKD 4)**

1. **Lower urinary oxalate**
2. **Maintain adequate (not low) calcium intake**
3. **Avoid excess protein, sodium, potassium, and phosphorus**
4. **Prevent dehydration without fluid overload**

**What to Eat (and Why)**

**1. Calcium: Adequate, Not Restricted**

Low calcium intake *increases* CaOx stone risk.

**Goal:** ~800–1,000 mg/day **from food**, spread across meals
(Exact target may vary in CKD)

**Good options (lower phosphorus preferred):**

- Small portions of milk or yogurt (check phosphorus content)
- Calcium-fortified rice milk (not almond milk—high oxalate)
- Calcium supplements **only if prescribed**, and **with meals**

📌 Calcium binds oxalate in the gut → less oxalate absorbed

**2. Limit High-Oxalate Foods**

Your urine oxalate (0.7 mmol/day) is elevated, so this matters.

**Avoid or strictly limit:**

- Spinach
- Beets
- Rhubarb
- Swiss chard
- Nuts & nut butters
- Almond milk
- Dark chocolate
- Wheat bran
- Soy products

**Lower-oxalate alternatives:**

- Lettuce
- Cabbage
- Cauliflower
- Broccoli
- Rice
- Pasta
- Apples, grapes, pears, berries (moderate portions)

**3. Protein: Moderate Intake**

Excess animal protein increases calcium excretion and stone risk and worsens CKD.

**General target:**
~0.6–0.8 g/kg/day (often used in CKD4, confirm with your nephrologist)

**Prefer:**

- Small portions of chicken or fish
- Egg whites
- Limited plant proteins (but avoid soy due to oxalate)

**Limit:**

- Red meat
- Processed meats
- Large portions of animal protein

**4. Sodium: Keep It Low**

High sodium increases urinary calcium.

**Target:** ≤2,000 mg/day (often lower in CKD)

**Avoid:**

- Processed foods
- Canned soups
- Fast food
- Salty snacks

**5. Potassium: Be Careful**

Many stone-friendly foods are **dangerous in CKD4**.

**Use caution / limit:**

- Bananas
- Oranges
- Avocados
- Potatoes
- Tomatoes
- Dried fruits

Choose **lower-potassium fruits/vegetables** in controlled portions.

**6. Fluids: Balance Is Critical**

High urine volume prevents stones, but CKD may limit fluids.

**Typical stone goal:** urine output ≥2 L/day
**CKD reality:** follow your nephrologist’s fluid limit

**Best choices:**

- Water
- Water with lemon juice (small amounts)

⚠️ Avoid large volumes of:

- Tea (oxalate)
- Cola (phosphorus)
- Vitamin C drinks

**7. Avoid High-Dose Vitamin C**

Vitamin C converts to oxalate.

**Limit:** ≤100 mg/day unless directed otherwise
Avoid supplements.

**Summary Table**

| **Do** | **Avoid** |
| --- | --- |
| Eat calcium with meals | Spinach, nuts, almond milk |
| Moderate protein | High meat intake |
| Low sodium | Processed foods |
| Kidney-safe fruits/veg | High-potassium foods |
| Adequate fluids (as allowed) | High-dose vitamin C |

**Very Important**

Because you have **Stage 4 CKD**, stone prevention must be coordinated with:

- **Nephrology**
- **Renal dietitian**
- Review of **potassium, phosphorus, calcium, and PTH levels**

If you’d like, I can:

- Help design a **1-day kidney-safe, low-oxalate meal plan**
- Translate this into a **“foods to choose / foods to limit” checklist**
- Help you prepare questions for your nephrologist or dietitian

Just tell me 👍

**Case ID: C23**

**Output (Agent):**

1. Metabolic Diagnosis:

- Hypercalciuria: urine calcium 9.0 mmol/day (≈ 360 mg/day) is elevated for a CaOx stone former (commonly >200–250 mg/day in women, depending on lab cutoffs).

- Likely sodium-driven (renal leak/idiopathic) hypercalciuria: urine sodium 150 mmol/day (≈ 3,450 mg sodium/day ≈ 8.8 g salt/day) is high and is a common dietary driver of elevated urine calcium.

- (Other key CaOx risks—low urine volume, hypocitraturia, hyperoxaluria—cannot be assessed because they weren’t provided.)

2. Key Dietary Modifications: (Top 3 priorities)

1) Lower sodium intake (primary lever for urine calcium):

- Aim for ≤2,000 mg sodium/day (≈ ≤87 mmol/day, ≈ 5 g salt/day) if obstetrics/nephrology have no contraindication.

- Practical targets: avoid processed foods; choose “no added salt”; limit restaurant/fast food; use herbs/lemon for flavor.

2) Maintain normal calcium intake from food (do NOT restrict calcium in pregnancy or CaOx):

- Target ~1,000 mg/day calcium from foods (pregnancy requirement), spread across meals. This supports fetal needs and helps reduce intestinal oxalate absorption.

- Avoid intentional low-calcium diets, which can worsen CaOx risk.

3) Moderate animal protein portions (not high-protein diets):

- Excess animal protein raises calciuria and lowers citrate; in pregnancy you still need adequate protein, but avoid “high-protein” regimens/shakes.

- Use pregnancy-appropriate portions (coordinate with OB); include plant proteins as tolerated.

3. Sample Menu Suggestions: (examples tailored to sodium-driven hypercalciuria in pregnancy)

Choose more often (lower sodium, adequate calcium):

- Breakfast: plain yogurt or milk + oats + berries; unsalted nuts in small portions

- Lunch: homemade chicken/turkey salad on low-sodium bread; side of fruit; milk or calcium-fortified alternative (if used)

- Dinner: baked salmon/chicken, rice/potatoes, vegetables (no added salt), olive oil + herbs; small portion of cheese if sodium allows

- Snacks: fruit, unsalted popcorn, carrots/cucumber with low-sodium dip; milk/yogurt

Limit/avoid (high sodium = worsens urine calcium):

- Processed meats (deli meat, bacon, sausage), instant noodles, canned soups, packaged sauces, chips/salty snacks

- Restaurant/fast food (often >1,500–2,500 mg sodium per meal)

- High-salt cheeses and salty condiments (soy sauce, pickles, bouillon)

Calcium sources that usually fit a low-sodium approach:

- Milk, plain yogurt, low-sodium / “reduced sodium” dairy where possible

- If using cheese: choose lower-sodium options and small portions (many cheeses are sodium-dense)

4. Safety Warning:

- Pregnancy (24 weeks): do not pursue aggressive dietary restriction or weight loss. Maintain adequate calories, calcium, and protein per OB guidance.

- Avoid pharmacologic stone-prevention meds commonly used outside pregnancy (e.g., thiazides, potassium citrate) unless specifically directed by obstetrics/urology—management is usually dietary/hydration-based during pregnancy.

- Hydration is important for stone prevention, but fluid goals should be individualized (especially if nausea, reflux, or any pregnancy complications).

**Output (Control):**

I’m glad you’re thinking about prevention—this is especially important **during pregnancy**, when we want to protect both you and the baby while reducing stone risk.

You have **calcium oxalate (CaOx) stones**, **high urine calcium**, and **high sodium intake**, which are key drivers of recurrence. Below is **pregnancy-safe, evidence-based dietary guidance** tailored to your situation.

**1. Fluids (MOST important)**

**Goal:** produce ≥2–2.5 L urine/day
**Practical target:** drink ~2.5–3 L fluid/day unless your OB has restricted fluids

**Best choices**

- Water
- Lemon water or citrus (natural citrate helps prevent stones)
- Diluted fruit juice (small amounts)

**Limit / avoid**

- Sugary drinks
- Cola (phosphoric acid)
- Excess tea (oxalate)

💡 Sip consistently throughout the day—don’t “catch up” at night.

**2. Sodium: lower it (very important for you)**

Your urine sodium of 150 mmol/day is high and **directly increases urine calcium**.

**Goal:**

- ≤2,000 mg sodium/day (≈ 85 mmol)

**Avoid / limit**

- Processed foods, fast food
- Canned soups
- Chips, crackers
- Frozen meals
- Sauces (soy sauce, teriyaki, ketchup)

**Choose**

- Fresh meats, fish, eggs
- Fresh or frozen vegetables
- Home-cooked meals
- Use herbs, lemon, garlic instead of salt

📌 Lowering sodium will **reduce urine calcium without harming pregnancy**.

**3. Calcium: do NOT restrict (this is critical in pregnancy)**

Even with high urine calcium, **you should NOT cut dietary calcium**.

**Target intake (pregnancy):**

- ~1,000–1,200 mg/day from food

**Best sources**

- Milk, yogurt, cheese
- Calcium-fortified plant milks
- Calcium-set tofu

**Key rule**

- **Eat calcium WITH meals**, especially meals containing oxalate
  → calcium binds oxalate in the gut and prevents stone formation

🚫 Avoid calcium supplements unless your OB specifically recommends them.

**4. Oxalate: moderate, not eliminate**

Avoid extreme restriction—balance is the goal.

**Limit high-oxalate foods**

- Spinach
- Beets
- Rhubarb
- Almonds
- Cashews
- Peanuts
- Chocolate
- Wheat bran

**Okay in moderation**

- Sweet potatoes
- Berries
- Beans
- Tea (small amounts)

💡 Pair oxalate foods with calcium (e.g., yogurt with berries).

**5. Protein: moderate animal protein**

Excess animal protein increases calcium excretion and lowers citrate.

**Target**

- Normal pregnancy needs (~1.1 g/kg/day), not high-protein diets

**Prefer**

- Fish
- Poultry
- Eggs
- Plant proteins (lentils, beans—watch portions)

**Limit**

- Large portions of red meat
- Processed meats

**6. Citrate: protective**

Citrate prevents calcium oxalate crystals from forming.

**Increase naturally**

- Lemon or lime juice in water
- Oranges
- Melons

(Do not take potassium citrate unless prescribed.)

**7. Vitamins & supplements (important in pregnancy)**

- **Avoid high-dose vitamin C** (>500 mg/day) → increases oxalate
- Continue **prenatal vitamin** as prescribed
- Do not add calcium or vitamin D unless advised by your OB/nephrologist

**Sample Day (Stone-Preventive & Pregnancy-Safe)**

**Breakfast**

- Oatmeal with milk
- Banana
- Water with lemon

**Lunch**

- Grilled chicken salad (mixed greens, not spinach)
- Yogurt
- Olive oil & lemon dressing

**Snack**

- Apple + cheese

**Dinner**

- Salmon
- Rice
- Steamed broccoli
- Water

**Fluids throughout the day**

**When to follow up**

After pregnancy, a **repeat 24-hour urine test** is usually recommended to reassess calcium, sodium, and citrate and fine-tune prevention.

**Key takeaways**

- Hydrate aggressively
- **Lower sodium**
- **Keep normal calcium intake**
- Moderate oxalate + pair with calcium
- Avoid excess vitamin C
- All recommendations are **safe in pregnancy**

If you want, I can:

- Create a **pregnancy-safe stone-prevention meal plan**
- Translate this into a **shopping list**
- Adjust advice for **gestational diabetes, nausea, or food aversions**

Just tell me.

**Case ID: C24**

**Output (Agent):**

1. Metabolic Diagnosis:

- Severe low urine volume (low fluid intake / high fluid losses): 0.8 L/day is markedly below stone-prevention targets and is a major driver of high calcium oxalate supersaturation even if all other urine chemistries are normal.

- Given the comorbidity, this is very likely intentional fluid restriction due to urinary incontinence.

2. Key Dietary Modifications: (Top 3 priorities)

1) Increase total fluid intake strategically to reach a safer urine output

- Goal (typical guideline target): ≥2.0–2.5 L urine/day. With significant incontinence, a pragmatic first step is to increase urine volume to ≥1.5 L/day, then titrate upward as feasible.

- Use scheduled drinking rather than “drink whenever,” and distribute fluids earlier in the day (see below).

2) Choose fluids that are practical and stone-safe

- Prefer water, and if tolerated, citrate-containing low-sugar options (e.g., water with lemon/lime).

- Limit sugar-sweetened beverages (can raise stone risk) and avoid excessive grapefruit juice (drug interactions). Moderate coffee/tea are usually acceptable if they help total volume.

3) Reduce evening/nighttime fluids while increasing daytime intake (to accommodate incontinence/nocturia)

- Front-load fluids morning through late afternoon; consider minimal fluids 2–3 hours before bed (unless medically contraindicated), while maintaining total daily intake.

3. Sample Menu Suggestions: (fluid-focused examples tailored to low urine volume + incontinence)

A practical “timed fluid” day (example):

- On waking: 250–300 mL water

- With breakfast: 250 mL (water/tea)

- Mid-morning: 250 mL

- With lunch: 300 mL

- Mid-afternoon: 250–300 mL

- With dinner: 150–250 mL

- After dinner: only small sips if thirsty / with meds

Fluids/foods that help hydration without large boluses:

- Water; sparkling water if preferred

- Water with lemon/lime (unsweetened)

- Soups with controlled sodium (too much sodium can increase calciuria)

- High-water foods: melon, grapes, oranges, cucumber, zucchini (useful adjuncts, not a substitute for fluids)

Limit if they worsen urgency/leakage (individual response):

- Large single boluses of fluid

- Caffeine late in the day (may worsen urgency in some)

- Alcohol (can worsen urgency/falls risk)

4. Safety Warning:

- At age 80 with BMI 20, avoid overly aggressive fluid loading that increases falls risk from rushing to the bathroom, and watch for hyponatremia if drinking large volumes of very low-solute fluids. Increase fluids gradually (e.g., +250 mL/day every few days).

- If there is any history of heart failure, advanced CKD, or edema, fluid targets must be individualized—ask before aiming for ≥2–2.5 L urine/day.

- Because incontinence is the barrier, stone prevention should be paired with continence strategies: timed voiding/bladder training, pelvic floor therapy, and review of diuretics/caffeine with her clinician.

**Output (Control):**

I’m glad you’re thinking about prevention—**calcium oxalate (CaOx) stones are very preventable**, even at older age. I’ll tailor this to your details (80-year-old woman, low urine volume, urinary incontinence).

**The MOST important issue for you**

Your **urine volume is very low (0.8 L/day)**.
For CaOx stone prevention, the goal is usually **≥2.0–2.5 L urine/day**.

I understand urinary incontinence makes this difficult, but **low urine volume is your #1 risk factor** for recurrence.

We’ll balance hydration *strategically* so it’s manageable.

**What to Eat (and What to Avoid)**

**1. Calcium: Eat NORMAL amounts (do NOT restrict)**

This is critical.

✅ **Target:** ~1,000–1,200 mg/day from food
❌ Do **not** follow a low-calcium diet (this increases stone risk)

**Best calcium sources (with meals):**

- Milk, yogurt, kefir
- Cheese (moderate portions)
- Calcium-fortified plant milks
- Calcium-fortified oatmeal

💡 Calcium **binds oxalate in the gut**, preventing absorption.

⚠️ Avoid calcium supplements unless your doctor specifically recommends them.

**2. Limit High-Oxalate Foods**

You don’t need to eliminate oxalate completely—just avoid the worst offenders.

🚫 **High oxalate foods to limit/avoid:**

- Spinach
- Beets
- Swiss chard
- Rhubarb
- Almonds, peanuts
- Wheat bran
- Dark chocolate
- Black tea (large amounts)

✅ **Lower-oxalate alternatives:**

- Lettuce, cabbage, broccoli
- Green beans
- Apples, pears, berries
- Rice, pasta
- Poultry, fish, eggs

💡 If you eat oxalate foods, **eat them with calcium-containing foods**.

**3. Reduce Salt (Very Important)**

High sodium increases calcium in the urine.

✅ **Goal:** <2,000 mg sodium/day (ideally ~1,500 mg)

🚫 Limit:

- Processed foods
- Canned soups
- Deli meats
- Frozen meals
- Fast food
- Salty snacks

**4. Moderate Animal Protein**

Too much increases calcium and lowers citrate.

✅ **Recommended:**

- Small portions (3–4 oz) of:
  - Chicken
  - Fish
  - Eggs

🚫 Limit:

- Red meat
- Organ meats
- Large portions of meat at every meal

**5. Increase Citrate (Natural Stone Inhibitor)**

Citrate helps prevent calcium stones.

✅ Best sources:

- Lemon juice 🍋
- Lime juice
- Oranges
- Melons

💡 Practical tip:
Add **2–4 tablespoons of lemon juice** to water daily (no sugar).

**Hydration Strategy (Adjusted for Urinary Incontinence)**

Instead of “drink all day,” use **smart timing**:

**✅ Practical plan:**

- Drink **most fluids earlier in the day**
- Small sips every 30–60 minutes (not large boluses)
- Reduce fluids **2–3 hours before bedtime**
- Prioritize fluids **between meals**

**Best fluids:**

- Water
- Water with lemon
- Diluted citrus juice

🚫 Avoid:

- Sugary drinks
- Large amounts of tea
- Cola (phosphate)

**Sample Day (Simple & Realistic)**

**Breakfast**

- Oatmeal with milk
- Blueberries
- Water or lemon water

**Lunch**

- Grilled chicken
- Rice
- Steamed broccoli
- Yogurt

**Snack**

- Orange or melon

**Dinner**

- Fish
- Green beans
- Small portion of pasta
- Cheese or milk

**When to Talk to Your Doctor**

You may benefit from:

- **24-hour urine recheck** after changes
- **Potassium citrate** if citrate is low
- Review of bladder/incontinence management to allow better hydration

**Key Takeaways**

- 💧 Low urine volume is your biggest risk
- 🥛 Eat **normal calcium**, not low
- 🧂 Reduce salt
- 🥬 Limit high-oxalate foods
- 🍋 Increase citrate
- ⏰ Drink fluids strategically due to incontinence

If you’d like, I can:

- Create a **low-oxalate grocery list**
- Suggest **hydration strategies specifically for incontinence**
- Review medications that may affect stones

Just tell me 👍

**Case ID: C25**

**Output (Agent):**

1. Metabolic Diagnosis:

- Hypercalciuria: urine calcium 9.5 mmol/day (≈ 380 mg/day) is elevated and increases CaOx supersaturation.

- High risk for enteric hyperoxaluria after bariatric surgery (especially Roux-en-Y gastric bypass; also possible with other malabsorptive procedures). Even though oxalate wasn’t provided, this comorbidity strongly suggests a second key driver may be hyperoxaluria and/or hypocitraturia contributing to recurrent CaOx stones.

2. Key Dietary Modifications: (Top 3 priorities)

1) Do not restrict calcium; instead ensure adequate calcium with meals (oxalate binding + bone health):

- Target normal calcium intake (~1,000–1,200 mg/day total), preferably as calcium citrate foods/supplement form taken with meals (often better absorbed post-bariatric).

- This is crucial post-bariatric to reduce intestinal oxalate absorption and protect bone; calcium restriction can worsen stone risk.

2) Lower sodium and avoid high animal-protein patterns to reduce urine calcium:

- Sodium goal: ≤2,000 mg/day (≈87 mmol/day). High sodium drives calciuria.

- Avoid “high-protein diet” approaches sometimes used after bariatric surgery; keep protein adequate but not excessive, and distribute through the day.

3) Assume risk of hyperoxaluria: adopt a low-oxalate, low-fat strategy until proven otherwise

- Limit highest-oxalate foods and avoid high-fat meals (fat malabsorption increases oxalate absorption).

- Request/complete a full 24h urine to confirm: oxalate, citrate, volume, sodium, uric acid, pH, and supersaturation.

3. Sample Menu Suggestions: (specific foods to eat/avoid for hypercalciuria + post-bariatric enteric risk)

With each meal: include a calcium source (preferably lower sodium) to bind oxalate

- Examples: milk, plain yogurt, kefir; if supplements are used, calcium citrate with meals (dose per bariatric team).

Lower sodium swaps (to reduce calciuria):

- Choose: fresh meats/fish/eggs, home-cooked meals, “no salt added” canned goods, herbs/spices, lemon.

- Avoid/limit: deli meats, jerky, bacon/sausage, fast food, packaged sauces, chips, salty cheeses.

Low-oxalate pattern (important post-bariatric):

- Prefer: cauliflower, cabbage, zucchini, cucumbers, mushrooms, onions, peppers; apples, grapes, melons; rice/pasta/bread (not bran-heavy).

- Avoid/strongly limit: spinach, Swiss chard, beet greens, almonds/nut flours, wheat bran, rhubarb, cocoa/dark chocolate; “green powders.”

Lower fat approach (to reduce enteric oxalate absorption):

- Choose: baked/grilled lean proteins; small portions of healthy fats.

- Avoid: very high-fat meals (fried foods, heavy cream sauces), which can worsen fat malabsorption and oxalate uptake.

4. Safety Warning:

- Post-bariatric patients are at risk of nutrient deficiencies (calcium, vitamin D, magnesium) and bone loss; any calcium/vitamin D plan should be coordinated with the bariatric team to avoid deficiency while preventing stones.

- If he has reduced urine volume from limited intake, nausea, or dumping, aggressive fluid advice must be individualized; aim for frequent small fluid doses between meals.

- This case is incomplete without urine oxalate and citrate —both are commonly abnormal post-bariatric and would change priorities (e.g., need for alkali/citrate therapy vs intensified oxalate-binding strategy).

**Output (Control):**

I’m glad you’re thinking about prevention—**recurrent calcium oxalate (CaOx) stones after bariatric surgery** need a *very targeted* approach. Below is **evidence-based dietary guidance tailored to your profile** (45-year-old male, BMI 35, post-bariatric surgery, recurrent CaOx stones, urine Ca 9.5 mmol/day).

⚠️ This is educational guidance, not a substitute for care from your urologist/nephrologist or dietitian—especially important post-bariatric surgery.

**Your Main Stone Risks**

You likely have **multiple contributors**:

1. **Enteric hyperoxaluria** (common after bariatric surgery)
2. **High urinary calcium**
3. **Low urine volume**
4. Possibly **low urinary citrate**

Diet can significantly reduce recurrence.

**What You SHOULD Eat (and How)**

**1. Fluids: Your #1 Priority**

**Goal:**

- **Urine output ≥ 2.5–3.0 L/day**
- Usually means **3–3.5 L fluid intake daily**

**Best choices:**

- Water (mainstay)
- Lemon or lime water (natural citrate)
- Diluted citrus juices (no added sugar)

❌ Avoid: sugary drinks, excessive cola (phosphoric acid)

**Tip:** Small, frequent sips work best post-bariatric surgery.

**2. Calcium: Eat Enough (With Meals!)**

This is **critical** after bariatric surgery.

**Goal:**

- **1,000–1,200 mg/day of calcium**, ideally **with meals**

**Best sources:**

- Low-fat milk or yogurt
- Cheese (moderate amounts)
- Fortified plant milks (check calcium content)

**If supplements are needed:**

- ✅ **Calcium citrate** (preferred post-bariatric)
- Take **with meals**, not between meals

❌ Avoid calcium restriction — this *increases* oxalate absorption and stone risk.

**3. Oxalate: Limit High-Oxalate Foods**

Because bariatric surgery increases oxalate absorption, this matters more for you than most patients.

**Avoid or strictly limit:**

- Spinach
- Rhubarb
- Beets
- Nuts (especially almonds)
- Nut butters
- Chocolate, cocoa
- Bran, wheat germ
- Sweet potatoes
- Black tea

**Lower-oxalate alternatives:**

- Kale, cabbage
- Broccoli, cauliflower
- Rice, pasta
- Apples, bananas, berries
- Lean meats, eggs

**Key rule:**
👉 **Eat calcium and oxalate together** (e.g., dairy with meals) so oxalate binds in the gut instead of your kidneys.

**4. Fat Intake: Moderate (Very Important Post-Bariatric)**

Excess fat binds calcium in the gut → leaves oxalate unbound → ↑ oxalate absorption.

**Aim for:**

- Moderate fat intake
- Avoid greasy/fried foods

Choose:

- Lean proteins
- Baked or grilled foods
- Small amounts of olive oil

**5. Sodium: Keep It LOW**

High sodium directly increases urinary calcium.

**Goal:**

- **<2,000 mg sodium/day** (ideally <1,500 mg)

❌ Limit:

- Processed foods
- Fast food
- Cured meats
- Packaged snacks

✅ Choose:

- Fresh foods
- Herbs/spices instead of salt

**6. Protein: Moderate, Not High**

Too much animal protein increases calcium excretion and lowers citrate.

**Target:**

- ~0.8–1.0 g/kg *ideal* body weight per day

**Better choices:**

- Fish
- Poultry
- Eggs
- Some plant proteins (but avoid high-oxalate ones)

❌ Avoid high-protein / keto diets

**7. Citrate: Natural Stone Protection**

Citrate prevents calcium stones.

**Increase citrate via:**

- Lemon or lime juice (½ cup/day diluted)
- Oranges (in moderation)

Your doctor may consider **potassium citrate** if urine citrate is low.

**8. Vitamins & Supplements: Be Careful**

❌ Avoid:

- **Vitamin C > 500 mg/day** (converts to oxalate)

✅ Often needed post-bariatric:

- Calcium citrate
- Vitamin D (as directed by labs)

Always coordinate supplements with your clinician.

**Sample Daily Eating Pattern**

**Breakfast**

- Greek yogurt + berries
- Water or lemon water

**Lunch**

- Grilled chicken
- Rice + broccoli
- Low-fat cheese or milk

**Snack**

- Banana or apple

**Dinner**

- Fish
- Pasta or potatoes
- Cauliflower or zucchini

**Fluids throughout the day**

**What to Discuss With Your Doctor**

Given your history, ask about:

- 24-hour urine follow-up (oxalate, citrate, volume, sodium)
- Calcium citrate dosing
- Potassium citrate
- Fat malabsorption management
- Dietitian referral experienced in **post-bariatric stone prevention**

If you want, I can:

- Build a **personalized low-oxalate meal plan**
- Help you read food labels for sodium & oxalate
- Translate this into a **post-bariatric eating schedule**

Just tell me 👍

**Case ID: C26**

**Output (Agent):**

1. Metabolic Diagnosis:

- Idiopathic (non–sodium-driven) hypercalciuria in a recurrent CaOx stone former: urine calcium 8.0 mmol/day (≈ 320 mg/day) is elevated, while urine sodium 100 mmol/day (≈ 2,300 mg sodium/day) is not high and is already near guideline sodium targets.

- This pattern suggests hypercalciuria is less likely due to excess salt and more consistent with idiopathic renal calcium leak / high animal protein load / high refined sugar intake / high calcium supplementation, or simply genetic predisposition. (We’d also want urine volume and citrate to complete risk stratification.)

2. Key Dietary Modifications: (Top 3 priorities)

1) Keep sodium low (don’t loosen it), but shift focus to protein and “acid load”:

- Maintain sodium around the current level or slightly lower (roughly ≤2,000–2,300 mg/day sodium).

- Avoid “hidden salt” increases that could further raise urine calcium.

2) Moderate animal protein (reduce calciuria and protect citrate):

- Aim for ~0.8–1.0 g/kg/day protein unless there’s a competing need (athletic training, etc.).

- Practical: keep animal protein to ~1 palm-sized portion per meal and add more plant proteins (lentils/beans in reasonable portions).

3) Maintain normal dietary calcium with meals; avoid unnecessary calcium supplements

- Keep dietary calcium ~1,000–1,200 mg/day (do not restrict for CaOx), spread across meals to reduce oxalate absorption.

- If taking calcium supplements without a clear indication, consider stopping or reducing and meeting calcium via food (coordinate with his clinician if prescribed for bone health).

3. Sample Menu Suggestions:

Eat more often (supports lower urine calcium risk profile):

- Breakfast: oats with milk/yogurt + fruit; or eggs with whole-grain toast (not heavily salted)

- Lunch: chicken/fish salad bowl with mixed low-oxalate vegetables + olive oil/lemon dressing; yogurt

- Dinner: smaller portion of meat (fish/poultry) + rice/potatoes + vegetables; add a plant-protein meal a few times/week (lentil soup, bean chili—watch sodium)

- Snacks: fruit, yogurt, unsalted nuts (moderation), vegetables

Limit/avoid (can worsen hypercalciuria despite low sodium):

- Large portions of red meat/processed meat; “high-protein diet” patterns and frequent protein shakes

- High sugar loads (sugary drinks, desserts)—can increase urinary calcium in some individuals

- Excess calcium supplements (unless medically necessary)

Keep (do not restrict):

- Normal calcium foods with meals (milk, yogurt, calcium-containing alternatives) to help bind dietary oxalate

4. Safety Warning:

- No major comorbidity-based restrictions identified.

- To fully personalize prevention per AUA/EAU approach, he still needs the rest of the 24h urine (especially volume, citrate, oxalate, pH, uric acid). If urine volume is low or citrate is low, the top 3 priorities may change (fluids/citrate can outweigh protein changes).

**Output (Control):**

Given your history of **recurrent calcium oxalate (CaOx) stones** and **high urinary calcium (hypercalciuria)** with **low urinary sodium**, the goal is to **reduce urinary calcium and oxalate supersaturation while maintaining overall nutrition**. Below is evidence-based, practical dietary advice tailored to your data.

**Core Principles (Most Important)**

1. **High fluid intake**
2. **Normal dietary calcium (do NOT restrict calcium)**
3. **Limit oxalate absorption**
4. **Moderate animal protein**
5. **Maintain low sodium (you’re already doing well here)**
6. **Increase urinary citrate via fruits & vegetables**

**1. Fluids (Highest Impact)**

**Target:**

- Produce **≥2.5–3.0 L urine/day** (usually means drinking ~3 L/day)

**Best choices:**

- Water (primary)
- Citrus-based drinks (lemon or lime water)

**Why:**
Dilutes calcium and oxalate → lowers stone formation risk.

**Tip:**
Add **½ cup lemon juice daily** (≈30–40 mEq citrate).

**2. Calcium Intake (Very Important)**

**Target:**

- **1,000–1,200 mg/day from food**

**Good sources (with meals):**

- Milk, yogurt, kefir
- Cheese (moderate amounts)
- Calcium-fortified plant milks

**Why this matters:**
Low dietary calcium **increases oxalate absorption**, worsening CaOx stones—even if urine calcium is high.

🚫 **Avoid calcium supplements** unless prescribed (they raise stone risk if not taken with meals).

**3. Oxalate Management**

You don’t need to eliminate oxalate—just **pair it with calcium and avoid excess**.

**Limit or avoid (especially without calcium):**

- Spinach
- Rhubarb
- Beets
- Almonds, cashews
- Wheat bran
- Dark chocolate
- Black tea
- Soy products (tofu, soy milk)

**Lower-oxalate alternatives:**

- Kale, bok choy, cabbage
- Broccoli, cauliflower
- Apples, pears, bananas
- White rice, pasta
- Poultry, fish, eggs

**Key strategy:**
👉 Eat oxalate-containing foods **with calcium-rich foods**.

**4. Protein (Moderate, Not High)**

**Target:**

- ~0.8–1.0 g/kg/day (≈60–75 g/day for most men)

**Limit:**

- Red meat
- Processed meats
- Excess poultry or fish

**Why:**
Animal protein increases urinary calcium and lowers citrate.

**Better options:**

- Plant proteins (lentils, chickpeas)
- Fish in moderation
- Balanced mixed meals

**5. Sodium (You’re Doing Well)**

Your urine sodium (100 mmol/day) suggests **good adherence**.

**Goal:**

- **<2,300 mg sodium/day**
- Ideally **<1,800 mg/day**

**Why:**
Higher sodium → higher urinary calcium.

Keep doing what you’re doing here 👍

**6. Increase Citrate (Natural Stone Inhibitor)**

Citrate binds calcium and prevents crystal growth.

**Best sources:**

- Lemons, limes
- Oranges
- Melons
- Avocados
- Vegetables in general

**Avoid:**
Low-carb / high-protein diets (they reduce citrate).

**7. Sugar & Refined Carbs**

**Limit:**

- Sugar-sweetened beverages
- Fructose-heavy foods
- Desserts and candy

**Why:**
Sugar increases urinary calcium and oxalate.

**Example Day (Stone-Smart)**

**Breakfast:**

- Yogurt with berries
- Oatmeal with milk
- Lemon water

**Lunch:**

- Grilled chicken
- White rice
- Steamed broccoli
- Olive oil

**Snack:**

- Apple or pear
- Cheese stick

**Dinner:**

- Baked salmon
- Roasted cauliflower
- Mixed greens (no spinach)

**Fluids throughout the day:**

- Water + citrus

**Medical Note (Non-Diet)**

Because your urinary calcium is high **despite low sodium**, many patients benefit from:

- **Thiazide diuretics** (discuss with your physician)
- Periodic **24-hour urine monitoring**

**Bottom Line**

✔ Drink more
✔ Eat **normal calcium**
✔ Pair calcium with oxalate
✔ Moderate protein
✔ Keep sodium low
✔ Eat fruits & vegetables daily

If you want, I can:

- Build a **weekly meal plan**
- Tailor advice to **vegetarian / low-carb preferences**
- Interpret a **full 24-hour urine panel**

**Case ID: C27**

**Output (Agent):**

1. Metabolic Diagnosis:

- Recurrent calcium oxalate stone disease with no identifiable metabolic abnormality on 24‑hour urine (“normometabolic stone former”).

- This commonly still reflects episodic risk (intermittently low urine volume, variable sodium/protein intake, or missed collections), and/or non-diet factors (anatomy, Randall’s plaques, genetics). With 5 events, she is high recurrence-risk despite “normal” labs.

2. Key Dietary Modifications: (Top 3 priorities)

1) Optimize urine volume consistently (highest-yield even when labs are “normal”):

- Aim for ≥2.0–2.5 L urine/day (usually requires ~2.5–3.0 L fluid/day depending on sweat).

- Use a simple routine: a bottle with markings + “drink on schedule” (reduces reliance on anxious monitoring).

2) Keep sodium modest and maintain normal calcium intake with meals:

- Sodium goal: ≤2,000–2,300 mg/day (avoid wide day-to-day swings).

- Calcium: 1,000–1,200 mg/day from food, spread with meals (do not restrict; helps bind oxalate).

3) Avoid high-risk extremes and supplements:

- Avoid high-dose vitamin C supplements and “detox/green powders” (can raise oxalate).

- Avoid very high animal-protein patterns; keep portions moderate and balanced.

3. Sample Menu Suggestions:

Simple, low-stress pattern (repeatable):

- Breakfast: yogurt + fruit + oats; water

- Lunch: turkey/chicken sandwich (not processed deli if possible) + salad; water

- Dinner: fish/chicken/tofu + rice/potatoes + vegetables; water with lemon/lime

- Snacks: fruit, yogurt, unsalted nuts (small portion)

Avoid/limit:

- Sugar-sweetened beverages; frequent restaurant/fast food (high sodium)

- Vitamin C supplements (>~500 mg/day), “superfood” powders

- Large, frequent portions of processed meats

4. Safety Warning:

- No medical contraindications noted.

- Anxiety consideration: avoid overly restrictive rules that increase preoccupation. Use 2–3 repeatable habits (e.g., “finish 1 bottle by noon, 1 by 5 pm, 1 by bedtime”) rather than tracking many nutrients.

- With 5 recurrences despite “normal” urine, confirm: stone analysis truly CaOx, adequacy of 24h urine collection (creatinine), and consider repeating 24h urine on a typical day and assessing imaging/anatomical factors with urology.

**Output (Control):**

I’m sorry you’re dealing with recurrent stones — that’s frustrating, especially when your metabolic workup is normal. The good news is that **dietary strategies are very effective for preventing calcium oxalate (CaOx) stone recurrence**, even when labs look “normal.”

Below is **evidence-based, practical advice tailored to recurrent CaOx stones** like yours.

**1. Fluids (Most Important)**

**Goal:**

- **Urine output ≥ 2.5 L/day** (usually means drinking ~3 L / 100 oz fluid daily)

**Best choices**

- Water (primary)
- Lemon or lime water (natural citrate)
- Low-sugar citrus drinks

**Limit**

- Sugary sodas
- Grapefruit juice (associated with higher stone risk)
- Excess alcohol

**Tip:**
Your urine should be **pale yellow to clear** most of the day.

**2. Calcium: Eat Normal Amounts (Do NOT Restrict)**

This is a common misconception.

**Target intake:**

- **1,000–1,200 mg/day from food**

**Good sources**

- Milk, yogurt, kefir
- Cheese (moderate amounts)
- Calcium-fortified plant milks
- Calcium-set tofu

**Why this helps:**
Dietary calcium **binds oxalate in the gut**, preventing absorption and stone formation.

⚠️ **Avoid calcium supplements** unless specifically advised by your doctor — supplements increase stone risk when taken without meals.

**3. Oxalate: Moderate, Don’t Eliminate**

You do **not** need an extreme low-oxalate diet, but avoid excess.

**High-oxalate foods to limit**

- Spinach, Swiss chard
- Beets
- Rhubarb
- Almonds, cashews
- Peanut butter
- Dark chocolate
- Wheat bran

**Lower-oxalate alternatives**

- Kale, bok choy
- Broccoli, cauliflower
- Blueberries, strawberries
- Rice, oats
- Pumpkin seeds (small amounts)

**Key strategy:**
👉 **Eat oxalate foods WITH calcium-containing meals** (e.g., yogurt with berries).

**4. Sodium: Keep It Low**

**Target:**

- **< 2,000 mg sodium/day** (ideally closer to 1,500 mg)

High sodium increases urinary calcium.

**Reduce**

- Processed foods
- Restaurant meals
- Canned soups
- Salty snacks

**Tip:**
Check labels — “healthy” foods are often sodium-heavy.

**5. Protein: Moderate Animal Protein**

**Limit excessive intake**, especially:

- Red meat
- Poultry
- Fish
- Eggs (moderation is fine)

**Target**

- ~0.8–1.0 g/kg/day total protein

**Encourage**

- Plant proteins (beans, lentils — eaten with calcium foods)
- Dairy protein

**6. Increase Citrate (Natural Stone Inhibitor)**

Citrate prevents calcium crystals from forming.

**Best sources**

- Lemons, limes (½ cup lemon juice/day if tolerated)
- Oranges
- Melons

**Simple habit**

- Lemon water daily (no sugar or minimal sweetener)

**7. Avoid Excess Vitamin C**

- **Do not exceed 500–1,000 mg/day**
- High vitamin C converts to oxalate

**8. Supplements to Be Careful With**

Avoid unless medically indicated:

- High-dose vitamin D
- Calcium supplements
- Herbal “detox” products

Magnesium **may help**, but only if advised by your clinician.

**9. Anxiety & Stones (Often Overlooked)**

Anxiety can indirectly increase risk via:

- Dehydration
- Skipped meals
- High caffeine or soda intake

**Helpful strategies**

- Scheduled hydration reminders
- Stress-reduction routines (yoga, walking)
- Avoid using soda or energy drinks as coping tools

**10. Sample Day (Stone-Friendly)**

**Breakfast**

- Greek yogurt + blueberries
- Oatmeal
- Water or lemon water

**Lunch**

- Grilled chicken (moderate portion)
- Quinoa
- Steamed broccoli
- Olive oil + herbs

**Snack**

- Apple with cheese

**Dinner**

- Salmon or tofu
- Brown rice
- Roasted vegetables (non-spinach)

**Fluids throughout day**

- Water + lemon/lime

**When to Consider Medical Therapy**

Even with normal labs, **recurrent stones (x5)** may justify:

- Potassium citrate
- Thiazide diuretic (if hypercalciuria develops)

This is something to discuss with your nephrologist/urologist.

**Bottom line**

For you, **hydration + normal dietary calcium + low sodium + moderate oxalate** will give the biggest benefit.

If you’d like, I can:

- Create a **personalized grocery list**
- Review a **7-day meal plan**
- Help you identify **hidden oxalate/sodium traps** in your current diet

Just tell me 👍

**Case ID: C28**

**Output (Agent):**

1. Metabolic Diagnosis:

- Persistently acidic urine (low urine pH 5.3) causing high uric acid supersaturation → uric acid nephrolithiasis.

- In the setting of type 2 diabetes/metabolic syndrome, this is very commonly due to impaired renal ammoniagenesis/acid excretion, not simply “too much purine,” though purine load can contribute.

2. Key Dietary Modifications: (Top 3 priorities)

1) Raise urine pH (main priority for uric acid stones):

- Use alkali from diet: increase vegetables and certain fruits; use citrate-containing fluids (e.g., water with lemon/lime).

- Goal urine pH for prevention is typically ~6.0–6.5 (do not overshoot chronically much above ~6.5 without supervision due to CaP risk). Diet alone may not be enough at pH 5.3; many patients ultimately require prescribed alkali (e.g., potassium citrate), but you asked for dietary strategies.

2) Limit purine-rich animal protein and avoid high-protein/low-carb diets (also helps diabetes):

- Reduce red meat and organ meats; keep portions moderate and shift protein toward plant sources and lower-purine options.

3) Reduce sugar/fructose and improve insulin resistance pattern:

- Avoid sugar-sweetened beverages, fruit juice, and high-fructose snacks, which raise uric acid production and worsen insulin resistance—both promote uric acid stone risk.

3. Sample Menu Suggestions:

Emphasize (alkali-producing, diabetes-friendly):

- Breakfast: plain Greek yogurt or eggs + vegetables; berries; water/coffee (unsweetened)

- Lunch: big salad (greens, cucumber, peppers) + olive oil/lemon dressing + grilled chicken or tofu; water with lime

- Dinner: salmon or poultry (moderate portion) + roasted vegetables; small portion of whole grains/legumes as tolerated for glycemic control

- Snacks: nuts (small portions), cheese, vegetables with hummus, berries

Limit/avoid (uric acid + diabetes risk):

- Organ meats (liver), large portions of beef/lamb, meat gravies

- Anchovies/sardines and some shellfish frequently (purine-dense)

- Beer and spirits (alcohol increases urate and dehydration risk)

- Sugary drinks/juice (including “natural” fruit juice), sweetened iced tea, energy drinks

- Very low-carb “keto”/high-protein diets (often worsen urine acidity)

4. Safety Warning:

- If you are on diabetes medications that increase dehydration risk (e.g., SGLT2 inhibitors ), prioritize steady hydration and discuss stone prevention with your clinician.

- If alkali therapy is prescribed later (commonly needed when pH is 5.3), it should be monitored—especially if there is any CKD or risk of hyperkalemia.

- One value (pH) is enough to identify the primary driver for uric acid stones, but full 24h urine (volume, uric acid excretion, citrate, sodium) helps tailor diet vs medication intensity.

**Output (Control):**

I’m glad you’re thinking about prevention—**uric acid stones are very preventable**, especially when diet and urine pH are addressed. I’ll tailor this to your profile: **55-year-old male, BMI 30, uric acid stones, urine pH 5.3 (acidic), type 2 diabetes**.

**The Big Picture (Most Important Points)**

Uric acid stones form when:

1. **Urine is too acidic** (pH < 5.5) ← *this is your main risk*
2. **Uric acid levels are high**
3. **Urine volume is low**

👉 Your prevention strategy focuses on:

- **Alkalinizing urine**
- **Reducing uric acid production**
- **Increasing urine volume**
- **Keeping blood sugar controlled**

**1. Fluids (Non-Negotiable)**

**Goal:** Produce **≥2.5 liters of urine/day**
This usually means **3 liters (≈100 oz) of fluids daily**, more if you sweat.

**Best choices**

- Water (primary)
- Sparkling water
- Water with lemon or lime 🍋 (alkalinizing)
- Unsweetened herbal tea

**Avoid / limit**

- Sugary drinks (worsen diabetes & stone risk)
- Fructose-heavy juices (apple juice, soda)
- Excess alcohol (especially beer)

**2. Raise Urine pH (Critical for You)**

Your urine pH of **5.3 is too acidic**. The target is **pH 6.0–6.5**.

**Foods that help alkalinize urine**

Eat **more of these daily**:

- Vegetables (very important)
  - Spinach, kale, broccoli, cauliflower
  - Zucchini, cucumber, bell peppers
- Fruits (moderate portions for diabetes)
  - Citrus fruits (lemon, lime, orange)
  - Berries
  - Melon
- Legumes (lentils, chickpeas – moderate portions)

💡 Lemon or lime juice in water is especially helpful.

**Foods that acidify urine (limit)**

- Red meat
- Organ meats
- Shellfish
- High amounts of animal protein

Many patients with uric acid stones ultimately need **potassium citrate** to reliably raise urine pH—this is something to discuss with your physician.

**3. Protein: Right Amount, Right Type**

**Target:** ~0.8–1.0 g/kg/day (not high-protein)

**Limit high-purine foods (very important)**

Avoid or strictly limit:

- Red meat (beef, pork, lamb)
- Organ meats (liver, kidney)
- Anchovies, sardines
- Shellfish
- Game meats

**Better protein choices**

- Plant proteins (beans, tofu, lentils)
- Eggs
- Low-fat dairy
- Fish (salmon, tilapia) in **moderate** amounts

**4. Carbohydrates & Diabetes Control**

Poor glucose control → more acidic urine.

**Choose**

- Whole grains (moderate portions)
- Non-starchy vegetables
- Low-glycemic fruits (berries)

**Limit**

- Refined carbs (white bread, pastries)
- Added sugars
- High-fructose corn syrup (strongly linked to uric acid stones)

**5. Fats & Weight Management**

Your BMI of 30 increases stone risk.

**Favor**

- Olive oil
- Nuts (moderate portions)
- Avocado

Weight loss (even 5–10%) can:

- Improve insulin resistance
- Raise urine pH
- Lower uric acid production

**6. Salt (Sodium)**

**Limit sodium to <2,300 mg/day**

- High salt increases stone risk and worsens diabetes and blood pressure

Avoid:

- Processed foods
- Fast food
- Salty snacks

**7. Sample Day of Eating (Stone- & Diabetes-Friendly)**

**Breakfast**

- Scrambled eggs with spinach and tomatoes
- ½ avocado
- Water with lemon

**Lunch**

- Grilled salmon (small portion)
- Quinoa or brown rice (½ cup)
- Steamed broccoli and zucchini

**Snack**

- Greek yogurt or nuts (small handful)

**Dinner**

- Lentil or tofu stir-fry with mixed vegetables
- Olive oil & herbs
- Sparkling water with lime

**8. Monitoring (Very Helpful)**

- **Check urine pH at home** (pH strips)
  - Aim for **6.0–6.5**
- Periodic **24-hour urine testing**
- Tight glycemic control (A1c goal per your physician)

**Key Takeaway**

For you, **urine alkalinization + hydration + reduced animal protein** are the most powerful tools to prevent recurrence. Diet alone helps, but many patients with uric acid stones benefit from **medical alkalinization** in addition to diet.

If you want, I can:

- Create a **7-day meal plan**
- Help you interpret urine pH results
- Explain **potassium citrate vs diet alone** in more detail

**Case ID: C29**

**Output (Agent):**

1. Metabolic Diagnosis:

- Marked hyperoxaluria: urine oxalate 0.8 mmol/day (≈ 72 mg/day), substantially above common targets (often >0.45 mmol/day / >40 mg/day).

- In a CaOx stone former on a vegan diet, this is most consistent with dietary hyperoxaluria (high intake of oxalate-rich plants and/or low calcium co-ingestion reducing gut oxalate binding). Enteric hyperoxaluria is possible but not suggested from the data provided.

2. Key Dietary Modifications: (Top 3 priorities)

1) Lower the highest-oxalate plant foods (target the “worst offenders,” not all plants):

- The biggest gains usually come from removing/limiting: spinach, chard, beet greens, almonds/almond flour, tahini/sesame in large amounts, cocoa/dark chocolate, wheat bran , and frequent large portions of beets.

2) Increase calcium with meals using vegan sources (do NOT restrict calcium):

- Goal: ~1,000–1,200 mg/day calcium, taken with oxalate-containing meals to bind oxalate in the gut and lower absorption.

- Use calcium-fortified plant milks/yogurts, calcium-set tofu (calcium sulfate), and (if acceptable) calcium citrate supplements with meals.

3) Avoid “stacking oxalate” via vegan specialty products and supplements:

- Limit green smoothies/juice blends (often spinach-based), “supergreens” powders, and frequent almond-based products (almond milk, almond flour baking) if not calcium-fortified and used in large quantities.

- Avoid high-dose vitamin C supplements (ascorbate → oxalate).

3. Sample Menu Suggestions:

Better vegan swaps (lower oxalate + calcium pairing):

- Breakfast: calcium-fortified soy milk or pea milk + low-oxalate cereal/oats; berries/banana

- Avoid: spinach smoothie, almond-flour muffins daily, cocoa-heavy smoothies

- Lunch: large salad built on romaine/iceberg + cucumbers/peppers + beans/lentils; add calcium-set tofu or a calcium-fortified yogurt

- Avoid: spinach/chard base, beet greens, heavy tahini dressing

- Dinner: stir-fry with calcium-set tofu + cauliflower/cabbage/zucchini + rice

- Avoid: frequent beet dishes; large nut-based sauces

- Snacks: fruit; carrots; hummus (moderate); calcium-fortified yogurt

- Avoid: daily large servings of almonds, dark chocolate as a staple

Foods to emphasize (generally lower oxalate):

- Vegetables: cabbage, cauliflower, mushrooms, onions, zucchini, cucumbers, lettuce

- Fruits: apples, grapes, melons, mango, peaches

- Starches: rice, pasta, potatoes, corn-based products (watch added salt)

4. Safety Warning:

- Vegan diets can be low in calcium unless intentionally planned; inadequate calcium can worsen hyperoxaluria and may harm bone health. Use fortified foods and/or supplements deliberately.

- If you have any GI malabsorption symptoms (chronic diarrhea, steatorrhea) or prior bowel surgery, tell your clinician—management shifts toward enteric hyperoxaluria strategies.

- To fully personalize, we still need urine volume, calcium, citrate, sodium, and pH; low citrate or low volume often coexist and change priorities (e.g., hydration/citrate strategies).

**Output (Control):**

I’m not a doctor, but I can give you **evidence-based dietary guidance** commonly used to reduce **calcium oxalate (CaOx) kidney stone recurrence**, tailored to your profile (40-yo male, BMI 28, **high urine oxalate**, **vegan diet**).

**Your Key Risk Factors (what we’re targeting)**

- **History of calcium oxalate stones**
- **High urine oxalate (0.8 mmol/day)** → major driver of recurrence
- **Vegan diet** → healthy overall, but often **higher in oxalate** and sometimes **low in calcium**
- **BMI 28** → modestly increases stone risk

**What You SHOULD Eat (and How)**

**1. Get Enough Calcium — Even on a Vegan Diet**

This is *critical* and often misunderstood.

**Goal:** ~**1,000–1,200 mg calcium/day**, **with meals**

Why:

- Calcium binds oxalate **in the gut**, preventing absorption → **less oxalate in urine**
- Low calcium intake actually **increases CaOx stone risk**

**Vegan calcium sources (low-oxalate preferred):**

- Calcium-fortified plant milks (soy, almond, oat) ✔
- Calcium-set tofu (check label for “calcium sulfate”) ✔
- Fortified orange juice ✔
- Bok choy, kale, turnip greens ✔ (low oxalate)
- Calcium supplements **with meals only** if diet is insufficient

⚠️ Avoid taking calcium supplements **between meals**.

**2. Limit High-Oxalate Foods (This Is Priority #1 for You)**

Given your **high urine oxalate**, restriction matters.

**Strongly limit or avoid:**

- Spinach 🚫
- Beets 🚫
- Swiss chard 🚫
- Rhubarb 🚫
- Almonds, cashews 🚫
- Tahini / sesame seeds 🚫
- Wheat bran 🚫
- Dark chocolate / cocoa 🚫

**Moderate intake (pair with calcium):**

- Sweet potatoes
- Soy products
- Raspberries
- Black beans
- Quinoa

👉 **Always eat oxalate-containing foods with calcium** (e.g., tofu + kale).

**3. Increase Citrate (Natural Stone Inhibitor)**

Citrate prevents calcium stones from forming.

**Good vegan sources:**

- Lemons & limes (best) 🍋
- Oranges
- Grapefruit (if no medication interactions)

**Practical tip:**

- Lemon water daily (½ cup lemon juice spread through the day)

**4. Hydration Is Non-Negotiable**

**Goal:** ≥ **2.5–3.0 L urine/day**
(usually ~3–3.5 L fluid intake)

Best fluids:

- Water
- Citrus-based water
- Diluted lemonade (low sugar)

Avoid:

- Excess iced tea (oxalate)
- Sugary drinks

**5. Reduce Sodium**

High sodium → more calcium in urine.

**Goal:** < **2,300 mg sodium/day** (ideally <1,800)

Tips:

- Limit processed vegan foods (meat substitutes, canned soups)
- Choose unsalted nuts/seeds (or avoid high-oxalate ones)
- Read labels carefully

**6. Moderate Vitamin C Intake**

Excess vitamin C → converts to oxalate.

- Keep **<1,000 mg/day**
- Avoid high-dose supplements

**7. Adequate (Not Excess) Protein**

Plant protein is safer than animal protein, but excess still matters.

- Aim for **moderate protein**
- Emphasize lentils, chickpeas, tofu (with calcium)

**Sample Day (Vegan, Low-Oxalate)**

**Breakfast**

- Fortified soy milk
- Oatmeal (moderate portion)
- Blueberries
- Calcium-set tofu scramble

**Lunch**

- Kale & bok choy salad
- Chickpeas
- Lemon-olive oil dressing
- Fortified orange juice

**Snack**

- Apple
- Low-oxalate crackers

**Dinner**

- Brown rice
- Stir-fried tofu (calcium-set)
- Zucchini, carrots, mushrooms

**Fluids**

- Water + lemon throughout the day

**Weight & Lifestyle**

- Gradual weight loss (if appropriate) reduces stone risk
- Regular physical activity helps metabolic health

**Follow-Up That Would Improve Precision**

If available, these labs help fine-tune advice:

- Urine calcium
- Urine citrate
- Urine volume
- Urine sodium
- Serum calcium / PTH (if recurrent)

If you’d like, I can:

- Design a **1-week vegan, low-oxalate meal plan**
- Review a **food list you eat regularly**
- Help interpret a **24-hour urine test**

Just tell me 👍

**Case ID: C30**

**Output (Agent):**

1. Metabolic Diagnosis:

- Medication-associated calcium phosphate (CaP) stone risk from topiramate, characterized by inappropriately alkaline urine (urine pH 6.5) due to carbonic anhydrase inhibition (renal tubular acidosis–like effect). This favors CaP supersaturation and is often accompanied by hypocitraturia (not provided) and sometimes hypercalciuria.

2. Key Dietary Modifications: (Top 3 priorities)

1) Maximize fluid to lower CaP supersaturation (most reliable dietary lever):

- Target urine volume ≥2.0–2.5 L/day (unless a clinician has restricted fluids). Spread intake across the day.

2) Avoid urine-alkalinizing supplements/behaviors unless specifically directed:

- Do not self-start potassium citrate, sodium bicarbonate, “alkaline waters,” or high-dose alkali supplements without urology guidance, because further increasing urine pH can worsen CaP risk.

- Maintain a balanced diet; don’t pursue “alkaline diet” extremes.

3) Lower dietary sodium and keep animal protein moderate (to reduce urinary calcium and CaP risk):

- Sodium goal: ≤2,000–2,300 mg/day.

- Avoid high animal-protein patterns; keep portions moderate.

3. Sample Menu Suggestions:

Emphasize (hydrating, lower sodium, not intentionally alkalinizing):

- Fluids: water; sparkling water; low-sugar beverages. (Citrus is not automatically forbidden, but avoid high-dose citrate/alkali products without guidance.)

- Meals:

- Breakfast: oatmeal with milk/yogurt; fruit

- Lunch: home-prepared chicken/egg/legume bowl with vegetables; olive oil-based dressing

- Dinner: fish/poultry + rice/potatoes + vegetables; herbs/spices instead of salt

- Calcium: keep normal dietary calcium (~1,000–1,200 mg/day) from food; do not restrict (unless specifically instructed for another condition).

Limit/avoid (CaP risk enhancers):

- “Alkaline” supplements: potassium citrate (unless prescribed), sodium bicarbonate, alkaline water concentrates

- High-sodium foods: deli meats, fast food, salty snacks, instant soups, packaged sauces

- Very high-protein dieting (can increase calcium excretion)

4. Safety Warning:

- Topiramate substantially increases stone risk; dietary steps may not fully offset it. She should discuss with her prescriber whether dose reduction, alternative therapy, or formal stone-prevention pharmacotherapy is appropriate.

- Many topiramate users develop low serum bicarbonate/metabolic acidosis and low urine citrate —treating acidosis can require alkali, but this must be specialist-managed in CaP stone formers to avoid pushing urine pH even higher.

- To refine recommendations, obtain a complete 24h urine (volume, calcium, citrate, sodium) and serum electrolytes/bicarbonate.

**Output (Control):**

Thanks for sharing the details — that helps a lot. I’ll tailor this specifically to **calcium phosphate (CaP) stones**, **urine pH 6.5**, and **topiramate use**, which is an important risk factor.

**Big picture (very important for CaP stones)**

Calcium phosphate stones form **more easily in alkaline urine**.
Topiramate **raises urine pH and lowers citrate**, both of which **increase CaP stone risk**.
So your diet goals are a bit different from calcium oxalate stone prevention.

Your priorities are:

1. **High urine volume**
2. **Lower urinary calcium**
3. **Avoid further alkalinizing urine**
4. **Maintain (not restrict) dietary calcium**
5. **Reduce sodium and animal protein**

**1. Fluids (most important)**

Aim for **≥2.5–3.0 L urine/day** (usually ~3 L fluid intake).

**Best choices**

- Water (still or sparkling)
- Water flavored with a splash of lemon/lime (small amounts are okay)
- Unsweetened tea (moderate amounts)

**Limit / avoid**

- Sugary drinks
- Energy drinks
- Excessive citrus juices (large volumes can raise urine pH further)
- Cola-type sodas (phosphoric acid, dehydration risk)

📌 Spread fluids throughout the day and **drink before bed**.

**2. Calcium intake (do NOT restrict)**

Target: **1,000–1,200 mg/day from food**

**Good sources**

- Milk, yogurt, kefir
- Cheese (moderate portions)
- Calcium-fortified plant milks (check labels)

**Important**

- Get calcium **with meals**
- Avoid calcium supplements unless your doctor specifically recommends them

Low dietary calcium → **higher stone risk**, not lower.

**3. Sodium restriction (critical)**

High sodium → **more calcium in urine**

**Goal:** ≤2,000 mg sodium/day (ideally 1,500 mg)

**Limit**

- Processed foods
- Fast food
- Canned soups
- Chips, salty snacks
- Frozen meals

**Tips**

- Cook at home when possible
- Read labels (sodium adds up fast)
- Use herbs/spices instead of salt

**4. Protein (moderate, not high)**

Excess animal protein increases urinary calcium and lowers citrate.

**Recommended**

- ~0.8–1.0 g/kg/day protein

**Prefer**

- Fish
- Poultry
- Plant proteins (beans, lentils, tofu)

**Limit**

- Red meat
- Processed meats
- Large protein portions

You don’t need to avoid meat entirely — just avoid **high-protein diets**.

**5. Fruits & vegetables (but choose wisely)**

Vegetables are good, but **very alkaline diets can worsen CaP stones**.

**Good choices**

- Berries
- Apples, pears
- Grapes
- Cucumbers
- Zucchini
- Lettuce
- Bell peppers

**Use in moderation**

- Large amounts of citrus
- Very alkaline-heavy diets (e.g., extreme “alkaline diet” plans)

Unlike calcium oxalate stones, **aggressively alkalinizing the urine is not helpful** for you.

**6. Oxalate (less important for CaP stones)**

Oxalate restriction is **not a major focus** for calcium phosphate stones, but avoid extremes:

- Don’t overconsume spinach, rhubarb, beets, almonds
- Normal mixed diet is fine

**7. Citrate: food vs supplements**

Citrate can help bind calcium, **but** in CaP stone formers:

- **Potassium citrate supplements may worsen stones** by raising urine pH further
- Food-based citrate (small amounts of lemon/lime) is usually safer

⚠️ Do **not** start citrate supplements without a stone specialist’s guidance.

**8. Topiramate-specific advice**

Topiramate:

- Raises urine pH
- Lowers citrate
- Increases CaP stone risk

**Important steps**

- Stay very well hydrated
- Avoid alkalinizing supplements
- Ask your clinician if dose reduction or alternatives are possible
- Periodic **24-hour urine testing** is strongly recommended

**Sample day (stone-friendly)**

**Breakfast**

- Greek yogurt with berries
- Whole-grain toast
- Water

**Lunch**

- Grilled chicken or tofu
- Rice or quinoa
- Steamed zucchini + salad
- Water

**Snack**

- Apple or pear
- Cheese stick

**Dinner**

- Fish
- Roasted vegetables
- Small portion of dairy
- Water

**When to follow up medically**

You should ideally have:

- **24-hour urine testing** (calcium, citrate, pH, volume)
- Periodic stone analysis
- Review of topiramate risks/benefits

If you want, I can:

- Build a **weekly meal plan**
- Review a **24-hour urine report**
- Help tailor advice if you’re vegetarian or on a specific diet

Just tell me 👍
