# Supplementary material for: Evaluating a Guideline-Integrated Clinical Interaction Framework Vs a Standard Large Language Model Interaction for Dietary Recommendations in Recurrent Urolithiasis: In Silico Study
Source: J Med Internet Res. 2026 Sep 4;28:e95162. doi: 10.2196/95162 (PMC13544699; doi:10.2196/95162)
Supplement: Supplementary file 3 [file jmir-v28-e95162-s004.docx]

**Full Prompts Structure:**

**StoneAgent + Query A (SA + QA):**

[StoneAgent prompt (Which is in Multimedia Appendix 3)]

Now answer the following patient question:

[Patient_Query_A (Which is in Multimedia Appendix 1)]

**Standard LLM + Query A (Std + QA):**

[Standard LLM prompt (Which is in Multimedia Appendix 3)]

Please answer the following patient question:

[Patient_Query_A (Which is in Multimedia Appendix 1)]

**StoneAgent + Query B (SB + QB):**

[StoneAgent prompt (Which is in Multimedia Appendix 3)]

Now answer the following patient question:

[Patient_Query_B (Which is in Multimedia Appendix 1)]

**Standard LLM + Query A (Std + QB):**

[Standard LLM prompt (Which is in Multimedia Appendix 3)]

Please answer the following patient question:

[Patient_Query_B (Which is in Multimedia Appendix 1)]

**Outputs:**

**Case1:**

**SA + QA:**

Below is your individualized, guideline-based metabolic interpretation and diet plan, written from an Endourology + Renal Dietetics perspective.

1. Metabolic Diagnosis

You have clear hypercalciuria secondary to very high sodium intake (urine sodium 260 mmol/day), with low urine volume.
Because sodium and calcium compete for reabsorption in the renal tubules, excess sodium intake increases urinary calcium excretion—this is the primary modifiable driver of your calcium oxalate stones.

Hypertension strengthens the need for sodium restriction, as this intervention benefits both blood pressure and stone prevention.

2. Key Dietary Modifications (Top Priorities)

① Reduce sodium intake to <2 g sodium/day (≈5 g salt/day).

This typically lowers urinary calcium significantly within weeks.
Practical targets:

- Avoid restaurant meals & packaged foods.
- Choose products with <140 mg sodium per serving (“low sodium”).
- Cook with herbs, citrus, garlic instead of salt.

② Increase fluid intake to achieve ≥2.5 L urine/day.

Your 1.8 L/day is below guideline targets.
Aim for 3 L of total fluids/day (more if sweating).

③ Maintain normal calcium intake (1000–1200 mg/day) from food, not supplements.

Do not restrict calcium — it reduces intestinal oxalate absorption.
Pair calcium-containing foods with oxalate-containing meals.

3. Sample Menu Suggestions

Foods to Prioritize

- Low-sodium proteins: fresh chicken, turkey, fish, eggs, tofu.
- Calcium sources (spread across meals):
  - Milk or lactose-free milk, yogurt, kefir, fortified plant milks, hard cheeses in small amounts.
- High-citrate fruits (natural inhibitors of calcium stones):
  - Lemons, limes, oranges, melon.
- Vegetables & whole grains that are naturally low in sodium.
- Hydration strategy:
  - Water, citrus-infused water, herbal tea, sparkling water (unsalted).

Foods to Limit or Avoid

- High-sodium foods (biggest priority):
  - Restaurant meals, soups, deli meats, bacon, sausage, canned foods, salted snacks, fast food, pickles, sauces (soy sauce, teriyaki, BBQ).
- Excessive animal protein (>1–1.2 g/kg/day), which increases urinary calcium and lowers citrate.
- High-oxalate foods without calcium: spinach, almonds, beets, rhubarb, Swiss chard.
  - These are not banned, but should be eaten with a calcium source.
- Vitamin C supplements >200 mg/day (increase oxalate production).

4. Safety Warning

Because you have hypertension, sodium restriction is strongly beneficial and safe.
No contraindications to higher fluid intake were noted, as long as you do not have heart failure, advanced CKD, or fluid-restricted conditions (let me know if you do).

**Std + QA:**

Below is **general educational guidance**, *not personalized medical advice*. Kidney-stone prevention should be managed with your clinician, especially because you also have **hypertension** and **elevated urinary calcium and sodium**.

That said, your urine profile shows **low urine volume**, **high urine sodium**, and **high urine calcium**—all strong drivers of **calcium oxalate stone recurrence**. Your diet can meaningfully improve all three.

**🚰 1. Increase Fluid Intake (Most Important)**

Your urine volume is **1.8 L/day**, but stone prevention typically requires **≥2.5 L/day** of urine output.

**Target**

- **Drink 3.0 L/day of fluid** (mostly water) to achieve >2.5 L urine output.

**Tips**

- Start the morning with 500 mL water.
- Carry a 1 L bottle and finish at least 2–3 per day.
- Increase fluids with exercise, hot weather.

**🧂 2. Strongly Reduce Sodium Intake**

Your urine sodium is **260 mmol/day** (very high). High sodium intake increases urinary calcium excretion, which worsens stone risk *and* hypertension.

**Target**

- **<2,300 mg sodium/day** (even better 1,500–2,000 mg/day with hypertension).

**How to do this**

- Avoid processed/packaged foods, deli meats, canned soups.
- Choose low-sodium versions of bread, sauces, and snacks.
- Limit restaurant meals (often >2,000 mg sodium in one dish).
- Cook with herbs/acids (lemon/lime/vinegar) instead of salt.

**Expected benefit:** Lowering sodium will drop your urinary calcium significantly.

**🥛 3. Maintain Normal Dietary Calcium**

Low calcium intake **increases** oxalate absorption and stone risk.
Even though your urinary calcium is high, you should **not restrict dietary calcium** unless your clinician instructs otherwise.

**Target**

- **1,000–1,200 mg calcium/day** from foods, ideally with meals.

**Good choices**

- Dairy: milk, yogurt, cheese
- or calcium-fortified alternatives

**Avoid**

- Calcium supplements (unless prescribed), as they can raise urinary calcium.

**🥬 4. Reduce High-Oxalate Foods (But Do Not Go Oxalate-Free)**

Oxalate restriction is useful, but extreme avoidance isn’t necessary if calcium intake is normal.

**Limit (not eliminate)**

- Spinach
- Almonds/almond flour
- Beets
- Rhubarb
- Swiss chard
- Peanuts
- Dark chocolate
- Black tea in large quantities

**Pair oxalate-containing foods with calcium**

This binds oxalate in the gut → less absorption → lower urinary oxalate.

**🥤 5. Increase Citrate Intake**

Citrate helps prevent calcium stone formation.

**Add citrus sources**

- **Lemon or lime juice** (½ cup/day diluted in water)
- Or oranges, grapefruit (if compatible with your medications)

**🍖 6. Moderate Animal Protein**

High animal protein increases calcium, uric acid, and reduces citrate.

**Target**

- Limit to **0.8–1.0 g protein/kg/day** (~60–75 g/day for most men your size).
- Prefer plant-based proteins; reduce large meat portions.

**⚖️ 7. Weight and Blood Pressure Considerations**

- Your BMI of **27** is overweight; gradual weight loss improves metabolic risk and stone recurrence.
- A **DASH-style diet** (fruits, vegetables, low-fat dairy, low sodium) is ideal for hypertension *and* kidney-stone prevention.
- DASH naturally increases potassium and citrate (helpful), but avoid very high-oxalate choices like spinach or beet greens.

**📌 Summary of Dietary Priorities**

**Most impactful changes for your specific urine profile:**

1. **Increase fluids to >3 L/day intake** → goal urine output >2.5 L/day.
2. **Cut sodium drastically (<2,000–2,300 mg/day)** → lowers urine calcium.
3. **Maintain normal calcium intake (1,000–1,200 mg/day)**.
4. **Moderate oxalate and pair with calcium foods**.
5. **Reduce animal protein**.
6. **Use citrate-rich beverages** (lemon water).
7. **Follow a DASH-like dietary pattern** for your hypertension and stone risk.

**SB + QB:**

Below is your personalized, guideline-consistent interpretation and plan.

**1. Metabolic Diagnosis**

**You have dietary-driven hypercalciuria caused primarily by high sodium intake.**

- Your **urine calcium = 9.2 mmol/day** (elevated; normal < 6.2–7.5 mmol/day depending on lab).
- Your **urine sodium = 260 mmol/day** is very high. EAU/CUA guidelines show that for every 100 mmol of sodium consumed, urinary calcium rises significantly.
- **Urine volume = 1.8 L/day** is below the recommended ≥ 2.5 L/day for calcium oxalate stone prevention.
- Because you also have **hypertension**, sodium restriction is doubly important.

➡️ **Cutting dietary calcium is NOT recommended.** Low calcium intake increases intestinal oxalate absorption and *raises* kidney stone risk.

**2. Key Dietary Modifications (Top 3 Priorities)**

**1) Reduce Sodium to < 100 mmol/day (~2 g sodium or 5 g salt/day)**

This is the main driver of your hypercalciuria.

- Reduce processed foods, restaurant meals, soups, deli meats, sauces.

**2) Maintain Normal Calcium Intake (1,000–1,200 mg/day from food)**

Calcium should be consumed **with meals** to bind oxalate.

- Do NOT restrict calcium unless instructed by a specialist for another reason.

**3) Increase Fluid Intake to ≥ 2.5–3 L/day (as tolerated with BP medications)**

Aim for pale-yellow urine all day.

**3. Sample Menu Suggestions**

**Foods to Prioritize**

- **Calcium sources with meals**:
  - Low-sodium yogurt, milk, kefir, fortified plant milks
  - Low-sodium cheese (small portions)
- **Naturally low-sodium whole foods**:
  - Fresh fruits (citrus is especially beneficial for citrate)
  - Vegetables
  - Fresh poultry, fish, lean meats without brine
  - Beans, lentils
  - Whole grains
- **Hydration**:
  - Water, lemon water, herbal tea

**Foods to Limit or Avoid (High Sodium or High Oxalate)**

- **Very high-sodium items**
  - Deli meats, bacon, canned soups, fast food
  - Packaged snacks (chips, crackers, ramen)
  - Soy sauce, bottled dressings, bouillon, pickles
- **High-oxalate foods** *(okay in small amounts only if paired with calcium)*
  - Spinach, almonds, beets, rhubarb, Swiss chard
  - Excessive nuts, nut butters, chocolate, black tea
- **Animal protein excess**
  - Large red-meat portions raise calcium excretion and reduce citrate.

**4. Safety Warning**

Because you have **hypertension**, sodium reduction is essential and entirely safe.
**BUT:**

- If you take diuretics (thiazides, loop diuretics), monitor for dehydration and electrolyte changes when increasing fluids.
- If you have any underlying heart failure (not stated), fluid goals must be individualized.

**Quick Answer to Your Question:**

👉 **Cut salt, NOT calcium.**
Your stone pattern is classic for **salt-driven hypercalciuria**, and maintaining normal calcium actually *protects* you from more calcium oxalate stones.

**Std + QB:**

I can give *general*, non-individualized information about kidney-stone nutrition, but this should **not** replace medical advice from your own clinician, especially because you have high blood pressure and detailed urine findings.

**⭐ Key principles for calcium-oxalate stone prevention (general information)**

**1. Do NOT reduce dietary calcium below normal**

It is a common misunderstanding that calcium should be cut.
In reality:

- **Normal dietary calcium (about 1,000–1,200 mg/day from food)** *reduces* stone risk.
- Calcium in food binds oxalate in the gut → less oxalate absorbed → fewer calcium-oxalate stones.
- **Low-calcium diets actually increase stone risk** and may weaken bones.

So in most people with calcium-oxalate stones, **you should NOT cut dietary calcium**, unless a doctor specifically instructs otherwise.

**2. High urine sodium is strongly linked to high urine calcium**

Your 24-hour urine shows:

- **Urine sodium: 260 mmol/day — very high**
  (Typical stone-prevention target is ~100 mmol/day or less.)

High sodium intake causes the kidneys to excrete more calcium.
More calcium in urine → higher stone risk.

Because you also have high blood pressure, **reducing sodium is beneficial for both stone risk and BP control.**

What this *generally* means:

- Limit salty foods (processed foods, restaurant foods, fast food).
- Aim for **2,000 mg sodium/day or less (≈ 85 mmol)** depending on your clinician’s advice.
- Lowering sodium often lowers urinary calcium, even if your dietary calcium stays normal.

**3. Your urine calcium is elevated**

A urine calcium of **9.2 mmol/day (~370 mg/day)** is higher than typical stone-prevention targets (usually < 6.2 mmol or < 250 mg/day for many adults).
This is consistent with sodium-driven hypercalciuria.

Reducing sodium is the first, safest nutritional step to help lower urine calcium.

**4. Other general dietary measures**

These are standard kidney-stone–prevention recommendations (not personalized):

- **Increase fluid intake** so urine volume ≥ 2.5 L/day.
- **Reduce high-oxalate foods** (spinach, almonds, beets, rhubarb).
- **Moderate animal protein**, which can raise calcium excretion.
- **Eat plenty of fruits and vegetables**, which increase urinary citrate (a stone inhibitor).

**✅ So, cut calcium, salt, or both?**

**General guidance (not personalized medical advice):**

- **Do NOT cut dietary calcium** — keep normal intake from food.
- **Do reduce sodium**, since your urine sodium is very high and you have high blood pressure.
- Sodium reduction will likely help *lower your urine calcium* and reduce stone risk.

**Case2:**

**SA + QA:**

Below is your interpretation and a personalized, guideline-level dietary plan based on **EAU** and **CUA** stone-prevention standards, adapted for your osteopenia.

**1. Metabolic Diagnosis**

Your 24-hour urine shows:

- **Urine Calcium: 8.5 mmol/day** (≈ 340 mg/day) → **Hypercalciuria**
- **Urine Sodium: 120 mmol/day** → **Moderately elevated sodium intake**

**Diagnosis:**
**Hypercalciuria likely driven by high sodium intake (renal leak hypercalciuria), with concurrent osteopenia requiring *adequate* dietary calcium.**

High sodium intake causes increased urinary calcium loss via reduced proximal tubular calcium reabsorption. This worsens stone formation *and* bone mineral loss. Your osteopenia makes sodium reduction a top priority.

**2. Key Dietary Modifications (Top 3 Priorities)**

**1. Reduce Sodium to < 65–80 mmol/day (~1500–2000 mg sodium)**

Lowering sodium directly decreases urinary calcium and protects bone.
**Practical changes:** eliminate salted snacks, packaged soups, deli meats, restaurant meals, and reduce added salt.

**2. Maintain Normal Dietary Calcium: 1000–1200 mg/day (NOT low calcium)**

Adequate calcium binds dietary oxalate in the gut, reducing stone formation, and is essential for osteopenia.
**Timing rule:** consume calcium *with meals* that contain oxalate.

**3. Moderate Animal Protein to ~0.8–1.0 g/kg/day**

Excess animal protein increases calcium excretion, lowers urine citrate, and acidifies urine.
Focus on plant-forward proteins (beans, lentils, tofu), limit large portions of meat.

**3. Sample Menu Suggestions**

**✔️ Foods to Prioritize**

**Calcium-rich, low-sodium options**

- Plain yogurt, Greek yogurt
- Milk or calcium-fortified plant milks
- Sardines with bones (unsalted)
- Tofu set with calcium sulfate
- Low-sodium cheeses (Swiss, fresh mozzarella in moderation)

**Vegetables & fruit that support stone prevention**

- Citrus fruits (lemons, limes, oranges) → boost **citrate**, a natural stone inhibitor
- Bananas, melons, peaches
- Low-oxalate vegetables: broccoli, cauliflower, zucchini, kale, bok choy

**Plant-forward proteins**

- Lentils, chickpeas, black beans
- Tofu, tempeh
- Unsalted nuts *in small amounts* (keep portions modest due to oxalate)

**Hydration pattern**

- Aim for urine output ≥ 2–2.5 L/day
- Spread fluid across the day
- Add lemon juice to water for citrate

**❌ Foods to Limit or Avoid**

**High-sodium foods**

- Fast food, takeout, pizza
- Packaged ramen, canned soups
- Deli meats, bacon, sausage
- Salted nuts, chips
- Restaurant meals (ask for no added salt)

**High-oxalate foods (limit, not eliminate)**

- Spinach, almonds, beets, Swiss chard
- Rhubarb, dark chocolate, sweet potatoes
- Wheat bran

If you eat these occasionally, pair with a **calcium source** (e.g., yogurt) to prevent oxalate absorption.

**Sample Day Menu (Bone + Stone Friendly)**

**Breakfast**

- Plain Greek yogurt + blueberries + chia seeds
- 1 slice whole-grain toast (no salted butter)
- Water with a squeeze of lemon

**Lunch**

- Lentil soup (homemade, low sodium)
- Side salad with kale, cucumbers, olive oil & lemon dressing
- 1 cup calcium-fortified plant milk

**Snack**

- Small handful of unsalted cashews
- Citrus fruit

**Dinner**

- Grilled salmon or tofu
- Steamed broccoli + quinoa
- Sparkling water with lemon

**Evening (if needed)**

- Glass of milk or fortified oat milk

**4. Safety Warning**

- With **osteopenia**, *do not* reduce calcium intake.
- If you take calcium supplements, take them **with meals**, not between meals (reduces stone risk).
- If you have hypertension or are on diuretics, confirm sodium and fluid goals with your clinician.
- If you develop swelling, shortness of breath, or have heart/kidney disease, fluid recommendations may need adjustment.

**Std + QA:**

Below is **evidence-based, practical dietary guidance** tailored to your situation: **recurrent calcium oxalate stones**, **high urine calcium (8.5 mmol/day)**, **high urine sodium (120 mmol/day)**, and **co-existing osteopenia**, where the goal is to **reduce stone recurrence without compromising bone health**.

**⭐ Key Principles for *Your* Case**

You need a diet that:

1. **Lowers urine calcium** (to reduce stone formation).
2. **Maintains adequate calcium intake** (to protect bone density).
3. **Reduces oxalate absorption** (another driver of stone formation).
4. **Supports citrate levels** (natural stone inhibitor).
5. **Manages sodium, protein, and hydration**, which strongly influence stone risk.

**✔️ What You *Should* Eat**

**1. Adequate Dietary Calcium (NOT Restriction!)**

Even though your urine calcium is high, **low calcium intake worsens both stones AND bone health**.
Aim for **1,000–1,200 mg/day** from food.

**Best sources (spread through the day):**

- Dairy: milk, yogurt, kefir, cheese
- Calcium-fortified plant milks
- Sardines or salmon with bones
- Tofu set with calcium
- Leafy greens *with low oxalate* (bok choy, kale—but **not spinach**)

**Why it matters:** Dietary calcium binds oxalate in the gut → **less oxalate absorbed → fewer stones**.

*Important:* Take calcium with meals, especially if oxalate-rich foods are included.

**2. Reduce Sodium Intake**

Your urine sodium (120 mmol/day) is high. Sodium increases urinary calcium.
Aim for **<2 g of sodium/day (~5 g salt)**.

**How to eat low-sodium:**

- Cook at home; avoid restaurant meals & processed foods.
- Choose “unsalted,” “low sodium,” or “no added salt” products.
- Season with herbs, lemon, pepper, garlic—not salt.

**Expected benefit:** Lower sodium → **lower urine calcium** → both bone and kidney protective.

**3. Moderate Animal Protein**

High animal protein increases urine calcium and lowers citrate.

Aim for:

- **0.8–1.0 g protein/kg/day** (generally 50–70 g/day for many women)
- Include more **plant proteins** (lentils, beans, tofu—note oxalate considerations below)

Good choices:

- Fish, poultry in moderate amounts
- Beans/lentils in moderate portions
- Limit: red meat, organ meats

**4. Manage Oxalate Without Over-Restricting**

Oxalate contributes to calcium oxalate stones, but you **do not need a zero-oxalate diet**.

**Avoid or limit:**

- Spinach
- Almonds and nut butters
- Beets
- Rhubarb
- Chocolate in large amounts
- Bran cereals
- Sweet potatoes
- Black tea in excess

**Eat with calcium:**

If you eat oxalate-rich foods occasionally, pair them with **calcium-containing food** (e.g., yogurt, cheese, milk at the same meal).

**5. Boost Citrate Intake (Natural Stone Inhibitor)**

Low citrate increases stone risk. Diet can raise it.

Eat:

- **Citrus fruits:** lemons, limes, oranges
- **Lemon water** (½–1 lemon/day squeezed into water)
- Fruits and vegetables daily (helps alkalinize urine)

**6. Hydration: the Most Powerful Intervention**

Goal: **≥2.5 L urine/day**, often requiring **3 L fluids/day**.

Include:

- Water (primary)
- Citrus-infused water
- Herbal teas
  Avoid:
- Large amounts of sugar-sweetened soda
- Excess cola (phosphoric acid harms bones and stone risk)

**7. Maintain Bone Health While Reducing Stone Risk**

For osteopenia, combine diet + lifestyle:

**Nutrients for bone support:**

- **Calcium:** 1,000–1,200 mg/day (from food)
- **Vitamin D:** 800–2,000 IU/day (as advised by your clinician)
- **Protein:** adequate but not excessive
- **Fruits & vegetables:** support alkaline load → improves calcium balance

**Lifestyle:**

- Weight-bearing exercises
- Resistance training
- Avoid smoking; moderate alcohol

**Calcium supplements:** Generally avoided in stone formers unless necessary—when used, take *with meals*.

**🥗 Example Daily Eating Pattern (Stone-Safe + Bone-Healthy)**

**Breakfast**

- Greek yogurt (calcium)
- Low-oxalate fruit (berries)
- Oatmeal (moderate oxalate but OK with calcium-containing foods)

**Lunch**

- Grilled chicken or tofu
- Kale or bok choy salad
- Brown rice
- Lemon water

**Snack**

- Cheese stick or yogurt
- Fruit

**Dinner**

- Baked salmon
- Roasted vegetables (zucchini, carrots, peppers)
- Small baked potato with skin (moderate oxalate, okay with meal calcium)

**Evening**

- Herbal tea
- Hydration to reach fluid goals

**🧂 Summary of Most Important Changes for *Your* Stone Profile**

| **Issue** | **Your Finding** | **Recommendation** | **Reason** |
| --- | --- | --- | --- |
| High urine calcium | 8.5 mmol/day | **Lower sodium**, moderate animal protein | Reduces calcium excretion |
| Recurrent CaOx stones | — | Adequate calcium, limit high-oxalate foods, hydrate | Lowers supersaturation |
| Osteopenia | — | **Do NOT reduce dietary calcium** | Protects bone |
| High urine sodium | 120 mmol/day | Reduce dietary sodium | Strong driver of urine calcium rise |

**SB + QB:**

Below is your personalized, guideline-based interpretation and plan.

**1. Metabolic Diagnosis**

Your 24-hour urine profile shows **hypercalciuria** (8.5 mmol/day) together with **high urinary sodium** (120 mmol/day).
The most likely mechanism—based on EAU & CUA metabolic stone guidelines—is **sodium-driven (renal) calcium wasting**, not excessive calcium intake.

Because you also have **osteopenia**, *restricting dietary calcium would be unsafe*, as it worsens bone loss and increases oxalate absorption in the gut, promoting more calcium oxalate stones.

**Diagnosis:**
**“Sodium-induced hypercalciuria in a patient with calcium oxalate stones and osteopenia.”**

**2. Key Dietary Modifications (Top Priorities)**

**1. Maintain Normal Dietary Calcium (1,000–1,200 mg/day) With Meals**

- This binds oxalate in the gut → lowers oxalate absorption → lowers CaOx stone risk.
- Also protects bone mineral density.
- Choose **food-based calcium**, not high-dose supplements (unless medically indicated).

**2. Reduce Sodium Intake to < 80–100 mmol/day (~< 2 g sodium or < 5 g salt/day)**

Your sodium is 120 mmol/day—high enough to significantly increase urinary calcium.
Reducing sodium is the **most powerful dietary intervention** to lower your urine calcium.

Practical sodium reduction:

- Avoid processed meats, canned soups, restaurant meals, pickled foods.
- Choose “no-salt-added” versions of foods.
- Stop adding salt at the table; flavor with citrus, herbs, spices.

**3. Moderate Animal Protein (0.8–1.0 g/kg/day)**

High protein increases calcium excretion and decreases citrate (a stone inhibitor).
Shift toward plant-based proteins where possible.

**3. Sample Menu Suggestions**

**Foods to Prefer**

**Calcium sources (with meals):**

- Plain yogurt (¾ cup)
- Milk or calcium-fortified plant milks
- Cheese in small portions
- Sardines or salmon with bones

**Low-sodium meal ideas:**

- Grilled chicken or tofu with lemon, herbs, and olive oil
- Brown rice or quinoa (unsalted)
- Steamed vegetables (no added salt)
- Mixed greens with olive oil + vinegar dressing

**Oxalate-safe fruits & vegetables:**

- Apples, grapes, berries, melon
- Cauliflower, cabbage, peas, zucchini, squash
- Low-oxalate greens like bok choy or kale

**Hydration plan:**

- Aim for urine output ≥ 2.0–2.5 L/day
- Add lemon/lime to water for natural citrate boost

**Foods to Limit or Avoid**

**High-sodium foods:**

- Cured meats (ham, bacon, deli meats)
- Restaurant meals, fast food
- Sauces like soy sauce, teriyaki, bouillon, packaged broths

**Oxalate-dense foods (do not eliminate entirely but limit):**

- Spinach, beet greens, almonds, peanuts
- Rhubarb, beets
- High-oxalate chocolate in large quantities

**High animal protein:**

- Red meat, organ meats
- Excessive poultry or fish portions (>1 serving/meal)

**4. Safety Warning**

Because you have **osteopenia**, **do NOT restrict dietary calcium**. Doing so can:

- Increase intestinal oxalate absorption
- Increase stone formation
- Accelerate bone loss

If you were ever advised to stop calcium intake, that advice does **not** apply based on current EAU/CUA evidence for stone formers with osteopenia.

**Bottom Line**

The **safest** and most effective approach for you is **normal calcium intake + low sodium diet**. This combination reduces urine calcium, protects bones, and lowers calcium oxalate stone risk.

**Std + QB:**

Here’s **evidence-based, safe, and practical dietary guidance** for someone with **recurrent calcium-oxalate stones *and* osteopenia**, based on what is known from nephrology and bone-health guidelines.
*(This is general education, not personal medical care.)*

**⭐ Key Principle**

**Do NOT restrict dietary calcium.**
For calcium-oxalate stone formers, restricting calcium *increases* stone risk and worsens bone health. You actually need **adequate calcium with meals** so calcium can bind oxalate in the gut and reduce absorption.

Your 24-hour urine values show:

- **High urine calcium (8.5 mmol/day ≈ ~340 mg/day)** → increases stone risk
- **High urine sodium (120 mmol/day)** → raises urine calcium

So the goal is **normal calcium intake + lower sodium intake**, not calcium restriction.

**✅ What Diet Pattern Is Safest for You?**

**1. Get enough calcium: ~1,000–1,200 mg/day from food**

This protects bone and reduces stone risk.

Best sources (with meals):

- Dairy: milk, yogurt, cheese
- Calcium-fortified plant milks
- Tofu set with calcium
- Canned fish with bones (sardines, salmon)

**Why this helps:**
Dietary calcium binds oxalate in the intestines → less oxalate enters urine → less stone risk.

**Important:** Avoid relying on calcium supplements unless your clinician advises it, because supplements increase urinary calcium more sharply.

**2. Reduce sodium to ≤ 2 g/day (≈ 80–90 mmol)**

Your sodium intake is likely driving your high urine calcium.

Strategies:

- Avoid salty snacks, fast food, processed meats, canned soups
- Choose foods with **< 300 mg sodium per serving**
- Cook with herbs/acids instead of salt
- Restaurant meals: request “no added salt”

**Effect:** For every 100 mmol of sodium reduced, urinary calcium drops by ~1 mmol—meaning sodium control directly protects your kidneys.

**3. Moderate animal protein**

Excess animal protein increases urine calcium and lowers citrate (a natural stone inhibitor).

Guidelines:

- 0.8–1.0 g protein/kg/day
- Favor plant proteins more often (beans, lentils, nuts in moderation)

**4. Limit high-oxalate foods — but don’t avoid all oxalate**

Focus on the major offenders:

**High oxalate (limit):**

- Spinach, beets, rhubarb
- Almonds, cashews
- Soy nuts
- Bran cereals
- Dark chocolate
- Potato skins
- Black tea (large quantities)

You *can* still eat many vegetables—just pair oxalate-containing foods with calcium at the same meal.

**5. Increase fruits and vegetables for citrate**

Citrate is a protective factor against stones.

Helpful foods:

- Citrus (lemon, lime, orange)
- Melons
- Many vegetables (except those very high in oxalate)

A daily glass of lemon water can meaningfully boost citrate.

**6. Maintain adequate hydration**

Aim for **urine volume ≥ 2–2.5 L/day**.

Tips:

- Spread water through the day
- Extra during exercise or hot weather
- Herbal teas or diluted juices are okay
- Avoid high-sugar beverages

**7. Don’t severely restrict calcium because of osteopenia**

Low calcium intake causes:

- Increased intestinal oxalate absorption → more stones
- Worse bone loss → higher fracture risk

Evidence consistently shows **normal calcium + low sodium** reduces urinary calcium better than restricting calcium.

**🔍 Summary for Your Situation**

**Safest diet approach:**

1. **Calcium 1,000–1,200 mg/day with meals**
2. **Sodium reduction to ≤ 2 g/day**
3. Moderate animal protein
4. Avoid only the *very* high-oxalate foods
5. Increase fruits/vegetables for citrate
6. High fluid intake

This combination lowers urine calcium, improves bone density, and decreases stone recurrence.

**Case3:**

**SA + QA:**

Below is an evidence-based, guideline-aligned interpretation tailored to your values **(citrate 0.8 mmol/day = severe hypocitraturia; pH 6.0 = mildly acidic to neutral)** and stone type (**mixed calcium oxalate + calcium phosphate**) with metabolic syndrome.

**1. Metabolic Diagnosis**

**Severe hypocitraturia with suboptimal urine alkalinization**, likely driven by features of **metabolic syndrome** (insulin resistance → impaired renal citrate handling).
Citrate this low significantly increases stone risk because citrate binds calcium and prevents crystal formation.

Your urine pH of **6.0** is borderline:

- Low for *calcium oxalate* stone prevention (which prefers pH ~6.5).
- Acceptable **but must not be pushed too high** for *calcium phosphate* stone prevention (risk increases when pH > 6.8).

**2. Key Dietary Modifications (Top Priorities)**

**Priority 1 — Increase dietary alkali to raise urine citrate (without overshooting pH).**

Focus on **citrate-rich, potassium-rich fruits & vegetables**, NOT supplements yet.

- Ideal choices: lemons, limes, oranges, melons, leafy greens, zucchini, tomatoes.
- Avoid excessive bicarbonate or strong alkaline waters that push urine pH > 6.8.

**Priority 2 — Reduce animal protein load**

This directly lowers acid production and increases citrate.

- High animal-protein diets (red meat, poultry, eggs) lower urinary citrate and increase stone risk.
- Target: **≤0.8–1.0 g protein/kg/day**, emphasizing **plant-forward protein sources**.

**Priority 3 — Implement sodium restriction (<2 g/day sodium)**

High sodium intake → increased urinary calcium → increased calcium oxalate & calcium phosphate supersaturation.
This is essential for metabolic syndrome patients.

**Secondary but important:**

- Maintain **normal dietary calcium (1000–1200 mg/day)** with meals to reduce oxalate absorption.
- Reduce high-oxalate foods *only if consumed excessively* (spinach, almonds, rhubarb, beets, dark chocolate).
- Weight reduction and low-glycemic dietary pattern improve citrate excretion.

**3. Sample Menu Suggestions**

**Breakfast**

**Good choices:**

- Greek yogurt with blueberries + chia seeds
- Oatmeal with sliced banana and cinnamon
- Water with lemon (freshly squeezed)

**Avoid:**

- Bacon, sausage (↑ sodium, ↑ acid load)
- Large egg-heavy breakfasts daily
- High-sugar pastries (metabolic syndrome worsening)

**Lunch**

**Good choices:**

- Grilled salmon or tofu with quinoa and roasted vegetables
- Mixed greens with chickpeas, tomatoes, cucumber, olive oil + citrus vinaigrette
- Sparkling water with lime

**Avoid:**

- Deli meats, fast food, soups (very high sodium)
- Large portions of chicken/beef (acid load ↓ citrate)

**Dinner**

**Good choices:**

- Lentil or bean stew with vegetables
- Stir-fried vegetables with small portion of chicken or fish
- Baked potato or sweet potato (alkali-rich)

**Avoid:**

- High-protein ketogenic meals
- Large steaks or high-salt marinades
- Cream-based sauces (often high in sodium)

**Snacks**

**Good:** citrus fruit, melon, yogurt, vegetable sticks, nuts (in moderation).
**Avoid:** salty nuts, chips, processed snacks.

**4. Safety Warning**

Because you have **metabolic syndrome**, you may have elements of insulin resistance, hypertension, or early CKD:

- **Increase potassium-rich fruits only if kidney function is normal.** If you have reduced kidney function, high-potassium foods require monitoring.
- Avoid aggressive alkalinizing agents (e.g., potassium citrate supplements) unless monitored, because they can **raise urine pH too high**, increasing **calcium phosphate stone** risk.

If you want, I can create a **personalized week-long menu** or calculate your **exact target protein, sodium, and alkali load** based on your height, weight, and kidney function.

**Std + QA:**

Below is **general educational information** on diet patterns known to help reduce kidney-stone risk in people with **calcium oxalate + calcium phosphate stones**, **low citrate**, **metabolic syndrome**, and **urine pH around 6.0**.
I cannot provide personalized medical advice, but these are evidence-based principles you can discuss with your clinician or dietitian.

**⭐ Key Dietary Priorities for Your Stone Pattern**

**1. Increase Alkali Intake to Raise Citrate**

Your citrate (0.8 mmol/day) is *very* low. Citrate is a natural stone inhibitor. Diet can help raise it:

**Eat More Citrate-Rich, Alkali-Producing Foods**

- **Fruits & vegetables** ≥ **5 servings/day**
  Best options: lemons, limes, oranges, melons, berries, leafy greens.
- **Citrus beverages** can modestly raise citrate
  Lemon water or orange juice (if blood sugar allows).
  For metabolic syndrome, prefer **lemon/lime water without added sugar**.

**Limit High Animal-Protein Load**

Animal protein increases acid production and lowers citrate.
Try:

- Replacing some red meat with fish, legumes, tofu, or plant-based proteins.
- Keeping animal protein to *moderate* portions (~0.8 g/kg/day unless advised otherwise).

**2. Follow a Low-Sodium Diet (≤ 2 g/day sodium)**

High sodium increases urinary calcium and raises stone risk.

**How to do this:**

- Avoid processed foods, fast food, canned soups, deli meats.
- Choose fresh ingredients; read labels targeting **<150 mg/serving** when possible.
- Use herbs, spices, lemon, vinegar for flavor instead of salt.

**3. Eat Normal Dietary Calcium (NOT low calcium)**

For calcium-based stones, reducing dietary calcium *increases* stone formation.

- Aim for **1,000–1,200 mg/day** from food, not supplements.
- Good sources: yogurt, milk, cheese, calcium-fortified plant milks.
- Take calcium **with meals** to bind oxalate from food and prevent absorption.

**4. Reduce Dietary Oxalate (But Not Excessively)**

Since you have calcium oxalate stones, pairing calcium with meals is more important than eliminating oxalate.
Moderate intake of:

- Spinach, almond products, rhubarb, beets, Swiss chard, peanuts, sweet potatoes.
- If you eat these foods, include a calcium source in the same meal.

**5. Manage Carbohydrates & Weight (Metabolic Syndrome)**

Metabolic syndrome increases stone risk—partly through higher acidity and lower citrate.

**Helpful eating patterns:**

- **Mediterranean-style diet**
- **Plant-forward meals**
- **Whole grains instead of refined carbs**

These patterns improve insulin resistance, which can help normalize urine chemistry.

**6. Hydration Target: ≥ 2.5–3.0 L Urine/Day**

This is the most important single action.

Tips:

- Drink water consistently throughout the day.
- Include lemon/lime water for citrate.
- Reduce high-sugar drinks (worsen metabolic syndrome).
- Mildly alkaline mineral waters may help citrate and pH.

**7. Balance Urine pH (6.0)**

A pH of 6.0 is borderline:

- **Calcium phosphate stones form in higher pH (≥6.5)**
- **Calcium oxalate stones form at lower pH**

Your pH is not excessively high, so increasing fruits/vegetables to raise citrate is still beneficial.
Avoid over-alkalinizing through supplements unless supervised.

**🔑 Summary of Most Helpful Dietary Changes**

If we combine your stone type + low citrate + metabolic syndrome:

| **Priority** | **Why it helps** |
| --- | --- |
| **Increase fruits/vegetables, especially citrus** | Boosts urine citrate (your biggest abnormality) |
| **Reduce sodium** | Lowers urine calcium and recurrence risk |
| **Moderate animal protein** | Reduces acid load → increases citrate |
| **Maintain normal calcium intake with meals** | Prevents oxalate absorption |
| **Moderate high-oxalate foods** | Lowers oxalate load |
| **Improve insulin resistance via Mediterranean-style diet** | Corrects biochemical patterns contributing to stones |
| **High fluid intake** | Dilutes stone-forming minerals |

**SB + QB:**

Below is a **personalized, guideline-based dietary plan** tailored to your low urine citrate (0.8 mmol/day), borderline urine pH (6.0), mixed CaOx/CaP stone history, and the presence of **metabolic syndrome**.

**1. Metabolic Diagnosis**

You have **significant hypocitraturia** (low urine citrate), likely driven by **dietary acid load and metabolic syndrome–related insulin resistance**.
Your urine pH of **6.0** is *not too high* (which is good for CaP) but is **too low for citrate excretion**, meaning your kidneys conserve citrate instead of excreting it.

**Working diagnosis:**
➡️ *Hypocitraturia secondary to metabolic syndrome and excess dietary acid load (high animal protein, low fruits/vegetables).*

**2. Key Dietary Modifications (Top 3 Priorities)**

**Priority 1 — Increase Citrate Intake (Alkali-rich foods)**

Goal: Raise urine citrate to >1.8–2.0 mmol/day

- Add **citrus fruits**: 1–2 servings/day (lemons, limes, oranges).
- Use **lemon water**: 60–120 mL (2–4 oz) lemon juice diluted daily.
- Increase **alkali-rich vegetables**: leafy greens, squash, zucchini, broccoli.

**Priority 2 — Reduce Dietary Acid Load**

High animal protein lowers citrate and lowers urine pH.

- Limit **animal protein** to **0.8–1 g/kg/day**.
- Prefer **plant proteins** 2–3 days/week (lentils, beans, tofu).
- Avoid high-protein weight-loss diets (keto, carnivore) — they worsen citrate.

**Priority 3 — Address Metabolic Syndrome (Improves Citrate and pH)**

- Replace refined carbs with **whole grains**.
- Choose **low-glycemic fruits** (berries, apples, oranges).
- Increase **insoluble + soluble fiber** which improves insulin sensitivity and lowers stone risk.

**3. Sample Menu Suggestions**

**Foods & Drinks to ADD**

**Citrate-boosting foods**

- **Lemon or lime water**
- **Citrus fruits** (oranges, mandarins, grapefruit*)
- **Melons** (cantaloupe, honeydew)
  *Avoid grapefruit if taking statins.

**Alkali-rich vegetables**

- Spinach*, kale, Swiss chard
- Broccoli, cauliflower, peas
- Zucchini, cucumbers
  *Spinach is high in oxalate → keep portions small (½ cup cooked max) and pair with calcium foods.

**Calcium-appropriate foods**

(Do **not** restrict calcium; it reduces oxalate absorption)

- Low-fat yogurt or milk with meals
- Calcium-fortified plant milks
- Cheese in moderation

**Metabolic-syndrome friendly choices**

- Whole grains (quinoa, oats, brown rice)
- Beans, lentils, tofu
- Nuts in moderation (¼ cup/day; avoid almonds if sensitive to their oxalate)

**Hydration plan**

- Aim for **2.5–3 L/day**, unless restricted by a doctor
- Best drinks: water, lemon water, herbal teas
- Limit fizzy drinks except **citrate-containing ones** like Crystal Light lemonade.

**Foods & Drinks to AVOID**

**Protein-related acid load**

- Large portions of **red meat**, poultry, or fish (>100–120 g per meal).
- High-protein diets (keto, Atkins).

**High-sodium foods**

Excess sodium = higher urine calcium + lower citrate

- Processed meats (sausages, deli meats)
- Fast food
- Canned soups, instant noodles
- Restaurant meals
- Salty snacks (chips, crackers)

**High-oxalate foods (important for the CaOx component)**

- Almonds, peanuts, cashews
- Spinach (minimize; don’t eliminate)
- Beets, rhubarb
- Dark chocolate, black tea in excess
  Pair any oxalate foods **with calcium** at the same meal.

**Drinks to minimize**

- Cola
- Sugary sodas
- High fructose beverages (promote metabolic syndrome)
- Excess coffee (>3 cups/day) if it replaces water

**4. Safety Warning**

You have **metabolic syndrome**, which increases cardiovascular risk and affects kidney stone chemistry.
If you also have **hypertension, diabetes, fatty liver, or early CKD**, then:

- Avoid extremely high-potassium foods *if you have impaired kidney function*.
- Avoid large fluid increases if you have **heart failure**.

If you want, I can tailor the plan to your exact comorbidities, medications, and full 24-h urine profile.

**Std + QB:**

Below is **general educational guidance** on diet for someone with **mixed calcium oxalate + calcium phosphate stones**, **very low urine citrate (0.8 mmol/day)**, **urine pH 6.0**, and **metabolic syndrome**.
It is **not a substitute for personal medical care**, but it outlines the nutritional principles typically recommended for this combination.

**✅ GOALS for Your Stone Type + Lab Pattern**

1. **Raise urine citrate** (a natural inhibitor of stone formation).
2. **Maintain urine pH in a moderate range** (not too acidic, not too alkaline—pH 6.0 is okay).
3. **Reduce oxalate load** without eliminating healthy foods.
4. **Maintain adequate—but not excessive—calcium intake** with meals.
5. **Improve metabolic syndrome markers** (insulin resistance, weight, triglycerides).

**🍋 Foods & Drinks to *ADD* (Helpful)**

**1. Citrate-Rich Foods to Raise Urinary Citrate**

These help the most for low citrate:

- **Lemon or lime juice** (best source)
  - 2–4 tbsp/day diluted in water (“lemon water therapy”).
- **Orange juice** (also raises citrate—use cautiously if watching sugar).
- **Cantaloupe, melon, mango, papaya**
- **Tomatoes**, **kiwi**, **berries**
- **Citrus fruits in general**

**Tip:**
If weight or sugar control is needed → prioritize **lemon/lime juice in water** over juices.

**2. Calcium WITH Meals (essential for binding oxalate in the gut)**

Aim for **1000–1200 mg/day** from diet, not supplements.

Good options:

- **Yogurt (unsweetened)**
- **Cheese** in moderate amounts
- **Milk** (regular or lactose-free)
- **Calcium-fortified plant milks**
- **Sardines canned with bones**

Importance:
Calcium eaten **with** higher-oxalate foods prevents those oxalates from being absorbed and forming stones.

**3. Fluids — High Volume**

Goal: **≥2.5–3 L/day** total fluid.

Best choices:

- **Water** (primary)
- **Citrus-infused water**
- **Herbal teas**
- **Sugar-free electrolyte water if needed**

Avoid high-sugar drinks because of metabolic syndrome.

**4. Foods to Improve Metabolic Syndrome (and stone risk)**

Insulin resistance increases stone formation by lowering citrate.

Helpful foods:

- **High-fiber vegetables**: leafy greens (but see oxalate note below), broccoli, cauliflower, zucchini, carrots
- **Whole fruits** instead of juice
- **Whole grains (oats, barley, brown rice)**
- **Legumes**
- **Fatty fish rich in omega-3s**
- **Nuts** in small portions (watch oxalate—almonds/walnuts are high)

Choose **Mediterranean-style** dietary patterns.

**🚫 Foods & Drinks to *LIMIT or AVOID***

**1. High-Oxalate Foods (especially without calcium in the meal)**

You don’t need to eliminate them—just moderate and pair with calcium.

Highest oxalate foods to limit:

- **Spinach** (very high)
- **Almonds / almond flour / nut butters**
- **Beets**
- **Swiss chard**
- **Rhubarb**
- **Dark chocolate / cocoa powder**
- **Soybeans / soy products (some are high)**
- **Black tea in excess**

Moderate-oxalate examples:

- Sweet potatoes
- Berries (still fine in moderation)

**2. Excess Sodium (raises calcium in urine → more stones)**

Goal: **<2,000 mg/day**.

Avoid:

- Processed foods
- Restaurant meals with added salt
- Deli meats
- Chips, salted snacks
- Bouillon/soups with high sodium

**3. Excess Animal Protein**

Animal protein → lowers citrate and increases acid load.

Limit:

- Large portions of **red meat**
- **Organ meats**
- High-protein fad diets

Prefer:

- Fish, poultry, plant proteins.

**4. Sugary Foods & Drinks**

These worsen metabolic syndrome and increase stone risk.

Avoid or minimize:

- Soda
- Fruit juice (except small amounts orange juice for citrate)
- Desserts
- Sweetened coffees
- Energy drinks

**5. Vitamin C Supplements (High Dose)**

**Avoid >500 mg/day.**
High vitamin C → converts to oxalate.

**⚖️ Special Note About Calcium Phosphate Component**

Calcium phosphate stones form more easily when urine is too **alkaline (high pH)**.
Your pH is **6.0**, which is *not high*, so adding citrate (lemon) is safe.

**Avoid potassium citrate supplements unless prescribed**, because they may raise pH too much for mixed stones.

**🧂 Putting It All Together — Sample Day**

**Breakfast**

- Plain yogurt with berries
- Oatmeal with chia seeds
- Water with lemon

**Lunch**

- Grilled chicken or tofu
- Salad with mixed greens (not spinach)
- Whole grain bread
- Water or lemon water
  *Add dairy or fortified plant milk if eating moderate-oxalate items.*

**Snack**

- Carrot sticks, apple
- Small handful of nuts (optional)

**Dinner**

- Salmon or lentils
- Brown rice
- Steamed vegetables
- Citrus water

**🧪 Additional Points**

- **Talk with your clinician** about whether potassium citrate therapy is appropriate. It strongly raises citrate but must be balanced against your stone type.
- **Weight loss** improves citrate levels and lowers stone recurrence.
- **Exercise** improves insulin sensitivity and reduces metabolic syndrome burden.

**Case4:**

**SA + QA:**

Below is your individualized metabolic interpretation and dietary strategy, following EAU/CUA evidence-based guidelines.

**1. Metabolic Diagnosis**

Your only abnormality is **mildly elevated urinary oxalate (0.9 mmol/day ≈ 80 mg/day)** with **normal urine calcium**.
This is consistent with **enteric-independent hyperoxaluria**, typically driven by **dietary oxalate excess**, **low calcium intake with meals**, or **high vitamin C intake**.

**2. Key Dietary Modifications (Top Priorities)**

**1. Reduce high-oxalate foods — not all oxalate foods, just the top offenders.**

Target the highest contributors: spinach, almonds, beets, rhubarb, Swiss chard, cashews, peanuts, sweet potatoes, and dark chocolate.

**2. Ensure *adequate calcium with meals* (1000–1200 mg/day from food).**

Do **not** restrict calcium. Eating normal calcium **together with oxalate-containing foods** binds oxalate in the gut and prevents absorption.

**3. Avoid excess vitamin C (>500 mg/day).**

Vitamin C converts to oxalate in the urine, significantly increasing levels.

**3. Sample Menu Suggestions**

**Foods to Emphasize**

- **Calcium sources with meals:**
  - Yogurt, milk, kefir, cheese
  - Calcium-fortified plant milks
- **Low-oxalate vegetables:**
  - Broccoli, cauliflower, cucumbers, zucchini, mushrooms, lettuce, cabbage
- **Low-oxalate fruits:**
  - Bananas, melons, mango, papaya, apples
- **Proteins:**
  - Poultry, fish, eggs, tofu
- **Carbohydrates:**
  - White rice, quinoa, barley, oats

**Foods to Limit or Avoid**

- **Very high-oxalate items (should be minimized):**
  - Spinach (highest), Swiss chard, beet greens
  - Almonds/almond flour, peanuts, cashews
  - Rhubarb, beets
  - Dark chocolate, cocoa powder
  - Sweet potatoes
- **Moderate-oxalate foods (limit portions but don't eliminate):**
  - Berries, oranges, black tea, wheat bran
  - Beans, lentils (rotate with lower-oxalate alternatives)

**Example Daily Menu**

**Breakfast:**

- Oatmeal with milk and sliced bananas
- Scrambled eggs
- Coffee or tea (limit tea to 1 cup if black)

**Lunch:**

- Grilled chicken bowl with white rice, zucchini, cucumber, and yogurt on the side
- Apple

**Snack:**

- Low-fat yogurt or cheese stick (paired with any moderate-oxalate snack)

**Dinner:**

- Baked salmon
- Steamed broccoli and mushrooms
- Quinoa

**4. Safety Warning**

You reported **no comorbidities**, so standard fluid goals apply:

- Aim for **2.5–3.0 liters of fluid/day** unless a doctor has told you otherwise.
- Avoid high-dose vitamin C supplements (>500 mg/day).

**Std + QA:**

Here’s evidence-based, practical guidance for **reducing oxalate-related calcium oxalate stone risk**, tailored to your situation:

**✅ Key Takeaways for *Your* Case**

- **Your urine oxalate (0.9 mmol/day ≈ ~80 mg/day)** is **above the typical target range** (<40–45 mg/day), so lowering dietary oxalate is appropriate.
- **Your urine calcium is normal**, so the focus should be on *reducing oxalate absorption*, not restricting calcium.

**🍽 What to Change in Your Diet**

**1. Reduce High-Oxalate Foods (but don’t eliminate everything)**

The biggest contributors to urinary oxalate are certain plant foods. You do **not** need a very low-oxalate diet—just avoid the major offenders eaten frequently.

**🚫 Foods to *limit or avoid* (highest impact)**

- Spinach (the #1 contributor)
- Beets
- Swiss chard
- Rhubarb
- Almonds & nut butters (including almond milk)
- Cashews
- Peanuts
- Soy products (tofu, soy milk)
- Dark chocolate / cocoa powder
- Wheat bran

Eating these occasionally is fine, but **daily consumption increases risk**.

**2. Keep Normal Dietary Calcium (don’t restrict!)**

This is one of the most important steps.

- Aim for **1,000–1,200 mg/day** of dietary calcium (prefer foods over supplements).
- Eat calcium **with** meals that contain oxalate.

**👍 Calcium-rich foods:**

- Dairy: milk, yogurt, cheese
- Calcium-fortified plant milks (non–high-oxalate ones like oat or rice milk)

**Why:** calcium binds oxalate in the gut → reduces absorption → lowers urine oxalate.

**3. Increase Fluid Intake**

- Target **2.5–3 liters of urine/day**, which usually means **3–3.5 L of fluid/day**.
- Water is best.
- Citrate-containing drinks (lemon water, lime water) can add benefit.

**4. Limit Vitamin C Supplements**

- Avoid >500 mg/day supplemental vitamin C, which increases oxalate production.
- Normal dietary vitamin C is safe.

**5. Moderate Animal Protein & Sodium**

Even though your calcium is normal, these can affect future risk.

- **Protein:** keep to ~0.8–1 g/kg/day unless you lift heavily.
- **Sodium:** limit to **<2,300 mg/day** (≤1 tsp salt) to prevent future changes in urine calcium.

**6. Choose Low-Oxalate Alternatives**

If you currently eat something high-oxalate, swap rather than restrict unnecessarily.

**⬇️ Low-oxalate greens:**

- Kale
- Bok choy
- Romaine
- Arugula

**⬇️ Low-oxalate nuts/seeds:**

- Pistachios
- Macadamia nuts
- Pecans

**⬇️ Safer grains:**

- White rice
- Corn tortillas
- Quinoa (moderate; ok in normal portions)

**7. Probiotics or gut strategies?**

In people with *normal* gut function, probiotics have **inconsistent** benefit. No need to prioritize them unless advised by a clinician.

**📊 The Practical Version (Do This)**

**Daily:**

- Drink enough to produce pale urine.
- Eat normal calcium with meals (~3 servings/day).
- Avoid spinach, almonds, and rhubarb.

**Weekly:**

- Keep high-oxalate foods to rare treats.
- Limit salty restaurant meals.

**Always avoid:**

- Vitamin C supplements >500 mg/day.

**🔍 Based on your 24-hour urine:**

Your elevated oxalate is likely **diet-driven** rather than metabolic. You probably don’t need extreme restriction—just **target the big contributors and improve calcium pairing**.

**SB + QB:**

Below is your personalized, guideline-consistent interpretation and plan.

**1. Metabolic Diagnosis**

You have **isolated hyperoxaluria** (urine oxalate 0.9 mmol/day ≈ 80 mg/day), with **normal urinary calcium**.
This pattern is most often **dietary hyperoxaluria**, meaning your oxalate intake or absorption is too high—commonly from foods like nuts, spinach, beets, rhubarb, excessive almond products, and high-dose vitamin C.

Calcium is normal, which means your intestines may be **absorbing too much free oxalate** from food—especially if high-oxalate foods are eaten **without calcium at the same meal**.

**2. Key Dietary Modifications (Top Priorities)**

**1. Pair high-oxalate foods with calcium at meals (never eliminate calcium).**

- Calcium binds oxalate in the gut → prevents absorption → lowers urine oxalate.
- Goal: **300–400 mg calcium per meal** when eating moderate oxalate foods.
  (E.g., dairy, fortified plant milks, yogurt, cheese.)

**2. Reduce *very* high-oxalate foods.**

The worst offenders for hyperoxaluria include:

- Spinach, almonds, cashews, peanuts, beets, rhubarb, Swiss chard
- Almond flour products (almond milk, almond butter, “healthy” nut-based snacks)
- Dark chocolate, black tea in large quantities

You don’t need to remove all nuts/greens, just avoid the highest-oxalate ones.

**3. Increase citrate intake (natural inhibitor of stones).**

- Lemon/lime water, citrus fruits, potassium-rich vegetables (if no contraindications).
- Citrate reduces calcium oxalate crystal formation.

**3. Sample Menu Suggestions**

**Good Choices (Oxalate-Smart Meals)**

- **Breakfast:**
  Greek yogurt with berries + small handful of *low-oxalate* nuts (walnuts, pistachios).
- **Lunch:**
  Grilled chicken salad with **kale or romaine** (NOT spinach). Add cheese or yogurt dressing for calcium.
- **Snack:**
  Cottage cheese + fruit, or carrot sticks with hummus.
- **Dinner:**
  Salmon, roasted zucchini, cauliflower mash, side of fortified oat milk.
- **Drinks:**
  Lemon water, plain water, herbal teas (peppermint, chamomile).

**Foods to Limit or Modify**

- Spinach (highest of all greens) → **replace with kale, bok choy, arugula**
- Almonds/almond milk → **choose dairy or calcium-fortified alternatives**
- Cashews, peanuts, nut butters → **limit to small quantities**
- Dark chocolate → small amounts only, after meals containing calcium
- Beetroot → avoid frequent servings
- Sweet potatoes → moderate intake with calcium-containing foods

**Smart Food Pairings**

- If you eat **nuts**, combine them with **yogurt**.
- If you eat **leafy greens**, choose **low-oxalate greens** or add **cheese**.
- If you drink **tea**, add milk and limit large-volume black tea.

**4. Safety Warning**

You have not reported CKD, heart failure, or potassium restrictions.
If any of these apply, **do not significantly increase potassium-rich citrus intake** without clinician guidance.

**Std + QB:**

Here’s practical, **evidence-based dietary guidance for preventing recurrence of calcium oxalate stones** when *urine oxalate is high but urine calcium is normal*.
(*This is general education—not a medical diagnosis or personalized medical care.*)

**⭐ Key Principle**

**You do NOT need to avoid all oxalate-rich foods.**
Instead, the goal is to **reduce very high-oxalate items** and **always pair moderate-oxalate foods with calcium at meals**, which helps oxalate bind in the gut so it is not absorbed.

**1. 🍽️ What to Reduce or Avoid (Highest-Oxalate Foods)**

These foods strongly raise urine oxalate and are the first to scale back:

**Strongly limit (or avoid if intake is high):**

- **Spinach** (one of the *highest* sources)
- **Almonds** and almond products (almond butter, almond flour, almond milk)
- **Cashews, peanuts**
- **Beets**, beet greens
- **Rhubarb**
- **Swiss chard**
- **Dark chocolate / cacao**
- **Black tea** (very strong brews)
- **Wheat bran**
- **Soy products** (soy nuts, tofu—moderation is key)

Even a single large spinach salad or frequent almonds can dramatically increase urinary oxalate.

**2. 👍 Foods You *Can Keep* With Adjustments**

These foods are healthy but moderately high in oxalate.
You don’t need to eliminate them; instead **eat them with calcium**:

- **Leafy greens other than spinach/chard** (e.g., kale, bok choy, romaine)
- **Most nuts**, except almond/cashew/peanut (e.g., pistachios, walnuts)
  → still use moderation (¼ cup portions)
- **Sweet potatoes**, **berries**, **whole grains**
- **Herbs**, **spices**, cocoa in small amounts

**3. 🧀 Combine Oxalate Foods With Calcium (Crucial!)**

Calcium in the gut binds oxalate → prevents absorption → lowers urine oxalate.

**Goal: ~ 300–400 mg calcium *with each meal that contains oxalate***

(equivalent to ~1 cup milk or yogurt, or a calcium-rich non-dairy equivalent)

**Examples of good pairing:**

- Nuts + **yogurt**
- Leafy greens + **cheese** or **calcium-fortified alternatives**
- Whole-grain toast + **calcium-fortified soy milk**
- Chocolate in small amounts + **glass of milk**

👉 If you avoid dairy, use *calcium-fortified* plant milks or supplements **taken with meals**, not separately.

**4. 💧 Hydration: The Most Important Habit**

Aim for **2.5–3 liters of fluid/day**, enough for **2–2.5 L of urine**.
Any fluid counts except sugary sodas. Citrus drinks (lemon water, lime water) add helpful citrate.

**5. 🧂 Keep Sodium Moderate**

High salt intake increases calcium in urine, even if your baseline is normal.

- Limit sodium to **≤2,300 mg/day** (or lower if advised)
- Avoid heavily salted nuts, chips, cured meats, restaurant meals

**6. 🍗 Protein in Moderation**

High animal protein → lower urine citrate + more stone risk.

- Keep portions moderate (85–100 g per meal)
- More plant protein is okay *as long as it’s not soy-heavy or nut-heavy*

**7. If You Want to Keep Eating Nuts & Leafy Greens**

You can! Just avoid the worst offenders and pair correctly.

**Better nut choices (lower oxalate):**

- Pistachios
- Macadamias
- Pecans
- Walnuts

**Better leafy greens (low/medium oxalate):**

- Kale
- Arugula
- Romaine
- Bok choy

Eat these with calcium at meals.

**8. When to Consider Further Work-Up**

If high urine oxalate persists after diet changes, clinicians may check for:

- Vitamin C overuse (>500–1000 mg/day increases oxalate)
- Fat malabsorption issues (rare)
- Very low calcium intake

If you want, I can help interpret your full 24-hour urine values and suggest a plan based on them.

**✔️ Summary: What You Should Change**

Because you said you eat *a lot of nuts and leafy greens* and your oxalate is high:

**Reduce:**

- Spinach
- Almonds & almond products
- Cashews, peanuts
- Beets, rhubarb, Swiss chard
- Large amounts of dark chocolate or black tea

**Keep in moderation—but always with calcium:**

- Other leafy greens
- Other nuts
- Sweet potatoes, berries, whole grains

**Add:**

- 300–400 mg calcium *per meal* containing oxalate
- ≥2.5 L/day fluid
- Lower-sodium diet

**Case5:**

**SA + QA:**

Below is your structured, guideline-based response.

**1. Metabolic Diagnosis**

You have **low urine pH (5.1)** with **elevated uric acid excretion (5.8 mmol/day)**, consistent with **uric acid stone disease driven primarily by acidic urine**, compounded by **hyperuricosuria related to high purine intake and gout**.

For uric acid stones, **urine pH is the dominant factor**—stones form when pH < 5.5.

**2. Key Dietary Modifications (Top Priorities)**

**1. Raise Urine pH through Alkali-Rich Foods**

- Target urine pH **6.0–6.5** through diet.
- Prioritize fruits/vegetables high in natural potassium alkali (unless contraindicated medically):
  **citrus fruits, melons, bananas, leafy greens, potatoes**.

**2. Reduce Dietary Purines (to lower uric acid production)**

- Limit: **red meat, organ meats, shellfish, anchovies, sardines, beer**.
- Prioritize: **plant-based proteins**, **low-fat dairy**, **eggs**, **white meat poultry**, **legumes** (in moderation).

**3. Increase Fluid Intake**

- Goal: **≥2.5 L urine output/day**.
  Citrus-containing beverages (e.g., lemonade) help alkalinize.

**3. Sample Menu Suggestions**

**Foods to Prioritize**

- **Breakfast:**
  Oatmeal with berries + low-fat yogurt; water with lemon.
- **Lunch:**
  Grilled chicken breast, quinoa salad with spinach, cucumber, olive oil; unsweetened citrus water.
- **Dinner:**
  Baked salmon (moderate portion), roasted potatoes, steamed broccoli.
- **Snacks:**
  Fresh fruit (banana, melon), nuts (small handful), vegetables with hummus.

**Foods to Limit or Avoid**

- **High-purine proteins:** beef, lamb, pork, liver, kidney, shellfish, sardines, anchovies.
- **Alcohol—especially beer** (raises uric acid and lowers urine pH).
- **Sugary beverages & fructose-heavy foods** (increase uric acid).
- **Large animal-protein portions** (acid load lowers urine pH).

**Helpful Substitutions**

| **Instead of…** | **Choose…** |
| --- | --- |
| Beef or lamb | Poultry, tofu, or lentils |
| Beer | Water with lemon/lime or herbal teas |
| Organ meats | Egg-based dishes |
| White rice | Quinoa or whole grains |

**4. Safety Warning**

Because you have **gout**, monitor potassium-rich foods if you take **ACE inhibitors, ARBs, or potassium-sparing diuretics**, as these can increase serum potassium.
If you have **CKD**, avoid excessive alkali-rich foods without supervision—urine alkalinization must be individualized.

**Std + QA:**

Below is **general educational guidance** for diet changes that commonly help reduce **uric-acid stone recurrence**. It is **not personal medical advice**—your clinician/dietitian can tailor this to your full medical history, medications (e.g., allopurinol), and lab trends.

**⭐ Why your results matter**

- **Uric acid stones form in acidic urine**.
  Your urine pH of **5.1** is strongly acid-forming (goal is generally **pH 6.0–6.5** to dissolve or prevent stones).
- **Uric acid excretion of ~5.8 mmol/day** is on the higher side, especially in someone with gout, meaning reducing dietary purines helps.
- Diet can meaningfully raise urine pH and lower uric-acid load.

**🥗 Dietary changes that help prevent uric-acid stones**

**1. Increase foods that alkalinize the urine**

These naturally raise urine pH (toward 6–6.5).

**Eat more of:**

- Fruits: citrus (lemons, oranges, grapefruit), melon, bananas
- Vegetables: leafy greens, squash, potatoes, tomatoes, cauliflower
- Legumes in moderate amounts
- Plant-forward meals overall

**Why:** Plant-rich diets generate alkaline metabolites, helping shift pH upward.

**2. Limit high-purine foods (to reduce uric acid production)**

Avoid or sharply limit:

**Very high purine sources**

- Organ meats (liver, kidney, sweetbreads)
- Certain fish/seafood: anchovies, sardines, mackerel, herring
- Game meats

**Moderate purine foods (limit portions)**

- Beef, pork, lamb
- Shellfish
- Poultry
- Some legumes (lentils, peas)—can still be eaten but moderate the amount

**Better choices (low purine):**

- Eggs
- Nearly all vegetables
- Low-fat dairy
- Whole grains
- Most fruits

**3. Reduce sugar-sweetened beverages, especially fructose**

- Soft drinks, sweetened teas, energy drinks increase uric acid and stone risk.
- Choose **water**, sparkling water, or unsweetened drinks instead.

**4. Optimize protein intake**

- Too much **animal protein** lowers urine pH and increases uric acid.
- Aim for **moderate protein**, with **more plant protein** when possible.
- If you eat meat: keep portions small (3–4 oz), choose lean poultry or fish, and limit red meat.

**5. Hydrate to dilute the urine**

- Goal: **≥2–2.5 liters of urine/day**, often requiring 2.5–3 liters of total fluid intake.
- Extra helpful: add **citrate-rich fluids** (lemon water), which support alkalinization.

**6. Limit alcohol—especially beer**

- Beer is high in purines.
- All alcohol increases uric acid production and gout risk.

**7. Weight management (if applicable)**

- Insulin resistance and metabolic syndrome lower urine pH.
- Slow, steady weight control improves stone and gout risk.

**🍋 Example day of stone-preventive eating**

**Breakfast:** Oatmeal with berries, low-fat yogurt, water with lemon
**Lunch:** Vegetable and bean soup, whole-grain bread, salad
**Dinner:** Grilled chicken or plant-based protein, roasted vegetables, baked potato
**Snacks:** Fruit, nuts (small amounts)
**Fluids:** Water, lemon water, unsweetened tea

**✔ Additional notes (important)**

- Diet can help but **alkalinizing medication** (e.g., potassium citrate) is often necessary to reach the target urine pH for uric-acid stone prevention.
- Ask your clinician before increasing potassium-rich foods if you take ACE inhibitors, ARBs, or have kidney disease.
- Repeat 24-hour urine tests confirm whether diet changes are working.

**SB + QB:**

Below is your tailored, guideline-based uric-acid–stone prevention plan, structured exactly as requested.

**1. Metabolic Diagnosis**

The patient has **uric acid urolithiasis driven by persistently low urine pH (5.1) and hyperuricosuria (5.8 mmol/day)**, consistent with **acidic metabolic milieu commonly seen in gout**.
Low urine pH—not high uric acid alone—is the *primary* driver of uric acid crystallization.

**2. Key Dietary Modifications (Top Priorities)**

**Priority 1 — Raise Urine pH through Alkaline Diet Patterns**

- Focus on **high-alkali fruits and vegetables** to shift systemic acid load.
- Emphasize **citrate-rich foods** (citrus fruits), which both alkalinize urine and raise urinary citrate.

**Priority 2 — Reduce Purine Load to Lower Uric Acid Production**

- Limit **red meat, organ meats, shellfish**, and **yeast-based foods** (e.g., brewer’s yeast, some supplements).
- Prefer plant-based proteins and low-fat dairy.

**Priority 3 — Reduce Fructose and Sugar-Sweetened Beverage Intake**

- Fructose increases endogenous uric acid production.
- Avoid soft drinks, energy drinks, fruit juices with added sugar, and excessive honey or agave.

**3. Sample Menu Suggestions**

**Foods & Drinks to *Focus On* (Alkalinizing & Low-Purine)**

**Proteins**

- Skinless poultry in moderation
- Plant proteins: lentils, beans, tofu, edamame
- Low-fat dairy: yogurt, milk, kefir (dairy **reduces** uric acid)

**Fruits**

- Citrus: lemons, limes, oranges (excellent for alkalinization)
- Bananas, melons, berries, apples

**Vegetables**

- All except spinach in excess; emphasize leafy greens, squash, bell peppers, cucumbers

**Hydration**

- Goal: ≥2.0–2.5 L/day **unless restricted**
- Best options: water, sparkling water, herbal teas
- **Add lemon/lime** to water to increase citrate intake

**Carbohydrates**

- Whole grains: oats, barley, brown rice
- Potatoes and sweet potatoes

**Foods & Drinks to *Limit/Avoid***

**High-Purine Animal Protein**

- Red meat (beef, lamb, pork)
- Organ meats (liver, kidney, heart)
- Shellfish (shrimp, mussels, sardines)

**Fructose-Containing Items**

- Sodas, sweet teas, flavored sports drinks
- Fruit juices with added sugar
- High-fructose fruits *in large quantities* (e.g., mango, grapes)

**Alcohol**

- Beer: highest uric acid impact
- Spirits: moderate impact
- Wine: lower but still not ideal during active stone/gout episodes

**Acidifying Diet Patterns**

- Ketogenic or high-animal-protein diets → strongly acidifying
- Excess sodium → increases renal uric acid handling and worsens gout

**4. Safety Warning**

No specific comorbidities were provided beyond gout.
**Important considerations:**

- If you have **CKD, heart failure, or are on potassium-sparing medications**, avoid aggressive potassium-based alkalinization (e.g., potassium citrate supplements) without medical supervision.
- Very high fluid intake may not be appropriate for patients with **cardiac or renal limitations**.
- If on **allopurinol or febuxostat**, dietary purine restriction remains important but medication dose adjustments should be coordinated with your physician.

**Std + QB:**

Below is **general, educational information** about nutrition patterns associated with lower uric-acid stone risk. It is **not a substitute for personal medical advice**, diagnosis, or treatment. Always review changes with your clinician—especially since you have gout and recurrent stones.

**⭐ Nutrition & Eating Habits to Reduce Uric-Acid Stone Recurrence**

Your situation—**recurrent uric-acid stones**, **gout**, **high uric-acid excretion**, and **very acidic urine (pH 5.1)**—means the most helpful strategies are those that:

1. **Alkalinize the urine** (raise pH into the 6.0–6.5 range)
2. **Reduce uric-acid production**
3. **Support adequate hydration**

Below is a focused breakdown of foods, drinks, and habits that support these goals.

**🍋 1. Prioritize Foods That Help Raise Urine pH (More Alkaline)**

These foods supply organic anions and minerals that lead to more alkaline urine.

**Eat more of:**

- **Fruits**: citrus (lemon, lime, orange), melon, mango, banana, berries
- **Vegetables**: leafy greens, broccoli, cauliflower, squash, tomatoes, potatoes
- **Legumes**: lentils, beans (in moderation if they trigger gout—varies between people)
- **Plant-based proteins** (to replace some animal protein): tofu, tempeh, edamame
- **Nuts** (especially almonds) and seeds

**Why this helps**

Low urine pH is usually the *main driver* of uric-acid stone formation. Diets rich in fruits and vegetables **raise urine pH naturally**, complementing medications such as potassium citrate (if prescribed).

**💧 2. Hydration Strategies to Lower Stone Risk**

- **Goal: at least 2–2.5 liters of urine per day** (for most people ≈ 2.5–3 L of fluid intake).
- Spread fluid intake **throughout the day** and include a glass before bed.
- Use mostly **water**; add:
  - **Citrate-containing beverages** (lemon water, lime water)
  - **Low-sugar flavored waters** if needed for adherence

**Drinks to Limit**

- **Sugary beverages** (raise uric acid)
- **High–fructose drinks** (sodas, energy drinks): fructose strongly increases uric-acid production
- **Alcohol** (especially beer and spirits): increases uric acid and dehydration

**🍗 3. Reduce High–Purine Animal Proteins (Key for Both Gout & Stones)**

**Limit**, don’t eliminate, the following:

**Highest purine sources (most important to reduce):**

- **Red meat** (beef, lamb, pork)
- **Organ meats** (liver, kidney, sweetbreads)
- **Certain fish & seafood**: anchovies, sardines, herring, mackerel, mussels, scallops
- **Game meats**

**Moderate-purine animal proteins (keep portions small):**

- Poultry (chicken/turkey)
- Moderate-purine fish (salmon, tilapia, cod)

**Healthier replacements:**

- Plant proteins (tofu, beans, lentils—individual tolerance varies for gout)
- Low-fat dairy products (milk, yogurt, cheese): **associated with *lower* uric-acid levels**

**🍞 4. Choose Carbohydrates That Do NOT Raise Uric Acid**

**Prefer:**

- Whole grains (oats, barley, brown rice)
- Potatoes, sweet potatoes
- Vegetables and fruit for carbs

**Avoid or minimize:**

- Foods with **high-fructose corn syrup**
- Large amounts of sweets or desserts
- “Energy drinks” or “vitamin waters” containing fructose

**🧂 5. Lower Sodium Intake**

High sodium increases calcium excretion and may worsen stone risk.

- Aim for **<2,300 mg/day** (or lower if advised for blood pressure/gout)
- Limit processed foods, fast foods, snacks, and restaurant meals

**☕ 6. Coffee & Tea: Usually Fine**

Moderate coffee and tea intake is generally neutral or beneficial for gout and stones **as long as**:

- You stay well hydrated overall
- You avoid high-sugar additions

**🥤 7. Best & Worst Drinks for Uric-Acid Stones**

**Best**

- Water
- Lemon/lime water (natural citrate)
- Herbal teas
- Low-fat milk

**Avoid / Limit**

- Soda (especially **cola** and **fruit-flavored high-fructose sodas**)
- Alcohol (beer > spirits > wine in terms of uric acid impact)
- Sweet teas & energy drinks

**🥗 8. Example Day of Eating (Stone- & Gout-Friendly)**

**Breakfast**

- Oatmeal with berries
- Low-fat yogurt
- Water with lemon

**Lunch**

- Large salad with colorful vegetables
- Grilled salmon (small portion) or tofu
- Whole-grain bread
- Herbal tea

**Snack**

- Fruit
- Handful of nuts

**Dinner**

- Stir-fried vegetables
- Brown rice or quinoa
- Small portion of poultry or plant protein
- Water or citrus water

**📌 9. Eating Habits to Prioritize**

- **Consistent hydration**, not just drinking large amounts at once
- **Regular meals** to avoid metabolic spikes
- **Reduce portion sizes** of animal protein (target 4–6 oz/day total if possible)
- **Baseline fruit and vegetable intake:** aim for **5+ servings/day**
- If overweight, **gradual weight loss** helps lower uric acid but avoid crash dieting (which increases uric acid)

**🔑 Key Takeaways**

- Raising urine pH is *the single most effective* dietary strategy for preventing uric-acid stones.
- High-fructose drinks, sugary foods, and heavy animal-protein intake worsen your stone and gout risk.
- Hydration + alkalinizing diet + reduced purine load forms the strongest prevention approach.

**Case6:**

**SA + QA:**

Below is your tailored, guideline-based hydration plan as an Academic Endourologist/Renal Dietitian.

Because you did **not yet provide full 24-hour urine data**, I will answer specifically for the problem you *did* report: **very low urine volume (0.9 L/day)** and **high calcium oxalate supersaturation**, which is one of the strongest predictors of recurrent stones.

**1. Metabolic Diagnosis**

**Severe low urine volume (<1.5 L/day) causing high supersaturation of calcium oxalate.**
This is one of the most powerful and modifiable risk factors for recurrent stones. Even if all other urine parameters were normal, a urine volume of 0.9 L/day alone can drive stone formation.

**2. Key Dietary (Hydration) Modifications**

**Priority 1 — Increase fluid intake to produce ≥2.0–2.5 L urine/day**

EAU & CUA guidelines strongly recommend targeting a **minimum of 2–2.5 L of urine output per day**, which usually requires **2.5–3.0 L of fluid intake/day**.

**Priority 2 — Distribute fluids throughout the day**

Stone formers often drink enough total volume but *inconsistently*. Aim for:

- **500–750 mL morning**
- **500–750 mL afternoon**
- **500–750 mL evening**
- **250–400 mL before bed** *only if you do not have nocturia or heart failure.*

**Priority 3 — Choose fluids that reduce stone risk**

Some drinks lower calcium oxalate supersaturation; others increase it.

**3. Sample Menu (What to Drink & What to Avoid)**

**Best Choices (Drink Daily)**

| **Fluid** | **Benefit** |
| --- | --- |
| **Water (plain or lightly infused)** | Safest and most effective way to dilute urine. |
| **Citrus water (lemon, lime)** | Provides citrate → binds calcium → lowers CaOx supersaturation. |
| **Unsweetened herbal tea** | Hydrating, low oxalate if avoiding black tea. |
| **Coconut water (½ cup/day)** | Mildly increases urinary citrate. |
| **Sparkling water** | OK as long as sodium content is low. |

**Daily example plan (to reach ~2.7–3 L intake):**

- Morning: 600 mL warm water + lemon
- Midday: 750 mL water or herbal tea
- Afternoon: 600 mL infused water (mint/cucumber)
- Evening: 500 mL water
- Pre-bed (optional): 200–300 mL water

**Drinks to Limit**

| **Fluid** | **Why to Limit** |
| --- | --- |
| **Cola (especially dark colas)** | High phosphoric acid → increases stone risk. |
| **Sweetened beverages** | Sugar increases urinary calcium. |
| **Black tea** | Moderate oxalate → acceptable in small amounts, but avoid >1–2 cups/day. |
| **Grapefruit juice** | Associated with increased stone risk in cohort studies. |

**Drinks to Avoid (or use very rarely)**

- **High-sodium mineral waters** (e.g., >200 mg sodium/L) → ↑ urinary calcium
- **Energy drinks** → high sugar + caffeine
- **Heavy alcohol intake** → dehydration

**4. Safety Warning**

If you have **heart failure, chronic kidney disease, or are on fluid-restricted instructions**, do **NOT** increase fluids without clearance from your physician.
If you experience nocturia, distribute fluids earlier in the day.

**Std + QA:**

Here is **general, educational information** about reducing calcium oxalate stone risk through changes in **fluid intake only**. This is **not personal medical advice**, and you should review any changes with your clinician.

**🥤 1. Increase Your Total Fluid Intake Significantly**

Your 24-hour urine volume was **0.9 L/day**, which is **well below** the recommended target.

**Goal urine output:**

➡️ **At least 2.0–2.5 liters of urine per day**
To reach that, most people need to **drink 2.5–3.0 liters (≈ 85–100 oz) of fluid per day.**

**Why it matters**

Higher urine volume **dilutes calcium and oxalate**, lowering supersaturation and reducing stone formation risk.

**How to do it**

- Drink **2–3 cups (500–700 mL)** of fluid at each meal.
- Add **1 cup (250 mL)** between meals.
- Drink **1 cup before bed** (if tolerated).
- Increase fluids with exercise, heat, or sweating.

**💧 2. Choose the Right Types of Fluids**

**✔️ Best choices for preventing calcium oxalate stones**

- **Water** — the most effective and preferred.
- **Citrus beverages** (lemonade, lime water, orange water)
  - Provide **citrate**, which helps prevent stones by binding calcium.
- **Carbonated water** is fine, **but avoid high-sodium mineral waters**.

**✔️ Helpful strategy: lemon water**

Add **4 oz (120 mL)** lemon juice to water daily (spread out).
This may raise urinary citrate, which inhibits stones.

**🧃 3. Drinks You Should Limit or Avoid**

Some beverages increase stone risk.

**🚫 Limit**

- **Cola drinks** (both regular and diet): contain phosphoric acid → ↑ stone risk
- **Sugary drinks** (sweet tea, fruit punch) → ↑ urine calcium and oxalate
- **Grapefruit juice** (associated with higher stone risk in studies)

**❗ Be cautious with**

- **High-oxalate teas** (black tea in large amounts)
- **Coffee** is generally OK in moderation, but still count it toward total fluids.

**🕒 4. Spread Fluids Throughout the Day**

Drinking a lot once or twice a day doesn’t help.
You want **steady hydration**.

Good routines:

- Carry a water bottle and sip hourly.
- Set reminders to drink if needed.
- Aim for **very pale yellow** urine.

**🌙 5. Nighttime Hydration**

Stone-forming risk increases overnight because urine becomes concentrated.

- Drink a small glass of water before bed.
- Consider another small amount if you wake up naturally at night.

**📏 6. How to Know You’re Getting Enough**

Your repeat 24-hour urine test should show:

- **Urine volume ≥ 2.0–2.5 L/day**
- **Lower supersaturation values**

Your clinician will interpret these results with you.

**Summary: What to Change Right Now**

1. **Increase total fluid intake to 2.5–3.0 L/day.**
2. **Prioritize water** and **add daily lemon or citrus beverages**.
3. **Limit colas, sugary drinks, and grapefruit juice.**
4. **Distribute fluid intake evenly throughout the day**, including a little at night.

**SB + QB:**

Below is practical, guideline-based advice tailored for **recurrent calcium oxalate stone formers with severely low urine volume (0.9 L/day).**
I am following your required structure.

**1. Metabolic Diagnosis**

**Severe low urine volume (oliguria) leading to very high supersaturation of calcium oxalate.**
This is the *single strongest and most correctable risk factor* for stone formation according to EAU/CUA guidelines.

**2. Key Dietary Modifications (Hydration-Focused Priorities)**

1. **Target a urine output ≥ 2.0–2.5 L/day**, which usually requires **2.5–3.0 L fluid intake/day** for most patients.
   (More in hot climates, heavy sweating, or physically active individuals.)
2. **Distribute fluids evenly**, not just in a single large bolus. The kidney needs *constant dilution* to reduce supersaturation.
3. **Choose fluids strategically**: Prioritize water, but include “anchor beverages” to make intake sustainable (examples below).

**3. Sample Menu & Practical Hydration Strategy**

**A. The “Anchor Bottle” Method (Most effective for low-volume patients)**

- Use a **1-liter bottle** and commit to 3 cycles per day:
  - **Bottle 1:** Finish between waking and noon
  - **Bottle 2:** Noon–6 PM
  - **Bottle 3:** 6 PM–bedtime (adjust to avoid nocturia)

**B. Build Hydration Around Routine Activities**

- **Morning:**
  - 500 mL water while getting ready
  - Coffee/tea allowed, but does *not* replace water requirement
- **Midday:**
  - 500–750 mL water with or between meals
  - Sparkling water, herbal teas, flavored waters are all acceptable
- **Evening:**
  - 300–500 mL water post-dinner
  - Add **citrate-rich options** 2–3× per week:
    - Lemon water (2–3 tbsp lemon juice in water)
    - Lime water
    - Orange-infused water (if no hyperoxaluria)

**C. Hydration “Boosters” (Low-effort, stone-safe)**

- Carry a bottle that shows time markers.
- Apps or smartwatch reminders every 2 hours.
- Drink 250 mL with every bathroom break.
- Use flavored water enhancers **without added sugar** if plain water is hard to maintain.

**D. Fluids to Avoid or Limit**

- High-oxalate dark teas (black tea excess)
- Cola beverages (phosphoric acid increases risk)
- Very high sugar drinks (raise calcium excretion)

**4. Safety Warning**

If you have **heart failure, severe CKD, or are on fluid-restricted regimens**, you must speak to your physician before increasing fluids to 2.5–3 L/day.
If not, this hydration target is guideline-standard and safe.

**Std + QB:**

Here’s **clear, practical, kidney-stone–safe hydration advice** tailored for someone with **recurrent calcium oxalate stones** and a **very low urine volume (0.9 L/day)**.
*(General medical guidance only — always coordinate with your clinician.)*

**💧 The Most Practical Hydration Strategy**

**1. Aim for 2.5–3 liters of urine a day (not just fluid intake).**

For calcium oxalate stone prevention, producing **at least 2–2.5 L of urine/day** is one of the strongest protective factors.
Because not all fluid you drink becomes urine, most people need to **drink ~3 liters (100 oz) a day**.

**🌤️ 2. Use a *schedule*, not thirst**

Thirst usually kicks in too late. The easiest strategy:

**Drink 10–12 oz (300–350 mL) every 2–3 hours while awake.**

Example:

- Morning: 10–12 oz when waking up
- Mid-morning: 10–12 oz
- Lunch: 10–12 oz
- Afternoon: 10–12 oz
- Dinner: 10–12 oz
- Evening: 10–12 oz

This gets you close to 2–2.5 L before even adding other beverages or food water content.

**🧋 3. Carry a bottle that *forces* you to notice intake**

People with low urine volumes often underestimate intake by half.
Most effective approach:

**Use a 1-liter (32 oz) bottle and refill it 2–3 times/day.**

If the bottle isn’t empty by X time of day, you instantly see it.

**⏰ 4. Anchor drinking to routines**

Habits stick if paired with something you already do:

- After brushing your teeth → 10 oz
- With each meal → 10 oz
- Every time you check your phone or email → a few sips
- After using the restroom → drink for 5–10 seconds

This removes willpower from the equation.

**🌙 5. Hydrate early; avoid overloading at night**

Drinking heavily late can disrupt sleep and reduce adherence.

**Front-load your fluids**:

- Get **1 liter by noon**
- Get **2 liters by 6 PM**

If you exercise, live in a hot climate, or sweat a lot, increase intake accordingly.

**🧃 6. Choose stone-safe fluids**

Most helpful:

- **Water** (plain or flavored)
- **Citrus drinks** (lemon water, lime water) → provide citrate, which inhibits stones
- **Unsweetened herbal tea**

Avoid relying heavily on:

- Sugary drinks
- Excess iced tea (high oxalate)
- Cola (phosphoric acid may promote stones)

**🔍 7. Verify success with a simple urine-color rule**

Use this daily test:

**If your urine isn’t pale yellow or almost clear by mid-day → drink more.**

This is surprisingly accurate for adjustment between 24-hour collections.

**📦 8. For people who struggle with volume: “micro-drinking”**

If large drinks feel difficult:

- Sip **1–2 oz every 10–15 minutes**
- Or set your bottle to remind you: drink to the next line every hour

This method gradually adds up to >2.5 L without feeling forced.

**🧪 9. Consider tracking for 1 week**

People who log intake for even **3–7 days** usually increase their urine volume significantly.

Use any of these:

- A hydration app
- A smart water bottle
- A simple note on your phone

**🌟 Most important takeaway**

**Your urine volume of 0.9 L/day is the strongest modifiable risk factor for your stones.
If you do nothing else but raise it to 2.5 L/day, your recurrence risk drops dramatically.**

The easiest method for most people is:

👉 **Drink from a 1-liter bottle and finish it 3 times a day.**
(Once before noon, once by late afternoon, once by bedtime.)

**Case7:**

**SA + QA:**

Below is an expert, guideline-aligned interpretation based on the **EAU** and **CUA** stone-prevention recommendations.

**1. Metabolic Diagnosis**

- **Mild–moderate hypercalciuria**
  Urine calcium **7.8 mmol/day** (>6.2 mmol/day threshold for men).
  Most commonly driven by **high sodium intake** and/or **high animal protein load**, rather than high dietary calcium.
- **Hyperuricosuria**
  Urine uric acid **5.2 mmol/day** (>4.0 mmol/day for men).
  Typically associated with **high purine intake** (red meat, organ meats, seafood), high fructose intake, and excess animal protein.

Because you form **both calcium oxalate and uric acid stones**, diet must target **both hypercalciuria and hyperuricosuria simultaneously**.

**2. Key Dietary Modifications (Top Priorities)**

**➊ Reduce Sodium to <2,000 mg/day (≈ <5 g salt/day)**

High sodium directly increases urinary calcium excretion.

- Target: <2 g sodium/day (ideally 1.5–2 g).

**➋ Reduce Animal Protein to ~0.8–1.0 g/kg/day**

Excess animal protein:

- Raises calcium excretion
- Lowers urinary citrate
- Increases uric acid production

Shift protein intake toward **plant-based proteins** and **low-fat dairy**.

**➌ Reduce Purine Load for Uric Acid Control**

Limit:

- Red meat
- Organ meats
- Sardines, anchovies, mussels
- High-fructose foods/drinks

Emphasize alkalinizing foods.

**3. Sample Menu Suggestions**

**Foods to Prioritize (help with BOTH high calcium and high uric acid)**

- **Calcium sources:** milk, yogurt, kefir, cheese (normal amounts; do NOT restrict)
- **Plant proteins:** lentils, tofu, chickpeas, edamame
- **Alkalinizing fruits/vegetables:** citrus, melon, bananas, leafy greens
- **Low-sodium whole foods:** fresh meats in small portions, unsalted nuts, whole grains
- **Hydration:** 2.5–3 L/day unless medically contraindicated
- **Healthy fats:** olive oil, avocado, nuts (moderate portions)

**Daily Example Menu**

**Breakfast**

- Greek yogurt (low-sodium) with berries
- Oats cooked with milk
- Unsalted almonds
- Water or coffee/tea (no added sugar)

**Lunch**

- Lentil or tofu salad with mixed vegetables
- Olive oil + lemon dressing (no salt)
- Whole-grain bread slice (low sodium)

**Snack**

- A piece of fruit
- Plain yogurt or low-salt cheese

**Dinner**

- **Small portion (80–100 g) of chicken or fish**
- Large serving of vegetables (steamed or roasted without salt)
- Quinoa or brown rice
- Optional: citrus fruit for dessert (supports citrate)

**Foods/Patterns to Avoid or Minimize**

**For Hypercalciuria**

- High-sodium processed foods: deli meats, canned soups, packaged snacks
- Fast-food meals
- Salted nuts or chips

**For Hyperuricosuria**

- Red meat >1 serving/day
- Organ meats, game meats
- Sardines, anchovies, shellfish
- High-fructose drinks (colas, energy drinks, fruit juice in excess)

**For Both**

- Large animal-protein portions
- Low-calcium diets (increases oxalate absorption — avoid)
- Excess supplements: vitamin C >1000 mg/day or calcium supplements (unless prescribed)

**4. Safety Warning**

If you have **kidney disease, heart failure, liver disease, or are on diuretics**, fluid intake targets and high-potassium foods (fruits/vegetables) may need adjustment. If any of these apply, let me know so I can tailor the plan safely.

**Std + QA:**

Below is **general educational guidance** about nutrition for people with **both calcium oxalate and uric-acid stone risk factors**. It is *not* a substitute for personal medical care, but it explains the evidence-based dietary patterns typically recommended in your situation.

**⭐ Overview of Your Risk Factors**

You have **mixed calcium oxalate + uric acid stones** and your 24-hr urine shows:

- **Urine calcium: 7.8 mmol/day (~312 mg/day)** → *mild-to-moderately elevated*
- **Urine uric acid: 5.2 mmol/day (~870 mg/day)** → *also elevated*

Thus, your diet should aim to:

1. **Reduce urinary calcium excretion**
2. **Reduce uric acid production**
3. **Increase urine pH (make urine less acidic)** to prevent uric-acid crystallization
4. **Reduce oxalate load**
5. **Increase urine volume**

Below is a simple, combined plan.

**✅ 1. Increase Fluid Intake (Most Important for Both Stone Types)**

- Target **2.5–3 L of urine output per day** → usually **3–3.5 L of fluid intake** daily.
- Water is best. Citrus waters (lemon/lime) add citrate, which reduces stone formation.
- Spread fluids evenly through the day, including before bed.

**✅ 2. Adopt a “Low-Moderate Sodium, Adequate Calcium” Plan**

High sodium intake makes kidneys excrete more calcium.

**Reduce sodium to:**

- **≤ 2,000 mg/day (≈ 5 g salt/day)**

**Keep normal dietary calcium:**

- **1,000–1,200 mg/day from food**, not supplements (unless prescribed)
- Best sources: dairy (milk, yogurt, cheese), calcium-fortified plant milks.
- **Why?** Adequate calcium binds oxalate in the gut → lowers oxalate absorption.

**✅ 3. Reduce High-Purine Foods (for Uric Acid Stones)**

Your urine uric acid is high. Reducing purine intake lowers uric acid production.

**Limit:**

- **Red meat**, **organ meats**, **game meats**
- **Shellfish** (shrimp, lobster, crabs), anchovies, sardines, herring, mackerel
- **Beer** and other alcohol, especially in excess
- High-fructose foods and drinks (sodas, sweetened drinks)

**Prefer:**

- Poultry in moderation, eggs, plant-based proteins
- Low-fat dairy (helps reduce uric acid)

**✅ 4. Moderate Animal Protein (for BOTH stone types)**

Animal protein increases calcium excretion and lowers urine pH.

**Aim for:**

- **0.8–1.0 g of protein per kg body weight per day**
  (e.g., 70–85 g/day for most men your size)

Shift more of your protein intake toward:

- Beans, lentils, tofu, nuts (in moderation; see oxalate note below)
- Low-fat dairy
- Fish/poultry in small portions rather than large red-meat meals

**✅ 5. Reduce High-Oxalate Foods (for Calcium Oxalate Stones)**

You don’t need to eliminate oxalate entirely—just limit the highest-oxalate foods.

**High-oxalate foods to limit:**

- Spinach, Swiss chard, beets, beet greens
- Almonds, cashews, peanuts
- Soy products (esp. soy nuts, tofu processed with calcium sulfate)
- Rhubarb, okra, sweet potatoes
- Wheat bran
- Dark chocolates and cocoa

**Pair oxalate-containing foods with calcium**

Eat them together with calcium-rich foods (e.g., yogurt) to bind oxalate in the gut.

**✅ 6. Increase Citrate Intake (helps both stone types)**

Citrate inhibits calcium stone formation and raises urine pH.

**Eat more:**

- **Citrus fruits**: lemons, limes, oranges, grapefruit*
- **Lemon water**: ½–1 lemon per day in water
- **Fruits and vegetables** generally (alkalinize urine)

*Check for interactions with medications if applicable.

**✅ 7. Limit Sugar, Fructose & Excess Calories**

High sugar intake increases uric acid and promotes stones.

Avoid:

- Sugary beverages, energy drinks, sweetened teas
- High-fructose corn syrup products

**👉 Optional but Often Relevant: Weight & Urine pH**

- If you are overweight, **gradual weight loss** lowers uric acid.
- A diet rich in fruits/vegetables (DASH-style diet) raises urine pH → beneficial for uric acid stones.

**🔎 Putting It All Together — A Simple Daily Strategy**

**Eat more:**

- Vegetables & fruits (especially citrus)
- Low-fat dairy (1–2 servings/day)
- Water, lemon water
- Plant-based proteins
- Whole grains

**Eat less:**

- High-sodium foods (fast food, packaged snacks)
- Red/processed meat
- Shellfish & organ meats
- High-oxalate greens (spinach, beets)
- Nuts in large amounts
- Sugary drinks, beer, alcohol excess

**🧪 Follow-up Testing**

Because dietary changes can shift urine chemistry, clinicians usually recommend:

- **Repeat 24-hour urine after 2–3 months** to ensure calcium and uric acid have decreased and urine pH is improving.

**SB + QB:**

Below is a patient-tailored interpretation based on EAU & CUA guideline principles.
(Your provided values are interpreted with standard reference cutoffs: **Urine calcium > 6.2–6.25 mmol/day = hypercalciuria**; **Uric acid > 4.0 mmol/day = hyperuricosuria**.)

**1. Metabolic Diagnosis**

You have **two concurrent metabolic abnormalities**:

**a. Hypercalciuria (Urine Ca 7.8 mmol/day)**

This degree of urinary calcium loss is most commonly **dietary sodium–driven** or **animal-protein–driven**, not due to dietary calcium itself.

**b. Hyperuricosuria (Urine uric acid 5.2 mmol/day)**

This is usually caused by **high intake of animal protein**, especially red meat, organ meats, shellfish, and high-fructose foods.

Because you form **both calcium oxalate and uric acid stones**, the diet must target **both abnormalities simultaneously**.

**2. Key Dietary Modifications (Top Priorities)**

**Priority 1 — Reduce sodium intake to < 2 g/day (≈ 5 g salt/day)**

High sodium → higher urinary calcium loss.
**This is the #1 intervention for your hypercalciuria.**

Specific goals:

- Avoid salted snacks, processed meats, canned soups, pickled foods.
- Choose “no-salt added” versions whenever possible.

**Priority 2 — Moderate animal protein (particularly red/organ meats)**

High animal protein increases **uric acid production**, lowers urine pH, and raises urinary calcium.

Targets:

- Limit meat/poultry/fish to **0.8–1.0 g/kg/day total protein**
  (usually ~1–2 palm-sized portions/day).
- Replace some animal protein with plant proteins (beans, lentils, tofu).

**Priority 3 — Maintain normal dietary calcium (1000–1200 mg/day)**

This prevents intestinal oxalate absorption and protects against calcium oxalate stones.

Use: dairy yogurt, milk, fortified plant milks, cheese **taken with meals** that contain oxalate (e.g., spinach, nuts).

**3. Sample Menu Suggestions**

**Breakfast (Good Choices)**

- Oatmeal with milk or calcium-fortified almond milk
- Greek yogurt with berries
- Whole grain toast with peanut butter
  **Avoid:** processed breakfast sandwiches, salted butter, bacon, ham.

**Lunch**

**Better options:**

- Grilled chicken (small portion), quinoa salad, steamed vegetables
- Lentil soup (low-sodium), whole grain bread
  **Avoid:**
- Deli meats, canned soups, ramen, large beef burgers, salty sauces (soy sauce, teriyaki).

**Dinner**

**Better options:**

- Baked salmon or tofu + roasted vegetables + brown rice
- Stir-fry with mixed vegetables, tofu, and low-sodium soy sauce alternative
  **Avoid:**
- Red meat daily
- Organ meats
- High-salt marinades, pre-seasoned meats
- Shellfish feasts (high purine load)

**Snacks**

**Good:** fruit, nuts (moderation), yogurt, low-salt popcorn
**Avoid:** chips, salted nuts, jerky, processed cheeses.

**Hydration Targets**

- 2.5–3.0 L/day **if no heart failure or kidney impairment**.
- Aim urine pH 6.0–6.5 for uric acid stone prevention (via citrus fruits or potassium citrate if prescribed).

**4. Safety Warning**

**If you have any of the following**: chronic kidney disease, heart failure, hypertension requiring diuretics, or are on potassium-sparing medications →
**DO NOT increase potassium-containing foods or citrate supplements without clinician oversight.**

**Bottom Line**

Given your urine chemistry, the **main dietary drivers** are:

👉 **#1 Cut sodium** (biggest impact on your urine calcium).
👉 **#2 Reduce animal protein** (improves both calcium and uric acid levels).
👉 **Do NOT cut dietary calcium** — keep it normal to prevent oxalate absorption.

**Std + QB:**

Below is **general educational information** about diet and kidney-stone prevention. It is **not medical advice** and **cannot replace your own clinician’s guidance**, especially since stone prevention depends on your full medical history, labs, medications, and imaging.

**📌 Key idea for mixed calcium-oxalate + uric-acid stones**

When both stone types are present, prevention usually targets **multiple factors**:

1. **High sodium intake → raises urinary calcium**
2. **High animal-protein intake → increases urine uric acid & lowers urine pH**
3. **Low urine volume → concentrates stone-forming substances**

Your urine values:

- **Calcium 7.8 mmol/day (~312 mg/day)** → slightly above typical target (<6.2 mmol/day / <250 mg/day).
- **Uric acid 5.2 mmol/day (~87 mg/day)** → moderately elevated.

This means **both sodium restriction and animal-protein moderation matter**. But which should you prioritize?

**🥇 What to prioritize based on physiology & your numbers**

**1. Reduce sodium (salt) → Best first target**

Because:

- High sodium intake directly **increases urinary calcium excretion**.
- Reducing salt often lowers urinary calcium significantly.
- Good target: **<2,300 mg sodium/day**, ideally **1,500 mg/day** if feasible.

This is especially important because your **urinary calcium is above goal**.

**2. Moderate animal protein (meat, poultry, fish, seafood)**

Because:

- Animal protein increases **uric acid production** and lowers **urine pH**, both of which promote uric-acid stones.
- It also raises calcium excretion (a double hit for mixed stones).
- You don’t need to eliminate meat — just **reduce large portions** and **avoid high-purine meats**.

Targets:

- About **0.8–1.0 g protein/kg body weight/day**, focusing more on plant protein sources.
- Limit red meat and organ meats especially.

**3. Increase fluid intake → Universal priority**

Target:

- **Urine volume ≥ 2.5 L/day**, often requiring **3 L/day fluid intake**.

This reduces recurrence risk across all stone types.

**Other helpful strategies (depending on your clinician’s advice)**

**Adequate dietary calcium (don’t restrict calcium!)**

- Aim for **1,000–1,200 mg/day** from food.
- Helps bind oxalate in the gut → reduces calcium oxalate stones.

**Reduce very high-oxalate foods only if needed**

E.g., spinach, almonds, beets, rhubarb, Swiss chard.
But: don’t over-restrict; pair them with calcium-containing foods.

**Increase fruits and vegetables**

Raises urinary citrate and alkalinizes urine → protective for both CaOx and uric-acid stones.

**If your urine pH is low (common with uric-acid stones)**

Dietary alkalinization helps: fruits, vegetables, lemon/lime water.
Clinicians sometimes prescribe **potassium citrate** if appropriate.

**🎯 So what should YOU focus on most right now?**

**Your top priorities based on your values are:**

**#1 Reduce salt**

Strongest impact on your elevated urine calcium.

**#2 Reduce animal protein (especially red/organ meats)**

Helps both uric-acid and calcium-oxalate stone risk.

**#3 Maintain high fluid intake**

Essential for both stone types.

After that: ensure adequate dietary calcium, watch very high-oxalate foods, and increase fruits/vegetables.

**Case8:**

**SA + QA:**

**1. Metabolic Diagnosis**

The patient has **enteric hyperoxaluria** (from Crohn’s-related fat malabsorption), reflected by **elevated urine oxalate (1.5 mmol/day)** and **very low urine volume (1.2 L/day)**—a strong driver of recurrent calcium oxalate stone formation.

**2. Key Dietary Modifications (Top 3 Priorities)**

**1. Increase Fluid Intake to Achieve ≥2.3–2.5 L Urine/Day**

- Your current volume (1.2 L) nearly doubles stone risk.
- Aim to *drink* ~2.7–3.0 L/day to ensure the urine target is met.
- Use frequent small sips if Crohn’s symptoms limit intake.

**2. Reduce Oxalate Absorption Using Calcium With Meals**

Because Crohn’s can cause fat malabsorption, unabsorbed fatty acids bind calcium → leaving oxalate free for absorption.

- **Take 300–400 mg calcium *with each meal*** (dietary preferred; supplements allowed if meals low in calcium).
- Do NOT restrict dietary calcium.

**3. Follow a Low-Oxalate, Low-Fat Diet**

- Limit oxalate-rich foods to ≤100 mg/day.
- Keep fat intake moderate (≈25–30% of calories) to reduce enteric oxalate absorption.

**3. Sample Menu Suggestions**

**Foods to Prioritize**

These help bind oxalate, reduce absorption, and maintain gut tolerance.

**Breakfast Options**

- Oatmeal cooked with milk, topped with blueberries (low oxalate).
- Scrambled eggs + whole-grain toast + yogurt.

**Lunch Options**

- Grilled chicken salad with lettuce, cucumber, bell peppers; olive-oil dressing.
- Rice bowl with salmon, steamed carrots, and a serving of cottage cheese (calcium source).

**Dinner Options**

- Baked cod or turkey, mashed potatoes, green beans.
- Rice pasta with chicken and cream sauce; side of low-oxalate vegetables (zucchini, cauliflower).

**Snacks**

- Cheese sticks, yogurt, apples, grapes, rice cakes.

**Hydration**

- Water, herbal teas, electrolyte beverages without excessive sodium.
- Avoid high–vitamin C drinks (>200 mg/day).

**Foods to Limit or Avoid (High Oxalate)**

- Spinach, almonds, peanuts, cashews
- Beets, rhubarb, swiss chard
- Sweet potatoes
- Dark chocolate, cocoa
- Wheat bran
- Black tea in excess
- Soy products (variable but often high)

**4. Safety Warning (Specific to Crohn’s Disease)**

- **If you experience active flare-ups**, avoid very high fluid loads in short periods—spread hydration throughout the day to prevent diarrhea worsening.
- **Fat restriction should not be extreme**; too little fat can cause malnutrition.
- If you have **short bowel syndrome** or significant malabsorption, calcium supplements *with meals* may be necessary—discuss with your gastroenterologist.
- Monitor for **electrolyte disturbances** if diarrhea is frequent, especially sodium and potassium.

**Std + QA:**

I can give **general, educational guidance** about factors that *typically* help reduce calcium oxalate stone recurrence. However, this is **not a substitute for medical advice**, especially because you also have **Crohn’s disease**, which can change how your body absorbs oxalate and nutrients. Always discuss any diet plan with your gastroenterologist and nephrologist.

**⭐ What Your Results Suggest**

- **Urine oxalate: 1.5 mmol/day (~135 mg/day)** → Upper-normal to mildly elevated; Crohn’s disease increases risk of **enteric hyperoxaluria**, even when levels appear only moderately high.
- **Urine volume: 1.2 L/day** → Too low. The single most powerful way to prevent stones is increasing urine volume to **>2.0–2.5 L/day**.

**🔹 1. Increase Fluid Intake (Top Priority)**

Aim for **at least 2.5 liters of urine per day**, which usually requires:

**✔️ Goal**

**Drink 3.0–3.5 L (100–120 oz) of fluid/day**, unless your doctors have given limits.

**✔️ Best fluids**

- Water
- Mineral water or flavored unsweetened sparkling water
- Citrus beverages (e.g., diluted lemonade) → increase **citrate**, a natural stone inhibitor

**✔️ Avoid or limit**

- High-sugar sodas
- Very high doses of vitamin C drinks (>500 mg/day vitamin C increases oxalate)

**🔹 2. Reduce Oxalate Absorption (Especially Important in Crohn’s Disease)**

Crohn’s disease can lead to fat malabsorption, which increases oxalate absorption in the gut. Even if your urine oxalate isn’t extremely high, controlling dietary oxalate and pairing it with calcium at meals is helpful.

**✔️ Eat calcium with every meal**

This is the **most effective diet strategy** for Crohn’s patients with oxalate stones.

- Aim for **800–1,200 mg/day** from food
- Spread across meals
- Examples:
  - Dairy: milk, yogurt, cheese
  - If lactose intolerant: calcium-fortified plant milks, lactose-free dairy

**Why?** Calcium binds oxalate in the gut so it cannot be absorbed.

**🔹 3. Moderate High-Oxalate Foods (Don’t eliminate all)**

Avoid the highest-oxalate foods **especially without calcium-containing foods**.

**Foods to *limit*:**

- Spinach (very high)
- Almonds and nut butters
- Beets
- Rhubarb
- Swiss chard
- Dark chocolate
- Wheat bran
- Sweet potatoes
- Soy / tofu (varies)

**Foods that are *fine in moderation*:**

- Berries
- Tea
- Coffee
- Cocoa in small amounts
- Most vegetables

**Important:** Strict oxalate restriction *alone* doesn’t help much unless combined with **adequate calcium intake**.

**🔹 4. Lower Sodium Intake**

High sodium increases urinary calcium.

**✔️ Goal: <2,000 mg sodium/day**

Practical steps:

- Cook at home when possible
- Avoid salty processed foods
- Choose low-sodium broths, sauces

**🔹 5. Moderate Animal Protein**

Too much increases calcium and decreases citrate.

**✔️ Aim: 0.8–1.0 g protein/kg/day unless you require more for Crohn’s nutrition.**

Prefer:

- Fish
- Poultry
- Plant-based proteins that are low in oxalate (lentils, certain beans)

**🔹 6. Increase Citrate Intake**

Citrate naturally prevents calcium stone formation.

**✔️ Good sources:**

- Lemons/limes (diluted juice daily)
- Orange juice (small amounts if tolerated)
- Fruits/vegetables in general

**🔹 7. Consider Crohn’s-Specific Issues**

Because Crohn’s disease increases stone risk through malabsorption:

**If you have diarrhea or fat malabsorption:**

- **Avoid very high-fat diets** → unabsorbed fat increases oxalate absorption
- Ensure adequate hydration with electrolytes
- Discuss with your doctor the possibility of:
  - **Calcium supplementation with meals**
  - **Bile acid binders** (when malabsorption is bile-acid–related)
  - **Potassium citrate** if urine citrate is low (requires testing)

**✔️ Summary: Best Diet + Fluid Strategy for You**

**Most impactful changes based on your urine profile + Crohn’s disease:**

1. **Increase fluids to achieve >2.5 L urine/day**
2. **Take 300–400 mg calcium with each meal** (food or supplement under physician guidance)
3. **Limit high-oxalate foods; pair moderate oxalate foods with calcium**
4. **Sodium <2,000 mg/day**
5. **Moderate animal protein**
6. **Increase dietary citrate (lemon/lime)**
7. **Avoid high-fat meals if they worsen malabsorption**

**SB + QB:**

Below is your personalized, guideline-based prevention plan for **Crohn’s disease with enteric hyperoxaluria** and **low urine volume**, formatted exactly as requested.

**1. Metabolic Diagnosis**

You have **enteric hyperoxaluria** secondary to Crohn’s-related fat malabsorption.
This leads to:

- **Very high urine oxalate (1.5 mmol/day)** due to increased intestinal oxalate absorption.
- **Low urine volume (1.2 L/day)**, which further concentrates oxalate and calcium, greatly increasing calcium oxalate stone risk.

**2. Key Dietary Modifications (Top Priorities)**

**1) Pair calcium with every meal/snack containing oxalate**

This is *critical* in Crohn’s.
Calcium binds oxalate in the gut → prevents absorption → lowers urine oxalate.

- Target: **400–600 mg/day**, distributed with meals.
- Use **lactose-free dairy**, calcium-fortified milk alternatives, or calcium citrate if dairy intolerant.

**2) Adopt a *low-fat, low-oxalate* meal pattern that is gentle on the bowel**

Crohn’s-related fat malabsorption increases unbound fatty acids → these bind calcium → more oxalate left free to absorb.

- Limit dietary fat to **< 50–60 g/day** (individual tolerance varies).
- Avoid high-oxalate foods (especially if your bowel is inflamed).

**3) Increase fluid intake in a *bowel-tolerant* way (goal 1.8–2.2 L urine/day)**

Large fluid boluses worsen diarrhea. Use:

- Small, frequent sips
- Oral rehydration solutions (ORS)
- Avoid hypertonic drinks (juice, soda), which may worsen diarrhea

**3. Sample Menu Suggestions**

**Foods to Prioritize**

**Low-fat, lower-oxalate, gut-friendly options:**

- **Proteins:** chicken, turkey, eggs, white fish, tofu
- **Calcium sources with meals:** lactose-free milk, fortified plant milks, yogurt alternatives, calcium citrate 200–300 mg with meals
- **Carbohydrates:** white rice, pasta, couscous, oats
- **Vegetables (low oxalate, low residue):** zucchini, cauliflower, cucumbers, lettuce, green beans
- **Fruits (low oxalate):** bananas, melons, mango, papaya, peeled apples
- **Hydration:** ORS, diluted electrolyte drinks, herbal teas, water in small divided doses

**Foods to Avoid or Limit (High Oxalate or High Fat)**

**High-oxalate foods to avoid:**

- Spinach, Swiss chard, beets, almonds/almond flour, peanuts, potatoes with skin
- Chocolate/cocoa, rhubarb, wheat bran
- Berries (esp. raspberries), soybeans, miso, tahini

**High-fat foods that worsen fat malabsorption and oxalate absorption:**

- Fried foods, cream sauces, fatty cuts of meat
- Full-fat cheese, butter-heavy meals
- Coconut milk/cream

**Example Daily Menu (Crohn’s-friendly & Oxalate-controlled)**

**Breakfast**

- Oatmeal cooked with lactose-free or fortified oat milk
- Banana
- *Calcium citrate 200 mg with the meal*

**Lunch**

- Grilled chicken breast
- White rice
- Steamed zucchini
- Small ORS drink (100–200 mL)

**Snack**

- Low-fat yogurt alternative or rice crackers
- Sips of water every 15 minutes

**Dinner**

- Baked white fish
- Mashed potatoes (peeled; low oxalate)
- Green beans
- *Calcium citrate 200 mg with this meal*

**Evening hydration**

- Herbal tea or electrolyte drink (diluted)

Aim for **2.2–2.5 L of total fluid intake/day**, spaced out every hour.

**4. Safety Warning**

- Because you have Crohn’s disease, **avoid very high fiber and rough raw vegetables during flare periods**, as they worsen diarrhea and fluid loss.
- If you have **short bowel syndrome**, salt needs may increase, and **ORS is preferred** over plain water to avoid worsening dehydration.
- If you experience active diarrhea, fluid and sodium losses rise; work with your gastroenterologist to avoid dehydration.

**Std + QB:**

Below is **general, educational information** about diet and kidney-stone risk in Crohn’s disease. It is **not medical advice** and cannot replace guidance from your treating gastroenterologist or nephrologist, who knows your specific clinical status, labs, medications, and surgical history.

**Why Crohn’s Disease Raises Oxalate Stone Risk**

People with Crohn’s—especially with **fat malabsorption**, ileal disease/resection, or frequent diarrhea—often absorb **far more oxalate** from food. This is called *enteric hyperoxaluria*. When fat isn’t absorbed properly, it binds calcium in the gut, leaving oxalate unbound and more easily absorbed.

Your labs show:

- **High urine oxalate: 1.5 mmol/day (~135 mg/day)** → very elevated
- **Low urine volume: 1.2 L/day** → strong risk factor for stone formation

So the two priorities are:

1. **Reduce oxalate absorption**, and
2. **Increase urine volume**, in ways your Crohn’s symptoms can tolerate.

**What You Can Realistically Eat and Drink With Crohn’s Disease**

**1. Hydration: The Highest-Impact Intervention**

Even if diet changes are difficult, raising urine volume dramatically lowers stone risk.

**Realistic strategies for people with bowel sensitivity:**

- Take **frequent small sips** instead of large boluses of fluid (large volumes may trigger urgency or diarrhea).
- Use **oral rehydration solutions (ORS)** or low-sugar electrolyte drinks — they improve absorption and decrease diarrhea compared to plain water.
- Aim for:
  **≥ 2–2.5 L/day of total fluid intake** *as tolerated*, with the goal of ≥ **2 L urine/day**.
  (Even reaching 1.6–1.8 L/day is meaningful improvement.)

Avoid:

- High-sugar beverages (can worsen diarrhea),
- High-vitamin C drinks (converts to oxalate).

**2. Pair Oxalate-Containing Foods With Calcium**

This method is especially important in Crohn’s because calcium binds oxalate *in the gut*, preventing absorption.

**How to do it:**

At **each meal**, include **200–300 mg of calcium** from food or supplements such as:

- Lactose-free milk/yogurt if tolerated
- Fortified plant milks
- Calcium citrate with food (often better tolerated, and citrate helps stones)

If dairy worsens Crohn’s, use:

- **Calcium citrate** split 2–3×/day **with meals**
- Calcium-fortified lactose-free products

Important: **Don’t take calcium supplements on an empty stomach**—they won’t bind oxalate effectively.

**3. Limit High-Oxalate Foods — But Not All Plant Foods**

A strict low-oxalate diet is usually impractical and unnecessary. Instead, avoid *very* high sources:

**Foods often worth minimizing:**

- Spinach
- Almonds and nut butters
- Beets
- Rhubarb
- Swiss chard
- Wheat bran
- Dark chocolate
- Sweet potatoes
- Soy products (very high)
- Black tea in large amounts

**Foods that are usually safer (lower oxalate):**

- White rice
- Most fruits (except berries in huge quantities)
- Most vegetables (carrots, cauliflower, cucumber, zucchini, lettuce)
- Animal proteins
- Oats (moderate)
- White bread/pasta
- Eggs

Your goal is **not zero oxalate**, but to reduce the big offenders and bind oxalate with calcium.

**4. Increase Citrate Intake (a Natural Stone Inhibitor)**

If your gut tolerates citrus:

- Lemon or lime juice diluted in water
- Low-acid or pulp-free versions if you are sensitive
- Avoid high-sugar lemonade → consider sugar-free versions

If not tolerated, your doctor may consider **potassium citrate**, but Crohn’s patients with diarrhea or electrolyte issues must be monitored closely.

**5. Moderate Fat Intake (When Fat Malabsorption Is Present)**

High-fat diets worsen oxalate absorption in Crohn’s disease.

If you have steatorrhea or post-ileal resection symptoms:

- Use **lower-fat cooking methods** (grill, bake, steam).
- Spread fat intake through the day.
- Consider **medium-chain triglycerides (MCT oil)** if recommended—absorbed differently and less likely to worsen malabsorption.

If fat is well tolerated and malabsorption is minimal, you don’t need aggressive fat restriction.

**6. Avoid Excess Vitamin C**

More than **250–500 mg/day** can convert to oxalate.
Avoid large-dose supplements and high-C energy drinks.

**7. Salt & Protein: Keep Moderate**

- High sodium → more calcium in urine.
- Large animal-protein loads → lower citrate and raise acid load.

But **malnutrition risk in Crohn’s is real**, so protein should *not* be overly restricted. Focus instead on:

- Moderate portions
- Well-tolerated lean proteins (chicken, fish, eggs)

**Putting It All Together: A Realistic Sample Day**

**Breakfast**

- Oatmeal with **calcium-fortified** milk alternative
- Banana
- Water with a splash of lemon OR an electrolyte drink

**Lunch**

- Grilled chicken with white rice and zucchini
- Lactose-free yogurt (calcium source)
- ORS / electrolyte drink in small sips

**Snack**

- Crackers or rice cakes
- Avoid nuts or nut butters

**Dinner**

- Baked salmon, carrots, potatoes
- Small glass of calcium-fortified beverage with the meal

**Throughout the day**

- Fluid intake spread evenly
- Avoid spinach salad, almonds, soy-based snacks, dark chocolate, and black tea

**When to Seek Personalized Care**

Because Crohn’s varies drastically between individuals, your care team may need to tailor strategies considering:

- Location and severity of disease
- Presence of fat malabsorption
- History of resections
- Electrolyte issues
- Risk of dehydration
- History of metabolic complications

A **renal dietitian familiar with inflammatory bowel disease** can be extremely helpful.

**Case9:**

**SA + QA:**

Below is your response **strictly following your requested structure** and tailored to **struvite (infection) stones**, which behave very differently from metabolic calcium stones.

**1. Metabolic Diagnosis**

You do **not** have a metabolic stone disorder.
Your findings — **urine pH 7.8 and high ammonium** — are classic for **infection-driven struvite stone formation**, caused by **urease-producing bacteria** (e.g., *Proteus, Klebsiella, Providencia*).

These bacteria split urea → **ammonia**, which alkalinizes the urine (pH 7.5–9.0), leading to precipitation of **magnesium ammonium phosphate (struvite)**.

👉 **Diet does NOT drive struvite stones. Infection does.**

**2. Key Dietary Modifications (Top 3 Priorities That Actually Matter)**

Diet plays a supportive—but not primary—role. The focus is reducing conditions that worsen infection and stone growth.

**1. Optimize Hydration to Reduce Bacterial Concentration**

- Goal: **2–2.5 L/day of urine output** (unless heart failure or CKD limits fluid).
- Steady hydration improves bladder flushing and reduces bacterial load.

**2. Avoid Excess Urinary Alkalinization**

Your urine is already **too alkaline**, so avoid foods/supplements that raise pH further:

- Avoid: **alkaline waters**, bicarbonate drinks, potassium citrate, “alkaline diets”.

**3. Support Urinary Tract Health (NOT stone chemistry)**

These do not dissolve struvite stones, but reduce recurrence of infection:

- **Cranberry** or **D-mannose** may help reduce *E. coli* UTIs (but less effective for *Proteus*).
- **Moderate animal protein**: high loads promote bacterial growth by increasing urinary substrate.
- **Increase dietary fiber** → improves gut microbiome and reduces overall infection risk.

**3. Sample Menu Suggestions**

**Foods That Support UTI Prevention (Evidence-based)**

- **Berries (cranberry, blueberry)** → may reduce bacterial adhesion.
- **High-fiber foods**: oats, beans, vegetables, whole grains.
- **Lean proteins**: poultry, tofu, fish (avoid very high protein loads).
- **Fluids**: water, herbal teas, citrus-free beverages.

**Foods to Limit Because They Further Alkalinize Urine**

(Your urine is already too alkaline from the infection.)

- Sparkling waters with bicarbonate
- “Alkaline” bottled waters
- High-dose fruits/vegetables in juiced or concentrated form
- Excessive citrus (orange, lemon juices)
- Supplements containing citrate or bicarbonate

**Foods That Have *No* Real Impact on Struvite Stones**

- Dietary calcium
- Oxalate
- Sodium (except for blood pressure management)
- Purines/uric acid

These matter for calcium or uric acid stone formers—not for infection stones.

**4. Safety Warning**

Because struvite stones arise from **infection**, diet alone **cannot prevent progression**. Key medical points:

- **Persistent alkaline urine + high ammonium = likely ongoing urease-producing infection.**
- Struvite stones **grow very rapidly**—they can form large staghorn calculi within months.
- Curative therapy typically requires:
  - Complete **stone clearance** (endoscopic or percutaneous)
  - **Eradication of infection** with culture-directed antibiotics
  - Sometimes **urease inhibitors** (e.g., acetohydroxamic acid) if infection cannot be cleared

If you have **CKD, heart failure, or are on fluid restrictions**, hydration targets must be individualized.

**Bottom Line for You**

**Diet has a minor supportive role. Infection control is the main therapy.**
Your elevated urine pH and ammonium reflect active bacterial urease activity, not something you are eating.

**Std + QA:**

Below is **general educational information** about diet and stone-prevention. It is **not medical advice** and cannot replace your clinician’s guidance—especially for struvite stones, which are medically complex.

**Key Point: With Struvite Stones, Diet Plays a Smaller Role Than Infection Control**

Struvite stones are **infection-related stones**, caused by bacteria that produce urease (e.g., *Proteus*, *Klebsiella*, some *Staphylococcus* species).
Because the bacteria convert urea into **ammonia**, your urine becomes **very alkaline (high pH)** and **high in ammonium**, creating the perfect environment for struvite to form.

👉 **So the #1 “diet intervention” for struvite stones is actually effective diagnosis and treatment of the underlying UTI**, plus complete removal of stone material so bacteria cannot persist inside it.

Diet can help with hydration and urinary flow, but cannot fix the biochemical issue that infection is driving.

**What Diet CAN (and Cannot) Do for Struvite Stones**

**✔️ Helpful Diet Approaches**

**1. Maintain High Fluid Intake**

The one universally helpful measure:

- Aim for **2–3 L of urine output per day**, unless your clinician advises otherwise.
- Water is best; citrus-containing fluids are fine, but for struvite stones they are not essential because alkalinization isn’t helpful here (your urine is already alkaline).

**Why it helps:**
Dilutes bacteria and minerals, may reduce stone growth rate.

**2. Adequate Protein, but Avoid Excess**

High protein intake **can acidify urine**, which is actually *not harmful* in struvite stone patients and might slightly reduce ammonia formation.
But:

- Do **not** drastically restrict protein at your age.
- Focus on **moderate, balanced** intake (e.g., ~0.8 g/kg/day unless otherwise directed).

**3. Limit Foods That Promote UTIs (Indirect Effect)**

Not strong evidence, but some patients find these helpful:

- Avoid high-sugar drinks if they worsen UTI frequency.
- Ensure good hydration around sexual activity.
- Cranberry products are **not proven** for struvite-forming bacteria but may help with *E. coli* UTIs.

**❌ Diet Strategies That Do *NOT* Help Struvite Stones**

These are important because they often get recommended for other stone types, but **do not apply** here:

**1. Oxalate restriction**

- Struvite stones contain **no oxalate**, so this is irrelevant.

**2. Low-sodium diet to reduce calcium stones**

- Good for calcium stones, but has **no direct impact** on struvite formation.

**3. Trying to acidify urine with food alone**

- Food cannot meaningfully change a urine pH of **7.8** when urease-producing bacteria are present.
- Only medical treatment (antibiotics, stone removal) can address this.

**4. Citrus, lemonade, or potassium citrate to “raise citrate”**

- These **increase urine pH**, which is *bad* for struvite stones.

**So What Actually Matters Most?**

In struvite stone disease, the real drivers are **bacterial infection + alkaline pH + ammonium formation**.

Meaningful prevention requires:

**1. Complete stone removal**

Residual struvite acts as a nidus for persistent infection.

**2. Culture-directed antibiotic therapy**

Long-term or suppressive antibiotics are sometimes needed—**food cannot replace this**.

**3. Follow-up imaging & urine cultures**

Detects recurrence early.

**4. In selected cases: urine acidification with medications**

Examples include **acetohydroxamic acid (AHA)**, which decreases bacterial urease activity.
This is **not** a dietary intervention and has side effects, so it’s used only when indicated.

**Practical Daily Diet Tips (the helpful part you *can* control)**

- Drink enough to keep urine **light yellow to clear**.
- Keep a **consistent meal pattern**; avoid extremely high-protein fad diets.
- Maintain a healthy weight, which reduces lifetime UTI risk.
- Stay well hydrated during travel, heat exposure, or periods of limited mobility.
- If constipation worsens UTIs for you, increase fruits/vegetables & fiber.

**Summary for You**

Given:

- **68-year-old man**
- **Recurrent struvite stones**
- **Repeated UTIs**
- **Urine pH 7.8 + high ammonium**

👉 **Diet will not correct the high pH or ammonium because infection is the true cause.**

Diet matters mainly for **hydration**, **general urinary health**, and **avoiding factors that worsen UTIs**, but **the real prevention strategy is medical and procedural**, not dietary.

**SB + QB:**

Below is a clear, guideline-based response tailored to **infection (struvite) stones**, which behave very differently from metabolic calcium stones.
*Note: No image groups or entities are appropriate here because this is purely clinical guidance.*

**1. Metabolic Diagnosis**

**The patient has infection-related (struvite) stone formation associated with recurrent UTIs from urease-producing bacteria, leading to alkaline urine (pH 7.8) and high ammonium.**
This is **not** a metabolic stone driven by diet. The root cause is **chronic bacterial colonization**, not calcium, oxalate, sodium, or protein intake.

**2. Key Dietary Modifications (Top 3 Priorities)**

**1. Prioritize Hydration — but understand this cannot cure infection stones**

- Aim for **2.5–3 L/day fluid**, unless medically contraindicated.
- Hydration reduces stone growth and prevents urinary stagnation, but **cannot eliminate bacteria** or dissolve struvite stones.

**2. Avoid excess alkalinizing beverages (because your pH is already high)**

Do **NOT** rely on:

- “Urine-alkalinizing” drinks (e.g., citrate powders marketed for UTI relief)
- Coconut water
- Milk in large quantities
- Baking soda mixtures
  These **raise urinary pH further**, which favors **struvite stone growth**.

**3. Prefer neutral or mildly acidifying fluids**

These **do NOT dissolve stones**, but help avoid pushing urine pH even higher:

- **Water (best choice)**
- **Coffee or tea** (without excessive milk)
- **Cranberry products** (slightly acidifying; may reduce certain bacterial adhesion but *not* urease-producers)
- **Blackberry, blueberry juices** (small amounts, not sugary)

**3. Sample Menu Suggestions**

**Fluids to Choose**

- Water, sparkling or still
- Unsweetened cranberry drink (not for stone dissolution, but okay for general UTI prevention)
- Herbal teas (non-alkalinizing; no baking soda additives)
- Moderate coffee

**Fluids to Avoid**

- Urinary alkalinizers: potassium citrate powders, sodium bicarbonate, “alkaline water”
- High-milk diets if they raise pH further (normal dairy intake is fine)
- Fruit smoothies heavy in banana/mango (tend to be alkalinizing and sugary)
- Sugary sodas (promote bacterial growth and worsen metabolic health)

**Foods to Focus On**

- Lean proteins, vegetables, whole grains
- Vitamin C–containing foods in normal dietary amounts (helps general immunity)
- Anti-inflammatory foods (berries, leafy greens, omega-3 rich fish)

**Foods to Avoid in Excess**

These do not “cause” struvite stones but can worsen UTIs or maintain alkalinity:

- Very high-sugar foods → increase UTI risk
- Heavy dairy intake
- Strong alkalinizers: baking soda, alkaline diet regimens

**4. Safety Warning & What Diet Cannot Fix**

**Critical point: Diet cannot dissolve or cure infection stones.**

Per EAU & CUA guidelines, **struvite stones form because urease-producing bacteria split urea into ammonium and raise pH**.
This cycle continues **until the stones are removed and the infection is eradicated**.

**You should NOT rely on diet alone to fix:**

- **Elevated urine pH** from urease activity
- **High ammonium production**
- **Recurrent UTIs due to stone colonization**
- **Existing struvite stones** (they often require *complete removal*, sometimes with percutaneous nephrolithotomy)

**When to be cautious:**

- If you have **CKD**, **heart failure**, or **fluid restrictions**, do not exceed prescribed fluid limits.
- If you are on **potassium-sparing drugs or ACE inhibitors**, avoid potassium citrate products (but you should avoid them anyway due to already high urine pH).

**Bottom Line**

- **Struvite stones are infection-driven, not diet-driven.**
- Hydration and avoiding alkalinizing drinks help prevent worsening, but **definitive treatment requires clearing infection and complete stone removal.**
- After stone removal, monitoring for recurrent bacteriuria is key.

**Std + QB:**

Below is **general educational information**, not personal medical advice or diagnosis. Infection-related stones—typically **struvite** (magnesium-ammonium-phosphate) or **carbonate-apatite**—behave very differently from the more common calcium oxalate stones. Diet helps *support* prevention, but **cannot cure or adequately prevent infection stones on its own** because the root problem is bacterial.

**🔍 Why infection stones form**

Infection stones form when **urease-producing bacteria** (e.g., *Proteus*, *Klebsiella*, *Morganella*, *Providencia*, some *Staphylococcus*) break down urea → ammonia → raise urine pH (often >7.2).
Your findings—**pH 7.8** and **high ammonium**—fit this pattern.

As long as the bacteria are present, the urine will stay alkaline and stones can keep growing.

**✅ What you *can* do with fluids & diet**

These steps help **support UTI prevention and hydration**, but **do not treat the infection stone process itself**.

**✔️ 1. Drink enough to produce ≥2–2.5 L urine/day**

This dilutes bacteria and stone components.

**Helpful fluids**

- **Plain water** (best choice)
- **Citrus-based drinks** **without added sugar**
  – e.g., lemon water or water with a splash of citrus
  (These mildly acidify urine *in some people*, but their main benefit is just improved hydration.)

**Possibly helpful for UTI prevention**

Evidence is mixed but reasonable as adjuncts:

- **Cranberry products** (juice low in sugar or tablets) may reduce some bacterial adherence but **do not work against all UTI bacteria**, and **do not acidify urine enough to stop struvite formation**.
- **D-mannose** may help with *E. coli* UTIs, but infection stones usually arise from *Proteus* species, where D-mannose is **not helpful**.

**🚫 What to *avoid***

**✖️ 1. Avoid trying to “alkalize” further**

Your urine is already alkaline from the infection. Avoid supplements marketed as alkalizers (e.g., magnesium citrate blends, “alkaline water,” baking soda) because they **increase urine pH**, which **accelerates struvite stone growth**.

**✖️ 2. Avoid sugary drinks**

- Promote bacterial growth
- Increase UTI risk
  Examples: soda, sweetened teas, energy drinks, fruit punch.

**✖️ 3. Avoid relying on cranberry juice with high sugar**

If you use cranberry, stick to unsweetened preparations.

**⚠️ What diet cannot fix (most important part)**

Diet **cannot**:

**❌ Eradicate urease-producing bacteria**

Antibiotics (guided by culture) and sometimes long-term suppression are often needed.

**❌ Lower urine pH meaningfully in infection stones**

The bacteria themselves create ammonia → raise pH. Food cannot counteract this.

**❌ Dissolve struvite stones**

These stones typically **require complete surgical removal** (e.g., percutaneous nephrolithotomy) because any leftover fragment can serve as a “scaffold” for regrowth.

**❌ Prevent recurrence if infection persists**

As long as the infection continues, stones can reform rapidly—even with a perfect diet.

**🧭 What *should* be addressed medically**

Your primary prevention strategy will come from **medical—not dietary—management**:

**1. Get a urine culture**

Identify if a urease-producing organism is present.

**2. Ensure complete stone removal**

Residual fragments → recurrent infection → recurrent stones.

**3. Culture-directed antibiotics**

Sometimes chronic suppressive therapy is needed until stones are cleared.

**4. Monitor urine pH and ammonium**

Persistently high pH usually means ongoing infection.

**🥤 Practical daily plan (diet + fluids)**

- **Drink primarily water.**
- Aim for **2.0–2.5 L urine output** daily (usually ~3 L intake).
- Add **citrus water** for variety—but do *not* expect it to lower pH significantly.
- Limit or avoid:
  - sugary drinks
  - alkaline waters
  - supplements that raise pH
  - excessive alcohol (dehydrating)
- If considering cranberry or D-mannose, use them **only as adjuncts**, not treatment.

**Case10:**

**SA + QA:**

Below is your **evidence-based, guideline-aligned metabolic interpretation and diet plan**, tailored for **brushite (calcium phosphate) stone disease**, where the key danger is **high urine pH + high urine calcium**—a combination that strongly promotes brushite crystallization.

No images or entities are needed for this clinical task.

**1. Metabolic Diagnosis**

You have **hypercalciuria (urine calcium 9.0 mmol/day ≈ 360 mg/day)** combined with **alkaline urine (pH 6.8)**.
For brushite stone formers, **pH ≥ 6.5** significantly increases risk.
This pattern is most consistent with:

**“Calcium phosphate stone disease driven by hypercalciuria—likely sodium- and/or protein-related—superimposed on excessively alkaline urine.”**

Your priority is to **reduce urinary calcium** *without further increasing urine pH*.

**2. Key Dietary Modifications (Top 3 Priorities)**

**1. Lower Sodium to < 2 g/day sodium (≈ 5 g salt/day)**

High dietary sodium increases urinary calcium excretion by reducing renal tubular calcium reabsorption.
✔ Low sodium = lower urine calcium
✘ Does NOT increase urine pH → safe for brushite disease

**Most important single dietary change.**

**2. Moderate Animal Protein (0.8–1.0 g/kg/day)**

Animal protein restriction lowers urine calcium and reduces acid load.
However: *excess* reduction can **increase urine pH** (bad for brushite).
So aim for **moderation**, not a low-protein diet.

Guideline-aligned targets:

- ~50–70 g/day for most women
- Emphasize **fish, poultry, legumes** over large red meat portions

**3. Maintain *normal* dietary calcium (1,000–1,200 mg/day) but taken WITH meals**

You should **not** restrict calcium—this worsens oxalate absorption and is not helpful for calcium phosphate stones.

Calcium with meals helps:

- Reduce intestinal oxalate absorption
- Avoid secondary hyperoxaluria
- Does **not** raise urine pH

Sources (neutral pH effect): yogurt, cheese, milk with meals.

**3. Sample Menu Suggestions**

**✔ Foods to Emphasize**

These lower urine calcium or avoid raising pH:

**Breakfast**

- Greek yogurt (unsweetened) + berries
- Oatmeal made with calcium-fortified milk
- Whole-grain toast with peanut butter
  (*All neutral acid-base effect*)

**Lunch**

- Grilled chicken salad with olive oil + vinegar
- Brown rice or quinoa (moderate portions)
- Low-sodium vegetable soup

**Dinner**

- Baked salmon or tofu
- Steamed vegetables (broccoli, carrots, zucchini)
- Whole-grain pasta or potatoes (small to moderate portions)

**Snacks**

- Almonds (small handful)
- Cheese sticks
- Fresh fruit
- Low-sodium crackers

**✘ Foods to Limit or Avoid (important for brushite)**

**High-sodium foods (major driver of your hypercalciuria)**

- Restaurant meals
- Cured meats (ham, bacon, sausage)
- Packaged soups
- Pickles, olives
- Chips, crackers unless low-sodium

**Excess animal protein (can worsen hypercalciuria)**

- 120 g meat per meal
- High-protein diets or protein powders

**Alkali-raising foods**

These can increase urine pH further:

- Large amounts of fruits/vegetables **in smoothies or juice form**
- Plant-based milks with added alkalizing agents (check labels)

Moderation is key—you should not avoid fruits/vegetables entirely.

**4. Safety Warning**

No kidney-related comorbidities were mentioned, so general kidney-stone hydration targets apply: **aim for ≥2.0–2.5 L urine/day**, unless you have heart failure, cirrhosis, or advanced CKD.
If any of those conditions apply, fluid intake must be individualized.

**Std + QA:**

Below is **general educational information** you can discuss with your clinician. I’m not diagnosing or giving individualized medical advice.

**🌿 Diet Strategies for Calcium Phosphate (Brushite) Stones**

Brushite stones form more readily when **urine pH is high (typically >6.2–6.5)** *and* urine calcium is elevated.
Your results show:

- **Urine pH: 6.8** → *high*, encourages Ca-phosphate crystallization
- **Urine calcium: 9.0 mmol/day (~360 mg/day)** → *elevated*

So the goal is to **reduce urinary calcium** *without further raising urine pH*.
This means your plan will differ somewhat from typical calcium-oxalate stone diets (which often encourage alkalinizing foods).

**✅ What Usually Helps in Calcium Phosphate Stone Formers**

**1. Moderate—but don’t restrict too low—dietary calcium**

- Aim for **~1,000–1,200 mg/day** from food (e.g., dairy with meals, fortified alternatives).
- Adequate calcium **binds dietary phosphate and oxalate**, reducing intestinal absorption.
- Avoid calcium supplements unless prescribed (they can raise urinary calcium).

**2. Reduce sodium intake**

High sodium → increased urinary calcium excretion.
**Target:**

- **<2,000 mg sodium/day** (about 5 g salt).
- Avoid: processed foods, salted snacks, deli meats, takeout.

**This is one of the most effective ways to lower urine calcium.**

**3. Moderate animal protein, especially red meat**

Animal protein increases acid load (↓ urine pH), which is actually **not harmful here**, but **too much protein increases urinary calcium**.

Recommendation:

- **0.8–1.0 g protein/kg/day** (≈50–70 g/day for many women)
- Prioritize: poultry, fish, plant proteins
- Limit: red meat, organ meats, high-protein supplements

**4. Be cautious with high-alkali diets**

For calcium phosphate stone formers, foods that strongly raise urine pH should **not** form the core of your diet.

You don’t need to avoid all fruits/vegetables, but:

**Use in moderation:**

- Large servings of spinach, kale, collard greens
- Very high fruit/veg smoothie intake
- Coconut water, aloe drinks
- Vegetarian diets high in alkali load

**Prefer:**

- Neutral–slightly alkaline fruits/vegetables in normal portions:
  berries, apples, carrots, broccoli, cauliflower, zucchini.

**5. Limit phosphorus-additive foods (not natural phosphorus)**

Excess phosphate load may influence brushite formation.

Avoid foods with additives such as:

- “**phos**-” ingredients in processed foods
- Soda with phosphate additives (cola, dark sodas)

Natural phosphorus foods (beans, nuts, meats, dairy) are fine in normal portions.

**6. Hydration – but not with alkaline waters**

Goal urine volume: **≥2.0–2.5 L/day**

Avoid:

- High-alkalinity waters (pH > 8)
- Waters containing potassium citrate (unless prescribed)

Use:

- Tap water
- Neutral bottled water
- Herbal teas (not strongly alkaline)

**7. Potassium citrate caution**

This supplement is **commonly used** for kidney stones but **contraindicated or used carefully in calcium phosphate stones** because it **raises urine pH**.

Do *not* take citrate supplements unless your nephrologist confirms a citrate deficiency and adjusts the dose.

**🍽️ Example Day of Eating That *Does Not* Over-alkalinize Urine**

**Breakfast**

- Oatmeal with blueberries
- Low-fat milk or fortified alternative
- Water or herbal tea

**Lunch**

- Grilled chicken salad with lettuce, tomatoes, cucumbers; light vinaigrette
- Whole-grain bread slice
- Water

**Snack**

- Greek yogurt or a small serving of nuts (not salted)

**Dinner**

- Baked salmon
- Steamed broccoli or green beans
- Brown rice
- Water

**What this plan avoids:**

- Excess salt
- Highly alkalinizing smoothies
- Sugary sodas
- Excess animal protein
- Highly alkaline waters

**🔍 Key Targets to Aim For**

| **Goal** | **Why** |
| --- | --- |
| Sodium < 2 g/day | Lowers urine calcium |
| Adequate calcium 1–1.2 g/day (food) | Reduces phosphate and oxalate absorption |
| Protein 50–70 g/day | Avoids excess urine calcium |
| Fluids 2–2.5 L/day | Dilutes calcium + phosphate |
| Avoid alkalinizing supplements | Prevents urine pH from rising further |

**🧪 Discuss With Your Clinician**

- Whether **thiazide diuretics** are appropriate (they lower urine calcium).
- Whether you have **low urinary citrate**, which complicates treatment.
- Review of any medications or supplements that affect pH (antacids, citrate, bicarbonate, topiramate).

If you want, I can help you design **a personalized weekly meal plan**, or analyze specific foods or supplements you’re wondering about.

**SB + QB:**

Below is a **clinical-grade, guideline-based explanation** tailored to **calcium phosphate (CaP) stone formers**, where **alkaline urine** is the central driver.

**1. Metabolic Diagnosis**

You form **calcium phosphate stones** in the setting of:

- **Alkaline urine (pH 6.8)** → This strongly promotes CaP crystallization (hydroxyapatite/brushite).
- **Hypercalciuria (9.0 mmol/day ≈ 360 mg/day)** → Above guideline thresholds (EAU/CUA cutoffs ~4–6 mmol/day or 200–250 mg/day).

**Interpretation:**
Your risk is driven by **hypercalciuria combined with a urine pH that is *already too alkaline***. In CaP stone formers, further alkalinization from certain citrate supplements may worsen supersaturation of CaP.

**2. Key Dietary Modifications (Top Priorities)**

**1) Lower Urine Calcium by Lowering Sodium & Moderating Animal Protein**

- Every **1 g of sodium** can increase urinary calcium excretion.
- High animal protein → increases calcium excretion and reduces citrate.

**Targets:**

- **Sodium < 2 g/day (≈ 5 g salt/day)**
- **Animal protein ~0.8–1.0 g/kg/day**

**2) Use Citrate Carefully (Food-Based Citrate > Supplements)**

- **Potassium citrate tablets can raise urine pH further**, which is risky in CaP stones.
- **Food-based citrate (lemons/limes)** increases citrate but raises pH *much less* than pharmacologic citrate.

**Conclusion:**

- **Avoid potassium citrate supplements unless prescribed with pH monitoring.**
- Prefer **dietary citrate** which helps bind calcium without excessively alkalinizing urine.

**3) Maintain Normal Dietary Calcium (1000–1200 mg/day)**

This prevents increased gut oxalate absorption AND helps regulate calcium metabolism.
**Do NOT restrict calcium intake.**

**3. Sample Menu Suggestions**

**Foods to Prioritize**

| **Goal** | **What to Eat** |
| --- | --- |
| **Increase citrate safely** | Fresh lemon/lime juice in water (1–2 Tbsp/day), oranges (small), berries. |
| **Normalize calcium** | Dairy or fortified plant milks with meals; yogurt; cheese in moderate portions. |
| **Lower urine calcium through sodium reduction** | Fresh vegetables, whole grains, unsalted nuts, homemade meals. |
| **Moderate animal protein** | Beans, lentils, tofu, fish, poultry in moderate amounts. |

**Example Day**

**Breakfast**

- Oatmeal cooked with milk
- Fresh berries
- Unsalted almonds (small handful)
- Water with a squeeze of lemon (not whole lemon concentrate)

**Lunch**

- Grilled chicken or tofu bowl
- Brown rice or quinoa
- Steamed vegetables
- Avoid salted sauces (soy sauce, teriyaki)

**Snack**

- Yogurt (plain or lightly sweetened)
- Fruit (orange, berries)

**Dinner**

- Baked salmon or lentil stew
- Roasted vegetables
- Small portion of whole-grain bread (unsalted if possible)

**Hydration**

- Water flavored with lemon/lime slices (NOT alkalinized waters).

**Foods to Limit**

| **Category** | **Why** |
| --- | --- |
| **High-sodium foods** (processed meats, canned soups, fast food) | Drives hypercalciuria. |
| **High-alkali supplements** (potassium citrate tablets, alkaline water) | Further raises urine pH → worsens CaP risk. |
| **Excess animal protein** (large meat portions, keto diets) | Raises urine calcium & lowers citrate. |
| **High-phosphate sodas** | May worsen calcium-phosphate dynamics. |

**4. Safety Warning**

Because your **urine is already alkaline**, **avoid potassium citrate supplements unless your urologist monitors your pH and CaP supersaturation**. They can be beneficial **only if** citrate is low **and** pH remains < 7.0.
Food-based citrate is **safer** because it increases citrate without sharply raising pH.

**Short Answer to Your Question**

**Yes—citrate supplements *can* backfire in CaP stone formers with alkaline urine.**
But **dietary citrate from fruits** is beneficial and safe. The priorities are **reducing sodium**, **moderating protein**, and **maintaining normal calcium intake**.

If you'd like, I can create a **personalized diet plan** once you share your **full 24-hour urine data**, stone composition breakdown, and any comorbidities.

**Std + QB:**

I can give **general, educational information** about diet and stone-risk mechanisms, but **not personal medical advice or treatment instructions.** Always confirm changes with your clinician, especially since calcium-phosphate (CaP) stone formers differ from calcium-oxalate (CaOx) stone formers in some key ways.

**🧪 Why calcium-phosphate stone formers are different**

CaP stones tend to form when:

- **Urine pH is high** (usually > 6.5)
- **Urine calcium is elevated**
- **Citrate is low** (citrate usually helps… but things are more nuanced for CaP stones)

You already have:

- **Urine pH:** 6.8 (on the higher side for CaP formation)
- **Urine calcium:** 9.0 mmol/day (borderline high depending on lab ranges)

**🍋 “Could citrate backfire for me?”**

**Possibly, yes.**
Potassium citrate is often used to prevent calcium stones, but:

- **Citrate itself is protective** because it binds calcium and reduces crystallization.
- **BUT potassium citrate also raises urine pH**, sometimes significantly.
- For a CaP stone former whose urine pH is *already* high, further alkalinization could **increase CaP supersaturation** and make stones more likely.

So it’s not that citrate is “bad,” but that **the pH-raising effect may outweigh the benefit** in some CaP stone formers. This is why doctors often individualize citrate therapy and monitor pH closely.

**Food-based citrate (lemon/lime juice)** usually raises citrate only modestly and has **less pH effect**, so its impact is less predictable but generally milder.

**🥗 What dietary patterns *generally* help CaP stone risk?**

**1️⃣ Maintain—but don’t restrict—dietary calcium**

Low calcium intake increases oxalate absorption and can paradoxically worsen stone risk overall.

Aim for normal dietary calcium from foods such as:

- Dairy in normal portions
- Leafy greens
- Calcium-fortified plant milks

**Avoid calcium *supplements*** unless your clinician prescribes them, as supplemental calcium can raise urine calcium.

**2️⃣ Reduce sodium intake**

This is one of the most impactful steps for CaP stone formers.

Higher sodium → more calcium excretion in urine.

Goal:

- **< 2,000 mg sodium/day** (varies by guideline)
- Avoid processed foods, fast foods, heavily salted snacks.

**3️⃣ Moderate animal protein**

High animal protein:

- Raises urine calcium
- Lowers citrate
- Can influence acid–base balance

But unlike CaOx stone formers, your urine pH is already high, so:

- You *don’t* need extreme protein reduction
- Just keep protein to moderate, food-based amounts
  (e.g., spread through the day, plant-based proteins included)

**4️⃣ Increase fruits & vegetables — but avoid over-alkalinizing**

Most fruits/vegetables are beneficial because they reduce dietary acid load and improve citrate, *but* extremely alkaline diets can push urine pH even higher.

You **do not** need to avoid them — just avoid:

- “Alkaline diet” plans
- Excessive fruit/vegetable juicing
- Large amounts of potassium citrate–rich supplements

Balanced plant intake is still protective for kidneys long-term.

**5️⃣ Avoid high-oxalate foods in large quantities**

Even though your stones are CaP, high urine oxalate still contributes to calcium-based stones generally.

Limit:

- Spinach
- Almonds
- Beets
- Rhubarb
- Dark chocolate
- Excessive nuts

You don’t need to eliminate them, just moderate.

**6️⃣ Stay well hydrated**

The single strongest dietary intervention across *all* stone types.

Goal:

- **≥ 2–2.5 liters of urine/day**
- Spread fluid throughout the day
- More during exercise or heat exposure

Water, diluted citrus drinks (if not causing pH rise), and herbal teas are fine.

**🥤 About citrus drinks (lemon, lime, orange)**

- **Lemon & lime juice:** Raise urine citrate modestly and *minimally* raise pH → often safest if you want citrate.
- **Orange juice:** More alkalinizing → may raise pH more.
- **Potassium citrate supplements:** Strongly alkalinizing → risk of increasing CaP supersaturation.

If your doctor is considering citrate therapy, they usually monitor **24-hour urine pH and supersaturation** to see its effect.

**📝 Summary: For CaP stone formers with alkaline urine**

**Helpful:**

- Normal calcium intake from food
- Low-sodium diet
- Moderate protein
- Hydration
- Moderate citrus intake (prefer lemon/lime over OJ)
- Balanced fruits & vegetables (not extreme alkaline diets)

**Potentially harmful if overdone:**

- Potassium citrate supplements (due to ↑ pH)
- Very alkaline diets or juices
- High-sodium foods
- Calcium supplements (unless prescribed)

**Case11:**

**SA + QA:**

Below is your cystinuria-specific prevention plan based on EAU/CUA guideline principles and tailored to your urine pH of **5.5**, which is too acidic for cystine solubility.

**1. Metabolic Diagnosis**

**Cystinuria with persistently low urine pH (5.5) leading to poor cystine solubility and increased cystine stone formation risk.**
Not due to diet, but diet and hydration strongly affect cystine solubility.

**2. Key Dietary Modifications (Top Priorities)**

**1) Extreme high fluid intake—goal > 3.5–4 L/day total intake**

- Cystine becomes soluble only when urine volume is very high.
- Aim for **> 3 L of urine output/day**, which usually requires drinking **3.5–4 L/day**, more if you exercise or live in a hot climate.

**2) Alkali-rich diet to raise urine pH toward 7.0–7.5**

Cystine solubility rises sharply above pH ~7.
Prioritize:

- Fruits/vegetables high in natural alkali (citrate and bicarbonate precursors).
- Potassium-rich plant foods **unless** you have renal impairment (you don’t indicate any).

**3) Moderate animal protein restriction**

Animal protein → acidic urine → cystine precipitation.
Target **≤ 0.8–1.0 g/kg/day**, emphasizing plant proteins and limiting:

- Red meat
- Poultry
- Fish
- Eggs

(Not zero protein—moderation.)

**3. Sample Menu Suggestions**

**Foods to Emphasize (help alkalinize urine)**

- **Fruits:** oranges, lemons, limes, melon, banana, kiwi
- **Vegetables:** spinach, kale, broccoli, zucchini, sweet potato
- **Plant proteins:** lentils, chickpeas, tofu, beans
- **Beverages:**
  - Water (primary)
  - Sugar-free lemonade (alkali promoting)
  - Herbal teas

**Foods to Limit**

- **Animal proteins:** red meat, chicken, fish, eggs
- **High-sodium foods:** canned soups, fast food, chips, deli meats
  - Sodium increases urine cystine excretion.
  - Target **< 2 g sodium/day** (≈ 5 g salt).

**Daily Structure Example**

**Breakfast:**
Oatmeal with banana + glass of water + herbal tea

**Lunch:**
Quinoa bowl with chickpeas, roasted vegetables, and lemon dressing

**Snack:**
Fresh fruit + 500 mL water

**Dinner:**
Stir-fried tofu with broccoli, zucchini, and rice

**Evening goal:**
Sip water to reach **3.5–4 L intake** for the day

**4. Safety Warning**

- High fluid intake is safe **only if you have no heart failure or advanced kidney disease**.
- Very high-potassium foods are generally safe for healthy young adults, but **if you ever develop kidney dysfunction, potassium-rich alkalinizing diets must be used cautiously.**
- Do **not** start potassium citrate supplementation without medical supervision—your pH goal is ~7–7.5, and overshooting can create calcium phosphate stones.

**Std + QA:**

Below is **general, educational guidance** commonly recommended for people with **cystinuria and cystine stones**. This **does not replace your clinician’s individualized medical advice**, but it can help you understand what is typically advised.

**🧪 Why Diet & Fluids Matter in Cystinuria**

Cystine stones form when **cystine concentration in urine becomes too high**, especially when:

- Urine volume is low
- Urine is acidic (your pH of 5.5 is low)
- Sodium and certain proteins (especially methionine-rich animal proteins) are high

The goals are to:

1. **Dilute urine** (high fluid intake)
2. **Alkalinize urine** (raise pH to 7.0–7.5)
3. **Lower urinary cystine levels** (primarily by reducing sodium and moderating animal protein)

**💧 FLUID ADVICE (Most Important)**

**✔ Aim for VERY high urine output**

- **Target urine volume:** **>3 liters per day** (many experts recommend **3–4 L/day** for cystinuria)
- Drink **throughout the day**, not in big intervals
- **Drink before bed** and consider a glass of water **during the night**, because stones form more easily during overnight concentration

**What to drink:**

- Water is best
- Lemon water or citrate-containing drinks can help alkalinize (but alkalinization is usually medication-driven, not purely dietary)

**🍽 DIET ADVICE**

**1. Reduce Sodium**

High sodium → more cystine in urine → more stones

- Limit to **<2 grams of sodium/day** (≈ <5 g salt/day)
- Avoid: processed foods, fast food, chips, canned soups, cured meats

**2. Moderate Animal Protein**

Animal protein increases cystine production and acid load (lowers pH).

- Prefer **plant-based proteins** when possible
- Limit large servings of:
  - Red meat
  - Poultry
  - Eggs
  - Fish
- You **do not need to eliminate animal protein**, but avoid high-protein diets.

**3. Increase Fruits & Vegetables**

These help alkalinize urine (raise pH):

- Citrus fruits
- Melons
- Leafy greens
- Potatoes, squash

Plant-forward diets generally shift pH upward.

**4. Do NOT restrict calcium**

Stone type here is **cystine**, not calcium.
Adequate calcium intake is normal and safe.

**🧂 Why Sodium Restriction Matters Most**

In cystinuria, sodium intake is directly linked to cystine excretion.
Less sodium → less cystine in urine → fewer stones.
This is more impactful than protein restriction for most patients.

**🌡 Urine pH Goal**

Your urine pH (5.5) is **too acidic**, increasing cystine stone risk.
Diet helps a little, but achieving **pH 7.0–7.5** usually requires prescribed alkalinizing therapy such as:

- **Potassium citrate**
- **Sodium bicarbonate** (not preferred due to sodium load)

Talk to your clinician about medication-based alkalinization if you are not already using it.

**📌 Summary of Best Practices**

**Most important changes to reduce cystine stone risk:**

1. **Drink enough to produce >3 L urine/day**
2. **Restrict sodium to <2 g/day**
3. **Moderate animal protein intake; favor plant sources**
4. **Increase fruits/vegetables for alkalinization**
5. **Work with your clinician to maintain urine pH 7.0–7.5**

**SB + QB:**

Below is your **evidence-based, guideline-aligned metabolic plan** for cystinuria, following EAU/CUA recommendations and your requested output format.

**1. Metabolic Diagnosis**

**You have cystinuria with persistently acidic urine (pH 5.5), which greatly increases cystine stone formation.**
Your major metabolic problems are:

- **Excess urinary cystine concentration** (inherent to cystinuria)
- **Low urine pH** → cystine is far less soluble below pH 7.0
- **Likely low urine volume**, unless fluid intake is extremely high

**2. Key Dietary Modifications (Top Priorities)**

**Priority 1 — Urine Dilution**

- Target **> 3.0 L urine output/day**
  → Usually requires **3.5–4.0 L fluid intake/day** (more if hot climate or heavy sweating).
- Goal: Keep **urine specific gravity < 1.010**.

**Priority 2 — Urinary Alkalinization**

- Raise urine pH to **7.0–7.5** (ideal for cystine solubility).
- Use **alkali-rich beverages** and low-acid foods.
- **Potassium citrate** is the standard pharmacologic option (not water-soluble baking soda unless medically supervised).

**Priority 3 — Reduce Cystine Production**

- Restrict **sodium to < 2 g/day (≈ 5 g salt)** → lowers cystine excretion.
- Restrict **animal protein to 0.8–1.0 g/kg/day** → decreases acid load & cystine generation.

**3. Sample Menu Suggestions**

**Fluids: What You SHOULD Drink**

✔ **Alkaline water** (pH ≥ 8–9 preferred if available)
✔ **Citrate-containing beverages:**

- Lemon water
- Lime water
- Low-sugar lemonade
  ✔ **Herbal teas** (non-caffeinated)
  ✔ **Plain water** (most of total intake)
  ✔ **Coconut water** (moderate; contains potassium)

**How Much to Drink**

- **Spread fluid intake across the day and night**.
- Set alarms to drink every **2–3 hours**.
- Drink **400–500 mL before bed** and **a small amount if you wake overnight** (critical — cystine stones often form at night due to concentration).

**Foods to Prioritize (Alkalinizing, Low-Sodium, Moderate-Protein)**

✔ **Fruits & vegetables** (strongest alkalinizing effect)
✔ **Legumes, tofu, quinoa** (lower sulfur amino acids → lower cystine production)
✔ **Whole grains**
✔ **Low-sodium dairy or plant milks**
✔ **Fresh herbs, lemon, lime** for flavor instead of salt
✔ **Nuts** (in moderation — do *not* affect cystine the way oxalate stones do)

**Foods to Limit or Avoid**

❌ **High-sodium foods:**

- Fast food
- Cured meats
- Chips, crackers, salted nuts
- Canned soups
- Restaurant meals (ask for no salt)

❌ **High animal protein intake**

- Limit beef, pork, lamb
- Keep poultry and fish to **1–2 modest servings/day max**

❌ **Acidifying drinks**

- Cola
- Excess coffee
- Sports drinks with high sodium
- Cranberry juice (acidic; worsens cystinuria)

**4. Safety Warning**

- **If you have heart failure, advanced CKD, or are on potassium-sparing medications**, discuss with your physician before increasing potassium-rich foods or using potassium citrate — risk of hyperkalemia.
- Extremely high fluid intake may be unsafe if you have cardiac or renal fluid-handling issues.
- Never attempt extreme sodium restriction (<1 g/day) without guidance.

**Summary Targets (Print or Screenshot This)**

| **Parameter** | **Goal** |
| --- | --- |
| **Daily fluid intake** | 3.5–4.0+ L/day |
| **Urine output** | > 3 L/day |
| **Urine pH** | 7.0–7.5 |
| **Sodium intake** | < 2 g/day (≈ 5 g salt) |
| **Protein intake** | 0.8–1.0 g/kg/day, minimize red meat |
| **Nighttime hydration** | Yes — 300–500 mL before bed |

**Std + QB:**

Here is **evidence-based, clinically standard, but general (not personal medical)** guidance for **cystinuria with recurrent cystine stones**, with a focus on **hydration, urine alkalinization, and diet**.
Because cystinuria is a genetic condition requiring individualized management, please review these recommendations with your nephrologist/urologist before making changes.

**🥤 1. What Should You Drink?**

**Primary Goal: Very high fluid intake**

Cystine becomes much more soluble when urine is very dilute.

**Target urine volume:**

- **At least 3 liters/day of urine output**
- This usually requires **3.5–4+ liters/day of fluid intake**, depending on your climate, activity level, and sweating.

**Best fluids**

- **Water** (mainstay)
- **Carbonated water** is fine
- **Citrate-containing beverages**, such as:
  - *Lemon water / lemonade made with real lemon juice*
  - *Orange juice* (alkalinizes urine but contains sugar—use in moderation)

**Avoid or limit**

- Drinks high in sodium (some sports drinks)
- Cola beverages (acidifying and increase stone risk)
- Excess caffeine (mild diuretic; not dangerous but can reduce net hydration)

Try to **spread fluids throughout the day**, including one large glass before bed and possibly during the night, since cystine stones often form when urine becomes concentrated overnight.

**🧪 2. What Should Your Urine pH Be?**

Cystine solubility rises sharply as pH increases.
Your current urine pH of **5.5 is too acidic** and promotes stone formation.

**Target pH**

- **pH 7.0–7.5**, with many guidelines recommending **7.5** for cystinuria if tolerated.

**How to raise pH**

The most effective and commonly prescribed treatment is:

- **Potassium citrate** (prescription)
  - Typically divided 2–3 times daily.
  - Avoid sodium bicarbonate as a primary therapy because added sodium increases cystine excretion.

Use home urine pH strips to see how your urine responds and share the data with your clinician.

**🍽️ 3. Diet Targets for Cystinuria**

**✔ Low sodium**

High sodium ↑ cystine excretion → more stones.

**Target sodium intake:**

- **< 2,300 mg/day**, and ideally **< 1,500 mg/day**.

This is one of the **most important** dietary interventions for cystinuria.

**✔ Moderate protein intake**

Cystine comes from an amino acid (cysteine), but restricting protein too much is unhealthy.
General recommendations:

- **0.8–1 g protein per kg body weight/day**
  (Example: if you weigh 70 kg → about 56–70 g/day)
- Limit **animal protein**, especially:
  - Red meat
  - Poultry
  - Fish (large portions)

Plant-based proteins are generally more favorable.

**✔ Increase fruits & vegetables**

These foods naturally alkalinize urine and support citrate levels.

Examples:
Leafy greens, bananas, oranges, melons, potatoes, avocados, legumes.

**✔ No need to excessively restrict oxalate**

Cystine stones are *not* oxalate stones, so typical oxalate avoidance diets do not apply.

**⚠️ 4. What About Supplements or Medications?**

For recurrent cystine stones **despite high hydration and alkaline urine**, clinicians may add:

- **Tiopronin** (first-line cystine-binding medication)
- **D-penicillamine** (second line, more side effects)

These are prescription drugs and require monitoring of blood counts and kidney function.

**📌 Summary of Targets**

| **Category** | **Target** |
| --- | --- |
| **Daily fluid intake** | Aim for **3.5–4+ L/day** |
| **Urine output** | **≥ 3 L/day** |
| **Urine pH** | **7.0–7.5** |
| **Sodium intake** | **< 1,500–2,300 mg/day** |
| **Protein intake** | ~**0.8–1 g/kg/day**, emphasize plant sources |

**🩺 Important safety notes**

- Do **not** start potassium citrate without clinician approval if you have chronic kidney disease, are on ACE inhibitors/ARBs, or have a history of high potassium.
- Monitor urine pH regularly; pH above 7.5 may raise risk of calcium phosphate stones.
- Cystinuria usually requires **lifelong management**.

**Case12:**

**SA + QA:**

Here is your structured, guideline-based response:

**1. Metabolic Diagnosis**

**You have hypercalciuria likely driven by *excessive sodium intake*.**
Your urine calcium (10 mmol/day) is elevated **because urine sodium is extremely high (300 mmol/day)**.
EAU & CUA guidelines: **For every ~100 mmol increase in sodium intake, urinary calcium rises by ~1 mmol.**

**2. Key Dietary Modifications (Top Priorities)**

**Priority #1 — Reduce sodium intake to <100 mmol/day (~2.3 g sodium or ~5–6 g salt).**

This is the single most important intervention to lower urinary calcium.

**Additional helpful changes:**

2. **Maintain normal dietary calcium (1000–1200 mg/day).**
   – Prevents increased intestinal oxalate absorption.
3. **Moderate animal protein (especially red meat and processed meats).**
   – Reduces acid load and urinary calcium.

**3. Sample Menu Suggestions**

**Foods to Eat (Low Sodium + Stone-Friendly)**

- Fresh poultry, fish, or lean meat seasoned with herbs, lemon, garlic (not salt).
- Plain rice, quinoa, oats.
- Fresh fruits and vegetables (especially citrus for citrate).
- Dairy with normal calcium content: milk, yogurt, cheese (in moderate amounts).
- Nuts in small portions (avoid very salty roasted varieties).

**Foods to Avoid or Greatly Limit (High Sodium Offenders)**

- Fast food, take-out meals, cured meats (bacon, sausage, deli meats).
- Canned soups, instant noodles, frozen entrées.
- Restaurant foods (often >2,000 mg sodium per meal).
- Snack foods: chips, salted nuts, crackers.
- “Hidden sodium” items: soy sauce, salad dressings, ketchup, sports drinks.

**One-Day Example (Low Sodium)**

- **Breakfast:** Oatmeal cooked in milk with berries; unsalted almonds.
- **Lunch:** Grilled chicken breast + quinoa + steamed vegetables; olive oil + lemon dressing.
- **Snack:** Greek yogurt or fruit.
- **Dinner:** Baked salmon with herbs + roasted potatoes + spinach.

**4. Safety Warning**

You did not report kidney disease, heart failure, or other conditions requiring fluid or potassium restriction.
If you **do** have hypertension requiring a low-sodium diet or are on diuretics, speak to your clinician before making large changes.

**Summary**

Your top priority is **lowering sodium intake**. This will directly reduce urinary calcium excretion and your risk of recurrent calcium oxalate stones.

**Std + QA:**

For **recurrent calcium oxalate stones**, the *single most important dietary change* for you—based on the numbers you provided—is to **reduce your sodium (salt) intake**.

**🔍 Why sodium reduction should be your top priority**

Your 24-hour urine sodium is **300 mmol/day**, which is **very high** (target is generally **<100–150 mmol/day**). High sodium intake directly increases urinary calcium excretion. For someone forming calcium oxalate stones, this is one of the strongest dietary drivers of stone risk.

**How it works**

- High sodium → kidney excretes more sodium
- Calcium is carried with that sodium → urinary calcium rises
- More urinary calcium = more risk of calcium oxalate stone formation

Even if your urine calcium (10 mmol/day ≈ 400 mg/day) is only modestly elevated, **lowering sodium can substantially reduce it**, often by 20–30%.

**🎯 What to do**

Aim for **<2 grams of sodium per day** (~5 g salt), which is:

- No added salt in cooking or at the table
- Avoiding processed foods (soups, deli meats, canned foods, fast food, snacks)
- Choosing “low sodium” versions when possible

**👥 Why not calcium restriction?**

People often believe they need to cut calcium—but **that is not recommended**. Normal dietary calcium (1000–1200 mg/day) actually **reduces** oxalate absorption and lowers stone risk.

**🥤 Other helpful measures (secondary priorities)**

These are also important but not *your* top priority:

- **Increase fluid intake** (target urine output ≥ 2–2.5 L/day)
- **Moderate animal protein** consumption
- **Ensure adequate dietary calcium**
- **Limit high-oxalate foods** *only if urine oxalate is elevated* (not provided here)

**✅ Bottom line**

**Cutting sodium intake is the single most important dietary change for you**, because your very high urinary sodium is almost certainly driving your urinary calcium up and increasing stone recurrence risk.

**SB + QB:**

Below is your metabolic interpretation and personalized dietary plan based on EAU/CUA stone-prevention guidelines, written exactly in your requested format.

**1. Metabolic Diagnosis**

You have **marked hypernatriuria** (urine sodium 300 mmol/day), which is strongly driving a **secondary hypercalciuria** (urine calcium 10 mmol/day ≈ 400 mg/day).
In calcium oxalate stone formers, **high sodium is one of the most powerful promoters of urinary calcium loss**, independent of dietary calcium. Your recurrence pattern fits this mechanism.

**Diagnosis:** *Sodium-driven hypercalciuria contributing to recurrent calcium oxalate nephrolithiasis.*

**2. Key Dietary Modifications (Top 3 Priorities)**

**Priority #1 — Aggressively reduce sodium to < 100 mmol/day (≈ 2–2.3 g sodium or 5–6 g salt/day).**

This alone can reduce urinary calcium by **25–40%**.
What to stop *first*:

- Processed, packaged, and restaurant foods (they account for 70–80% of sodium intake, not the salt shaker).

**Priority #2 — Maintain normal dietary calcium (1000–1200 mg/day) with meals.**

Do **NOT** restrict calcium — that increases oxalate absorption and worsens stone formation.
Use dairy or calcium-fortified alternatives *with meals* to bind oxalate in the gut.

**Priority #3 — Moderate animal protein intake.**

Aim for **0.8–1.0 g/kg/day**.
High protein → increases sodium excretion, calcium, and acid load → lowers citrate.

**3. Sample Menu Suggestions (Eat vs Avoid)**

**Foods to STOP or SEVERELY LIMIT (major sodium sources)**

- Deli meats, bacon, sausages
- Canned soups, ramen, cup noodles
- Salty snacks: chips, crackers, popcorn mixes
- Fast food & take-out
- Restaurant sauces: soy sauce, teriyaki, BBQ, gravy
- Pre-seasoned chicken, frozen entrées
- Sports drinks and electrolyte powders (hidden sodium)

**Foods to Choose More Often**

**Breakfast options**

- Plain yogurt + berries + oats
- Omelet with vegetables (no cured meats)
- Oatmeal with milk or fortified almond milk

**Lunch options**

- Grilled chicken/fish with brown rice and vegetables
- Homemade lentil or bean salad (no canned salted versions unless rinsed thoroughly)

**Dinner options**

- Baked salmon with lemon + steamed vegetables + quinoa
- Stir-fry with fresh ingredients using herbs, garlic, lemon instead of soy sauce

**Calcium-rich foods to include daily**

- Milk, yogurt, kefir, cheese (moderate portions)
- Calcium-fortified plant milks
  Take with meals to reduce oxalate absorption.

**Low-oxalate produce**

- Cabbage, cauliflower, cucumber, zucchini, mango, melon, apples

**Hydration target**

- Produce **>2–2.5 L urine/day** unless restricted by heart/kidney disease.

**4. Safety Warning**

If you have **heart failure, cirrhosis, or advanced CKD**, fluid goals or potassium-rich diets may require modification — tell me if you have any of these so I can adjust safely.

**Bottom Line**

Because your sodium is *extremely* high, **the single most impactful action is eliminating processed and restaurant foods**. Most patients with your sodium level must reduce intake by **at least 60–70%** to hit guideline targets.

**Std + QB:**

Below is **general educational guidance**, not personal medical advice. Kidney stone prevention must be individualized, so **confirm all diet changes with your clinician or dietitian**, especially with your elevated urine sodium and urine calcium.

**🧂 If your urine sodium is 300 mmol/day, the *first* thing to reduce is salt (sodium) intake**

This level of sodium is *very* high. High sodium intake directly increases urinary calcium excretion—this is a major driver of **calcium oxalate stone recurrence**.

**Why sodium matters most**

For every ~100 mmol of sodium you lower in your diet, urinary calcium typically drops by ~20–40 mg/day.
A lower urine calcium → less calcium oxalate supersaturation → fewer stones.

**What to stop or sharply reduce first**

**1️⃣ High-sodium foods (your top priority)**

Your sodium intake is likely >4,000–5,000 mg/day. Target is ideally **<2,000 mg/day**.

**Biggest culprits to eliminate or restrict:**

- Restaurant and fast foods (major source of hidden sodium)
- Processed meats: bacon, sausage, deli meats, jerky
- Packaged soups, canned foods with added salt
- Chips, crackers, salted nuts
- Cheese (especially processed cheese)
- Frozen meals and prepared entrées
- Sauces: soy sauce, teriyaki, fish sauce, barbecue sauce, salad dressings
- Sports drinks, electrolyte powders with sodium

**How strict?**

**Very strict**, at least until your urine sodium comes down near target.
You should be reading labels and aiming for meals with **<400–500 mg sodium each**.

**2️⃣ High-oxalate foods (but *only after* correcting sodium)**

Your urine calcium is high, so even moderate oxalate intake becomes riskier.

Instead of eliminating oxalate across the board (not necessary), focus on the **top offenders**:

- Spinach (the worst) – avoid completely
- Almonds & nut butters – minimize
- Beets, rhubarb, Swiss chard
- Dark chocolate/cocoa
- Buckwheat, bran cereals
- Sweet potatoes
- Star fruit

**How strict?**

You don’t have to avoid *all* oxalates—just eliminate the worst foods listed above, **especially spinach and almonds**, until your urine parameters improve.

Always pair moderate-oxalate foods with **calcium-containing foods** (yogurt, milk, cheese) at meals, which binds oxalate in the gut.

**3️⃣ Ensure adequate calcium intake (do NOT restrict calcium)**

This surprises many people.

- Target **1,000–1,200 mg/day** from food
- Low calcium → more oxalate absorption → more stones
- Best sources: dairy, fortified plant milks, yogurt

**4️⃣ Protein: reduce excess animal protein**

High animal protein increases urine calcium and lowers citrate.

You don’t need to eliminate it—just moderate it:

- ~0.8–1.0 g/kg/day for most people
- Limit large portions of meat (keep servings palm-sized)
- Avoid high-sodium cured meats (double risk: sodium *and* protein)

**5️⃣ Increase fluid intake**

Goal: enough to make **≥2.5 liters of urine/day**.
This is the *most important overall* protection against stones.

**Summary of what to stop first and how strict to be**

**🚨 Top Priority (strict): Sodium reduction (<2,000 mg/day)**

This has the strongest effect on your elevated urine calcium.

**⚠️ Next Priority (moderate strictness): Eliminate highest-oxalate foods**

Spinach & almonds are the most important to stop.

**👍 Moderate Adjustments:**

- Normal calcium intake with meals
- Moderate animal protein
- High fluid intake

**Case13:**

**SA + QA:**

Below is an interpretation tailored to your metabolic profile (low **urine magnesium** and **urine citrate**) and based on EAU/CUA guideline-consistent dietary principles.

**1. Metabolic Diagnosis**

- **Hypomagnesuria** – Urine magnesium 1.2 mmol/day is low; magnesium normally complexes with oxalate and inhibits CaOx crystallization. Low intake or poor absorption is typical.
- **Hypocitraturia** – Urine citrate 1.0 mmol/day is low; citrate is a major inhibitor of calcium stone formation. Causes include low fruit/veg intake, high animal protein, metabolic acidosis, or low potassium intake.

Together, these deficiencies significantly reduce your natural urinary stone inhibitors.

**2. Key Dietary Modifications (Top Priorities)**

**1. Increase citrate-rich, potassium-rich fruits and vegetables**

Especially those shown to increase urinary citrate (lemon, lime, oranges, melons).

**2. Increase magnesium-rich foods (but not supplements unless medically indicated)**

Dietary magnesium reduces urinary oxalate absorption and promotes formation of soluble Mg-oxalate complexes.

**3. Maintain a high fluid intake (target ≈ 2–2.5 L urine/day)**

Ensures dilution of calcium and oxalate, crucial for CaOx stone prevention.

**3. Sample Menu Suggestions**

**Foods and drinks that *help prevent* more stones**

**Boosting citrate**

- **Daily lemon or lime water:**
  *½ cup lemon juice (fresh or bottled) mixed into water twice daily* → reliably raises urine citrate.
- **Citrus fruits:** oranges, mandarins, grapefruit.
- **Melons:** cantaloupe and honeydew (naturally alkalinizing).
- **High-potassium vegetables:** spinach (in moderation due to oxalate), zucchini, potatoes, carrots, squash.

**Boosting magnesium (low-oxalate options preferred)**

- **Nuts/seeds (moderation):** cashews, almonds, pumpkin seeds
  *(these contain some oxalate—fine in small portions with meals if dietary calcium is adequate).*
- **Legumes:** black beans, edamame, chickpeas.
- **Whole grains:** quinoa, oats, whole wheat.
- **Leafy greens low in oxalate:** kale, bok choy.
- **Dark chocolate (moderation).**

**Meals that support citrate + magnesium improvement**

- **Breakfast:** Oatmeal with almonds + a citrus fruit; lemon water.
- **Lunch:** Quinoa salad with chickpeas, zucchini, olive oil, lemon dressing.
- **Snack:** Greek yogurt (provides dietary calcium to bind oxalate) + melon slices.
- **Dinner:** Baked salmon, roasted squash or potatoes, steamed bok choy.
- **Bedtime option:** Glass of low-sodium sparkling water with lemon.

**Foods/drinks to *limit* because they reduce citrate or worsen CaOx risk**

- **High animal protein intake** (large portions of meat, especially red meat): increases acid load → lowers citrate.
- **High sodium foods** (processed foods, canned soups, fast food): increases urinary calcium and reduces citrate.
- **Cola beverages with phosphoric acid:** associated with lower urine citrate.
- **Very high-oxalate foods in excess:** spinach, beets, rhubarb, nuts in large quantity, almond flour, chocolate.
  *(These are not strictly forbidden—just keep portions moderate and consume with calcium-containing foods.)*

**4. Safety Warning**

You did not mention **chronic kidney disease**, **heart failure**, or use of medications affecting potassium.

- If you have **CKD stage ≥3**, be cautious with potassium-rich foods used to raise citrate—please let me know so I can tailor the plan.
- If you take **ACE inhibitors, ARBs, potassium-sparing diuretics**, or have restricted fluid allowances, your plan needs adjustment.

**Std + QA:**

Below is **general educational information**, not personal medical advice. Kidney-stone prevention must be individualized, so please review any changes with your clinician, especially if you have other medical conditions or take medications.

**Best Foods & Drinks to Help With Low Magnesium and Low Citrate (Calcium Oxalate Stones)**

Your urine profile suggests you may benefit from **higher dietary magnesium** and **citrate-rich foods/drinks**, both of which can reduce calcium oxalate stone formation.

**1. Increase Citrate-Rich Foods & Drinks**

Citrate binds calcium in urine and reduces stone risk. Low urine citrate is one of the most common correctable contributors to calcium oxalate stones.

**Helpful choices**

**Most effective:**

- **Lemon or lime juice** (highest natural citrate)
- **Homemade lemonade** or **citrate-enhanced water** (unsweetened or lightly sweetened)

**Other good options:**

- **Oranges / orange juice**
- **Grapefruit** (only *if safe* with your medications — grapefruit interacts with many drugs)
- **Melons** (cantaloupe, honeydew)

**Easy daily strategies**

- Add **2–4 tbsp of lemon or lime juice** to water 1–2× daily.
- Drink **citrus-infused water** throughout the day to raise citrate naturally.

**2. Boost Magnesium Intake**

Magnesium binds oxalate in the gut, lowering absorption and stone risk. Your urine magnesium is low, so increasing dietary intake may help.

**Magnesium-rich foods**

- **Leafy greens:** spinach*, Swiss chard, kale
- **Nuts:** almonds, cashews, peanuts
- **Seeds:** pumpkin, chia, flax
- **Whole grains:** brown rice, quinoa, whole wheat
- **Legumes:** black beans, lentils, soybeans
- **Avocado**
- **Dark chocolate** (in moderation)

**Important note:**

- *Spinach is very high in oxalate* despite being magnesium-rich. If you use spinach at all, keep portions small and pair with a **calcium source** (e.g., yogurt, milk, cheese) at the same meal to reduce oxalate absorption.

**3. Maintain Adequate Calcium Intake (do *not* restrict)**

Calcium at meals binds oxalate in the intestines and reduces stone formation.

**Best options**

- Dairy: milk, yogurt, cheese
- Calcium-fortified alternatives (soy, almond, oat milk)

**Target:** ~1,000–1,200 mg/day from food (supplements only if your clinician recommends them).

**4. Hydrate Strategically**

High fluid intake lowers stone recurrence by diluting urine.

**Tips**

- Aim for **2–2.5 liters of urine/day** (usually ~2.5–3 liters of fluid).
- Favor: **water**, **citrus-enhanced water**, **herbal teas**
- Limit: high-sugar drinks, cola (contains phosphoric acid), and excessive black tea (high oxalate).

**5. Limit Excess Oxalate — *But Don’t Over-restrict***

Instead of eliminating oxalate foods, the goal is **balance + pairing with calcium**.

**High-oxalate foods to watch (moderate, don’t avoid unless advised)**

- Spinach
- Almonds (limit to small portions)
- Rhubarb
- Beets
- Swiss chard
- Dark chocolate (small servings okay)
- Soy products

**Pair with calcium** at the same meal to reduce oxalate absorption.

**6. Reduce Sodium & Excess Animal Protein**

Both can lower urine citrate and increase calcium excretion.

**Targets**

- **Sodium:** <2,000 mg/day
- Prefer poultry or fish over large red-meat portions
- Use plant protein (beans, lentils, tofu) several times a week

**Sample Day for Citrate + Magnesium Support**

**Breakfast**

- Oatmeal with chia seeds and berries
- Greek yogurt (calcium)
- Water with lemon

**Lunch**

- Quinoa bowl with roasted vegetables, pumpkin seeds
- Small amount of cheese or yogurt-based dressing (calcium)

**Snack**

- Orange or citrus fruit
- Small handful of peanuts or cashews

**Dinner**

- Grilled chicken or fish
- Steamed broccoli (low oxalate)
- Brown rice
- Lime water or low-sugar lemonade

**SB + QB:**

Below is your structured, guideline-based interpretation and plan.

**1. Metabolic Diagnosis**

You have **hypomagnesuria (low urinary magnesium)** and **hypocitraturia (low urinary citrate)**—both increase calcium oxalate stone risk.

- **Low magnesium** reduces binding of oxalate in the gut → more oxalate absorbed → more CaOx crystallization.
- **Low citrate** reduces a key urinary inhibitor of stone formation.

**2. Key Dietary Modifications (Top Priorities)**

**1. Increase natural magnesium intake (food-first approach).**

Target foods with **150–400 mg magnesium/day total intake**, preferably from plant sources.

- Almonds, cashews, peanuts
- Pumpkin seeds, chia, flax
- Whole grains (oats, quinoa, brown rice)
- Legumes (black beans, soybeans, lentils)
- Dark leafy greens (spinach—OK *cooked in moderation*; raw spinach is too high in oxalate)

**2. Raise citrate intake through potassium-rich fruits/vegetables (unless you have CKD or are on potassium restrictions).**

- Citrus: lemons, limes, oranges
- Melons, kiwifruit, bananas
- Potatoes, sweet potatoes, squash
- Avocado
  (These increase urinary citrate and raise urine pH.)

****3. Avoid high-oxalate foods when magnesium and citrate are low, because you have fewer natural inhibitors.**

- Spinach (raw), rhubarb, beets, Swiss chard
- Almond flour or nut-based milks in large amounts
- Starfruit
- Excess dark chocolate
- High-dose vitamin C supplements (>500–1000 mg/day → increases oxalate)

**3. Sample Menu Suggestions**

**Breakfast**

**Good choices:**

- Oatmeal topped with chia/flaxseed and blueberries
- Greek yogurt with banana slices
- Scrambled eggs with low-oxalate vegetables (bell pepper, mushrooms)

**Avoid / limit:**

- Smoothies containing spinach or almond butter
- High-dose vitamin C powders

**Lunch**

**Good choices:**

- Quinoa bowl with grilled chicken, roasted sweet potatoes, zucchini
- Lentil or black bean soup (magnesium-rich)
- Lemon water with meals to boost citrate

**Avoid / limit:**

- Beet salads
- Large quantities of nuts in one sitting (almonds especially high in oxalate)

**Dinner**

**Good choices:**

- Grilled fish with brown rice and steamed broccoli
- Turkey stir-fry with bell peppers and carrots
- Baked potato (with skin) + olive oil + lemon

**Avoid / limit:**

- Spinach-based dishes
- Rhubarb, Swiss chard
- Excess cocoa or dark chocolate desserts

**Snack Ideas**

**Helpful:**

- Pumpkin seeds (magnesium-rich, moderate oxalate)
- Fresh fruit (citrus, melon, berries)
- Low-sodium popcorn

**Be careful with:**

- Almonds/almond flour energy bars (high oxalate if eaten frequently)

**4. Safety Warning**

If you have **CKD, heart failure, or are on medications affecting potassium**, avoid dramatically increasing potassium-rich foods or citrate supplements without medical supervision.
If none of these apply, the above plan is safe and guideline-concordant.

**Std + QB:**

Here’s **general, education-focused nutrition guidance** based on the pattern of **calcium oxalate stones with low urinary magnesium and low citrate**. This is *not* personalized medical advice, but it can help you understand typical strategies people use to reduce recurrence risk. Always review changes with your clinician.

**⭐ Key Goals for Your Stone Pattern**

1. **Increase citrate intake** → citrate binds calcium in urine and reduces crystal formation.
2. **Increase dietary magnesium** → magnesium complexes with oxalate, lowering calcium-oxalate crystal formation.
3. **Maintain normal calcium intake** → too little calcium *raises* oxalate absorption.
4. **Moderate—not eliminate—oxalate-rich foods**.
5. **Lower sodium** and **keep animal protein reasonable**, both of which worsen calcium excretion.

**✅ Foods to *Add* (Help With Magnesium + Citrate)**

**1. Citrate-rich foods**

Great because they raise urinary citrate.

- **Citrus fruits**: lemons, limes, oranges, clementines, grapefruits
- **Lemon or lime water**: many patients aim for regular intake throughout the day
- **Citrus-based marinades/salad dressings**

✔ Tip: Many clinicians recommend **adding lemon juice to water** (e.g., a few tablespoons per day), but amounts should be individualized.

**2. Magnesium-rich foods**

Magnesium reduces stone risk by binding oxalate in the gut.

- **Nuts & seeds**: almonds, pumpkin seeds, cashews
  - Note: some have moderate oxalate—portion control is key.
- **Whole grains**: brown rice, oats, whole-wheat bread
- **Legumes**: black beans, lentils, soybeans
- **Leafy greens**: kale, bok choy (avoid spinach—see below)
- **Avocado**
- **Dark chocolate (small portions)**
- **Mineral waters high in magnesium** (if you enjoy them)

✔ Tip: Spread magnesium foods across meals for steady intake.

**3. Adequate calcium with meals (VERY important)**

Dietary calcium binds oxalate in the intestine → less oxalate absorbed.

- Aim for **2–3 servings/day** of:
  - Dairy: milk, yogurt, cheese
  - Non-dairy fortified alternatives with calcium
  - Canned fish with bones
  - Calcium-set tofu

✔ The key is **calcium *with* higher-oxalate meals**.

**⚠ Foods to Be Careful With (Moderation, Not Elimination)**

**High-oxalate foods**

These aren’t forbidden, just better in small amounts *with calcium*.
Especially high sources:

- **Spinach (very high—best to limit)**
- **Rhubarb**
- **Beets**
- **Almonds, almond flour, almond milk**
- **Swiss chard**
- **Dark chocolate (moderate amounts okay)**
- **Potato skins**
- **Soy products (some forms)**
- **Black tea**

✔ If you eat them, pairing with yogurt/cheese or another calcium source helps reduce absorption.

**High-sodium foods**

Sodium increases urinary calcium.

- Fast food
- Packaged snacks
- Frozen meals
- Cured meats
- Canned soups (unless low sodium)

✔ Goal: Often **<2,000 mg/day sodium**, depending on your provider’s recommendations.

**Excess animal protein**

Too much animal protein reduces citrate and raises calcium excretion.

- Large portions of beef, poultry, pork
- High intake of eggs
- Processed meats

✔ Moderate portions (e.g., 3–4 oz per meal) and some plant-protein swaps help.

**💧 Hydration (Most Important Overall)**

- Target: **Urine output ~2–2.5 liters per day**
- Use citrus-infused water for citrate boost
- Spread water throughout the day rather than large, infrequent amounts

**🍽 Putting It All Together (Example Pattern)**

- **Breakfast:** yogurt with oats and berries
- **Lunch:** grain bowl with brown rice, chicken, kale, avocado; lemon vinaigrette
- **Snack:** small handful of nuts OR fruit with calcium-containing food
- **Dinner:** salmon, roasted vegetables, whole grains
- **Fluids throughout day:** water + lemon/lime, occasional magnesium-rich mineral water

**Case14:**

**SA + QA:**

Here is your personalized, guideline-based prevention plan:

**1. Metabolic Diagnosis**

Your 24-hour urine profile is *normal*, so this is classified as a **“stone former with no identifiable metabolic abnormality.”**
In such cases, recurrence risk is reduced mainly through **general dietary and fluid measures**, not targeted metabolic correction.

**2. Key Dietary Modifications (Top Priorities)**

**1. Increase Fluid Intake to Produce ≥2.5 L of Urine Daily**

- This is the *strongest* evidence-based intervention for preventing recurrence.
- Most adults need **3 L/day of fluid intake** to achieve this.

**2. Maintain Normal Dietary Calcium (1,000–1,200 mg/day)**

- Prefer dairy with meals.
- Avoid low-calcium diets, which increase intestinal oxalate absorption.

**3. Moderation of Sodium & Animal Protein**

- **Sodium target:** <2,000 mg/day
  (High sodium increases urinary calcium even when labs are normal.)
- **Animal protein:** Aim for 0.8–1.0 g/kg/day
  Avoid very high-protein diets (keto, carnivore, heavy grilling/meat-dominant diets).

**3. Sample Menu Suggestions**

**✔ Foods to Focus On**

- **Hydration:** Water, lemon water, sparkling water, herbal tea.
  Add **citrus** (lemon, lime) to increase natural citrate intake.
- **Calcium sources:**
  Milk, yogurt, kefir, fortified plant milks, cheese (in moderation).
- **Low-oxalate fruits/vegetables:**
  Bananas, melons, mango, cauliflower, cabbage, zucchini, carrots.
- **Plant-forward proteins:**
  Lentils, chickpeas, tofu, eggs, fish.
- **Whole grains:**
  Oats, barley, brown rice.

**✖ Foods to Limit or Avoid**

- **High-oxalate foods** (not banned, just avoid large daily quantities):
  Spinach, almonds, beets, rhubarb, Swiss chard, peanuts.
  *If eaten, pair with calcium-containing foods.*
- **High-sodium foods:**
  Fast food, deli meats, canned soups, chips, ramen seasoning packets.
- **Excess animal protein:**
  Large portions of red meat, high-protein diets, protein shakes >1 serving/day.

**4. Safety Warning**

You have no comorbidities listed, so **no major restrictions** are required.
However:

- If you engage in heavy exercise or live in a hot climate, you may need **more fluids** to maintain 2.5 L urine output.
- Avoid overhydration only if you develop heart, liver, or kidney disease in the future.

**Std + QA:**

Here are **evidence-based, general prevention guidelines** for someone like you — a 30-year-old man with a **single calcium oxalate stone** and an otherwise **normal metabolic evaluation**. These are standard recommendations used when no specific metabolic abnormality is found.

**✅ 1. Increase Fluid Intake (Most Important Step)**

- Aim for **2.5–3 liters of urine output per day**.
- This usually requires **3 liters (about 100 oz) of fluid intake daily**.
- Water should make up most of your fluid intake.
- You can include:
  - Water with lemon/lime (citrate helps prevent stones)
  - Herbal tea
  - Diluted fruit juices
- Avoid **large amounts of soda, sweetened drinks, or energy drinks**.

**Why it matters:** Dilution decreases crystal formation and lowers recurrence risk by up to **50%**.

**✅ 2. Maintain Normal Dietary Calcium**

This surprises many people — **do NOT restrict calcium** unless instructed by a specialist.

- Target **1,000–1,200 mg/day** from food, not supplements.
- Good sources:
  - Dairy: milk, yogurt, cheese
  - Calcium-fortified alternatives (soy, almond milk)
  - Leafy greens (except spinach, which is high oxalate)

**Why:** Dietary calcium binds oxalate in the gut, reducing oxalate absorption and lowering stone risk. Low calcium **increases** stone formation.

**✅ 3. Limit High-Oxalate Foods (But Don’t Avoid All Oxalate)**

You do *not* need a low-oxalate diet overall — just avoid excess from major offenders:

**High-oxalate foods to moderate:**

- Spinach (one of the highest)
- Almonds, cashews, peanuts
- Beets
- Rhubarb
- Swiss chard
- Soy products (esp. tofu)
- Wheat bran
- Dark chocolate
- Sweet potatoes
- Black tea (in large amounts)

**Tips:**

- Eat high-oxalate foods **with calcium-containing foods** (e.g., spinach + cheese), which reduces absorption.
- Normal portions of most vegetables are safe.

**✅ 4. Reduce Sodium Intake**

High sodium increases urinary calcium.

- Aim for **< 2,300 mg/day**, ideally closer to **1,500 mg/day** if possible.
- Practical strategies:
  - Limit processed/packaged foods
  - Choose “low sodium” labeled items
  - Cook at home when possible

**✅ 5. Moderate Animal Protein**

Too much animal protein increases urinary calcium and uric acid and lowers citrate.

- Limit to **1–2 servings per day**:
  - Meat, poultry, fish, shellfish
- Use plant proteins more often (beans, lentils—just avoid very high oxalate ones in excess)

**✅ 6. Increase Fruits and Vegetables (Especially Citrate-Rich Foods)**

Citrate inhibits stone formation.

Good options:

- Lemons, limes
- Oranges, grapefruits
- Melons
- Most vegetables other than spinach/beets/chard

You can also add **lemon juice to water** daily.

**✅ 7. Maintain a Healthy Weight**

Obesity is associated with stone formation, so aim for regular exercise and balanced nutrition.

**✅ 8. Avoid Vitamin C Supplements**

- Vitamin C > 500 mg/day can convert to oxalate in the body.
- Food-based vitamin C is fine.

**Quick Summary**

| **Goal** | **Target** |
| --- | --- |
| Fluids | **2.5–3 L urine/day** (~3 L fluid intake) |
| Calcium | **1,000–1,200 mg/day** from food |
| Sodium | **<2,300 mg/day** |
| Protein | Moderate animal protein |
| Oxalate | Avoid excess from high-oxalate foods |
| Citrate | Increase via fruits/vegetables & lemon water |

**SB + QB:**

Below is a simplified but *evidence-based* prevention plan for someone with **a single calcium oxalate stone and an entirely normal 24-hour urine profile**.
(When no metabolic abnormality is present, the EAU & CUA classify patients as *low-risk stone formers*, and the goal is general lifestyle-level prevention—not aggressive metabolic therapy.)

**1. Metabolic Diagnosis**

You have **no identifiable metabolic abnormality** on 24-hour urine evaluation.
This is consistent with **idiopathic calcium oxalate stone formation**—meaning the stone likely resulted from modifiable lifestyle factors such as hydration, sodium intake, or low dietary calcium *at the time* of stone formation, even if labs are now normal.

**2. Key Dietary Modifications (Top 3 Priorities)**

These are the *highest-yield*, simplest habits proven to reduce recurrence:

**1. Increase fluid intake to achieve ≥2.0–2.5 L urine output/day**

- This is the strongest evidence-based intervention for all stone formers.
- Practical cue: Your urine should be very pale yellow most of the day.

**2. Maintain normal calcium intake (1000–1200 mg/day) while limiting oxalate “bombs”**

- Adequate dietary calcium **reduces** oxalate absorption → fewer stones.
- Avoid *unnecessary* calcium restriction.

**3. Reduce excessive sodium & animal protein intake**

Even if your current urine sodium/calcium are normal, these are modifiable drivers of stone formation over time.

- Sodium target: **≤2 g/day sodium** (≈5 g salt).
- Animal protein: Keep to **moderate portions** (1–2 palm-sized servings/day).

**3. Sample Menu Suggestions**

**Foods to Favor**

- **Hydration:** Water, herbal teas, sparkling water, lemon water (citrate source).
- **Calcium sources:**
  - Yogurt, kefir
  - Milk or fortified plant milks
  - Cheese (moderation due to salt)
- **Low-oxalate fruits & vegetables:**
  - Apples, bananas, melons, berries
  - Cauliflower, cabbage, cucumbers, zucchini, mushrooms
- **Whole grains** instead of refined grains.
- **Plant proteins:** lentils, beans, tofu (moderation but encouraged).
- **Healthy proteins:** fish, poultry, eggs.

**Foods to Limit—but not eliminate**

(These foods are high in oxalate; the goal is simply not to consume them in large daily amounts.)

- Spinach
- Beets
- Almonds & nut butters
- Rhubarb
- Swiss chard
- Dark chocolate
- Wheat bran
- Sweet potatoes

If you *do* eat high-oxalate foods occasionally, pair them with a calcium-containing food (e.g., yogurt with a spinach-containing meal) to bind oxalate in the gut.

**Drink choices to adjust**

- **Limit:** Cola, black tea in excess, large amounts of coffee (>3–4 cups), grapefruit juice.
- **Alcohol:** Moderate intake is acceptable if hydration is adequate.

**4. Safety Warning**

Because your labs and history show no comorbidities (CKD, heart failure, or electrolyte disorders), there are **no specific safety restrictions** on fluid or potassium-containing foods.
If in the future you develop kidney dysfunction or are prescribed diuretics, fluid and potassium recommendations may need adjustment.

**Std + QB:**

Here are **simple, high-yield, evidence-based habits** that help prevent **calcium oxalate kidney stone recurrence**—even when your metabolic workup is normal. These are the *universal* prevention strategies that benefit almost everyone with a prior stone and don’t require complicated tracking.

**✅ 1. Drink enough fluids (the #1 habit)**

Aim for **2.5–3 liters of urine per day**, which usually requires drinking **3 liters (about 100 oz) of fluid daily**.

**Tips that make this easier:**

- Keep a water bottle with you all day
- Drink a glass of water before each meal
- Include some citrus drinks (lemon water, lime water) for natural citrate
- More fluids on hot/sweaty days

**✅ 2. Get normal—not low—calcium in your diet**

Low calcium *increases* oxalate absorption and raises stone risk.

Target: **1,000–1,200 mg calcium/day** from food.

Best choices:

- Dairy (milk, yogurt, cheese)
- Calcium-fortified plant milks
- If eating high-oxalate foods, pair them with calcium (e.g., spinach + cheese, nuts + yogurt)

**Avoid taking large calcium supplements unless advised by a clinician.**

**✅ 3. Moderate oxalate intake (without avoiding everything)**

You don’t need a strict oxalate diet since your urine tests were normal, but it helps to be aware of **very high-oxalate foods** and avoid extreme overconsumption.

Highest-oxalate foods to moderate:

- Spinach
- Almonds & almond flour
- Beets
- Swiss chard
- Rhubarb
- Cashews
- Peanut butter (large amounts)
- Wheat bran
- Dark chocolate (large amounts)

You don’t need to eliminate them—just **don’t eat them daily in large quantities**.

**✅ 4. Reduce sodium**

Why? High sodium increases calcium in the urine, raising stone risk.

Target: **<2,300 mg/day**, ideally closer to **1,500 mg/day**.

Practical tips:

- Avoid salty snacks, canned soups, fast food
- Check food labels
- Cook more at home
- Use herbs/spices instead of salt

**✅ 5. Eat a more plant-forward, balanced diet**

Patterns that help:

- **More fruits and vegetables** → increases citrate
- **Moderate animal protein**, especially red meat
- **Limit very sugary beverages** (soda raises stone risk)

A Mediterranean-style diet is ideal.

**✅ 6. Get enough citrate (natural stone inhibitor)**

Ways to increase citrate:

- Lemon or lime juice daily (approx. 4 oz lemon juice in water)
- Eating lots of fruits + vegetables
- Avoiding excessive animal protein

Citrate binds calcium and reduces stone formation.

**⭐ Putting it all together: the “Big Three” priorities**

If you only focus on **three habits**, make them these:

1. **Drink enough to produce 2.5–3 L urine/day**
2. **Keep sodium low and calcium normal**
3. **Eat a plant-forward diet with citrus and avoid huge amounts of high-oxalate foods**

These alone significantly reduce recurrence risk—even with normal metabolic testing.

**Case15:**

**SA + QA:**

Below is your structured, guideline-aligned interpretation and plan.

**1. Metabolic Diagnosis**

The patient has **dietary (protein-driven) hypercalciuria**, most likely worsened by **high animal protein intake**, as suggested by elevated urine sulfate. This pattern is typical in calcium oxalate stone formers: excess animal protein increases calciuria, lowers urinary citrate, and increases acid load.

Because you also have **obesity**, the metabolic milieu increases stone risk further (low urine pH, higher uric acid load).

**2. Key Dietary Modifications (Top Priorities)**

**1) Reduce animal protein to guideline targets**

- Aim for **0.8–1.0 g/kg/day of protein**, primarily from **plant and dairy sources** rather than meat.
- Limit **red meat, poultry, eggs, and fish** to **1 serving per meal max**; avoid >2 servings/day.
- Target: **Lower urine sulfate → lower acid load → lower urine calcium + higher citrate**.

**2) Reduce sodium intake to <2 g/day (≈5 g salt/day)**

- Sodium strongly increases urinary calcium excretion.
- Reducing sodium can lower urine calcium by **20–25%**.

**3) Maintain normal dietary calcium (1000–1200 mg/day)**

- Essential to bind oxalate in the gut.
- Prefer calcium from **meals** (dairy or fortified alternatives).
- Avoid restricting calcium, which **increases urinary oxalate**.

**3. Sample Menu Suggestions**

**Foods to Prioritize**

- **Protein:**
  - Plant proteins: lentils, chickpeas, tofu, edamame.
  - Moderate dairy: yogurt, low-salt cheese, milk (binds oxalate).
- **Fruits & Vegetables:**
  - Citrus (lemons, oranges) for citrate.
  - Low-oxalate greens: kale, bok choy, cabbage.
- **Carbohydrates:**
  - Brown rice, whole grains, quinoa.
- **Fats:**
  - Olive oil, nuts in moderate portions (mind oxalate in almonds; choose cashews/pecans instead).

**Foods to Limit**

- **High-animal-protein foods:**
  - Beef, sausages, bacon, large portions of poultry, high-protein diets (keto/Atkins).
- **High-sodium foods:**
  - Processed meats, canned soups, restaurant foods, pickles, chips.
- **High-oxalate foods in excess:**
  - Spinach, almonds, beets, rhubarb, Swiss chard.
- **Vitamin C supplements >200 mg/day** (raises oxalate).

**1-Day Sample Menu**

**Breakfast:**

- Greek yogurt + berries + oats
- Lemon water

**Lunch:**

- Lentil soup (low sodium)
- Whole-grain bread
- Side salad (kale, cucumber, lemon vinaigrette)

**Snack:**

- Pear or apple
- Small handful of cashews

**Dinner:**

- Grilled tofu or small portion of fish (3–4 oz)
- Quinoa
- Steamed broccoli
- Glass of milk with the meal to bind oxalate

**4. Safety Warning**

- If you have **hypertension, heart failure, or CKD**, discuss **fluid goals and potassium-rich foods** with your clinician before increasing fluids or fruits/vegetables substantially.
- Obesity increases risk of low urine pH; weight loss should be gradual—**avoid crash diets** (they increase acid load and stone risk).

**Std + QA:**

Below is **general educational information**, not personal medical advice. Kidney-stone prevention must be individualized by your own clinician, especially since you have obesity and documented metabolic risk factors.

**🍽️ Dietary Changes Likely to Help Most**

For a **50-year-old man with calcium oxalate stones, obesity, high urine calcium (8.0 mmol/day), and high urine sulfate (marker of high animal-protein intake)**, the most impactful changes target **lowering urinary calcium**, **reducing acid/protein load**, and **improving urine volume and citrate**.

**1️⃣ Reduce Animal Protein (Major Priority)**

High animal-protein intake increases:

- Urine calcium
- Urine oxalate
- Acid load → lowers urine citrate (a natural stone inhibitor)

High urine sulfate confirms this pattern.

**Practical steps**

- Limit animal protein to ~0.8–1.0 g/kg/day (ask your clinician for a personal target).
- Replace some **red meat, poultry, and eggs** with:
  - Beans, lentils
  - Tofu/soy products
  - Nuts (in moderation—see below for oxalate)
  - Low-fat dairy for protein instead of meat

**2️⃣ Maintain Normal Dietary Calcium (1,000–1,200 mg/day)**

This is crucial despite having calcium stones.

Why?
**Adequate calcium at meals binds oxalate in the gut**, reducing absorption and lowering urine oxalate.

Best options:

- Low-fat milk or yogurt
- Calcium-rich greens (kale, broccoli)
- Fortified plant milks (not high-oxalate greens like spinach)

Avoid **calcium restriction**, which paradoxically increases stone risk.

**3️⃣ Lower Sodium Intake**

High sodium intake increases urinary calcium losses.

Target:

- **≤2 grams of sodium per day** (≈5 g salt).
  This often means limiting:
- Restaurant/processed foods
- Cured meats
- Salty snacks, canned soups
- Fast food

Pairing **low sodium + lower animal protein** is one of the strongest ways to reduce urine calcium.

**4️⃣ Increase Fluids (Critical Step)**

Goal:

- Urine output ≥ **2–2.5 L/day**
- Pale/yellow-clear urine

Helpful choices:

- Water
- Citrus-based drinks (e.g., lemon water) → boosts citrate
- Avoid excessive soda, especially cola.

**5️⃣ Address Weight & Metabolic Factors**

Obesity increases stone risk by:

- Increasing insulin resistance → more acidic urine
- Lowering urine citrate

Helpful dietary patterns:

- **Mediterranean-style diet**
- **DASH diet**

Both reduce stone risk and support weight loss:

- High fruits/vegetables (raise urine citrate)
- Lower in animal protein
- Lower sodium
- Balanced calcium intake

**6️⃣ Moderate High-Oxalate Foods (but do not eliminate all oxalate)**

Oxalate reduction is usually *less impactful* than the protein/sodium adjustments above, but still useful.

Limit:

- Spinach
- Almonds & nut butters
- Beets
- Rhubarb
- Swiss chard
- Black tea (if consumed in large amounts)

If eating oxalate-rich foods → pair them with calcium-containing foods.

**Summary: What Helps Most for You**

Given your profile (high urine calcium + high sulfate + obesity), the biggest impact will come from:

**⭐ Top Priority Changes**

1. **Reduce animal protein intake**
2. **Lower sodium intake**
3. **Maintain normal dietary calcium (not low calcium)**
4. **Increase fluids to raise urine volume**

**Additional Useful Adjustments**

5. **Boost fruits/vegetables / citrate intake**
6. **Limit high-oxalate foods**
7. **Adopt a Mediterranean or DASH-style diet for weight and metabolic health**

**SB + QB:**

Below is a **guideline-based, highly targeted plan** for your situation as a **calcium oxalate stone former with elevated urine calcium (8.0 mmol/day) and high sulfate (marker of high animal-protein load).**

**1. Metabolic Diagnosis**

You have **diet-induced hypercalciuria** and **excessive acid load from high animal-protein intake**, reflected by:

- **Urine calcium: 8.0 mmol/day** (above typical threshold of concern ≈ 6.2–7.5 mmol/day).
- **High sulfate:** indicates high intake of sulfur-containing amino acids (methionine/cysteine → animal protein), which increases acid load → increases urinary calcium → increases stone risk.

This pattern is consistent with **protein-driven hypercalciuria** rather than calcium overconsumption.

**2. Key Dietary Modifications (Top Priorities)**

**Priority 1 — Reduce animal protein to guideline targets**

Aim for:

- **0.8–1.0 g protein/kg/day** (example: 60–75 g/day for a 75-kg person).
- Limit: **meat, poultry, fish, eggs** to **1–2 servings/day max**.
- Replace with: plant proteins (lentils, tofu, beans), which do **not** increase sulfate or urinary calcium.

**Priority 2 — Reduce sodium to <2,000 mg/day**

High sodium dramatically increases urinary calcium excretion.

- Goal: **<2 g salt/day sodium (~5 g salt)**.
- Cut: deli meats, soups, fast food, seasoning blends, sauces.

**Priority 3 — Maintain normal dietary calcium (1,000–1,200 mg/day)**

Do **not** restrict calcium.

- Adequate calcium binds oxalate in the gut → **reduces oxalate absorption** and lowers stone risk.
- Obtain calcium from food (dairy, fortified plant milks), **not supplements** unless medically required.

**3. Sample Menu Suggestions**

**✔ Foods to Prefer**

**Protein**

- 1 palm-size serving (85–100 g) of chicken/fish OR
- 2 eggs OR
- ½ cup tofu or legumes
  (Choose **1–2 of these per day**, total 60–75 g protein.)

**Calcium sources (keep normal, not low):**

- 1 cup low-fat milk or fortified soy milk
- ¾ cup yogurt
- 30 g cheese
  (Choose 2–3 servings daily.)

**Plant-forward items that lower acid load & stone risk**

- Beans, lentils, tofu
- Fruits & vegetables (alkali-producing → increases urine citrate)
- Whole grains

**Hydration**

- Target urine output ≥2–2.5 L/day
- Citrus intake (lemon/lime) for natural citrate

**✘ Foods to Limit or Avoid**

**High-sulfur animal proteins (greatest impact on sulfate & hypercalciuria):**

- Red meat (beef, lamb, pork)
- Large portions of poultry
- Large portions of fish (esp. 200–300 g servings)
- High-protein diets (keto, carnivore, bodybuilding diets)

**High-sodium items**

- Processed meats (ham, bacon, sausages)
- Fast food, packaged meals
- Chips, pretzels, salted nuts
- Soy sauce, bouillon cubes, seasoning packets

**Excess oxalate (still relevant)**

- Spinach, almonds, beets, rhubarb — OK occasionally but pair with calcium at the same meal.

**Example Daily Meal Pattern (Balanced for Stone Prevention)**

**Breakfast**

- Oatmeal with fortified soy milk
- Berries or banana
- 1 boiled egg (optional)

**Lunch**

- Lentil or chickpea salad with olive oil + lemon
- Whole-grain pita
- Yogurt cup for calcium

**Dinner**

- Palm-size portion (85–100 g) baked chicken or fish
- Steamed vegetables
- Brown rice
- Glass of milk or calcium-fortified alternative

**Snacks**

- Fruit, vegetables, unsalted nuts (small handful)
- Cottage cheese or yogurt if additional calcium needed

**4. Safety Warning**

If you have **kidney disease, heart failure, or are on potassium-altering medications**, use caution with:

- High-potassium foods (fruits/vegetables)
- High fluid intake

If any of these apply, let me know so I can tailor your plan safely.

**Std + QB:**

Below is **general, evidence-based dietary guidance** for reducing recurrence of **calcium oxalate kidney stones**, especially when urine tests suggest **high protein intake, elevated urinary calcium (8.0 mmol/day), and high sulfate** (a marker of animal-protein load).
This is **not a substitute for personal medical care**, but it should help you understand how to adjust your diet safely.

**✅ What Your Results Suggest**

**High urinary sulfate** + **high urinary calcium** commonly indicate **excess animal-protein intake** (meat, poultry, fish, eggs).
Animal protein can:

- Increase urinary calcium
- Lower urinary citrate
- Increase uric acid and acid load
- Increase kidney-stone risk overall

So your diet strategy should **moderate animal protein**, **increase plant foods**, and **not overly restrict calcium**.

**🍗 1. Meat & Animal Protein: How Much and What Kind?**

**Recommended limit**

For stone prevention, aim for:
**0.8–1.0 g of protein per kg body weight per day**,
*with no more than half of that from animal sources.*

**Examples (daily limits):**

- If you weigh 70 kg → target **56–70 g protein/day**
- Animal protein allowance in that total: **~30–35 g/day**

**What this looks like in portions**

Meat/fish/poultry portions to aim for:

- **1 small portion per day** or
- **3–4 modest portions per week**

A “modest” portion = **75–90 g (3 oz) cooked**, about the size of your palm.

**Better animal-protein choices**

- **Fish** (especially non-predatory kinds)
- **Skinless poultry**
- **Egg whites (low sulfur amino acids)**

**Reduce or avoid**

These drive up sulfate, calcium, and stone risk:

- Large servings of **red meat**
- Organ meats
- Processed meats (very acid-forming)
- High-protein diets (keto, carnivore)

**🌱 2. Increase Plant Protein Instead**

Plant protein produces **less acid load** and **lower urine calcium**.

Good replacements:

- Beans and lentils
- Tofu and tempeh
- Nuts (in moderation—handful/day)
- Whole grains like quinoa

These are much gentler on kidney-stone risk.

**🧂 3. Reduce Sodium to Help Lower Urinary Calcium**

When salt intake is high, the kidney dumps more calcium into the urine.

Aim for:

- **≤ 2,000 mg sodium/day**
- Avoid processed foods, restaurant meals, chips, salted snacks.

This step alone can **significantly reduce urinary calcium**.

**🥛 4. Keep Normal Dietary Calcium (Do NOT Reduce It)**

People often think they should cut calcium—but that increases stone risk.

Aim for:

- **1,000–1,200 mg/day from food**, not supplements
- Good sources: dairy, fortified alternatives, yogurt, leafy greens

Calcium eaten with meals **binds oxalate in the gut**, lowering stone formation.

**🥤 5. Hydration Targets**

- **2.5–3 L fluid/day**
- Aim for **urine that stays pale yellow** throughout the day

**🥗 6. Other Dietary Adjustments**

**Moderate high-oxalate foods**

Do *not* eliminate, but pair with calcium at meals:

- Spinach
- Almonds
- Chocolate
- Beets
- Rhubarb

**Increase citrate-rich foods**

Natural stone inhibitors:

- Lemons & limes
- Oranges
- Fruits & vegetables in general

**Limit added sugars and excessive vitamin C supplements**

Vitamin C >1,000 mg/day can increase oxalate.

**🧭 7. Summary: Your Action Plan**

**Reduce**

- Large meat portions
- Red and processed meats
- Sodium
- Excess animal protein, especially multiple servings per day

**Aim for**

- 1 modest meat/fish portion per day or less
- Most protein from **plants**
- 1,000–1,200 mg calcium daily
- Good hydration
- Plenty of fruits/vegetables
- Citrate-rich drinks (water with lemon)

**Case16:**

**SA + QA:**

Below is diet guidance tailored to **recurrent calcium phosphate stones in the setting of primary hyperparathyroidism (PHPT)**.
Because PHPT causes **endogenous overproduction of calcium**, the metabolic logic and dietary priorities differ from standard calcium-oxalate prevention.

**1. Metabolic Diagnosis**

**Primary hyperparathyroidism–driven hypercalcemia and hypercalciuria**
Your **urine calcium of 12 mmol/day (≈480 mg/day)** plus **elevated serum calcium** indicate that your high urinary calcium is **not primarily dietary**, but driven by **PTH-mediated bone resorption and renal calcium leak**.
This predisposes you to **calcium phosphate stones** due to:

- **High urine calcium**, and
- **Higher urine pH** (common in CaP formers), promoting calcium-phosphate supersaturation.

Diet can *modestly* help, but **definitive treatment is correction of hyperparathyroidism** (parathyroid surgery per EAU/CUA guidelines).

**2. Key Dietary Modifications (Top 3 Priorities)**

**1. Restrict Sodium to <2 g/day (≈5 g salt/day)**

High sodium intake increases urinary calcium excretion.
Even with PHPT, lowering sodium **reduces renal calcium wasting**.

**2. Maintain *normal* dietary calcium (1000–1200 mg/day) from food**

Do **NOT** restrict dietary calcium.
Low calcium intake increases intestinal oxalate absorption and bone resorption, worsening hypercalcemia and hypercalciuria in PHPT.
→ Avoid calcium supplements unless prescribed.

**3. Moderate animal protein**

Aim for **0.8–1.0 g/kg/day**.
High animal protein raises calcium excretion and acid load, which can worsen bone turnover in PHPT.

Optional supportive steps:

- Increase fruits/vegetables for natural alkali, but avoid excessive bicarbonate-raising supplements that may push urine pH too high in CaP stone formers.
- Ensure **adequate—but not excessive—hydration** (≈2–2.5 L urine/day).

**3. Sample Menu Suggestions**

**Foods to Prioritize**

- **Calcium from food**: milk, yogurt, kefir, fortified plant milks, cheese (in moderate portions).
- **Low-sodium choices**: fresh meats, unsalted nuts, low-sodium breads, fresh vegetables.
- **Plant-forward proteins**: lentils, beans, tofu—less calciuric than animal proteins.
- **Produce**: berries, citrus, bananas, melons, leafy greens (normal portions).
- **Fluids**: water, herbal teas.

**Foods to Limit**

- **High-salt foods**: processed meats, canned soups, restaurant meals, pickles, snack chips.
- **High-animal-protein items**: large red-meat portions (>120–150 g/meal), high-protein fad diets.
- **Calcium supplements** or antacids containing calcium (unless directed by your physician).
- **Top-alkalizing supplements** (potassium citrate, bicarbonate) must be used cautiously because too-high urine pH favors **calcium phosphate** stones.

**Example Day**

**Breakfast:**

- Low-sodium oatmeal with blueberries + ½ cup low-fat milk
- Herbal tea

**Lunch:**

- Lentil soup (homemade, low sodium)
- Mixed green salad with olive oil
- Unsalted almonds (small handful)

**Dinner:**

- Grilled salmon or tofu
- Steamed vegetables
- Brown rice
- Water with lemon

**Snacks:** fresh fruit, yogurt (small cup), raw veggies

**4. Safety Warning**

Because you have **primary hyperparathyroidism with elevated serum calcium**, you must observe the following:

- **High fluid intake is safe**, but avoid dehydration; however, if you have **heart failure or CKD**, fluid recommendations must be individualized.
- **Avoid calcium supplements** unless your endocrinologist directs otherwise.
- Treatment of PHPT (usually parathyroidectomy) is the **only intervention that corrects the root cause** of hypercalciuria and is strongly recommended in recurrent stone formers in EAU/CUA guidelines.
- Any citrate supplementation must be **cautiously considered**, as raising urine pH further may worsen calcium phosphate stone risk.

**Std + QA:**

Below is **general educational guidance** about diet in the context of **recurrent calcium-phosphate kidney stones with primary hyperparathyroidism (PHPT)**. This **does not substitute for individualized medical care**, especially because PHPT alters calcium handling and usually requires targeted treatment (often surgical).

**💡 Key Principle**

**Diet alone cannot correct stone risk when primary hyperparathyroidism is present.**
Because PHPT raises **serum calcium** and **urine calcium**, stone formation is driven mainly by the hormonal abnormality rather than diet.
Still, diet can help *reduce additional risks*.

**✔️ Appropriate Diet Advice in This Setting**

**1. Do *not* restrict dietary calcium**

It may seem intuitive, but restricting calcium actually **increases stone risk** by:

- Raising intestinal absorption of **oxalate**
- Potentially worsening bone health in PHPT

👉 Aim for **normal calcium intake** from food (≈ 1,000–1,200 mg/day).
Prefer **dietary sources** such as dairy, fortified plant milks, leafy greens, almonds, etc.
Avoid calcium supplements unless specifically advised by your clinician.

**2. Limit sodium intake (very important)**

High sodium → increased calcium excretion in urine.
With your high urine calcium (12 mmol/day), **sodium restriction is one of the most effective dietary strategies**.

**Target:**

- ≤ 1,500–2,000 mg sodium/day
- Avoid processed foods, restaurant meals, salty snacks, cured meats.

Every 100 mEq drop in sodium intake can reduce urine calcium by ~50 mg (≈1.25 mmol).

**3. Moderate animal protein**

Excess animal protein:

- Increases calcium excretion
- Reduces citrate (a natural inhibitor of stones)

You don’t need to avoid it entirely—just keep portions moderate:

- Choose more plant-based proteins
- Limit large servings of meat, especially daily

**4. Stay well hydrated**

Aim for enough fluid to produce **>2–2.5 liters of urine/day**.
This dilutes stone-forming minerals.

Water, herbal tea, and citrus-based drinks are good choices. Lemon water can help raise citrate.

**5. Avoid very alkaline urine unless directed**

Calcium-phosphate stones form more readily in **high urinary pH**.
Some alkalinizing foods or supplements (like potassium citrate) can raise pH further—these may or may not be appropriate in your case.

👉 Only adjust urine pH under direct clinician supervision.

**6. Vitamin D and supplements**

In PHPT:

- Vitamin D deficiency is common
- But supplementation must be **careful** because it can increase calcium

Never start vitamin D, calcium supplements, or bone-health agents without physician guidance.

**✔️ Special Note: Primary Hyperparathyroidism**

Because your **serum calcium is high** and **urinary calcium is high**, diet changes alone cannot fully resolve stone risk.
**Definitive treatment of PHPT (often parathyroid surgery) is the most effective way** to reduce calcium levels and prevent recurrent stones.

**Summary of Diet Advice**

| **Goal** | **Recommendation** |
| --- | --- |
| Reduce urine calcium | **Low sodium**, **moderate animal protein**, **adequate hydration** |
| Prevent worsening stone risk | **Normal—not low—dietary calcium** |
| Avoid raising urine pH further | Be cautious with alkaline supplements (e.g., citrate) |
| Address underlying cause | PHPT evaluation and potential surgery |

**SB + QB:**

Below is a safe, guideline-consistent interpretation **without revealing internal reasoning**.

**1. Metabolic Diagnosis**

The patient has **severe hypercalciuria secondary to Primary Hyperparathyroidism (PHPT)**.

- In PHPT, **excess PTH increases bone resorption and renal calcium filtration**, driving **very high urine calcium (12 mmol/day)** and **serum hypercalcemia**.
- **Diet cannot correct PHPT-related hypercalcemia or hypercalciuria.**
- Calcium phosphate stones are common in PHPT because high calcium and alkaline urine promote hydroxyapatite/brushite formation.

**This condition requires definitive management of PHPT (often parathyroid surgery), not dietary correction alone.**

**2. Key Dietary Modifications**

Diet **cannot normalize calcium excretion in PHPT**, but it *can* reduce stone risk while awaiting treatment or if surgery is delayed.

**Top 3 Priorities**

1. **Do NOT restrict dietary calcium**
   - Restriction increases intestinal oxalate absorption → increases CaOx risk after surgery.
   - Maintain **1,000–1,200 mg/day** from food (not supplements unless medically required).
2. **Aggressively reduce sodium intake (<2 g sodium/day = <5 g salt/day)**
   - Sodium restriction is the single most effective diet change for lowering urine calcium **even in PHPT** because Na and Ca share proximal tubular handling.
3. **Moderate animal protein (≤0.8–1.0 g/kg/day)**
   - Reduces acid load → increases citrate → improves urine chemistry.
   - High protein intake worsens hypercalciuria and lowers citrate.

**3. Sample Menu Suggestions**

**Foods to choose (stone-protective)**

- **Calcium-rich foods with meals** (to bind oxalate):
  - Yogurt, milk, fortified plant milks, tofu set with calcium, cheese in moderation.
- **Low-sodium staples:**
  - Fresh poultry/fish, legumes, quinoa, unsalted nuts, fresh vegetables & fruits.
  - Homemade soups rather than canned.
- **Hydration:**
  - 2.5–3 L/day *if no heart/kidney failure*.
  - Include water, citrus water, herbal teas; citrate is beneficial.

**Foods to limit**

- **High-sodium items:**
  - Restaurant meals, deli meats, canned soups, pickles, chips, sauces (soy sauce, teriyaki, BBQ).
- **Excess animal protein:**
  - Large servings of beef, pork, chicken, or fish (>150 g per meal).
- **Calcium supplements unless prescribed:**
  - Supplements can worsen hypercalcemia in PHPT.

**Example Daily Plan**

- **Breakfast:** Oatmeal with milk or fortified soy milk, berries, handful of almonds (unsalted).
- **Lunch:** Lentil or chickpea salad with olive oil/lemon dressing; low-salt whole grain crackers.
- **Dinner:** Grilled salmon or tofu, quinoa, steamed vegetables.
- **Snacks:** Yogurt, fruit, unsalted nuts.
- **Avoid:** Restaurant burritos, ramen, processed meats, salty cheeses, protein shakes.

**4. Safety Warning**

- **In Primary Hyperparathyroidism, diet alone cannot correct hypercalcemia or prevent stones.**
  The definitive treatment is often **parathyroidectomy**, which dramatically reduces stone recurrence and normalizes urine calcium.
- **Do NOT restrict fluids** unless you have heart failure or advanced kidney disease.
- **Avoid calcium supplements** unless specifically ordered by your endocrinologist.
- If you have **reduced kidney function**, consult before increasing potassium-rich fruits/vegetables.

**Bottom Line**

You **should not avoid dietary calcium**.
You **should restrict sodium and moderate animal protein**, but **PHPT requires medical/surgical treatment** to stop calcium phosphate stone formation.

**Std + QB**

Below is **safe, accurate, evidence-based medical guidance**, but **not a substitute for personal medical care**. Calcium phosphate stones in the setting of **primary hyperparathyroidism (PHPT)** require specialist evaluation because **diet alone cannot correct the underlying cause**.

**🧠 Why your stones are happening**

Calcium phosphate stones commonly occur when:

- **Blood calcium is high**
- **Urine calcium is high (hypercalciuria)**
- **Urine pH is high**
- Often driven by **primary hyperparathyroidism**, which increases calcium release from bone and increases kidney excretion of calcium.

When PHPT is present, **no amount of dietary restriction can normalize the calcium levels**, because the parathyroid glands are overproducing PTH, driving calcium up regardless of diet.

**❗ Can diet fix this?**

**No — diet cannot fix high calcium from primary hyperparathyroidism.**

Even on a low-calcium diet, the parathyroid gland continues to raise blood and urine calcium by pulling calcium from bone. This is why:

➡️ **Primary hyperparathyroidism is usually treated by parathyroid surgery**, which often corrects the high calcium, reduces urine calcium, and reduces stone risk.

**🥛 Should you avoid dietary calcium?**

**Do NOT avoid calcium unless your doctor instructs you to.**
Here’s why:

**1. Low calcium intake is harmful in PHPT**

- If you eat too little calcium, the parathyroid gland increases PTH even more.
- This **pulls calcium from your bones**, worsening bone loss and **does NOT reduce urinary calcium**.
- It may paradoxically **increase stone risk**.

**2. Normal dietary calcium is recommended**

Most guidelines recommend **normal calcium intake (~1000–1200 mg/day)** from food, not supplements, unless your physician advises otherwise.

**3. Calcium supplements are different**

**Avoid calcium supplements**, especially calcium carbonate, unless a specialist advises them, because supplements can spike urinary calcium.

**✔️ What diet CAN help (even though PHPT must be treated medically)**

**1. Stay well hydrated**

Aim for **2.5–3 liters of urine/day** to dilute calcium and phosphate.

**2. Reduce sodium intake**

A high-salt diet increases urinary calcium.
Target: **≤ 2 g sodium/day (~5 g salt)**.

**3. Moderate animal protein**

Too much animal protein increases urinary calcium and phosphate.

**4. Avoid excessive vitamin D supplementation**

Vitamin D increases calcium absorption. **Discuss with your doctor** before taking any, especially since you are hypercalcemic.

**5. Limit foods/drinks that raise urine pH**

Calcium phosphate stones favor alkaline (high pH) urine.
High-alkali loads include:

- Large amounts of fruits/vegetables (moderation is fine)
- Certain supplements (citrate mixtures, baking soda, alkali therapy)

Do **not** stop prescription alkali therapy without doctor guidance, but **avoid unnecessary over-alkalinizing supplements**.

**🏥 Treatment beyond diet**

With:

- High urine calcium (12 mmol/day is very high)
- High blood calcium
- Recurrent calcium phosphate stones
- Diagnosed primary hyperparathyroidism

➡️ **Parathyroid surgery is the definitive treatment and often prevents recurrence.**

After surgery, urinary calcium typically drops, and many patients stop forming stones.

**📌 Summary**

| **Question** | **Answer** |
| --- | --- |
| **Can diet fix this?** | ❌ No. Underlying PHPT must be treated. |
| **Should I avoid calcium?** | ❌ No. Eat normal dietary calcium; avoiding it worsens bone health and doesn’t fix the hypercalcemia. |
| **What can help meanwhile?** | Hydration, low sodium, moderate animal protein, avoid calcium supplements, avoid unnecessary alkali. |

**Case17:**

**SA + QA:**

Below is a structured, guideline-aligned interpretation **without revealing internal reasoning steps**, as requested.

**1. Metabolic Diagnosis**

The patient has **hypocitraturia** (urine citrate 1.2 mmol/day is markedly low) combined with **low urine potassium**, consistent with **dietary potassium deficiency and possibly chronic low-grade metabolic acidosis**.
Hypokalemia further supports inadequate intake or excessive losses of potassium, which directly suppresses renal citrate excretion and increases calcium oxalate stone risk.

**2. Key Dietary Modifications (Top Priorities)**

**1. Increase Alkali-Rich Potassium Intake (primarily from fruits & vegetables)**

This raises both serum potassium and urine citrate. Focus on natural potassium citrate foods, not supplements unless prescribed.

**2. Boost Citrate-Containing Foods**

Citrate directly inhibits calcium oxalate crystallization and raises urinary citrate.

**3. Reduce Acid Load from Diet**

Excess animal protein and low fruit/vegetable intake worsen metabolic acidosis and lower urine citrate. Balance protein intake (0.8–1.0 g/kg/day unless otherwise indicated).

**3. Sample Menu Suggestions (Targeted to Your Abnormalities)**

**Best Foods to ADD (high potassium & high alkali load)**

These are safe and beneficial unless you have advanced CKD (not stated here):

- **Citrus:** lemons, limes, oranges, mandarins
  - *Goal:* ½–1 cup lemon or lime juice per day diluted in water → significantly boosts urinary citrate.
- **High-potassium fruits:** bananas, melons, kiwi, apricots, mangoes
- **High-potassium vegetables:** spinach, chard, potatoes (especially baked with skin), sweet potatoes, squash, tomatoes
- **Legumes:** lentils, chickpeas, black beans
- **Nuts (moderate amounts):** almonds, pistachios (avoid excess sodium)

**Specific Meal Ideas**

- **Breakfast:**
  - Oatmeal topped with banana + blueberries
  - Water with lemon juice
- **Lunch:**
  - Baked potato with skin, topped with low-salt cottage cheese
  - Side salad with tomatoes, cucumbers, and olive oil
- **Snack:**
  - Orange or apricots
- **Dinner:**
  - Grilled chicken or tofu
  - Steamed spinach or roasted squash
  - Lentil soup
- **Hydration Strategy:**
  - Water infused with lemon/lime throughout the day
  - Aim for **>2.5 L urine output/day** unless medically restricted

**Foods to Limit (worsen acidosis and lower citrate)**

- Excess animal protein: large servings of beef, pork, poultry (>1.0 g/kg/day)
- High-sodium foods: processed meats, fast food, salty snacks
- Cola beverages (phosphoric acid → lower citrate)
- Very low-carbohydrate diets (can provoke metabolic acidosis)

**4. Safety Warning**

- Because you have **hypokalemia**, increasing dietary potassium is beneficial, but **serum potassium should be monitored**, especially if you take:
  - ACE inhibitors
  - ARBs
  - Spironolactone
  - Beta-blockers
    These can raise serum potassium unpredictably.
- If you have **CKD stage 3 or higher**, consult your physician before significantly increasing potassium-rich foods.

**Std + QA:**

Below is **general educational information**, not a medical diagnosis or individualized treatment plan. Kidney stone prevention—especially with low citrate, low potassium, and hypokalemia—**must be managed with your clinician**, who can guide testing and prescriptions (e.g., potassium citrate).
With that said, here’s the evidence-based dietary pattern typically recommended for **calcium oxalate stone formers with low urine citrate and low potassium**:

**✅ Key Dietary Goals for You**

Because your urine citrate (1.2 mmol/day) and urine potassium (25 mmol/day) are both low—and you have **hypokalemia**—the nutritional priorities are:

**1. Increase Citrate Intake (especially as potassium citrate from foods)**

Citrate helps prevent stones by binding calcium and inhibiting crystal formation. Many fruits and vegetables contain citrate, particularly when naturally high in **potassium**.

**2. Increase Dietary Potassium (from food, not supplements unless supervised)**

Potassium-rich foods tend to raise urinary citrate and help correct low serum potassium.

**3. Maintain normal calcium intake**

Contrary to intuition, **do not restrict calcium** unless instructed—normal dietary calcium reduces oxalate absorption.

**4. Reduce high-oxalate foods—but do NOT overrestrict**

Pair oxalate foods with calcium at meals to reduce absorption.

**🌿 Foods to Focus On**

These categories specifically address **low urinary citrate**, **low urinary potassium**, and **hypokalemia**.

**🍊 1. Citrate-rich fruits (especially also high in potassium)**

These tend to raise urinary citrate the most:

- **Lemons, lemon juice** (most concentrated citrate source)
- **Limes**
- **Oranges and mandarins**
- **Grapefruit** (avoid if on interacting medications)
- **Melons**: cantaloupe, honeydew
- **Kiwi**

➡️ **Practical tip**:
Daily “citrate water”: squeeze **½–1 lemon** (or lime) into water 2–3 times per day.

**🥑🍌 2. Potassium-rich fruits & vegetables**

These help address both **hypokalemia** and **low urinary citrate**. Excellent choices:

**High-potassium fruits**

- **Bananas**
- **Oranges**, orange juice (limit if watching sugar)
- **Avocado**
- **Cantaloupe**
- **Apricots** (fresh > dried)
- **Mango**, **papaya**

**High-potassium vegetables**

- **Leafy greens** (spinach is high oxalate—use in moderation; others encouraged)
- **Potatoes** (especially baked; eat the skin)
- **Sweet potatoes**
- **Squash**
- **Tomatoes & tomato juice**

➡️ These foods can noticeably increase both serum and urinary potassium.

**🥛 3. Normal calcium intake (important for stone prevention)**

Aim for **1,000–1,200 mg/day** unless your clinician advises otherwise.

Good sources:

- Milk
- Yogurt
- Cheese
- Calcium-fortified foods

**Why?**
Dietary calcium binds oxalate in the gut → prevents absorption → reduces stone risk.

**🫘 4. Low-oxalate, high-potassium plant foods**

Great options that don’t increase oxalate load:

- Cauliflower
- Broccoli
- Zucchini
- Green beans
- Bell peppers
- Cabbage
- Mushrooms
- Lentils & chickpeas (moderate potassium, low oxalate)

**🚰 5. Hydration: the most important “food”**

Goal urine output: **2.5–3 L/day**, unless restricted for medical reasons.

Use mostly:

- Water
- Citrus-infused water
- Lemonade without added sugar
- Herbal teas

**❗ Foods to Limit (not eliminate)**

These contain high oxalate and may worsen calcium oxalate stone risk *especially* if eaten without calcium:

- Spinach (very high—use rarely)
- Swiss chard
- Beets & beet greens
- Almonds and almond flour
- Cashews
- Rhubarb
- Sweet potatoes (moderate—pair with calcium)
- Chocolate / cocoa
- Black tea
- Soy products (varies)

➡️ If consuming these, pair with **120–300 mg calcium** (e.g., dairy or calcium-rich food) during the same meal.

**🧂 Sodium and Animal Protein (Important for Citrate)**

High sodium and high animal protein **lower urine citrate** and **increase stone risk**.

**Sodium target:**

- **< 2,000 mg/day**

**Animal protein:**

Prefer plant-forward meals. If eating meat, aim for:

- **< 0.8–1 g protein/kg/day from animal sources**

Examples:

- Lean poultry or fish in moderate portions
- Replace some meals with bean- or lentil-based options

**⭐ Putting It All Together:**

A daily pattern that works well for calcium oxalate stone formers with your labs:

**Breakfast**

- Oatmeal with milk or yogurt
- Banana or orange
- Herbal tea with lemon

**Lunch**

- Large salad with mixed greens (not spinach), tomatoes, bell peppers
- Grilled chicken or legumes
- Water with lemon

**Snack**

- Yogurt + fruit
- Citrus (tangerine, kiwi)

**Dinner**

- Baked potato or sweet potato (with calcium-containing food)
- Fish or tofu
- Steamed vegetables (broccoli, squash, zucchini)
- Citrus water

**⚠️ Important Medical Note**

With *documented hypokalemia*, **diet alone is often insufficient**, and potassium citrate or other potassium supplementation is typically considered. Please discuss your lab values and kidney stone history with your clinician—they may want to repeat serum electrolytes, check acid-base status, and adjust medications.

**SB + QB:**

Below is a **clinical-grade, guideline-based interpretation** of your findings, structured exactly as requested.
(General educational purposes only — not a substitute for your physician’s individualized management.)

**1. Metabolic Diagnosis**

**You have *hypocitraturia* (low urinary citrate) with concurrent *dietary potassium insufficiency* and *systemic hypokalemia*.**
This combination strongly suggests that your low citrate is *partly driven by low alkali intake* (low fruits/vegetables) and possibly *chronic mild metabolic acidosis* — both recognized risk factors for **calcium oxalate stone formation** in EAU & CUA guidelines.

Because citrate is a major inhibitor of calcium stone formation, your low levels significantly elevate recurrence risk.

**2. Key Dietary Modifications (Top Priorities)**

**🔑 Priority 1: Increase Potassium-Rich Alkali Foods to Raise Urinary Citrate**

- Goal: improve citrate excretion and correct dietary potassium deficit.
- Best sources: fruits and vegetables, especially **citrus**, **melon**, **banana**, **kiwi**, **avocado**, **spinach**, **sweet potato**, **tomatoes**.

**🔑 Priority 2: Add Citrate-Boosting Foods Daily**

- Lemon/lime juice (natural citrate) 60–120 mL/day diluted in water.
- Other high-alkali choices: oranges, cantaloupe, watermelon, coconut water (if no CKD/heart failure).

**🔑 Priority 3: Preserve Normal Dietary Calcium (1,000–1,200 mg/day)**

- Prevents increased oxalate absorption and reduces calcium oxalate stone risk.
- Pair calcium with meals that contain oxalate (e.g., leafy greens, nuts, chocolate) to bind oxalate in the gut.

**3. Sample Menu Suggestions**

**Foods to Emphasize**

**Breakfast**

- Greek yogurt + banana + handful of berries
- Whole-grain toast with avocado
- Water with fresh lemon

**Lunch**

- Spinach or mixed-greens salad with grilled chicken or tofu
- Add tomatoes, bell peppers, citrus segments
- Side of roasted sweet potato
- Sparkling water + lemon/lime

**Snacks**

- Orange slices, melon, or kiwi
- Raw vegetables with hummus
- Low-fat cheese with an apple

**Dinner**

- Grilled salmon or lentils
- Steamed vegetables (broccoli, zucchini, carrots)
- Brown rice or quinoa
- Herbal tea with lemon

**Foods to Limit (Specific to Hypocitraturia & CaOx Stones)**

- **High-sodium foods** (processed meats, canned soups, restaurant meals).
  Sodium increases calcium excretion → worsens stone risk.
- **High-oxalate foods in large amounts** unless paired with calcium:
  - Spinach (moderate amounts okay), almonds, beets, rhubarb, Swiss chard, dark chocolate.
- **Excess animal protein**, which increases acid load → lowers citrate.
  Aim for ~0.8–1.0 g/kg/day unless your clinician advises otherwise.

**4. Safety Warning**

Because you mentioned **hypokalemia**, be aware:

**⚠️ Increasing potassium-rich foods is usually safe, but NOT if you have:**

- Chronic kidney disease (CKD)
- Heart failure
- Are taking ACE inhibitors, ARBs, spironolactone, eplerenone, or potassium-sparing diuretics

If any of these apply, potassium intake must be monitored with periodic blood checks.

**⚠️ Lemon juice or potassium citrate supplements**

- Potassium citrate supplementation is often prescribed for hypocitraturia but can be dangerous if serum potassium is not monitored.
- Dietary citrate is safer, but still discuss with your clinician to make sure potassium replacement is being done appropriately.

**Std + QB:**

Below is **safe, non-prescriptive, educational information** you can discuss with your clinician. I won’t give medical orders or diagnose, but I *can* explain how diet and physiology relate to kidney-stone risk—especially in the setting of **calcium oxalate stones**, **low urinary citrate**, and **low potassium (hypokalemia)**.

**⭐ Why citrate and potassium matter**

- **Low urinary citrate** is a major risk factor for calcium oxalate stones. Citrate binds calcium in the urine, reducing stone formation.
- **Potassium-rich fruits/vegetables** are natural sources of alkali that help raise urinary citrate.
- **Hypokalemia (low blood potassium)** means the body is short on potassium system-wide. **Do NOT self-treat hypokalemia with supplements unless supervised**, because excessive or unsupervised potassium intake can be dangerous for the heart.

**🍎 Helpful dietary strategies (generally recommended for calcium oxalate stone formers)**

**1. Increase fruits and vegetables**

Fruits/vegetables provide **potassium** and **alkali**, both of which can increase urinary citrate.
Good options (low/medium oxalate):

- Citrus (lemons, limes, oranges)
- Melons (cantaloupe, honeydew)
- Bananas
- Mango, papaya
- Cauliflower, cabbage
- Zucchini, mushrooms
- Cucumbers, bell peppers
- Leafy greens *other than spinach* (spinach is very high oxalate)

💡 **Citrus is particularly helpful** because of its citrate content—lemon or lime water can raise urinary citrate mildly.

**Safety note:**
If you have **kidney disease**, **cardiac issues**, or are on **ACE inhibitors, ARBs, or potassium-sparing diuretics**, potassium from foods might still matter—ask your clinician what your upper limit should be.

**2. Maintain normal—not low—dietary calcium**

People with calcium oxalate stones often think they should cut calcium, but that **increases** stone risk because calcium binds oxalate in the gut.

Aim for **2–3 servings/day** from:

- Dairy (milk, yogurt, cheese)
- Calcium-fortified plant milks
- Tofu set with calcium

**Safety note:**
Calcium supplements (pills), especially taken away from meals, **can increase stone risk**; food sources are safer.

**3. Reduce high-oxalate foods**

Since you form calcium oxalate stones, limit the very high-oxalate items:

- Spinach, Swiss chard
- Almonds/almond flour
- Beets & beet greens
- Rhubarb
- Concentrated nuts/seeds
- Chocolate in excess
- Nut butters (limit)

Pair moderate-oxalate foods with **calcium at the same meal** to reduce absorption.

**4. Reduce sodium intake**

High sodium → lower urinary citrate & higher urinary calcium → more stone formation.

Practical tips:

- Keep total sodium ~2,000 mg/day (your clinician may individualize).
- Avoid processed meats, canned soups, fast food, salty snacks.

**5. Moderate animal protein**

High animal protein lowers urinary citrate and increases acid load, raising stone risk.

You don't need to eliminate it; aim for:

- Smaller portions
- More plant-based proteins (beans, lentils) if tolerated
- Fish and poultry over red/processed meat

**6. Hydration—most important**

Goal urine output: **≥2–2.5 liters/day**, pale yellow urine.
Add lemon/lime for citrate boost if you enjoy it.

**⚠️ Special safety considerations for YOU (low citrate + hypokalemia)**

**✔️ Foods can help, but self-treating hypokalemia is unsafe**

- Potassium supplements (pills/powders) in someone with unmonitored hypokalemia can be risky, and too much potassium can be dangerous even if you start low.
- Treating hypokalemia often requires investigating the **cause** (diuretics? diarrhea? magnesium deficiency? renal potassium loss? endocrine causes?).
- Dietary potassium alone usually **cannot correct significant hypokalemia**.

**✔️ If your clinician considers potassium citrate**

Potassium citrate is commonly prescribed for stone formers with low citrate.
Ask about:

- Safe dosing
- Monitoring blood potassium
- Kidney function
- Interactions with medications (ACE inhibitors, ARBs, potassium-sparing diuretics)

**✔️ If you get GI upset with citrus or potassium citrate**

This is common; taking with meals and adjusting the dose may help—ask your clinician.

**📋 Summary**

**Most helpful dietary steps for your situation:**

- Increase fruits/vegetables (especially citrus) for citrate and potassium
- Ensure normal dietary calcium at meals
- Lower sodium
- Moderate animal protein
- Limit high-oxalate foods
- Stay well hydrated

**Safety:**

- **Do NOT self-treat hypokalemia with potassium supplements.**
- Check with your clinician before making major potassium-rich dietary changes if you have heart, kidney, or medication considerations.
- Potassium citrate (if prescribed) requires monitoring.

**Case18:**

**SA + QA:**

Here is your personalized, guideline-based dietary plan, written for a **calcium phosphate stone former with Type 1 (distal) Renal Tubular Acidosis (dRTA)**.
*(No images or entities are included because this is a clinical content answer.)*

**1. Metabolic Diagnosis**

**You have:**

- **Hypocitraturia** (very low urinary citrate), driven by **chronic metabolic acidosis from distal RTA**.
- **Alkaline urine (pH 7.2)**, which is typical for dRTA but **promotes calcium phosphate stone formation (especially brushite)**.
- **High risk of calcium phosphate stones** due to the combination of **high pH + low citrate**.

**Key implication:**
Your stones form **not** because your urine is “too acidic” but because **it is too alkaline while citrate is too low**.
Diet alone cannot normalize pH in dRTA, but **diet can increase citrate, reduce urinary calcium load, and reduce phosphate crystallization risk**.

**2. Key Dietary Modifications (Top Priorities)**

**✔️ Priority 1 — Increase Alkali Intake *Strategically* to Raise Citrate (but not urine pH further)**

Counterintuitive, but evidence-based:
In distal RTA, **alkali therapy is needed to correct metabolic acidosis → this increases urinary citrate**, even if urine pH does not fall.

**Best dietary sources** (moderate amounts to prevent pH over-alkalinization):

- Citrus fruits (lemon, lime, orange)
- Melons
- Vegetables rich in citrate (zucchini, cucumber, leafy greens)

**✔️ Priority 2 — Reduce Sodium Intake < 2 g/day (≈ 5 g salt/day)**

High sodium **increases urinary calcium**, worsening CaP stone risk.

Avoid:

- Canned soups
- Fast foods
- Processed deli meats
- Restaurant meals with unknown sodium loads

**✔️ Priority 3 — Maintain Normal Calcium Intake (1,000–1,200 mg/day)**

**Do NOT restrict calcium** — restriction **raises oxalate absorption** and does *not* reduce CaP stones.
But pair calcium with meals to bind intestinal phosphate and oxalate.

Sources:

- Dairy (yogurt, milk, cheese)
- Calcium-set tofu
- Almonds, leafy greens (in moderation)

**3. Sample Menu Suggestions**

**Breakfast**

**Good choices:**

- Greek yogurt + fresh berries
- Oatmeal cooked with milk
- Small glass of orange juice (½ cup)

**Avoid:**

- Salted breakfast sandwiches
- Bacon/sausage (sodium & animal protein load)

**Lunch**

**Good choices:**

- Grilled chicken breast
- Quinoa or brown rice
- Spinach or cucumber salad with lemon dressing
- Unsalted nuts (small portion)

**Avoid:**

- Pickles, olives, canned beans without rinsing (high sodium)
- Large portions of cheese-heavy meals

**Dinner**

**Good choices:**

- Baked salmon
- Steamed vegetables (zucchini, carrots, broccoli)
- Baked potato with plain yogurt instead of salty toppings

**Avoid:**

- High-salt sauces (soy sauce, teriyaki, marinades)
- Processed meats

**Snacks**

**Good:**

- Fruit (orange, melon, grapes)
- Yogurt
- Raw veggies
- Lemon water

**Avoid:**

- Chips, crackers, pretzels
- Salted nuts

**Fluid Strategy**

- Aim for **2–3 L/day**, unless restricted by another doctor.
- Include **citrate-rich beverages**, e.g., lemon water.
- Avoid alkaline bottled waters (pH > 8), which may raise urine pH further.

**4. Safety Warning**

**Important for Distal RTA**

- You **require medical alkali therapy (usually potassium citrate)** to correct systemic acidosis and raise urinary citrate adequately.
  Diet **cannot** normalize citrate without pharmacologic alkali in dRTA.

**Potassium considerations**

- If you take **potassium citrate**, avoid very high-potassium diets without monitoring (e.g., large amounts of bananas, avocado, coconut water), especially if you have any reduced kidney function.

**Urine pH target**

- In calcium phosphate stone formers, **urine pH > 6.8 increases stone risk**.
  Your physician may adjust potassium citrate dose to raise citrate without increasing pH.

**Summary**

You should focus on:

1. **Increasing citrate intake** (foods + prescribed alkali therapy).
2. **Lowering sodium to reduce urinary calcium.**
3. **Maintaining normal dietary calcium (do NOT reduce).**
4. **Moderating alkali-rich waters** so urine pH does not rise further.

**Std + QA:**

Here is evidence-based **general dietary guidance** for someone with **calcium phosphate kidney stones** and **type 1 renal tubular acidosis (RTA)**, **not individualized medical instructions**. Because you have a real medical condition, please review this with your nephrologist or dietitian, who can tailor it to your labs, medications, and overall health.

**⭐ Key Issues in Your Case**

- **Calcium phosphate stones** tend to form in **high-pH urine** (typically >6.8). Your urine pH of **7.2** increases this risk.
- **Type 1 RTA** causes **chronic metabolic acidosis**, **low citrate**, and can paradoxically raise **urine pH**, creating a perfect environment for calcium phosphate stones.
- **Low citrate** matters because citrate is a natural inhibitor of stone formation.

Your dietary strategy must:

1. Increase urinary citrate
2. Avoid making urine pH even higher
3. Prevent calcium and phosphate supersaturation
4. Support management of RTA

**✔️ Diet Recommendations (General Guidance)**

**1. Ensure adequate dietary calcium — but from food, not supplements**

- Aim for **1,000–1,200 mg/day** from food such as dairy, fortified plant milks, yogurt, cheese, or leafy greens.
- **Why:** Adequate calcium binds dietary phosphate and oxalate in the gut and reduces stone risk.
- **Avoid:** Calcium supplements unless your doctor prescribes them, because excess supplemental calcium may increase stone risk.

**2. Limit high-phosphate processed foods**

Phosphate additives absorb extremely well and increase urinary phosphate.

Avoid or limit:

- Processed meats
- Cola beverages (contain phosphoric acid)
- Fast foods
- Packaged baked goods
- Processed cheeses

Choose whole, minimally processed foods whenever possible.

**3. Encourage citrate-rich foods (but be cautious about alkalinizing load)**

Normally, people with low citrate are encouraged to eat citrus fruits.
**But calcium phosphate stone formers must be careful**, because many fruits/juices raise urine pH further.

Better tolerated options:

- **Lemon juice** (in modest amounts): provides citrate with **minimal alkalinizing effect** compared to orange juice.
- **Diluted lemonade** without excess sugar.

Avoid or limit:

- Orange juice, grapefruit juice, and smoothies with large fruit loads → These can **significantly raise urine pH**, worsening Ca-phos stone risk.

**4. Maintain a moderate-protein diet**

High animal protein increases acid load, which **may help lower pH** slightly, but also:

- Reduces urinary citrate
- Increases calcium excretion

So aim for **moderation** rather than high-protein diets.

Good choices:

- Fish
- Poultry
- Eggs
- Plant proteins (lentils, beans, tofu)

Avoid excessive intake of:

- Large portions of red meat daily
- High-protein weight-loss diets (keto, carnivore, etc.)

**5. Reduce sodium intake**

High sodium increases urinary calcium loss.

Target:

- **< 2,000 mg sodium/day**
- Avoid packaged soups, chips, cured meats, pickles, and restaurant foods when possible.

This is one of the most effective dietary steps for lowering stone risk.

**6. Hydration — but avoid alkaline waters**

Your urine pH is already high. Some waters (alkaline water, mineral waters high in bicarbonate) raise pH further.

Hydration tips:

- Aim for **2–3 liters/day** total fluid intake.
- Stick to plain water, lightly flavored waters, herbal teas.
- Avoid alkaline waters with pH > 8 or marketed as “alkalizing.”

**7. Be mindful with fruits & vegetables**

They are generally healthy, but large amounts of highly alkalizing plant foods can raise urine pH.

Tips:

- Eat moderate portions of fruits and vegetables.
- Prefer less alkalizing options (berries, apples, cauliflower, zucchini).
- Limit very alkalizing foods (spinach, beets, potatoes, sweet potatoes in large amounts).

This does *not* mean to avoid vegetables — just avoid very large servings that shift urine pH.

**8. Avoid added sugars & refined carbohydrates**

High sugar intake increases calcium excretion and can worsen stone risk.

Reduce:

- Sweetened beverages
- Pastries
- Candy
- Sugary cereals

**⭐ Special Consideration: Type 1 RTA**

Most people with Ca-phos stones are advised **not** to take alkalinizing agents because they raise urine pH.
But **in type 1 RTA, alkali therapy (such as potassium citrate)** is often required to:

- Correct chronic acidosis
- Raise blood bicarbonate
- Increase urinary citrate

However, alkali dosing must be **very carefully titrated** to avoid worsening your already high urine pH.
This is a **medical management issue**, not a diet-only issue.

**🚫 Diet Myths to Ignore**

- **“Avoid all calcium.”** → This increases stone risk.
- **“Drink lots of orange juice for citrate.”** → Not good for Ca-phosphate stones with high urine pH.
- **“Alkaline diets prevent stones.”** → Opposite effect for your stone type.

**✅ Summary — Best Eating Pattern for You**

A **balanced, whole-food diet** focusing on:

- Normal calcium intake from food
- Low sodium
- Moderate animal protein
- Citrus **only from lemon/lime**
- Avoiding high-phosphate processed foods
- Hydration with non-alkaline water

This approach supports stone prevention **without excessively raising urine pH**.

**SB + QB:**

Below is a **distal RTA–specific, calcium-phosphate–specific** prevention plan following EAU/CUA dietary principles **plus** the unique physiology of incomplete/complete dRTA.

**1. Metabolic Diagnosis**

**The patient has calcium phosphate stone disease driven by distal renal tubular acidosis (dRTA) with marked hypocitraturia.**

- dRTA → **chronically alkaline urine** (often > 6.8) + **systemic acidosis**, which suppresses citrate production and increases stone risk.
- Low citrate is the *primary* metabolic abnormality; the alkaline urine is *not* from excessive alkali intake but from impaired acid secretion.

Diet alone cannot correct the underlying tubular defect; **medication (potassium citrate or alkali therapy) is essential** to correct systemic acidosis and raise urinary citrate.

**2. Key Dietary Modifications (Top 3 Priorities)**

**Priority 1: Protect Citrate Excretion**

Even though urine pH is high, **you still need citrate supplementation**—dietary or pharmacologic. Dietary strategies can help but **cannot replace pharmacologic citrate** in dRTA.

- Increase intake of **citrate-rich, low-potassium-risk fruits** such as lemon juice, lime juice, small portions of oranges (if potassium levels allow).
- Avoid **high-protein acid loads**, which worsen systemic acidosis and suppress citrate.

**Priority 2: Reduce Calcium Phosphate Supersaturation Without Lowering Calcium Intake**

For CaP stones, aim to:

- **Reduce sodium** → lowers urinary calcium. *(Target < 2 g sodium/day ≈ < 5 g salt/day)*
- Maintain **normal dietary calcium** (1,000–1,200 mg/day from food).
- **Avoid excessive alkali foods** *only if they worsen urine pH above ~7.5*. (In dRTA, diet rarely changes pH meaningfully.)

**Priority 3: Moderate Animal Protein**

High animal protein → metabolic acid load → increased bone resorption → increased calcium + lower citrate.
Target: **0.8–1.0 g/kg/day** total protein, with more **plant-based** sources.

**3. Sample Menu Suggestions**

**Foods & Drinks to Prioritize**

**Citrate-boosting (but not excessively alkalinizing)**

- **Lemon water:** 60–120 mL lemon juice/day diluted in water.
- **Lime water** as an alternative.
- **Citrus fruit** in small portions (monitor potassium if needed).

**Low-sodium, calcium-appropriate meals**

- Breakfast:
  - Greek yogurt (unsweetened) + blueberries
  - Oatmeal cooked in milk
- Lunch:
  - Grilled chicken or tofu
  - Brown rice or quinoa
  - Steamed vegetables (avoid adding salt; use herbs instead)
- Dinner:
  - Baked salmon or lentils
  - Sweet potato or whole grains
  - Leafy greens with olive oil & lemon

**Hydration**

- **2–2.5 L/day**, spaced throughout the day.
  - Do not force >3 L if you have cardiac or renal comorbidities.

**Foods to Limit or Avoid**

**Raise urine calcium or phosphate supersaturation**

- Excessive **sodium**: processed foods, restaurant meals, deli meats.
- **High animal protein**: large portions of beef, poultry, fish in one meal.

**Strong alkalinizers that can push pH > 7.5**

(This is rare but worth monitoring with home urine dipsticks.)

- Excessive vegetable-only meals (large green smoothies)
- Large doses of bicarbonate-containing supplements
- Some alkaline waters (pH > 9)

**High-oxalate foods are less central in CaP stones, but moderation is still reasonable:**

- Spinach, almonds, beets, rhubarb.

**4. Medication vs. Diet—What Does Each Do?**

**Medication (Essential): Potassium Citrate or Alkali Therapy**

In dRTA, stones form because systemic acidosis suppresses citrate and promotes calcium release from bone.
**Only alkali replacement corrects this.**

Potassium citrate will:

- Raise urine citrate significantly
- Reduce calcium phosphate crystallization
- Improve systemic acid-base balance
- Sometimes slightly increase urine pH (this matters, see below)

**Dose adjustments allow your clinician to increase citrate without excessively raising urine pH above ~7.2–7.4.**

**Bottom line: In dRTA, citrate therapy is not optional. Diet alone cannot normalize citrate.**

**Diet (Adjunctive but Very Important)**

Diet helps by:

- Lowering urinary calcium (via sodium restriction)
- Reducing acid load (more plant proteins; less animal protein)
- Supporting citrate formation (citrus intake)
- Improving hydration and reducing supersaturation

Diet **cannot normalize systemic acidosis**, and thus cannot fully correct the underlying problem.

**4. Safety Warning**

- **Monitor serum potassium** if you take potassium citrate, especially if on ACE inhibitors, ARBs, or have CKD.
- If you have kidney impairment, do **not exceed 2–2.5 L/day** fluid intake without your nephrologist’s guidance.
- Track home **urine pH** to ensure potassium citrate does not raise pH > 7.5, which increases CaP crystallization.

**Summary (One Sentence)**

**You need potassium citrate medication to correct systemic acidosis and raise citrate, while diet (low sodium, moderate protein, citrus intake) lowers urinary calcium and supports citrate—but cannot replace the medication in distal RTA.**

**Std + QB:**

Below is **general medical information**, not personal medical advice. With **distal renal tubular acidosis (dRTA)** and **calcium phosphate (CaP) stones**, the strategy is different from the more common calcium-oxalate stone prevention. The key issues in *your* scenario are:

- **Urine is already alkaline (pH ~7.2)** → further alkalinization can worsen CaP stone formation.
- **Citrate is low** → citrate is protective *if* you can raise citrate without raising urine pH too much.
- **dRTA itself causes low citrate, high pH, and high calcium excretion** → so medication is often required alongside diet.

Below is what is typically recommended.

**1. What to eat or drink**

**✔ Hydration: the single strongest dietary tool**

- Goal: **2.5–3 L of urine/day** (usually ~3–3.5 L fluid intake).
- Best fluids: **water**, **citrus-containing drinks** (lemon/lime) *with caution* (see below).
- Avoid: high-sugar beverages.

**Why:** Dilutes urinary calcium and phosphate and reduces supersaturation.

**✔ Citrate-containing foods—but carefully**

Because your urine is already alkaline, the goal is to raise **citrate** without raising **pH** too much.

- **Lemon or lime juice** provides **citric acid**, which can increase urinary citrate with *less* pH rise than potassium citrate.
- Avoid large amounts of **orange juice** and **alkaline waters**—they raise urine pH more aggressively.

**Typical food strategy:**

- Add **1–2 oz lemon or lime juice per day** (diluted in water).
- Monitor urine pH (home dipsticks): if it rises above ~7.4, reduce citrus intake.

**✔ Maintain normal dietary calcium**

- Include **2–3 servings/day** of dairy or calcium-rich foods.
- Take calcium **with meals** if using supplements.

**Why:** Helps bind dietary phosphate and oxalate in the gut; paradoxically reduces stone risk.
**Avoid:** Very low calcium diets—they worsen hypercalciuria and stone risk.

**✔ Reduce sodium (major in CaP stone formers)**

- Target: **<2 g sodium/day** (~5 g salt).
- Avoid processed foods, fast foods, canned soups, salted snacks.

**Why:** Sodium directly increases **renal calcium excretion**, a big contributor to CaP stones.

**✔ Moderate animal protein**

- High animal protein → increases calcium excretion and lowers citrate.
- Aim for **0.8–1.0 g/kg/day** or your clinician’s target.
- Favor plant proteins more often.

**✔ Avoid excessive alkaline foods and supplements**

- Avoid **alkaline waters**, baking soda drinks, “alkaline diets,” or potassium / magnesium alkalinizing supplements unless prescribed. You already have a high urine pH.

**2. How much is diet vs. medication in your case?**

With **distal RTA**, diet alone usually **cannot** correct the underlying issues:

**✔ Distal RTA causes:**

- Chronic metabolic acidosis
- Low urinary citrate (hypocitraturia)
- High urine pH (paradoxical)
- Calcium leaching from bone → hypercalciuria

Because of this physiology:

**Medication usually plays a major role (often essential):**

**1. Potassium citrate (but carefully titrated)**

- First-line for dRTA to correct systemic acidosis and raise citrate.
- *But* if dose is too high → raises urine pH even more → increases CaP stone formation.

**Typical approach by clinicians:**

- Start low; target correction of acidosis and enough citrate rise **without letting urine pH exceed ~7.0–7.2**.
- Adjust based on 24-hr urine.

Some patients with CaP + dRTA require **split dosing**, bedtime dosing, or alternative alkalinizers.

**2. Thiazide diuretics (if hypercalciuria)**

- Reduce urinary calcium.
- Often prescribed when sodium restriction alone is insufficient.

**3. Alkali therapy guided by blood bicarbonate**

- dRTA treatment aims to normalize serum bicarbonate (~22–24 mEq/L).
- Doing this reduces bone calcium loss and citrate wasting.

**3. What diet can accomplish vs. what medication must accomplish**

| **Goal** | **Diet Helps?** | **Medication Needed?** | **Notes** |
| --- | --- | --- | --- |
| Increase urine volume | ⭐⭐⭐⭐ | – | Hydration has biggest impact. |
| Reduce urinary calcium | ⭐⭐ (via low sodium, moderate protein) | ⭐⭐⭐⭐ (thiazides often needed) | dRTA promotes hypercalciuria regardless of diet. |
| Raise urinary citrate | ⭐ (citrus) | ⭐⭐⭐⭐ (K-citrate) | Food-based citrate rarely sufficient in dRTA. |
| Lower urine pH | ❌ | ❌ | In dRTA, pH stays high; cannot be lowered through diet. |
| Correct systemic acidosis | ❌ | ⭐⭐⭐⭐ | Requires alkali replacement. |
| Reduce phosphate supersaturation | ⭐ | ⭐ | Balance calcium + hydration. |

**4. Practical daily plan (generic template)**

**Morning**

- Large glass of water
- Optional: water with ½ oz lemon juice
- Normal-calcium breakfast
- Keep sodium low

**Midday**

- Water + lemon splash
- Balanced meal → avoid very salty foods
- Moderate protein

**Evening**

- Hydration to meet daily goal
- Calcium-containing food with dinner
- Take prescribed potassium citrate **only as directed** and monitor urine pH

**Throughout the week**

- Avoid juices/sodas with added sugar
- Track urine pH/citrate if instructed
- Keep a low-sodium eating pattern

**5. When to follow up / what to monitor**

Ask your clinician about:

- A **24-hour urine test** after any diet or medication change
- Monitoring **serum potassium**, bicarbonate, and kidney function while on citrate therapy
- Whether your urine pH on the current regimen is acceptable (goal often ~6.8–7.2, individualized)

**Summary**

For **calcium phosphate stones + distal RTA**, diet helps significantly with **hydration, sodium restriction, and normal calcium intake**, but **cannot fix low citrate or systemic acidosis**. Medication—particularly **potassium citrate (carefully titrated)** and sometimes **thiazides**—is almost always necessary. Food-based citrate is helpful but not enough alone.

**Case19:**

**SA + QA:**

Below is your structured interpretation and recommendations based on EAU/CUA guideline-aligned metabolic management principles.

**1. Metabolic Diagnosis**

Your urine oxalate of **0.6 mmol/day** (≈ **53 mg/day**) is **elevated**, as the typical reference upper limit is ~**0.46 mmol/day (40 mg/day)**.
This suggests **secondary hyperoxaluria**, and in your case the most likely driver is **high-dose vitamin C supplementation (1000 mg/day)**, which is metabolized to oxalate.

**2. Key Dietary Modifications (Top Priorities)**

**1. Stop high-dose vitamin C supplements (>200 mg/day).**

- Vitamin C is converted to oxalate; doses above **500–1000 mg/day** significantly increase urinary oxalate and stone risk.
- The CUA and EAU specifically caution against megadosing vitamin C in stone formers.

**2. Increase dietary calcium with meals (NOT supplements on an empty stomach).**

- Aim for **1000–1200 mg/day**, mainly from food (milk, yogurt, cheese).
- Calcium binds oxalate in the gut → reducing its absorption → lowering urinary oxalate.

**3. Reduce high-oxalate foods only if you consume them frequently.**

- Avoid unnecessary restriction; target the highest offenders.

**3. Sample Menu Suggestions**

**Foods to Prioritize**

**At meals (for calcium–oxalate binding):**

- 1 cup **yogurt** with lunch
- A slice of **cheese** with dinner
- **Milk** with breakfast cereals

**Low-oxalate fruits & vegetables:**

- Apples, pears, grapes, mango, melons
- Cauliflower, cabbage, cucumbers, zucchini, mushrooms

**Protein & Sodium moderation:**

- Chicken, fish, tofu
- Limit processed meats and salty foods to keep sodium <2 g/day

**Foods to Limit (High-Oxalate) — Only if eaten frequently**

- **Spinach**, Swiss chard
- **Almonds**, cashews
- **Beets**, rhubarb
- **Dark chocolate**, cocoa
- **Potato skins**
- **Large amounts of berries**

**4. Safety Warning**

If you have **CKD**, heart failure, or are on medications affecting potassium or calcium balance, DO NOT increase calcium-rich foods or fluids without medical supervision.

**Summary**

Yes — your **vitamin C 1000 mg/day is very likely contributing to your elevated urinary oxalate**. Stopping high-dose vitamin C and optimizing calcium intake with meals are the most effective first steps to lowering oxalate excretion and reducing recurrence risk.

**Std + QA:**

Here’s a clear, safe, evidence-based explanation you can take to your clinician for confirmation:

**Could vitamin C be part of the problem?**

Possibly **yes**. High-dose vitamin C (ascorbic acid) can increase urinary oxalate because part of it is metabolized into oxalate. Several studies have shown that **1,000 mg/day or more** can raise urinary oxalate and may increase the risk of **calcium oxalate stone recurrence**, especially in people who have formed stones before.

Your urinary oxalate level of **0.6 mmol/day (~52 mg/day)** is in the upper range of normal and may be influenced by supplemental vitamin C.

**What you should consider doing**

These steps are generally recommended for calcium oxalate stone prevention, but **your treating clinician must confirm** what is right for you:

**1. Stop or reduce high-dose vitamin C supplements**

- Many guidelines recommend **avoiding doses >200 mg/day** if you have calcium oxalate stones.
- Most people can meet their vitamin C needs through food alone.

**2. Maintain normal—not low—dietary calcium**

- Aim for **1,000–1,200 mg/day** from food (e.g., dairy, fortified alternatives).
- Calcium binds oxalate in the gut and lowers its absorption.

**3. Moderate high-oxalate foods only if needed**

Since your urinary oxalate is not extremely high, you may not need strict restriction.
Still, avoid over-consuming:

- Spinach, rhubarb
- Almonds, cashews
- Beets
- Dark chocolate
- Wheat bran

**4. Increase citrate intake**

Citrate helps prevent calcium stones.

- Drink **lemon or lime juice** in water
- Your clinician may recommend **potassium citrate** if your urinary citrate is low

**5. Stay well hydrated**

- Goal: **≥2–2.5 liters of urine/day**
- This is one of the strongest factors for preventing recurrence.

**6. Reduce sodium intake**

- High sodium increases urinary calcium.
- Limit to **<2,300 mg/day**, often aiming for **1,500 mg/day** if you have stones.

**7. Moderate animal-protein intake**

- Large amounts increase calcium and lower citrate.
- Typical target: **0.8–1.0 g/kg body weight/day** of total protein.

**Summary**

Yes—**1,000 mg/day of vitamin C could be increasing your urinary oxalate and contributing to stone recurrence**.
Discuss with your clinician stopping high-dose supplementation and following the above prevention strategies.

**SB + QB:**

Here is your structured metabolic analysis and dietary plan based on the information you provided.

**1. Metabolic Diagnosis**

**Mild Hyperoxaluria likely driven by excessive Vitamin C intake** → High-dose ascorbic acid is metabolized to oxalate, increasing urinary oxalate excretion.
Your urine oxalate of **0.6 mmol/day (~53 mg/day)** is above the ideal threshold (<0.46 mmol/day / <40 mg/day), consistent with this mechanism.

Because you form **calcium oxalate stones**, even mild elevations in urinary oxalate increase supersaturation and stone risk.

**2. Key Dietary Modifications (Top Priorities)**

**1. Discontinue high-dose Vitamin C supplements**

- EAU & CUA guidelines both identify supplemental vitamin C >500 mg/day as a modifiable cause of hyperoxaluria.
- Dietary vitamin C from fruits/vegetables is safe.

**2. Ensure calcium intake with meals (NOT restriction)**

Dietary calcium **750–1200 mg/day**, consumed **with** oxalate-containing meals, binds oxalate in the gut and *reduces absorption*.
Examples: dairy, fortified alternatives, leafy greens (low-oxalate), canned fish with bones.

**3. Moderate high-oxalate foods + match with calcium sources**

Not complete avoidance—just portion control + pairing with calcium.

**3. Sample Menu Suggestions**

**Foods to Eat (Helpful for Reducing Oxalate Absorption)**

- **Greek yogurt or milk with meals** (binds oxalate)
- **Citrus fruits** (natural citrate increases urinary citrate)
- **Low-oxalate vegetables:** cauliflower, cabbage, mushrooms, carrots, zucchini
- **Lean proteins:** chicken, fish, tofu (moderation per guidelines)
- **Whole grains, oats, barley** (lower oxalate vs. wheat bran)

**Foods to Limit (High Oxalate or Worsen Risk)**

- **Stop vitamin C supplements** >250–500 mg/day
- **Avoid megadose vitamin C powders or "immune booster" packets**
- **Reduce high-oxalate foods:** spinach, almonds, beets, rhubarb, Swiss chard, peanuts
- **Limit very high-oxalate beverages:** black tea, dark chocolate drinks, certain kombuchas
- **Limit excess animal protein** (raises calcium and lowers citrate)

**Example Daily Menu**

**Breakfast**

- Oatmeal with milk + berries
- Scrambled egg
  *(Avoid smoothies with spinach or high-dose vitamin C packets.)*

**Lunch**

- Grilled chicken with rice, carrots, and zucchini
- Small yogurt cup with the meal

**Dinner**

- Salmon with roasted cauliflower and quinoa
- Citrus fruit (orange or mandarin)

**Snacks**

- Cheese stick
- Fresh fruit (avoid excessive kiwis if you're sensitive)

**4. Safety Warning**

No comorbidities were provided, so **standard fluid recommendations apply (2.5–3.0 L/day)** unless you have:

- Heart failure
- Advanced CKD
- Hyponatremia
  If any of these exist, fluid recommendations must be individualized—tell me if they apply.

**Bottom Line**

**Yes — you should stop high-dose vitamin C supplements.**
They directly increase urinary oxalate and promote calcium oxalate stone recurrence.
Focus on **adequate calcium with meals**, **moderating high-oxalate foods**, and **eliminating supplemental vitamin C**.

**Std + QB:**

Below is **general educational information**, not personal medical advice. Kidney-stone prevention decisions should always be confirmed with your clinician, especially because they depend on your full medical history, lab results, and medications.

**🟣 1. Should you stop high-dose vitamin C?**

**Very likely yes.**
High-dose vitamin C (ascorbic acid) is a well-known contributor to elevated urinary oxalate because excess vitamin C is metabolized into oxalate. Even “mild” hyperoxaluria (like your **0.6 mmol/day**) can matter if you form **calcium oxalate stones repeatedly**.

**Typical guidance:**

- Limit vitamin C supplements to ≤ **100–200 mg/day** unless medically indicated.
- Avoid high-dose tablets (e.g., 500–2000 mg/day). These have been shown to raise urinary oxalate and increase stone risk.

So yes—**discontinuing the high-dose supplement or reducing it to a minimal daily amount is usually recommended.**

**🟣 2. Additional steps to reduce calcium oxalate stone recurrence**

**✔️ Maintain adequate dietary calcium**

This may sound counterintuitive, but **calcium binds oxalate in the gut**, preventing absorption.

- Aim for **1,000–1,200 mg/day** from food (e.g., dairy, fortified plant milks, leafy greens that are low in oxalate).
- **Do NOT** take calcium supplements unless your clinician instructs, because supplements can sometimes raise stone risk if not taken with meals.

**✔️ Increase hydration**

- Target **≥2.5 liters of urine per day** (usually ~3 liters of fluid intake), unless restricted for medical reasons.
- Water is best; citrus-based beverages (e.g., lemon water) can add citrate, which is protective.

**✔️ Moderate high-oxalate foods**

Your oxalate level is only mildly high, so a strict low-oxalate diet is usually unnecessary. But moderating the major offenders can help:

- Spinach, almonds, rhubarb, beets, Swiss chard, peanuts, chocolate, wheat bran.
- If you eat them, pair with calcium-containing foods **within the same meal**.

**✔️ Boost urinary citrate**

Low urinary citrate promotes calcium oxalate stones. If your citrate is low (not stated here), your clinician may recommend:

- **Citrate-rich foods**: lemons/limes, oranges, melon.
- **Potassium citrate supplement** if needed.

**✔️ Reduce sodium intake**

High sodium increases urinary calcium, which raises stone risk.

- Aim for **<2,000 mg sodium/day**.
- Avoid processed or packaged foods as much as possible.

**✔️ Keep animal protein moderate**

High animal protein increases calcium and lowers citrate.

- Choose reasonable portion sizes (~0.8–1 g protein/kg body weight/day unless otherwise directed).

**🟣 3. When to pursue further evaluation**

If you continue to form stones despite dietary changes, your clinician may consider:

- repeating a **24-hour urine analysis**,
- checking serum calcium and parathyroid hormone,
- reviewing medications or supplements that may contribute.

**🟣 Summary**

**Stopping the high-dose vitamin C supplement is strongly advisable**, since it likely contributes to your elevated urine oxalate and recurring calcium oxalate stones. In addition, emphasize:

- adequate dietary calcium,
- high fluid intake,
- moderate oxalate,
- reduced sodium,
- moderate animal protein,
- increased citrate intake.

**Case20:**

**SA + QA:**

Below is your personalized, guideline-based prevention plan for calcium oxalate stones.

**1. Metabolic Diagnosis**

- **Low urine volume** (1.5 L/day → target is ≥2.0–2.5 L/day).
- **Borderline hypercalciuria** (7.0 mmol/day is at the upper limit of normal; often driven by excess dietary sodium or animal protein).
- No evidence of other metabolic abnormalities provided.

**Interpretation:**
Your stone risk is primarily driven by **concentrated urine** and **sodium- or protein-sensitive urinary calcium excretion**, not by dietary calcium intake.

**2. Key Dietary Modifications (Top Priorities)**

**1. Increase daily fluid intake**

- Goal: **2.5–3.0 L/day total intake** to produce **≥2.0–2.5 L urine/day**.
- Best fluids: water, diluted citrus beverages (e.g., lemon/lime water for citrate).

**2. Reduce dietary sodium**

- Goal: **<2,300 mg/day**, ideally **1,500–2,000 mg/day**.
- Rationale: Every additional gram of sodium increases urinary calcium losses and worsens stone risk.

**3. Moderate animal protein intake**

- Goal: **0.8–1.0 g protein/kg/day** (usually 60–80 g/day for a 60-year-old male).
- Rationale: Animal protein increases calcium excretion and lowers citrate.

**Important:**
**Do NOT reduce dietary calcium.** Keep **1,000–1,200 mg/day** from food to bind oxalate in the gut.

**3. Sample Menu Suggestions**

**Foods to Include**

**Breakfast**

- Oatmeal with low-fat milk (calcium source)
- Fresh fruit (banana, berries)
- Lemon water or plain water

**Lunch**

- Grilled chicken breast or tofu
- Brown rice or quinoa
- Steamed vegetables without added salt
- Water flavored with lemon

**Dinner**

- Baked salmon or lentils
- Roasted vegetables seasoned with herbs only
- Small portion of whole-grain pasta
- Water or citrus-infused water

**Snacks**

- Yogurt, almonds (small handful), fresh fruit
- Low-sodium popcorn
- Water bottle carried throughout the day

**Foods to Limit or Avoid**

- Processed foods: deli meats, canned soups, packaged snacks
- Fast food, restaurant meals (very high sodium)
- Excessive animal protein (>2 servings/day)
- High-oxalate foods in large amounts: spinach, beets, almonds, rhubarb
- Cola beverages (acidic, phosphate-containing)

**4. Safety Warning**

- If you have **heart failure, uncontrolled hypertension, or advanced kidney disease**, fluid recommendations must be individualized—consult your physician before increasing fluid intake.
- If you take **thiazide diuretics**, sodium restriction is mandatory to prevent potassium loss.

If you’d like, I can provide a tailored plan that incorporates your complete 24-hour urine report (citrate, oxalate, uric acid, pH), medications, or comorbidities for a more comprehensive prevention strategy.

**Std + QA:**

Below are the **most evidence-based lifestyle and diet strategies** to prevent recurrence of **calcium oxalate kidney stones**, tailored to your information (urine volume 1.5 L/day and borderline-high urine calcium at 7.0 mmol/day).

**🚰 1. Increase Fluid Intake (Top Priority)**

Your current urine volume (1.5 L/day) is **below** the recommended level for stone prevention.

**Goal:**

- Produce **≥ 2.5 L of urine per day**.
- This usually requires drinking **3.0 L/day** of fluids, depending on activity and climate.

**Tips:**

- Water is best.
- Spread intake across the day.
- Include one glass at bedtime.
- Light-colored urine is a good sign.

**🧂 2. Reduce Sodium Intake (Supports Lower Urine Calcium)**

Borderline-high urine calcium responds strongly to **salt reduction**.

**Target:**

- **< 2,000 mg sodium/day** (about 5 g table salt).

**Why it matters:**
High sodium increases calcium loss into the urine, raising stone risk.

**How to cut sodium:**

- Avoid processed foods, canned soups, deli meats, fast food.
- Cook at home with herbs and spices instead of salt.
- Choose “low-sodium” labeled items.

**🥛 3. Maintain *Normal* Dietary Calcium (Do NOT restrict)**

Many people mistakenly avoid calcium, but that **increases** oxalate absorption.

**Goal:**

- **1,000–1,200 mg/day** from food, primarily dairy or fortified alternatives.

**Best sources:**

- Milk, yogurt, cheese
- Calcium-fortified plant milks

**Key point:**
Take calcium **with meals**, especially those containing oxalate.

**🌿 4. Moderate High-Oxalate Foods (Don’t eliminate completely)**

Because your stone type is calcium oxalate, you should reduce—but not forbid—certain foods.

**Limit (not eliminate):**

- Spinach
- Beet greens, Swiss chard
- Rhubarb
- Almonds, peanuts
- Wheat bran
- Chocolate
- Black tea

**Practical strategy:**
Combine oxalate-containing foods with **calcium-rich foods** at meals to bind oxalate in the gut.

**🥩 5. Reduce Excess Animal Protein**

High animal protein intake can increase urine calcium and decrease citrate.

**Recommended approach:**

- Keep animal protein to **0.8–1 g/kg/day** (for most men, ~60–80 g/day).
- Favor plant proteins more often.
- Limit large portions of red meat, poultry, and fish.

**🍋 6. Increase Citrate Intake (Natural Stone Inhibitor)**

Citrate helps prevent crystals from forming.

**Ways to increase citrate:**

- Drink **lemon or lime water** regularly (e.g., juice of ½–1 lemon daily).
- Include more fruits and vegetables in meals.

**🥤 7. Avoid Excess Sugar and Soda**

- High sugar intake increases stone risk.
- Colas contain phosphoric acid, which increases calcium in urine.
- Prefer water, infused water, or citrus-based drinks.

**🍺 8. Alcohol & Caffeine**

Moderate caffeine is fine.
Alcohol is not directly harmful for stones, but can cause dehydration—so balance with extra water.

**⚖️ 9. Maintain a Healthy Weight**

Obesity increases the risk of recurrent stones. Aim for a gradual, sustainable weight strategy if needed.

**🧪 10. Follow-Up Testing**

Because your urine calcium is borderline, repeat **24-hour urine testing** after 2–3 months of lifestyle changes is recommended.
If urine calcium remains high, your clinician may consider adding a **thiazide diuretic**.

**📌 Summary of What Will Help You Most**

1. **Increase total fluid → aim for ≥ 2.5 L urine/day.**
2. **Reduce sodium below 2,000 mg/day.**
3. **Maintain normal dietary calcium (1,000–1,200 mg/day).**
4. **Limit high-oxalate foods and pair them with calcium.**
5. **Reduce large amounts of animal protein.**
6. **Increase citrate via fruits & lemon water.**

**SB + QB:**

Below is a **realistic, guideline-based priority plan** according to the EAU & CUA recommendations for a patient with **borderline hypercalciuria** and **low urine volume** who continues to form **calcium oxalate stones**.

**1. Metabolic Diagnosis**

**Mild (borderline) hypercalciuria with low urine output**, consistent with a **fluid-dependent and sodium-sensitive calcium leak**, which is enough to sustain calcium oxalate stone formation even without severe metabolic abnormalities.

Because calcium is only *borderline high*, your **urine volume of 1.5 L/day** is likely the *biggest driver* of ongoing stone formation.

**2. Key Dietary Modifications (Top 3 Priorities)**

**Priority #1 — Increase Fluid Intake to Reach ≥2.0–2.5 L Urine/Day**

This is the **single most effective intervention** (40–60% recurrence reduction).

- Target **~2.5–3.0 L fluid intake/day** to achieve ≥2.0 L urine.
- Spread fluids evenly, especially **afternoons and evenings**, when most people crystallize.

**Priority #2 — Reduce Sodium Intake to <2 g/day (≈5 g salt/day)**

Borderline hypercalciuria is **most often sodium-driven**, not calcium-driven.

- Every **1 g of sodium** excreted drags out **~25 mg of calcium** in urine.
- Cutting sodium can drop urinary calcium **by 20–40%**.

**Focus on:**

- Avoiding processed foods, fast food, deli meat, soups, packaged snacks.
- Reading labels: choose items with **<5% DV sodium per serving**.

**Priority #3 — Maintain Normal Dietary Calcium (1000–1200 mg/day) With Meals**

Do **not** restrict calcium.
Normal intake binds oxalate in the gut → reduces oxalate absorption → lowers CaOx supersaturation.

Best strategy:

- Divide intake across meals → reduces oxalate spikes more effectively.

**3. Sample Menu Suggestions**

**Foods to Prioritize**

- **Hydration:** Water, herbal tea, infused water with lemon (adds citrate).
- **Calcium-with-meals approach:**
  - Breakfast: Greek yogurt + berries
  - Lunch: Chickpea salad + a small portion of cheese
  - Dinner: Vegetable stir-fry + tofu or a small glass of milk
- **Low-Sodium, Whole Foods:**
  - Fresh meats instead of deli meats
  - Fresh vegetables instead of canned (unless no-salt-added)
  - Homemade soups instead of packaged ones
- **Citrate-rich fruits:** Lemon, lime, melon

**Foods to Limit**

- **High-sodium foods:**
  Pretzels, chips, ramen, canned soups, restaurant meals, pickles, soy sauce
- **High-oxalate foods (don’t eliminate, just moderate & pair with calcium):**
  Spinach, almonds, beets, rhubarb, Swiss chard
- **Excess animal protein:**
  Large portions of beef, poultry, fish (>1.5 g/kg/day) → increases calcium & reduces citrate.

**4. Safety Warning**

If you have **heart failure, advanced CKD, or are on fluid-restricted medical therapy**, you must **not** increase fluid intake without clinician approval.
(If you tell me your comorbidities, I will adjust these recommendations safely.)

**Bottom Line**

Your labs *do not need to look severely abnormal* for stones to form.
With a urine volume of **1.5 L/day** and borderline high calcium, the **first and most impactful targets** are:

1. **Increase fluids (to ≥2.0–2.5 L urine/day)**
2. **Cut dietary sodium**
3. **Keep normal calcium intake with meals**

**Std + QB:**

Here’s the **realistic, highest-yield starting point** based on what you shared. This is **general educational guidance**, not personal medical advice.

**⭐ Your Top Priorities to Reduce Calcium Oxalate Stone Recurrence**

Even with only borderline-high urine calcium and otherwise not severely abnormal labs, **two factors stand out as the most impactful to focus on first**:

**1. Increase your urine volume (your #1 lever)**

Your current urine volume: **1.5 L/day**
Target for stone prevention: **≥ 2.0–2.5 L/day**

**Why this matters most**

Low volume is one of the strongest, most consistent risk factors for stone formation. Even modest increases reduce supersaturation of calcium oxalate.

**Practical approach**

- Aim for **2.5–3.0 L of fluid intake/day** to produce ≥2.0 L urine.
- Spread intake through the day (large evening loads may not help overnight).
- Water is best, but you can include:
  - Citrate-containing beverages (e.g., lemon water)
  - Herbal teas
  - Diluted juices in moderation

This single action provides more benefit than most dietary tweaks **combined**.

**2. Maintain normal dietary calcium (don’t restrict it)**

Your urine calcium is **borderline high**, not severely elevated. Restricting dietary calcium is *not* recommended.

**Why**

Low dietary calcium → more oxalate absorption in the gut → **increased calcium-oxalate stone risk**.

**What to do**

- Aim for **1,000–1,200 mg/day** from food.
- Pair calcium-containing foods *with meals that contain oxalate* to bind oxalate in the gut.
  - Examples: yogurt + berries, cheese + spinach-containing meals.

**3. Reduce sodium (this directly lowers urine calcium)**

Even borderline-high urine calcium is strongly influenced by dietary sodium.

**Why this matters**

High sodium intake → kidneys excrete more calcium → higher supersaturation.

**Goal**

- **< 2,000 mg sodium/day** (some guidelines allow <2,300 mg).

**Practical swaps**

- Avoid salty snacks, fast foods, soups, processed meats.
- Choose fresh over packaged foods.
- Read labels: aim for <5% daily value sodium per serving when possible.

**4. Moderate animal protein intake**

Not as urgent as fluid or sodium, but still meaningful.

**Why**

Animal protein increases:

- Calcium excretion
- Acid load → lowers urinary citrate
- Uric acid levels

**Target**

- **0.8–1.0 g/kg/day** unless you have specific medical dietary needs.

**5. Oxalate reduction (only after the above are optimized)**

Since your urine abnormalities are mild, this is **not** the first priority—only adjust oxalate if you still form stones despite improving volume, sodium, and diet.

Higher-oxalate foods include spinach, almonds, beets, rhubarb, peanuts, potatoes, and dark chocolate.

**But do not cut oxalate without ensuring adequate dietary calcium**, or it may worsen absorption.

**🔑 If you had to choose just *one* thing to start:**

**Increase your urine volume to ≥2.0 L/day.**

This is the simplest, safest, and most effective initial step.

**🔑 If you choose two things:**

1. Increase urine volume.
2. Lower dietary sodium.

These directly and reliably lower stone risk *even when labs are only borderline abnormal*.

**Case21:**

**SA + QA:**

Below is a **heart-failure-safe, guideline-based metabolic plan** tailored to your uric-acid stone disease and very low urine pH (5.0).

**1. Metabolic Diagnosis**

- **Uric acid stone disease driven by persistently low urine pH (5.0)** — the *primary* risk factor for uric-acid stones per EAU & CUA guidelines.
- **Hyperuricosuria (high urine uric acid)** likely exacerbated by excess dietary purines, high animal protein load, and insulin resistance physiology common at age 72.
- **Fluid intake must be restricted carefully** due to **congestive heart failure (CHF)**—large-volume hydration is unsafe.

**2. Key Dietary Modifications (Heart-Failure-Safe)**

**Priority #1 — Raise urine pH through more alkali-producing foods (NOT large fluid loads)**

- Emphasize **vegetables and low-potassium fruits**, but choose *heart-failure-safe potassium levels* depending on your serum potassium (not provided).
- Use **dietary alkali** from food (not supplements unless prescribed):
  - Good: cauliflower, cabbage, zucchini, bell peppers, apples, berries, peaches.
  - Avoid excessive high-K foods (e.g., avocado, spinach, bananas) *unless cleared by cardiology*.

**Priority #2 — Reduce dietary purines to lower urine uric acid**

- Limit **red meats**, **organ meats**, **sardines**, **anchovies**, **shellfish**, and **beer**.
- Prefer **plant proteins** and **white meats** (e.g., skinless chicken or turkey) in moderate portions.

**Priority #3 — Maintain a heart-failure-appropriate sodium restriction (≤2 g/day)**

- This helps prevent fluid overload **and** lowers stone risk by reducing renal acid load and improving urine pH.
- Avoid canned soups, deli meats, salted snacks, sauces, fast foods.

**3. Sample Menu Suggestions (Targeting Low pH + High Uric Acid + CHF)**

**Breakfast**

- Oatmeal cooked in water with blueberries and a sprinkle of almonds.
- Herbal tea or water (small volume as allowed by CHF fluid limits).

**Lunch**

- Grilled chicken breast (3–4 oz).
- Steamed zucchini and bell peppers with olive oil and herbs (no added salt).
- Small baked potato *if potassium is safe*; otherwise substitute white rice.
- Sparkling water or regular water within fluid allowance.

**Dinner**

- Lentil or bean-based vegetable stew (low sodium).
- Side of cabbage or roasted cauliflower.
- Fresh fruit: apple slices or berries.

**Foods to Avoid or Strictly Limit**

- **High-purine meats/fish:** beef, lamb, pork, sardines, anchovies, organ meats.
- **Alcohol**, especially **beer** (high purines, lowers urine pH).
- Sugary beverages (increase uric acid production).
- High-sodium foods (worsen CHF and stone risk).

**Heart-failure-compatible ways to raise urine pH**

- Increase the *proportion* of vegetables and low-purine plant foods without increasing overall fluid intake.
- If prescribed, **potassium citrate** or **sodium bicarbonate** can raise urine pH, but in CHF **sodium-based alkalinizers must be used cautiously**—usually avoided unless cardiology approves.

**4. Safety Warning**

- **Fluid restriction applies.** Standard stone-prevention hydration goals **do NOT apply in CHF**. Increase urine volume only within cardiology-approved limits.
- **Potassium-rich alkalinizing foods must be tailored to your serum potassium.** Some HF medications (e.g., ACE inhibitors, ARBs, spironolactone) can cause hyperkalemia.
- **Avoid sodium bicarbonate supplementation** unless your cardiologist specifically approves—it may worsen fluid retention and blood pressure.
- **Do not make major potassium or fluid changes without reviewing labs (creatinine, potassium, bicarbonate) and heart-failure medications.**

**Std + QA:**

Here is **safe, general, non-individualized dietary guidance** that aligns with what is typically recommended for **uric acid stone prevention** *and* is appropriate for someone with **congestive heart failure (CHF)**, where fluid overload and sodium restriction are major concerns.

Because I cannot give personalized medical advice, use this as educational information to discuss with your clinician.

**⭐ Dietary Strategies to Prevent Uric Acid Stones Without Worsening CHF**

**1. Raise urine pH safely through diet**

Uric acid stones form in **acidic urine**, and your reported **pH of 5.0** is strongly acid. A goal pH is usually **≥6.0–6.5** (your clinician will set the target).

You can support urine alkalinization with **low-sodium, heart-failure–friendly foods**:

**Foods that naturally alkalinize urine**

- Vegetables: spinach, kale, broccoli, cauliflower, cucumbers, squash, carrots
- Fruits: lemon/lime, oranges, melons, berries, bananas
- Legumes (in moderate amounts): lentils, chickpeas, beans
- Nuts (small portions due to calories): almonds, walnuts

**Why this works**

These foods increase the alkaline load to the kidneys and raise urine pH **without requiring high fluid intake** or sodium bicarbonate (which is *not* appropriate in CHF because it contains sodium).

**2. Limit purine-rich foods to reduce uric acid formation**

High urine uric acid indicates your body is making/excreting more uric acid.

Avoid or minimize:

- **Red meat** (beef, lamb, pork)
- **Organ meats** (liver, kidney, sweetbreads)
- **Shellfish** (shrimp, lobster, crab)
- **Sardines, anchovies, mackerel**
- **Beer and alcohol** (beer is especially high-purine and worsens acidosis)

Use instead:

- Skinless poultry in moderate amounts
- Plant-based proteins (beans, tofu)
- Heart-healthy fish like salmon (moderate portions)

**3. Follow a *low-sodium* diet (essential for heart failure)**

High sodium → fluid retention → worsened CHF → prevents adequate urine dilution.

Most CHF guidelines recommend **≤1,500–2,000 mg sodium/day**.

Tips:

- Avoid canned soups, deli meats, salted snacks, packaged frozen meals
- Cook with herbs, lemon, garlic instead of salt
- Choose “low-sodium” or “no-salt-added” products

This also supports stone prevention because sodium increases uric acid stone risk indirectly.

**4. Fluid intake — adjust for CHF**

Typical kidney stone advice (“drink 2–3 L/day”) **does NOT apply** to heart failure without clinician approval.

Better approach:

- Follow **your cardiologist’s fluid restriction** strictly
- Spread fluids evenly throughout the day
- Use alkaline beverages **within your fluid limit**, such as:
  - Water with a squeeze of lemon or lime
  - Fruit-infused water
  - Low-sugar citrus beverages

Avoid:

- Sugary sodas
- High-fructose juices (increase uric acid production)

**5. Limit fructose and sugary drinks**

Fructose increases uric acid production in the liver.

Avoid or restrict:

- Regular soda
- Sweetened teas
- Fruit punch
- Desserts with high fructose corn syrup

**6. Weight management and diabetes control (if relevant)**

Insulin resistance promotes acidic urine. A diet higher in vegetables, low in refined carbs, and moderate in lean protein helps shift urine pH upward.

**7. Potassium-rich foods help alkalinize urine—but check with your doctor**

Many alkalinizing fruits/vegetables are high in potassium, which may be restricted in CHF patients **on certain medications (e.g., ACE inhibitors, ARBs, spironolactone).**

Safe step:

- Ask your doctor whether you **should limit or monitor potassium** before adding large amounts of fruits/vegetables.

**Summary Table**

| **Goal** | **CHF-Safe Dietary Action** |
| --- | --- |
| Raise urine pH | More fruits/vegetables; lemon/lime water |
| Reduce uric acid | Limit purine-rich meats and alcohol |
| Avoid fluid overload | Follow prescribed fluid restriction |
| Avoid worsening CHF | Strict sodium restriction |
| Avoid high potassium excess | Verify potassium limits with clinician |

**SB + QB:**

Below is a **CHF-safe**, guideline-based uric-acid–stone plan following the exact structure you requested.

**1. Metabolic Diagnosis**

The patient has **uric acid stone disease due to severe urinary acidification (urine pH 5.0)** and **hyperuricosuria**.
Low urine pH—not urine volume—is the primary driver of uric acid stone formation, because uric acid crystallizes readily when pH < 5.5. Excess dietary purines and metabolic acid load further worsen this.

Because you have **congestive heart failure (CHF)**, aggressive fluid loading is **contraindicated**.

**2. Key Dietary Modifications (Top 3 Priorities)**

**1. Raise urine pH safely (without increasing fluid volume excessively)**

- Increase intake of **alkali-producing fruits and vegetables** that are *low in sodium* (e.g., berries, citrus, melon, bananas, leafy greens).
- Prefer **potassium-citrate–rich foods**, *but within CHF potassium limits*.
- Discuss with your cardiologist whether **potassium citrate medication** is safe—this is the most effective therapy for uric acid stones, but may be restricted in CHF depending on potassium levels and ejection fraction.

**2. Reduce dietary purines to lower uric acid production**

- Restrict **red meat**, **organ meats**, **anchovies**, **sardines**, **shellfish**, and **high-purine broths/gravies**.
- Limit **alcohol**, especially **beer** and **spirits**, which lower urine pH and raise uric acid.

**3. Sodium restriction tailored to CHF**

- Target **<2 g sodium/day** (typical CHF target).
- Low sodium improves stone prevention indirectly by reducing acid load and improving the effect of alkali therapy.

**3. Sample Menu Suggestions**

**Foods to Prioritize**

**Alkali-producing, low-sodium, low-purine**

- Fresh fruits: **berries, melon, peaches, apples, bananas, oranges, kiwi**
- Vegetables: **spinach, kale, broccoli, zucchini, carrots, cauliflower, bell peppers**
- Plant proteins: **lentils (small portions), tofu, edamame, unsalted nuts**
- Grains: **oats, brown rice, barley, quinoa**
- Dairy: **low-fat milk or yogurt** (helps buffer acid load)
- Beverages:
  - **Citrate-containing drinks** such as *diluted* lemon water (without excess fluid volume)
  - Avoid sugary versions and keep total fluid to the CHF-approved daily allotment.

**Sample Daily Pattern (assuming CHF-restricted fluid total)**

- **Breakfast:** Oatmeal with berries; small glass of low-fat milk
- **Lunch:** Grilled chicken breast (small portion), quinoa, steamed broccoli
- **Snack:** Apple or unsalted nuts
- **Dinner:** Baked salmon (moderate portion), roasted vegetables (zucchini + peppers), brown rice
- **Drink throughout the day:** Your cardiologist-approved fluid allotment, flavored with lemon/lime to raise citrate intake.

**Foods to Avoid**

**High-purine sources that worsen uric acid stones**

- Organ meats: liver, kidney, sweetbreads
- Gravies, broths, bone-marrow soups
- Sardines, anchovies, mussels, scallops
- Large portions of red meat
- Excess poultry skin and dark meats

**Foods that acidify urine**

- High-protein ketogenic diets
- Excess animal protein (>0.8–1.0 g/kg/day)
- Alcohol (especially beer and spirits)

**Foods harmful for CHF**

- High-sodium foods: canned soups, salted snacks, processed meats, restaurant meals
- Sodium bicarbonate (“baking soda”) self-alkalinization—**contraindicated** due to sodium load.

**4. Safety Warning (CHF-specific)**

Because you have **congestive heart failure**, the following precautions are essential:

**Fluid Intake**

- Do **NOT** increase fluid intake beyond what your cardiologist permits.
- Uric acid stone prevention can be achieved primarily through **urine alkalinization**, not forced hydration.

**Potassium-containing foods and supplements**

- High-potassium strategies (**potassium citrate**, large amounts of citrus, bananas, etc.) may be unsafe if:
  - you are on **ACE inhibitors**, **ARBs**, or **spironolactone**, or
  - you have reduced kidney function.
- Always confirm with cardiology before increasing potassium intake or starting alkali therapy.

**Avoid sodium-based alkalinizers**

- Products like **baking soda, Alka-Seltzer, sodium citrate drinks** can worsen fluid retention and CHF exacerbations.

**Std + QB:**

I can give **general, educational nutrition guidance** for people with **uric-acid kidney stones and fluid restrictions due to CHF**, but I **cannot give personalized medical treatment instructions**. Please review any changes with your cardiologist and nephrologist/urologist, because CHF adds important safety considerations.

**⭐ What Makes Uric-Acid Stones Form?**

- **Acidic urine (pH < 5.5)** → uric acid crystallizes.
- **High uric acid levels** (from diet, metabolism, or medications).
- **Low urine volume**, but in CHF you *cannot* simply increase fluids freely.

So the safest approach focuses on **alkalinizing the urine with food choices**, **reducing uric-acid–producing foods**, and **keeping sodium and fluid intake safe for CHF**.

**✔ Safest Nutrition Priorities (Considering Uric Acid Stones + CHF)**

**1. Follow your cardiologist’s fluid and sodium limits**

This is the *top safety rule*.

- Many CHF patients must stay around **1.5–2 L/day of fluid**, sometimes less.
- **Sodium usually ≤ 2 g/day**, sometimes lower if recommended for your case.

Lower sodium helps prevent fluid retention and reduces stone risk indirectly by improving kidney function.

**2. Increase foods that *alkalinize* the urine (raise pH)**

Your urine pH of **5.0** is very acidic; uric-acid stones dissolve and stop forming when pH is ~6.0–6.5.

**Helpful alkalinizing foods:**

- Most **fruits** (especially citrus):
  - lemons, limes, oranges, strawberries, melons
  - (Note: *oranges* contain potassium—confirm safety if you are on ACE inhibitors/ARBs or spironolactone.)
- Most **vegetables**, especially:
  - leafy greens, cauliflower, broccoli, cucumbers, carrots, squash, potatoes
- **Legumes** (beans, lentils) — also lower-purine than meats
- **Whole grains** (oats, brown rice, barley)

**Use lemon or lime juice in your limited fluid allowance**; it helps raise urine pH without adding sodium.

**3. Reduce high-purine foods that raise uric acid**

Purines → uric acid. Limiting them reduces stone risk.

**Avoid or strictly limit:**

- **Red meats**: beef, lamb, pork
- **Organ meats**: liver, kidney, sweetbreads (very high purine)
- **High-purine seafood**: anchovies, sardines, shellfish, mussels, herring, mackerel
- **Alcohol**, especially **beer** and **spirits**
- **High-fructose corn syrup** (found in sodas, many processed foods) → raises uric acid sharply

**Choose instead:**

- **Poultry** or **plant-based proteins** (beans, tofu)
- **Low-purine seafood**: salmon, cod, trout (still moderate—watch portions)

**4. Adopt a “heart + kidney friendly” protein intake**

Excess protein increases uric acid and acid load.

- Most patients do best with **moderate**, not high, protein intake.
- Shift toward **plant-forward meals** (lentils, beans, tofu) when possible.
- If you have **renal insufficiency**, protein needs vary—confirm with your clinician.

**5. Limit foods that make urine more acidic**

These work against you when trying to raise urine pH.

Avoid excessive intake of:

- Large amounts of **animal protein**
- **Soda**, especially cola
- **Highly processed foods**
- **Sugary desserts** (fructose → uric acid)

**6. Potassium considerations**

Many alkalinizing foods are potassium-rich. This is helpful for pH, **but may be unsafe in CHF** if you take:

- ACE inhibitors (e.g., lisinopril)
- ARBs (e.g., losartan)
- Aldosterone blockers (e.g., spironolactone)

**Do not increase potassium-containing foods aggressively without confirming with your provider.**

**7. Weight management and metabolic syndrome control**

If applicable:

- A **Mediterranean-style diet** (low-sodium, plant-forward) helps reduce uric acid.
- Controlling insulin resistance lowers uric acid production.

**🚫 What to Avoid (Summary)**

- High-purine meats (red meat, organ meats)
- High-purine seafood (sardines, anchovies, shellfish)
- Alcohol—especially beer & hard liquor
- High-fructose corn syrup (sodas, sweetened drinks)
- Excess animal protein
- Excess sodium (worsens CHF and stone formation)
- Drinking beyond your CHF fluid limit
- Over-the-counter supplements that alter electrolytes without supervision

**✔ Sample “Safe” Meal Pattern (Needs doctor approval)**

- **Breakfast:** Oatmeal with berries + small amount of nuts
- **Lunch:** Lentil soup (low sodium), salad with lemon vinaigrette
- **Dinner:** Small portion of grilled chicken or tofu + steamed vegetables + brown rice
- **Fluids:** Water within your CHF limit, flavored with lemon or lime

**📌 Important**

**Raising urine pH often requires medical therapy (e.g., potassium citrate or sodium bicarbonate).**
Because you have CHF, these medications—especially sodium-based ones—must be chosen carefully and prescribed by your doctor.

**Case22:**

**SA + QA:**

Below is your structured, guideline-aligned answer.

**1. Metabolic Diagnosis**

Your **24-hour urine oxalate of 0.7 mmol/day (~62 mg/day)** is **normal**, so you do **not** have hyperoxaluria.
Your primary risk for recurrent calcium oxalate stones in the context of **stage 4 CKD (eGFR 25)** comes from **reduced ability to excrete acid and manage mineral balance**, not from oxalate excess.

Thus the correct metabolic interpretation is:

**“Calcium oxalate stone disease in the setting of advanced CKD without hyperoxaluria; dietary strategy must prioritize kidney-safe measures over traditional stone-reduction diets.”**

**2. Key Dietary Modifications (Top 3 Priorities)**

**1. Maintain normal calcium intake with meals (1,000–1,200 mg/day)**

- Prevents intestinal oxalate absorption
- Use **dietary calcium**, not supplements (unless prescribed).

**2. Moderate—not excessive—fluid intake**

- In CKD stage 4, aggressive high-fluid stone prevention is **dangerous**.
- Follow **nephrologist-set fluid goals**, often **1.5–2.0 L/day maximum**, adjusted for volume status.

**3. Reduce dietary acid load + sodium restriction**

- **Low sodium (<2 g/day sodium = <5 g salt)** lowers urine calcium and helps blood pressure/CKD progression.
- A **plant-forward, low-animal-protein diet** reduces acid load and improves citrate levels **without increasing potassium dangerously**—but must be done with careful potassium selection (see menu).

**3. Sample Menu Suggestions**

**Foods to Emphasize (Kidney-safe + stone-safe)**

✔ **Low-oxalate vegetables:** cauliflower, cabbage, zucchini, mushrooms, green beans
✔ **Lower-potassium fruits:** apples, berries, grapes, pineapple
✔ **Calcium sources with meals:**

- ½ cup calcium-fortified plant milk
- ½ cup low-potassium yogurt alternatives
  ✔ **Moderate plant proteins & CKD-safe proteins:**
- Egg whites
- Tofu (in moderate portions)
- White fish or chicken, small servings (2–3 oz)
  ✔ **Whole grains with lower potassium/phosphate:** white rice, couscous, pasta
  ✔ **Healthy fats:** olive oil, avocado oil (limit avocado due to high potassium)

**Foods to Limit or Avoid**

✖ **High-oxalate foods** (even though your oxalate is normal, CKD limits your ability to correct increases):
spinach, almonds, beets, sweet potatoes, peanuts, rhubarb, Swiss chard
✖ **High-potassium foods** (due to CKD stage 4):
bananas, oranges, tomatoes, potatoes, avocados
✖ **High sodium:** processed meats, canned soups, fast food
✖ **High animal protein:** red meat, large portions of poultry/fish
✖ **Soda/cola** (phosphate additives accelerate CKD progression)

**Example 1-Day Menu (CKD- & Stone-Safe)**

**Breakfast**

- Oatmeal made with water + ½ cup calcium-fortified low-K plant milk
- Blueberries
- Hard-boiled egg whites
- Herbal tea

**Lunch**

- Grilled chicken (2–3 oz)
- White rice or couscous
- Steamed green beans
- Apple slices

**Dinner**

- Baked white fish (cod/tilapia)
- Sauteed zucchini
- Pasta with olive oil & herbs
- Small portion of berries

**Snacks**

- Low-sodium crackers
- Yogurt alternative (calcium fortified, low potassium)

**4. Safety Warning**

Because you have **stage 4 CKD**, the following precautions are essential:

**⚠ Fluid intake must NOT be increased without nephrology approval.**

Overhydration can cause **fluid overload, pulmonary edema, and hypertension** in CKD.

**⚠ Potassium-rich plant foods must be limited.**

Well-known stone-prevention diets (e.g., DASH) are **unsafe** for eGFR 25 without potassium monitoring.

**⚠ Avoid calcium supplements unless specifically prescribed.**

They raise calcium load and may accelerate vascular calcification in CKD.

**⚠ Any major diet changes should involve your nephrologist and renal dietitian.**

**Std + QA:**

I can give **general, safety-oriented dietary principles** for someone with **calcium oxalate stones** *and* **stage 4 chronic kidney disease (eGFR ≈25)**—but this **cannot replace individualized guidance** from your nephrologist or renal dietitian, who must tailor recommendations based on potassium, phosphorus, calcium levels, protein needs, and your overall medical picture.

Because CKD stage 4 significantly changes what is *safe*, stone-prevention advice must be modified.

**✅ Most Important Principles for You**

**1. Maintain adequate—but not excessive—fluid intake**

- Hydration lowers stone risk, but CKD limits how much you can safely drink.
- Typical stone-prevention targets (2–2.5 L/day urine) are **not appropriate** in advanced CKD.
- **Follow your nephrologist’s fluid recommendations** exactly—often a *moderate* fluid intake to avoid volume overload.

****2. Keep normal dietary calcium (do NOT restrict calcium unless instructed)**

For calcium oxalate stones:

- Normal calcium intake (≈800–1,000 mg/day, depending on labs) **reduces** oxalate absorption.
- Low calcium diets **increase oxalate** and worsen stone risk.

Safe calcium sources for CKD often include:

- Controlled amounts of dairy (if potassium/phosphorus allow)
- Calcium-containing binders if prescribed
  *Only your kidney team can specify amounts based on your labs.*

****3. Moderate your oxalate intake, but do not eliminate it entirely**

Your urine oxalate (0.7 mmol/day ≈ moderate) is **not extremely high**, so:

- A **moderate** oxalate reduction is safest.
- Extremely low-oxalate diets can worsen nutrition and are unnecessary.

Common high-oxalate foods to **limit**, not ban:

- Spinach, beets, almonds, peanuts, rhubarb, Swiss chard
- Bran cereals, wheat germ
- Dark chocolate
- Tea in large quantities

You do **not** need to avoid all vegetables.

**4. Pair calcium-containing foods with oxalate-containing foods**

This reduces oxalate absorption in the gut.

Example:

- Eating a small serving of milk, yogurt, or calcium-fortified rice milk with a meal that contains moderate oxalate.

This strategy is **safe for CKD** when calcium intake is supervised.

**5. Sodium restriction is especially important for CKD + stones**

High sodium increases both:

- Urinary calcium (stone risk)
- CKD progression and blood pressure

Target (unless your nephrologist says otherwise):

- **<2,000 mg sodium/day**

Avoid:

- Processed meats
- Canned soups
- Fast food
- Salty snacks
- High-sodium seasonings

**6. Protein intake: avoid excess**

High animal protein increases acid load and stone risk, and burdens kidneys.

For stage 4 CKD, dietitians commonly recommend:

- **0.6–0.8 g/kg/day**, depending on your weight, nutritional status, and potassium levels.

Prefer:

- Plant proteins (beans, lentils) **only to the extent that potassium allows**
- Fish or poultry in moderate amounts

Do **not** follow high-protein diets (keto, carnivore, heavy meat intake).

**7. Potassium: adjust based on your labs**

Many stone-prevention diets (e.g., high fruit & vegetables, high citrate) assume normal kidney function, but **CKD stage 4 often requires potassium restriction**.

Your kidney team must guide this, but in general:

- If potassium tends to run **high**, limit bananas, oranges, potatoes, tomatoes, spinach, dried fruits.
- If potassium is normal, moderate intake of fruits/vegetables is beneficial.

**8. Citrate matters, but supplements may not be safe**

Citrate prevents stones, but:

- **Potassium citrate** (a common stone medication) may be **unsafe** in advanced CKD due to risk of hyperkalemia.
- **Lemon or lime juice** as a citric acid source is usually safer but must be evaluated for potassium content and fluid allowance.

**9. Avoid high-dose vitamin C**

Vitamin C converts to oxalate.

- Keep vitamin C intake **<200 mg/day** unless your doctor advises otherwise.
- Avoid high-dose supplements.

**✔️ Summary: The Safest General Diet Pattern for You**

If your labs allow it, the diet that usually balances CKD and stone needs is:

**A CKD-appropriate, moderate-oxalate, low-sodium, controlled-protein diet**

- Fluids: Only as allowed by your nephrologist
- Calcium: Normal daily intake with meals
- Oxalate: Moderate—not low—restriction
- Sodium: Low (<2 g/day)
- Protein: Controlled (0.6–0.8 g/kg/day)
- Potassium: Based on your lab levels
- Citrate: From diet if safe; supplements only with medical approval
- Avoid high-dose vitamin C

**⚠️ What to avoid because you have both CKD stage 4 and calcium oxalate stones**

- Over-restriction of fluids
- Low-calcium diets
- High-oxalate foods in large quantities
- High-protein diets
- High-sodium foods
- Potassium-rich foods *if your labs run high*
- Potassium citrate supplements unless cleared
- High-dose vitamin C

**SB + QB:**

Below is an evidence-based, CKD-adjusted interpretation that *avoids unsafe standard stone-prevention advice* (such as high fluid, high-alkali, or high-potassium diets) and follows EAU/CUA principles.

**1. Metabolic Diagnosis**

**You have hyperoxaluria** (urine oxalate 0.7 mmol/day ≈ 62 mg/day, above the typical target <40–45 mg/day) contributing to **calcium oxalate stone formation.**
Because your **eGFR is 25 (CKD stage 4)**, several standard kidney-stone recommendations (e.g., very high fluids, high-potassium citrate foods, unlimited dairy) must be modified for safety.

**2. Key Dietary Modifications (CKD-SAFE Adjustments)**

**Priority 1 — Reduce Oxalate Load WITH normal dietary calcium**

- Keep **dietary calcium at ~800–1000 mg/day**, *taken with meals* to bind oxalate in the gut.
- Focus on calcium from **low-phosphorus, CKD-appropriate sources** (e.g., calcium-fortified plant milks without added phosphate, controlled portions of dairy if phosphorus is managed).
- Avoid high-oxalate foods (*spinach, beet greens, almonds, peanuts, rhubarb, Swiss chard, sweet potatoes, bran cereals, matcha/black tea in excess, dark chocolate*).

**Priority 2 — Avoid Hidden Oxalate Amplifiers**

- Limit **vitamin C supplements** above 200 mg/day (they convert to oxalate).
- Avoid **very low calcium diets**, which increase oxalate absorption.
- Moderate **animal protein** intake (excess protein → low citrate & higher acid load → more CaOx risk).

**Priority 3 — CKD-Compatible Fluid Strategy**

- With eGFR 25, **standard stone advice of “drink 3 L/day” may NOT be safe**.
- Fluid intake must be individualized based on cardiology/nephrology guidance.
- General CKD-safe approach: maintain **steady hydration**, avoid large boluses, and avoid dehydration, but do *not* push high volumes unless cleared by your nephrologist.

**3. Sample Menu Suggestions (CKD-Adjusted)**

**Foods to Prefer**

- **Low-oxalate vegetables:** cabbage, cauliflower, cucumbers, zucchini, mushrooms, iceberg or romaine lettuce, green beans.
- **Calcium sources taken with meals (to bind oxalate):**
  - Calcium-fortified oat or rice milk (without phosphate additives).
  - Small portions of lower-phosphorus dairy (e.g., ½ cup yogurt) if phosphorus is controlled.
  - Calcium supplements *only if approved by nephrology* (commonly calcium carbonate with meals for CKD patients).
- **Lower-potassium fruits** (important because CKD stage 4 limits high-K options): apples, grapes, pineapple, berries.
- **Proteins:** limit to CKD targets. Focus on fish, poultry, or plant proteins in controlled portions.
- **Fats & grains:** olive oil, white rice, pasta, low-oxalate breads and cereals.

**Meal Example (CKD-Safe, Low Oxalate)**

- **Breakfast:** Oatmeal made with calcium-fortified oat milk; blueberries; herbal tea.
- **Lunch:** Grilled chicken with white rice, zucchini, and a side of lettuce salad.
- **Dinner:** Baked white fish, cauliflower mash, green beans.
- **Snacks:** Low-oxalate fruits (grapes, apples); rice crackers; cucumber slices.

**Foods to Avoid or Strictly Limit**

**Highest oxalate offenders**

- Spinach, chard, beet greens
- Almonds & almond flour, peanuts, cashews
- Sweet potatoes
- Rhubarb
- Bran cereals, wheat bran
- Soy products (tofu, edamame—moderate to high oxalate)
- Dark chocolate, cocoa powder
- Black tea & green tea concentrates (matcha especially high)

**CKD-specific restrictions**

- **High-potassium stone-prevention foods** often recommended for citrate boosting
  - e.g., bananas, oranges, potatoes, tomatoes, avocados → **not safe in CKD stage 4**
- **Overuse of dairy** for calcium binding may worsen **phosphorus** and **PTH**.

**4. Safety Warning (Critical for CKD Stage 4)**

**⚠️ High fluid intake is NOT automatically safe.**

Most stone guidelines advise >2.5 L/day urine output, but in CKD stage 4 this can cause:

- Volume overload
- Worsening hypertension
- Heart failure exacerbation

Your nephrologist must determine your **personalized fluid allowance**.

**⚠️ High-potassium “citrate-rich foods” are dangerous.**

Common stone advice (orange juice, coconut water, melon, high-K fruits, potassium citrate supplements) can cause **life-threatening hyperkalemia** in CKD4.

**⚠️ OTC potassium citrate is unsafe without nephrology oversight.**

**⚠️ Beware phosphorus overload.**

Many dairy products and processed foods contain **phosphate additives** that worsen CKD progression and mineral bone disease.

**Std + QB:**

Below is **general educational guidance only** — NOT medical advice.
Because you have **advanced CKD (eGFR ~25)** *and* **calcium oxalate stones**, your dietary strategy must carefully balance stone-prevention principles with kidney-disease safety. Some standard stone recommendations can actually be harmful in CKD. You must confirm any changes with your nephrologist and renal dietitian.

**✅ What You *Should* Focus On**

**1. Maintain a *moderate*, not high, calcium intake**

- Calcium binds oxalate in the gut → lowering absorption and urinary oxalate.
- In CKD, **you should NOT raise calcium excessively**, because:
  - The kidneys cannot excrete calcium well.
  - High calcium intake can worsen vascular calcification and hypercalcemia.
- **Target (if your provider agrees):** ~600–800 mg/day dietary calcium from food, not supplements.
  - Prefer **calcium-containing foods eaten with meals**, such as:
    - Small portions of dairy (⅓ cup yogurt, 1 oz cheese, ½ cup milk — only if allowed by your CKD plan)
    - Calcium-fortified plant milks (low potassium and phosphate versions)

**❌ Avoid:**

- **Calcium supplements**, unless specifically instructed by your nephrologist. These significantly increase stone risk and may worsen CKD-mineral bone disease.

**2. Reduce dietary oxalate to lower urinary oxalate**

Your urinary oxalate (0.7 mmol/day ≈ 62 mg/day) is elevated.
High-oxalate foods to limit or avoid:

**Very high oxalate foods: avoid**

- Spinach
- Almonds, cashews, peanuts
- Beets, beet greens, swiss chard
- Rhubarb
- Soy foods (tofu, soy milk)
- Bran cereals, wheat bran
- Dark chocolate / cocoa powder
- Sweet potatoes
- Black tea (heavy intake)

**Moderate oxalate foods: limit portions**

- Potatoes
- Berries
- Whole grains
- Lentils and beans (also limited in CKD due to potassium/phosphate)

**3. Keep sodium low (good for BOTH stones and CKD)**

- Target: **≤2 g sodium/day** (≈ 5 g salt)
- High salt increases:
  - Calcium excretion → more stones
  - Blood pressure → worsens CKD

**4. Moderate animal protein intake**

This helps reduce stone risk **and** protects kidney function.

**Guidelines:**

- Avoid high-protein diets.
- Emphasize plant protein where possible — but be careful since many plant proteins (legumes, nuts) are high in potassium/phosphate or oxalate.

**A renal dietitian can help blend these restrictions safely.**

**5. Fluid intake: You may NOT be able to follow typical stone advice**

Normally, calcium stone patients are told to drink **2.5–3 L/day**.
**With eGFR 25, fluid recommendations depend on:**

- Your blood pressure
- Edema
- Heart function
- Urine output

⚠️ **If you have fluid retention or heart issues, high fluid intake may be dangerous.**
You MUST get individualized guidance from your nephrologist.

General safe principle:

- Drink enough to keep urine *light yellow*, but **not force fluids without clearance**.

**6. Potassium restrictions complicate typical stone diets**

Many low-oxalate plant foods are high in potassium.
With CKD, you may need to **avoid or limit**:

- Avocado
- Potato / sweet potato
- Bananas
- Tomatoes
- Oranges
- Beans and lentils
- Nuts

(Your personal potassium blood level determines how strict this needs to be.)

**7. Vitamin C supplements are unsafe**

- High-dose vitamin C (>100 mg/day supplemental) → converts to oxalate → raises urinary oxalate
- CKD reduces clearance → even greater oxalate buildup

**Avoid vitamin C supplements unless prescribed.**

**8. Avoid high-dose vitamin D and calcium combo supplements**

These can increase calcium absorption and raise stone risk, and may worsen hypercalcemia in CKD.

**🚫 Common Stone-Prevention Advice That is NOT Safe for Advanced CKD**

| **Typical Stone Advice** | **Why It Can Be Unsafe in CKD (eGFR 25)** |
| --- | --- |
| **“Drink 3 liters daily.”** | Risk of fluid overload, hypertension, heart strain. |
| **High dietary calcium (1,200–1,500 mg/day)** | May cause hypercalcemia; CKD reduces excretion; risk of vascular calcification. |
| **Calcium supplements with meals** | Can worsen CKD-MBD and increase stone risk in CKD. |
| **Eat more fruits/vegetables to alkalinize urine** | Many are high in potassium → hyperkalemia risk. |
| **Nuts and nut butters as healthy snacks** | Very high oxalate and high potassium. |
| **Use potassium citrate freely** | Potassium citrate is often *contraindicated* in CKD → can cause dangerous hyperkalemia. |

**⭐ Foods Generally Safer for Both CKD and Calcium Oxalate Stones**

(*Always adjust based on your potassium and phosphate labs*)

**Lower potassium, lower oxalate vegetables**

- Cabbage
- Cauliflower
- Broccoli
- Green beans
- Zucchini
- Lettuce
- Cucumbers
- Bell peppers

**Lower oxalate, kidney-friendly fruits**

- Apples
- Grapes
- Pineapple
- Watermelon
- Peaches
- Berries (small portions; moderate oxalate)

**Proteins (moderate portions)**

- Skinless poultry
- Fish
- Eggs
- White rice-based dishes (low potassium/phosphate)

**Carbs**

- White rice
- Pasta
- White bread
- Tortillas
  (These are low oxalate and kidney-safe.)

**📌 Additional Strategies**

**Consider calcium-containing food with your highest-oxalate meal**

Even small amounts (½ cup dairy or fortified milk) can bind oxalate.

**Avoid ketogenic or high-protein diets**

They increase acid load and stone risk; harmful in CKD.

**Limit phosphate additives**

Processed foods with “phos-” ingredients worsen CKD progression.

**🔍 Summary of What You Should Do**

**Eat / Include**

- Moderate calcium *from food* with meals
- Low-oxalate vegetables and fruits
- Low sodium
- Moderate animal protein
- Controlled fluids (per nephrologist)

**Avoid**

- Spinach, nuts, chocolate, soy, beets
- High potassium foods (depending on labs)
- Vitamin C supplements
- Calcium supplements
- Potassium citrate unless approved
- Excess fluids

**Case23:**

**SA + QA:**

Below is a **pregnancy-safe, guideline-based stone-prevention plan** tailored to your metabolic data (high urine calcium + high urine sodium) *and* the unique physiology of pregnancy (where calcium handling and fluid/electrolyte balance differ significantly).

I will **not** recommend anything unsafe for pregnancy (e.g., calcium restriction, extreme fluid loading, supplements, or restrictive diets that impair fetal growth).

**1. Metabolic Diagnosis**

- **Hypercalciuria (elevated urinary calcium at 9.0 mmol/day)**
  In pregnancy, mild physiologic hypercalciuria is common due to increased intestinal calcium absorption, but your level is still *high enough to increase stone risk*.
- **High urinary sodium (150 mmol/day)**
  This is a major modifiable driver of urinary calcium loss even during pregnancy.

**Interpretation:**
Your symptomatic calcium oxalate stones are likely being worsened by **sodium-driven hypercalciuria**, not dietary calcium excess.

**2. Key Dietary Modifications (Pregnancy-Safe Priorities)**

**1. Reduce sodium intake to < 70–100 mmol/day (≈ 2–2.3 g sodium or 5–6 g salt/day)**

This is the most powerful and *safest* way to reduce urinary calcium in pregnancy.
**How sodium reduction helps:** For every 100 mmol drop in sodium, urinary calcium excretion drops by ~1 mmol.

**Avoid:**

- Restaurant/fast foods
- Broths, ramen, salted crackers
- Pickles, olives, deli meats
- Salted nuts, chips, processed snacks
- Cheese with very high sodium (e.g., halloumi, feta used heavily)

**2. Maintain—but do NOT exceed—normal pregnancy calcium intake (1,000–1,200 mg/day)**

Do **not** cut calcium. Restricting calcium **increases oxalate absorption**, worsening stone risk and impairing fetal skeletal development.

Aim to get calcium from **food sources**, not supplements unless medically indicated.

Best options (∼300 mg per serving):

- Milk or fortified plant milks
- Yogurt
- Cheese in moderate amounts
- Calcium-fortified orange juice

**3. Moderate animal protein**

High animal protein increases calcium excretion and lowers citrate (stone inhibitor).
Pregnancy-safe target: **0.8–1.0 g/kg/day** (~50–60 g/day for many women unless advised otherwise by obstetrics).

Choose:

- Eggs, beans, lentils, tofu
- Fish 2× weekly (avoid high-mercury fish)
- Lean poultry in moderate portions

Limit:

- Red meat
- Processed meats (also high sodium)

**3. Sample Menu Suggestions (Pregnancy-safe, stone-preventive)**

**Breakfast**

**Good choices:**

- Unsweetened yogurt with berries and oats
- Whole grain toast with avocado
- Fortified plant milk smoothie with spinach + banana
- Low-sodium cottage cheese + fruit

**Avoid:**

- Salted bagels, processed breakfast sandwiches
- High-sodium cereals or instant oatmeal packets

**Lunch**

**Good options:**

- Grilled chicken or tofu bowl with brown rice + vegetables (no added soy sauce—use lemon/herbs)
- Lentil soup made at home (no bouillon cubes)
- Low-sodium hummus with whole grain pita and salad

**Avoid:**

- Restaurant soups (extremely high sodium)
- Deli meats, pickles
- Fast-food sandwiches

**Dinner**

**Good options:**

- Baked salmon with lemon + roasted vegetables
- Stir-fry with tofu and vegetables using low-sodium tamari or none at all
- Pasta with tomato-vegetable sauce (no jarred sauces unless labeled low-sodium)

**Avoid:**

- Soy sauce, teriyaki, commercial sauces
- Pre-marinated meats
- Frozen dinners

**Snacks**

**Good options:**

- Fresh fruit
- Unsalted nuts
- Yogurt
- Carrot sticks with homemade low-sodium dip

**Avoid:**

- Salted nuts or chips
- Pretzels
- Processed snack bars high in sodium

**4. Safety Warnings (Pregnancy-specific)**

**Fluid intake**

In pregnancy, aim for **2–2.5 L/day**, not the >3 L sometimes recommended for stone formers.
Excessive fluid intake can worsen edema and dilute electrolytes.

**Avoid supplements unless prescribed**

- **No vitamin D megadoses** (can raise calcium).
- **No calcium supplements** unless your OB recommends them.
- **No herbal diuretics**—unsafe in pregnancy.

**Monitor blood pressure**

Your high sodium intake + pregnancy increases the risk of gestational hypertension. Reducing sodium improves both maternal cardiovascular safety and stone risk.

**Discuss with your obstetric provider**

Hypercalciuria in pregnancy is usually benign, but persistent stones or repeated pain episodes warrant obstetric and urologic co-management.

**Std + QA:**

Here is **general, educational information** about diet patterns typically recommended for people with **calcium oxalate stones**, with **important adjustments for pregnancy**. This is **not medical advice**, and because you are pregnant and symptomatic, any changes must be reviewed with your obstetric provider and/or a nephrologist/urologist.

**🌿 General Principles for Calcium Oxalate Stone Prevention *During Pregnancy***

Pregnancy changes hydration needs, electrolyte handling, and nutritional requirements, so kidney-stone guidance must be adapted carefully.

Below are evidence-based **patterns**, not prescriptions.

**1. Maintain normal dietary calcium — do NOT restrict it**

Your urine calcium is elevated (9.0 mmol/day), but the solution during pregnancy is *not* calcium restriction.

**Why:**

- Restricting calcium increases oxalate absorption from the gut → *more* calcium oxalate stone risk.
- Calcium is essential for fetal bone development and maternal bone preservation.

**Typical pattern (for education only):**

- Aim for **adequate calcium through food** (e.g., dairy, fortified plant milks).
- Calcium-rich foods **with meals** help bind oxalate in the intestine.

**Avoid:**

- High-dose non-prescribed calcium supplements unless your obstetrician recommends them.

**2. Meaningfully reduce dietary sodium**

Your urine sodium (150 mmol/day) suggests **high sodium intake**, which directly increases urinary calcium excretion.

**Why sodium reduction matters:**
Lowering sodium → lowers urinary calcium → reduces stone risk.

**Pregnancy-appropriate sodium-reduction strategies:**

- Prefer home-cooked meals; limit restaurant/packaged foods.
- Choose low-sodium versions of bread, soups, broths, tomato products.
- Avoid frequent cured meats, pickles, chips, and savory snacks.
- Do not aim for *very* low sodium (<1.5 g/day) in pregnancy unless supervised — the goal is **moderation**, not restriction severe enough to alter blood volume.

**3. Increase fluids safely**

Hydration is the cornerstone of stone prevention, but pregnancy adds nuance.

**General educational guidance:**

- Aim for frequent water intake throughout the day.
- Many clinicians target **urine output ≥ 2–2.5 L/day** for stone prevention, but in pregnancy, hydration goals must consider nausea, edema, and blood pressure.

**Work with your obstetric provider if you have:**

- Swelling
- Hypertension or preeclampsia risk
- Heart or kidney conditions
- Significant vomiting

**4. Moderate high-oxalate foods (do not eliminate them)**

Pregnancy requires a balanced diet; eliminating entire food categories is usually not recommended.

**Foods that are high in oxalate:**

- Spinach
- Beets and beet greens
- Almonds, cashews, nut butters
- Rhubarb
- Swiss chard
- Soy products (soy milk, tofu)
- Dark chocolate
- Wheat bran

**How to approach this in pregnancy:**

- Limiting *excessive* portions is reasonable.
- Combine higher-oxalate foods with **calcium-containing foods** in the same meal to reduce oxalate absorption.

**5. Moderate animal protein**

High animal-protein diets increase calcium excretion and lower citrate.

During pregnancy:

- You **should not restrict protein excessively**, but you *can* avoid very high-protein patterns (large meat portions, keto-style diets).
- Balanced intake from poultry, fish, eggs, legumes is typically appropriate.

**6. Increase citrate-rich foods**

Citrate is a natural inhibitor of calcium oxalate crystallization.

Pregnancy-safe sources:

- Lemons and limes (lemon water)
- Oranges
- Melons

Avoid high-dose potassium citrate supplements unless prescribed, as potassium handling changes during pregnancy.

**7. Avoid excessive vitamin C supplementation**

Vitamin C is metabolized to oxalate.

- Avoid high-dose supplements (>500 mg/day) unless recommended by your clinician.
- Standard prenatal vitamins are fine.

**Summary of Pregnancy-Appropriate Diet Patterns**

| **Goal** | **Pregnancy-safe approach** |
| --- | --- |
| ↓ Urine calcium | Reduce sodium; maintain normal dietary calcium |
| ↓ Oxalate absorption | Pair oxalate foods with calcium; avoid very high-oxalate foods |
| ↑ Urine volume | Steady water intake with OB oversight |
| ↑ Urine citrate | Emphasize fruits (especially citrus) |
| Avoid harmful supplements | Limit vitamin C and avoid unprescribed calcium/potassium supplements |

**When to seek individualized guidance**

You should get real-time guidance from:

- **Obstetrics/MFM**, because hydration and electrolyte needs change in pregnancy.
- **Nephrology or a stone specialist**, because your urine studies show patterns (high calcium, high sodium) that can be modulated safely with tailored changes.

**SB + QB:**

Below is an evidence-based, pregnancy-safe, guideline-aligned interpretation tailored to your values and metabolic profile.
*(I will keep recommendations within what is considered safe in pregnancy per EAU/CUA guidance and obstetric nutrition principles.)*

**1. Metabolic Diagnosis**

Your 24-hour urine results show:

**• Hypercalciuria (Urine calcium 9.0 mmol/day)**

Normal is typically **<6.2 mmol/day** for women.
In pregnancy, urine calcium can rise *slightly*, but **9.0 is high enough to contribute to calcium oxalate stone formation.**

**• High Sodium Intake (Urine sodium 150 mmol/day)**

This is above the recommended 24-hour sodium target of **<100 mmol/day**.
High sodium **directly increases urinary calcium**, so the main driver of your hypercalciuria is *almost certainly excess salt intake*, NOT dietary calcium.

**Pregnancy Consideration**

You *should not* reduce dietary calcium. Pregnancy increases calcium needs, and restricting calcium increases oxalate absorption → **more stones + risk to fetal bone development**.

**2. Key Dietary Modifications (Pregnancy-Safe Priorities)**

**1. Reduce dietary sodium to <2 g/day (≈5 g salt/day)**

This is the **most important** step for lowering urinary calcium safely during pregnancy.
Focus on: avoiding packaged/processed foods, limiting restaurant meals, flavoring with herbs instead of salt.

**2. Maintain adequate calcium intake: 1,000–1,200 mg/day**

This prevents oxalate absorption and supports fetal bone development.
Aim for **dietary calcium with meals**, not supplements (unless OB specifically prescribes).

**3. Moderate animal protein intake**

High animal protein increases calcium excretion and lowers citrate.
Goal: **1–2 servings/day**, prioritizing lean poultry, eggs, legumes, tofu, pregnancy-safe fish.

**3. Sample Menu Suggestions**

**Foods to Emphasize**

**Breakfast**

- Greek yogurt (½–1 cup) with fresh berries
- Low-sodium whole-grain toast with avocado
- Oatmeal with chia seeds + a splash of milk

**Lunch**

- Quinoa salad with chickpeas, cucumber, olive oil, lemon
- Low-sodium cottage cheese side
- Grilled chicken or tofu (85–100 g)

**Snacks**

- Fresh fruit: banana, pear, apple
- Raw almonds (small handful)
- Carrot sticks with hummus (check low-sodium variety)

**Dinner**

- Baked salmon (good omega-3s for pregnancy; avoid high-mercury fish)
- Steamed veggies (broccoli, zucchini, carrots)
- Brown rice / whole-grain pasta
- Add a calcium source (sprinkled cheese, yogurt-based dressing)

**Foods to Limit or Avoid**

**High-sodium foods (your #1 risk driver)**

- Processed meats (ham, sausages, deli meats)
- Fast food, restaurant soups, ramen
- Packaged snacks (chips, crackers)
- Store-bought sauces (soy sauce, teriyaki, bouillon cubes)

**High-oxalate foods (okay in moderation if eaten *with* calcium)**

- Spinach, beets, Swiss chard
- Almond butter (small amounts are okay)
- Rhubarb
- Dark chocolate (limit to occasional)

**Avoid calcium supplements unless OB approves**

Calcium from **food** is safer and decreases oxalate absorption.

**4. Safety Warning (Pregnancy-Specific)**

**✔ Fluid intake:**

General stone prevention recommends **2.5–3 L/day**, but in pregnancy:

- Drink to maintain **pale-yellow urine**, not excessive volumes.
- Avoid >3.5 L/day to prevent electrolyte issues.

**✔ Potassium-rich foods:**

Normally helpful (increase citrate), but **avoid deliberate potassium loading** (e.g., supplements) in pregnancy. Foods like bananas, oranges, melons are safe in normal portions.

**✔ Protein:**

Do not restrict below pregnancy requirements (~70–75 g/day). Focus on plant-based sources when possible.

**✔ Always coordinate with OB or midwife, especially if you develop swelling, high blood pressure, or need sodium adjustments for medical reasons (e.g., pre-eclampsia monitoring).**

**Std + QB:**

Here’s **safe, pregnancy-appropriate, evidence-based *general information*** about diet and calcium-oxalate stone prevention.
I am **not** giving you a diagnosis or personal medical plan — only education you can use to discuss your care with your obstetrician and urologist.

**⭐ Key Goals in Pregnancy With Calcium-Oxalate Stones**

For calcium-oxalate stone formers, two modifiable factors stand out in your numbers:

- **Urine calcium: 9.0 mmol/day** → on the higher side
- **Urine sodium: 150 mmol/day** → higher sodium increases urinary calcium, so reducing dietary sodium is *usually* the safest first step.

Because you are **pregnant**, you **should NOT restrict calcium intake** — the baby needs calcium, and low calcium intake can *increase* oxalate absorption (raising stone risk).

**❤️ What You *Generally* Can Eat Safely in Pregnancy to Reduce Stone Risk**

**1. Keep normal dietary calcium (not low!)**

- Aim for **1,000–1,200 mg/day** from food — this is standard for pregnancy.
- Best sources: dairy (milk, yogurt, cheese), calcium-fortified non-dairy milks, leafy greens that are low in oxalate (kale, bok choy).

**Why?** Calcium eaten **with meals** binds oxalate in the gut → lowers oxalate absorption → prevents stones.

⚠️ Avoid calcium restriction unless a specialist specifically instructs you otherwise.

**2. Reduce dietary sodium**

High sodium increases urinary calcium, which worsens stone risk.

**Pregnancy-safe general target:**

- **≤ 2,000 mg sodium/day** (your OB may adjust)

How to reduce sodium:

- Limit processed foods, fast food, deli meats, canned soups.
- Choose “no salt added” versions when possible.
- Cook at home with herbs/spices instead of salt.
- Avoid sports drinks unless medically indicated.

Even a modest reduction in sodium can lower your urinary calcium.

**3. Moderate animal protein**

Too much animal protein can increase calcium excretion and lower citrate (a natural stone inhibitor).

**Generally safe guidance in pregnancy:**

- Choose moderate portions (e.g., palm-sized) of chicken, fish, eggs, lean meat.
- Include plant proteins such as lentils, beans, tofu.
- Avoid high-protein fad diets.

**4. Limit very high-oxalate foods**

No need for a strict oxalate-free diet, but moderating the highest sources helps.

Some **very high-oxalate foods** to limit:

- Spinach (especially raw)
- Almonds and nut butters
- Beets
- Rhubarb
- Swiss chard
- Wheat bran
- Dark chocolate in large amounts

You do **not** need to avoid all oxalate foods.
**Tip:** Pair oxalate foods with a calcium-containing food at the same meal.

**5. Increase natural citrate**

Citrate protects against stones.

Pregnancy-safe citrate sources:

- Lemon or lime juice in water
- Fruits and vegetables in general

(Do not start potassium citrate medication unless prescribed.)

**6. Hydration: the most important habit**

Urine should be pale yellow.
In pregnancy, fluid needs are higher.

General safe program:

- About **2.3–2.5 liters/day total fluids** unless your obstetrician gives a modified amount.
- Spread water intake throughout the day.

**Foods That Often Work Well for Pregnant Stone Formers**

These *examples* are safe for most people, but confirm with your clinician:

**Breakfast**

- Yogurt + berries + oatmeal
- Eggs + whole-grain toast
- Calcium-fortified plant milk smoothie

**Lunch**

- Chicken or tofu salad with leafy greens **except spinach**
- Sandwich with low-sodium turkey, veggies, small amount of cheese

**Snacks**

- Fresh fruit
- Low-sodium cheese
- Rice cakes, plain popcorn
- Yogurt

**Dinner**

- Grilled fish or chicken + vegetables + rice or potatoes
- Lentil or bean-based meals (moderate sodium)

**Things to Avoid or Use With Caution**

- **Calcium supplements** → Only take if prescribed, because they may raise stone risk when taken without food.
- **Vitamin C supplements (>200 mg/day)** → Can increase oxalate (avoid unless OB prescribes).
- **Sodium-heavy prenatal snacks** (chips, instant noodles, sports drinks).
- **Very high-oxalate greens and nuts** as daily staples.

**When to Seek Clinician Input**

Because you are pregnant, discuss the following with your obstetrician:

- Whether sodium reduction targets should be tailored for you
- Safe hydration goals (especially if you have nausea or swelling)
- Whether your calcium intake is adequate from food alone
- Whether you need repeat urine testing during pregnancy

**Case24:**

**SA + QA:**

Below is a **stone-prevention plan tailored to your age, calcium oxalate stone history, and urinary incontinence**, following EAU & CUA metabolic-management principles.

**1. Metabolic Diagnosis**

Your **primary abnormality is very low urine volume (0.8 L/day)**.
This places you at high risk for calcium oxalate stone recurrence because urine becomes too concentrated.
However, **incontinence makes aggressive fluid loading unsafe and unrealistic**, so hydration must be *strategic* rather than simply “drink more.”

**2. Key Dietary & Hydration Modifications**

Here are the **top 3 priorities** that raise urine volume **without worsening incontinence**:

**① Timed, Distributed Hydration (Avoid Bolus Drinking)**

- Drink **small amounts (100–150 mL)** at a time every **60–90 minutes** while awake.
- Avoid large glasses of water, which worsen urgency and leak episodes.

**② Front-Load Fluids in Early Day**

- Consume ~70% of fluids **before 4 PM**.
- After 6 PM, reduce intake to **sips only** to avoid nocturia and nighttime leakage.

**③ Choose “Hydrating but Bladder-Friendly" Fluids**

Avoid bladder irritants (acidic, caffeinated, carbonated), and instead use:

- Water (still, not sparkling)
- Diluted herbal teas (chamomile, ginger)
- Milk or lactose-free milk (also provides protective dietary calcium)
- Oral hydration solutions during hot weather or exercise (but choose **low-sodium** versions)

**3. Sample Menu & Hydration Suggestions**

**Morning (Best time for most fluids)**

✔ 150 mL water on waking
✔ Herbal tea + breakfast
✔ 150 mL water mid-morning
✔ Milk with lunch (helps bind dietary oxalate in the gut)

**Afternoon**

✔ 120–150 mL water every 60–90 min
✔ Fruit with high water content but gentle on bladder:

- Melon
- Pear
- Blueberries

**Evening (Fluid restriction zone)**

✔ Small sips only
✔ Avoid: citrus juice, carbonated water, caffeine, tomato juice—these worsen urgency and frequency.

**Hydrating Foods That Don’t Irritate the Bladder**

- Cucumbers
- Lettuce
- Zucchini
- Oatmeal prepared with milk
- Soups with low sodium (earlier in the day)

These help raise urine output *without large volumes of liquid*.

**Foods & Drinks to Avoid Late-Day**

- Tea/coffee
- Citrus drinks (orange, lemonade)
- Sparkling water
- Spicy soups
  These increase urgency and bladder contractions.

**4. Safety Warning**

Because you are 80 years old and have urinary incontinence:

- **Do not rapidly increase fluids**; distribute fluids throughout the day.
- If you also have **heart failure or CKD**, fluid increases must be medically supervised. Please let me know if these conditions apply.
- Pelvic floor muscle training or referral to pelvic physiotherapy can significantly improve continence, making fluid goals easier to achieve.

**Std + QA:**

It’s a **very common and very real challenge**: you *need* more fluid to prevent calcium oxalate stone recurrence, but more fluid often *worsens* urinary incontinence.
Here are **safe, practical, non-medical educational strategies** that patients commonly use to raise urine output while limiting the impact on bladder control. Use these ideas to discuss a tailored plan with your clinician.

**⭐ Key Principle**

To prevent stones, most guidelines recommend **1.8–2.5 L of urine per day**. With a current urine volume of **0.8 L/day**, you likely need to increase fluid intake — but **it doesn’t have to mean drinking large volumes at once**, nor drinking before activities that worsen leakage.

**🔹 Strategies to Increase Fluids *Without* Worsening Incontinence**

**1. Distribute fluids evenly throughout the day**

Instead of drinking a full glass at once, try **small amounts (2–3 oz)** every 20–30 minutes.
Why it helps:

- Keeps urine production steady
- Avoids sudden bladder filling

**2. Shift most fluids to earlier in the day**

If nighttime leakage or urgency is a problem:

- Drink more in the **morning and early afternoon**
- Reduce intake **2–3 hours before bedtime**

This still increases 24-hour volume but protects nighttime continence.

**3. Choose fluids that hydrate efficiently**

Plain water works best.
Avoid (because they increase urine output sharply or irritate the bladder):

- Caffeine (coffee, tea, soda)
- Alcohol
- Carbonated drinks
- Artificial sweeteners if they bother your bladder

**4. Pair fluids with activities that already prompt bathroom access**

For example:

- Drink a small glass **before meals**
- Sip during times you’re already near a bathroom
- Take your largest fluid amount at a time when leakage bothers you the least

**5. Use bladder-friendly tools**

These don’t treat incontinence medically but help manage it while fluid increases:

- **Scheduled voiding** every 1.5–2 hours to stay ahead of urgency
- **Pelvic floor exercises** (Kegels), which can reduce leakage over time
- **Absorbent products** temporarily during the adjustment period

**6. Add fluids through high-water foods**

These can raise urine volume without feeling like additional drinking:

- Water-rich fruits (watermelon, berries, oranges)
- Soups or broths (low-salt)
- Cucumbers, tomatoes, lettuce

These contribute meaningfully to hydration but often cause less urgency.

**7. Avoid “rapid bladder fillers”**

Some drinks empty into the bladder very quickly, worsening urgency:

- Large cold beverages
- Chugging fluids
- Caffeinated drinks
  Spacing and warming fluids can reduce this effect.

**🔹 A Common Practical Goal**

Many patients find improvement by:

- Increasing to **~1.5 L intake/day at first**, not all the way to goal
- Monitoring leakage
- Gradually raising fluid by **4–6 ounces every few days**
  This slow titration often avoids worsening incontinence.

**🔹 When to talk to your clinician**

Because you have incontinence *and* need higher fluid intake, ask about:

- Evaluation for **urge vs. stress incontinence** (management differs)
- **Bladder training programs**
- Whether any medications you take affect the bladder
- If pelvic floor therapy would help

**✔️ Summary**

You *can* raise urine volume for stone prevention **without significantly worsening leakage** by:

1. Spreading fluids throughout the day
2. Front-loading intake earlier
3. Avoiding bladder irritants
4. Using small, steady sips
5. Adding water-rich foods
6. Incorporating bladder management techniques

**SB + QB:**

Here is the most practical, guideline-consistent approach for **hydrating safely in an older adult with urinary incontinence**, while still targeting a **≥2.0–2.5 L/day urine output** for stone prevention (EAU & CUA recommendations).
I will apply the required structure, even though this question focuses only on hydration.

**1. Metabolic Diagnosis**

The patient has **low urine volume (<1 L/day)**, a major independent risk factor for all stone types. The limiting factor is **urinary incontinence**, which makes large, infrequent fluid boluses impractical and increases fall/urgency risk.

**2. Key Dietary / Behavioral Modifications (Top 3 Practical Hydration Strategies)**

**1. Use “micro-drinking” instead of large fluid boluses**

- Sip **100–150 mL every hour** while awake.
- This raises urine volume gradually **without overwhelming bladder capacity**.

**2. Front-load hydration earlier in the day**

- Aim for **60–70% of daily fluid intake before 3 PM** to reduce nighttime voiding and incontinence.
- Avoid catching up at night.

**3. Use low-irritant fluids to protect bladder stability**

- Prefer **water, herbal teas, diluted fruit water**.
- Limit bladder irritants: **caffeine, citrus juices, carbonated beverages, artificial sweeteners**, which can worsen urgency and leakage.

**3. Sample Menu / Fluid Suggestions**

**Fluids to Prefer (spread evenly)**

- Plain water
- Warm water with a splash of lemon *only if citrate needs to be added*; otherwise avoid strong citrus if bladder irritation occurs
- Rooibos tea or herbal tea (non-caffeinated)
- Water-rich foods: cucumbers, melon, strawberries, zucchini, soups with low sodium
- Electrolyte tablets with **no added sodium** (if taste encourages drinking)

**Fluids to Limit or Avoid**

- Coffee or caffeinated tea (>1 cup/day)
- Citrus juices (orange, grapefruit) – can irritate bladder
- Soda/carbonated beverages
- Sweetened beverages
- High-sodium broths (increase calciuria)

**Example Daily Hydration Schedule (Safe for Incontinence)**

| **Time** | **Intake** | **Rationale** |
| --- | --- | --- |
| **7:30 AM** | 150 mL water | Gentle start; prevents morning dehydration |
| **8:30 AM** | 120 mL water | Micro-hydration |
| **9:30 AM** | Herbal tea 150 mL | Warm fluids ease bladder urgency |
| **11:00 AM** | 200 mL water | Biggest drink while daytime mobility is best |
| **12:30 PM** | 150 mL water + soup | Hydrating meal |
| **2:00 PM** | 150 mL water | Finish most fluids by now |
| **4:00 PM** | 120 mL water | Small top-off |
| **After 6 PM** | *Very small sips only (≤100 mL/hr)* | Reduces nocturia |

This schedule typically reaches **1.7–1.9 L intake**, enough to move urine volume closer to target with minimal bladder burden.

**4. Safety Warning**

- If the patient has **heart failure, CKD stage ≥3, or is on diuretics**, fluid goals **must be individualized**—over-hydration can worsen edema or electrolyte balance.
- If urgency or incontinence worsens despite micro-hydration, refer to pelvic floor physiotherapy or continence clinic.
- Sudden increase in nocturia or worsening leakage should prompt evaluation for UTI or pelvic organ prolapse.

**Std + QB:**

Here’s **safe, practical, older-adult–friendly hydration guidance** for preventing kidney stones **without worsening urinary incontinence**.
*(General education only—not personal medical advice.)*

**🌤️ Core Goal**

**Increase total daily fluid intake enough to reach ~1.5–2.0 liters of urine per day**, but **spread out** to avoid bladder stress.

For many adults with incontinence, the *pattern* of drinking matters just as much as the total amount.

**✅ Practical Strategies That Work Well for Older Adults**

**1. Small, steady sips—avoid large boluses**

Instead of drinking big glasses of water at once (which rapidly fills the bladder):

- Sip **½ cup (100–150 mL)** of fluid every hour or two while awake.
- This keeps urine dilute **without sudden surges** that trigger leakage.

**Why this helps:** Kidney stone prevention requires overall urine volume, not fast ingestion.

**2. Front-load fluids earlier in the day**

Most people with incontinence do better when evening intake is reduced.

- Drink **most fluids before 6 PM**.
- After dinner, limit to small sips unless instructed otherwise by your clinician.

This still supports good 24-hour urine volume while decreasing nighttime accidents.

**3. Pair drinking with routine activities**

Link hydration to things you already do:

- Take a few sips each time you take medications.
- Sip after bathroom trips.
- Keep a water bottle where you sit (TV chair, bedside table) and drink small amounts regularly.

Consistent cues make slow-and-steady drinking easier.

**4. Use bladder-friendly fluids**

To avoid worsening urgency/incontinence:

**Better choices:**

- Water (plain or flavored with lemon/lime)
- Herbal teas
- Diluted fruit water
- Broth

**Limit:**

- Coffee and caffeinated tea (increase urgency)
- Soda
- Citrus juices in large amounts (can irritate bladder in some)

**5. Hydrate with foods too**

If drinking bothers your bladder, include water-rich foods:

- Melons, berries
- Cucumbers, lettuce
- Soups (earlier in the day)

These contribute to urine volume **without suddenly filling the bladder**.

**6. Track urine color**

A simple daily guide:

- **Pale yellow** = good hydration for stone prevention
- **Dark yellow** = you likely need small additional sips earlier in the day

This avoids over-drinking while keeping your urine dilute.

**7. Discuss continence tools if needed**

Many older adults safely achieve higher hydration when using:

- Scheduled voiding (every 2–3 hrs)
- Pelvic floor therapy
- Absorbent pads designed for comfort
- Bladder training techniques

These can allow you to meet stone-prevention goals without fear of leakage.

**🧪 What Your Target Looks Like**

To reduce stone recurrence, guidelines aim for:

- **Urine output ≥ 2 liters/day**
  But if you have incontinence, even increasing from **0.8 → 1.2–1.5 liters/day** is a meaningful step. You don’t need to hit the full target immediately—**gradual increases are safe and effective**.

**🧘‍♀️ The Bottom Line**

**“Slow, steady, earlier-in-the-day hydration”** is the safest approach for someone with kidney stone risk *and* urinary incontinence.
You don’t need big volumes at once—just consistent small amounts.

**Case25:**

**SA + QA:**

Below is your personalized, guideline-based stone-prevention plan tailored to a **post–gastric bypass calcium oxalate stone former** with **urine calcium 9.5 mmol/day** (≈ *mild–moderate hypercalciuria*). Bariatric surgery introduces **unique metabolic risks**—mainly **enteric hyperoxaluria**, chronic **low urine volume**, and **fat-malabsorption–induced calcium binding**—so the strategy differs from standard stone prevention.

**1. Metabolic Diagnosis**

- **Enteric hyperoxaluria secondary to gastric bypass**, causing increased intestinal oxalate absorption.
- **Mild–moderate hypercalciuria (9.5 mmol/day)** likely **sodium- and protein-sensitive**, but also worsened by high oxalate absorption.
- **Low effective calcium availability in the gut** (post-bypass) → more free oxalate absorption → higher risk of calcium oxalate stones.

**2. Key Dietary Modifications (Top 3 Priorities)**

**1) Take calcium *with meals* (not restrict it) — mandatory after bariatric surgery**

- **1,200–1,500 mg/day calcium citrate** divided with meals.
- Calcium taken *with* oxalate-containing foods binds oxalate in the gut and prevents absorption.
- Calcium citrate is preferred due to better absorption after bypass and citrate’s inhibitory effect on stone formation.

**2) Reduce dietary oxalate + dietary fat to minimize enteric hyperoxaluria**

- Limit **high-oxalate foods** (spinach, almonds, rhubarb, beets, peanut butter, Swiss chard, dark chocolate).
- Keep **dietary fat moderate** (too much fat → unabsorbed fatty acids bind calcium → free oxalate rises).

**3) Control sodium and animal protein to reduce urinary calcium**

- Sodium goal: **<2 g/day (≈5 g salt)**.
- Protein goal: **0.8–1.0 g/kg/day** from lean sources.
- Both sodium and protein load directly increase urine calcium excretion.

**3. Sample Menu Suggestions**

**Best Foods for You**

**Breakfast**

- Scrambled eggs + low-fat Greek yogurt
- Oatmeal topped with blueberries
- *Calcium citrate supplement taken with breakfast*

**Lunch**

- Grilled chicken breast or tofu
- Quinoa or brown rice
- Steamed broccoli or green beans
- *Calcium citrate taken with this meal*

**Dinner**

- Baked salmon
- Roasted sweet potato
- Zucchini or cauliflower
- *Calcium citrate with dinner*

**Low-Oxalate Snacks**

- Cheese sticks
- Hard-boiled eggs
- Low-oxalate fruits: bananas, mango, melon
- Low-fat cottage cheese

**Foods to Avoid or Limit**

- **Very high oxalate:**
  Spinach, Swiss chard, almonds, peanuts, cashews, beets, rhubarb, dark chocolate, wheat bran.
- **High-fat meals:**
  Fried foods, creamy sauces, high-fat desserts.
- **High sodium:**
  Fast food, deli meats, packaged soups, pickles, chips.
- **Excess animal protein:**
  Large servings of red meat, protein shakes, keto-style diets.

**Special Bariatric-Specific Hydration Advice**

- Aim for **2.5–3 L fluid/day** *total* but consumed in **small, frequent sips** due to reduced gastric volume.
- Include **citrate-rich beverages** (lemon water, Crystal Light lemonade).
- Avoid drinking 30 minutes before or after meals if recommended by your bariatric team.

**4. Safety Warning**

- After gastric bypass, **malabsorption** can increase risk of deficiencies (calcium, vitamin D, iron, B12).
- Always coordinate calcium supplementation with your bariatric surgeon/dietitian.
- If you have **fat malabsorption, diarrhea, or steatorrhea**, lowering dietary fat becomes even more essential.
- If you have **CKD**, potassium-rich citrate sources should be moderated—tell me if this applies.

**Std + QA:**

Here is evidence-based, **safe, non-diagnostic dietary guidance** appropriate for someone with **recurrent calcium-oxalate kidney stones after gastric bypass (RYGB)** and **high urine calcium (hypercalciuria)**. Bariatric surgery changes how you absorb nutrients, so prevention strategies differ from those used in people without surgery.

**✅ Key Diet Priorities After Gastric Bypass to Prevent Stones**

**1. Ensure *adequate calcium intake from food with meals***

After gastric bypass, the small intestine absorbs *less* calcium. When calcium intake is too low, more oxalate gets absorbed → **higher urine oxalate → more stones**.

**What to do:**

- Aim for **1,200–1,500 mg/day of calcium**, ideally as **calcium citrate** (better absorbed after RYGB than calcium carbonate).
- Take **300–500 mg with each meal**—this binds oxalate *in the gut* and prevents absorption.
- Food sources: dairy, fortified plant milks, yogurt, cheese.
- If using supplements, choose **calcium citrate** and take with meals.

**2. Increase fluid intake**

After bariatric surgery, the stomach holds less volume, so drinking large amounts at once is difficult.

**What to do:**

- Aim for a **urine output of >2–2.5 liters/day**, which may mean drinking 2.5–3 liters of fluid daily.
- Sip fluids throughout the day.
- Avoid drinking in the 30 minutes before and after meals (standard post-bypass instructions), so hydration must occur between meals.

Good choices: water, lemon water, herbal tea, infused water.

****3. Follow a *low-oxalate diet* (because RYGB strongly increases oxalate absorption)**

Goal: reduce gut oxalate load while ensuring calcium binds ingested oxalate.

**High-oxalate foods to limit:**

- Spinach (extremely high)
- Almonds and other nuts
- Sesame/tahini
- Beets, rhubarb
- Chocolate and cocoa
- Wheat bran
- Sweet potatoes
- Black tea (moderation)

**Low-oxalate foods you can choose instead:**

- Kale, romaine, iceberg lettuce
- Broccoli, cauliflower
- Cucumbers, zucchini, mushrooms
- Berries, apples, grapes
- White rice, quinoa

**4. Moderate sodium intake (important because urine calcium is high)**

Sodium increases urinary calcium excretion → worsens hypercalciuria.

**What to do:**

- Keep sodium intake **<2 grams/day** (≈5 g of salt).
- Avoid fast food, processed meats, canned soups, salty snacks.

**5. Eat *normal* (not low) dietary calcium**

A common mistake is avoiding calcium, which increases oxalate absorption and stone risk.

You should *not* follow a low-calcium diet even if urine calcium is high—your elevated urine calcium may be driven by:

- malabsorption
- increased bone turnover after RYGB
- high sodium intake

Correct calcium intake is still protective.

**6. Limit animal protein to moderate levels**

High protein → increases urine calcium and uric acid → promotes stones.

**What to do:**

- Aim for **0.8–1.0 g protein per kg body weight/day**, unless your bariatric team has different goals.
- Emphasize plant-based proteins when possible (lower acid load).

**7. Include citrate-rich foods**

Citrate helps prevent stone formation and is often low after gastric bypass.

**Useful options:**

- Lemon or lime water
- Citrus fruits
- Low-sugar lemonade (e.g., homemade with lemon + water + small sweetener)

Consider asking your doctor whether potassium citrate supplementation is appropriate if urine citrate is low (common after bariatric surgery).

**8. Avoid high-dose vitamin C**

Vitamin C is converted to oxalate.

- Avoid supplements >200 mg/day unless prescribed.

**9. If you have fat malabsorption after bypass**

Unabsorbed fat binds calcium → less calcium left to bind oxalate → **oxalate absorption increases**.

If you have fatty stools or known malabsorption:

- Limit high-fat foods
- Use medium-chain triglycerides (MCT oil) if extra energy is needed (absorbed without fat–calcium binding)
- Discuss pancreatic enzyme support with your clinician if malabsorption is significant

**👍 Summary of Your Highest-Yield Steps**

1. **Fluids:** Sip to achieve >2–2.5 L urine/day.
2. **Calcium citrate with meals:** 300–500 mg with each meal (total 1,200–1,500 mg/day).
3. **Low-oxalate diet:** Avoid spinach, nuts, chocolate, etc.
4. **Low sodium:** <2 g/day.
5. **Moderate animal protein.**
6. **Add citrate (lemon water).**
7. **Avoid high-dose vitamin C.**
8. **Watch dietary fat if malabsorption is present.**

**SB + QB:**

Below is a **clinical-grade interpretation** tailored to **post–gastric bypass physiology**, where *enteric hyperoxaluria* is the dominant mechanism—not dietary calcium excess.

**1. Metabolic Diagnosis**

**The patient has *secondary enteric hyperoxaluria* after gastric bypass with *normal–low urine calcium* (9.5 mmol/day ≈ 380 mg/day).**
This means:

- You are *not* losing too much calcium in the urine.
- Your bypass surgery causes fat malabsorption → unbound fatty acids bind calcium in the gut → **oxalate remains unbound and over-absorbed → calcium oxalate stones**.
- Cutting dietary calcium will **worsen stone formation**, because even *less* calcium will be available to bind oxalate in the gut.

**Evidence:** EAU & CUA guidelines stress *increasing dietary calcium with meals* in enteric hyperoxaluria, not restricting it.

**2. Key Dietary Modifications (Top 3 Priorities)**

**1. Increase Calcium *with Meals* (NOT restrict it)**

- Goal: **1,200–1,500 mg/day total calcium**, ideally from food.
- Take calcium **citrate**, 300–500 mg **with each meal**, if you cannot meet dietary intake.
- Calcium binds oxalate in the gut → prevents absorption.

**2. Reduce High-Oxalate Foods (especially those absorbed aggressively post-bypass)**

Avoid or minimize:

- Spinach, almonds, almond flour, beets
- Rhubarb, Swiss chard
- Dark chocolate/cocoa
- Sweet potatoes
- Peanuts and nut butters
- Large quantities of tea

**3. Control Fat Intake**

After gastric bypass, high fat causes more calcium–fat binding → **more free oxalate gets absorbed**.

- Keep fat to **<30% of daily calories**.
- Avoid greasy, fried, or oil-heavy meals.

**3. Sample Menu Suggestions**

**Foods to Eat (help bind oxalate & reduce risk)**

- **Breakfast:**
  Greek yogurt + berries; whole-grain toast with calcium-fortified almond or cow’s milk.
  *(Add calcium citrate if intake is low.)*
- **Lunch:**
  Grilled chicken or fish + white rice + low-oxalate vegetables (broccoli, cauliflower, zucchini).
  Small serving of cheese or fortified soy milk **with the meal**.
- **Dinner:**
  Turkey chili with beans; steamed green beans; side of low-fat cottage cheese.
  OR
  Baked salmon + quinoa + roasted carrots.
- **Snacks:**
  Low-fat dairy, yogurt, cheese sticks, calcium-fortified plant milks, apples, pears.

**Foods to Avoid or Greatly Reduce**

- Spinach salad
- Almond flour baked goods (very high oxalate)
- Sweet potatoes
- Beet smoothies
- Dark chocolate bars
- Nuts in large quantities

**4. Safety Warning**

Because you are post–gastric bypass:

- **Avoid high-dose vitamin C** (>500 mg/day) → it converts to oxalate.
- **Calcium citrate** is preferred because bypass patients absorb citrate better and it reduces stone risk.
- If you have **fat malabsorption symptoms** (oil droplets in stool, chronic diarrhea), you may need additional therapies (e.g., bile acid binders) under medical supervision.

**Key Takeaway**

**Do NOT cut calcium.**
**Increase calcium *with meals*, moderate dietary oxalate, and reduce fat intake.**
This is the cornerstone of preventing calcium oxalate stones in post-bariatric patients.

**Std + QB:**

I can give **general, educational information** about kidney-stone risk after gastric bypass, but **I cannot give personal medical advice** or tell you what *you* should do. This type of decision—especially with a surgery-altered GI tract and abnormal urine chemistry—must be made with your bariatric and kidney-stone specialists.

That said, here is what is *typically* considered in patients who develop **calcium oxalate stones after Roux-en-Y gastric bypass (RYGB)**.

**⭐ Why kidney stones occur after gastric bypass**

After RYGB, people often develop:

- **Enteric hyperoxaluria** – more oxalate is absorbed from food because unabsorbed fat binds calcium in the gut.
- **Low urine citrate** – citrate protects against stones.
- **Very low urine volume** – due to limited stomach size.

These factors often matter **more than urine calcium**, even if your calcium output is normal or mildly high.

Your urine calcium (9.5 mmol/day ≈ ~380 mg/day) is on the higher side of normal, but not extremely high. Cutting dietary calcium is usually **not** recommended after bypass, because it can *increase* oxalate absorption.

**⭐ Should you cut calcium?**

**Generally, no.**
After gastric bypass, **reducing dietary calcium increases the risk of calcium-oxalate stones** because less calcium is available in the intestine to bind oxalate.

Low intestinal calcium → more free oxalate → more oxalate absorbed → more stones.

**⭐ Should you take calcium supplements?**

Often, **yes — with meals**, because:

- Calcium carbonate or calcium citrate taken **with food** binds oxalate and reduces absorption.
- Many bariatric guidelines prefer **calcium citrate** because it is absorbed better with reduced stomach acid and may raise urinary citrate slightly.

Typical general guidance (NOT personal medical advice):

- Calcium citrate with meals (dose individualized by clinician).
- Spread through the day rather than taken all at once.

⚠️ Your own clinician must determine the dose and whether it fits your lab results and bone-health needs.

**⭐ Foods you may be advised to avoid or limit**

Patients after RYGB who form calcium-oxalate stones are often told to **reduce high-oxalate foods**, especially when eaten without calcium.

Common high-oxalate foods:

- Spinach (extremely high)
- Almonds, peanuts, cashews
- Beets
- Rhubarb
- Swiss chard
- Dark chocolate / cocoa
- Sweet potatoes
- Wheat bran
- Soy products
- Black tea (in large amounts)

Instead of avoiding all oxalate, the better strategy is **pairing oxalate-containing foods with calcium**.

**⭐ Other important dietary considerations**

**✔ Increase fluid intake**

The most effective general strategy is aiming for **>2–2.5 L urine/day**, but this must respect gastric-bypass limitations, so clinicians usually recommend:

- Frequent small sips throughout the day
- Avoiding large-volume drinking at once

**✔ Limit dietary fat if you have steatorrhea**

Unabsorbed fat binds calcium and increases oxalate absorption.

**✔ Reduce sodium intake**

High sodium increases urinary calcium.
General diet recommendations often aim for **<2 g sodium/day**.

**✔ Moderate animal protein**

High animal protein increases urinary calcium and lowers citrate.

**✔ Citrate sources**

Lemon or lime juice (natural citrate) may help raise urine citrate.

**⭐ Putting it together (generally)**

Most RYGB stone-formers are advised to:

1. **Do NOT reduce calcium** intake.
2. **Take calcium citrate with meals**, if recommended by their clinician.
3. **Limit very high-oxalate foods**, especially if not eaten with calcium.
4. **Increase fluids** as tolerated.
5. **Reduce sodium** and **moderate animal protein**.
6. Address **fat malabsorption**, if present.

**⚠️ Very important**

Because you have:

- A history of gastric bypass
- Recurrent calcium-oxalate stones
- Specific urine chemistry abnormalities

…your management **requires individualized medical guidance**. A nephrologist and bariatric dietitian can tailor:

- The correct calcium supplement type and dose
- Whether you need citrate supplementation
- Oxalate limits appropriate for your diet
- How your urine calcium should be interpreted
- Whether fat malabsorption is contributing

**Case26:**

**SA + QA:**

Below is an evidence-based, guideline-aligned interpretation tailored to your situation as a recurrent **calcium oxalate stone former** with **persistently high urine calcium despite already low urine sodium (100 mmol/day).**

Because no comorbidities were provided, I will give standard recommendations; feel free to share additional medical history for further refinement.

**1. Metabolic Diagnosis**

**Persistent idiopathic hypercalciuria not driven by sodium intake — likely influenced by excessive animal protein load, low dietary alkali (fruits/vegetables), or high refined carbohydrate intake.**

- Your urine sodium is already within the target (<100–120 mmol/d), so sodium is *not* the driver.
- High animal protein → increases acid load → suppresses citrate + increases urinary calcium.
- Low dietary calcium paradoxically increases oxalate absorption → more CaOx stone formation.
- High sugar/carbohydrate spikes (especially sucrose and fructose) increase urinary calcium.

**2. Key Dietary Modifications (Top Priorities)**

**1. Moderate Animal Protein to 0.8–1.0 g/kg/day (≈60–80 g/day for a 50-yr-old man)**

- Excess protein → ↑ urinary calcium + ↓ citrate.
- Replace some animal protein with plant-based protein sources.

**2. Increase Dietary Alkali (Fruits + Vegetables) to Raise Citrate and Reduce Ca Excretion**

- EAU + CUA guidelines emphasize **potassium-rich, alkali-producing foods** to counteract acid load.
- Target: **≥6–8 servings per day** of low-oxalate fruits/vegetables.

**3. Maintain Normal Dietary Calcium (1000–1200 mg/day) But Pair Calcium with Meals**

- Adequate calcium binds oxalate in the gut → lowers urinary oxalate.
- Calcium at meals is more important than total daily calcium.

**3. Sample Menu Suggestions**

**Foods to Emphasize**

**Breakfast**

- Greek yogurt (¾ cup) with blueberries
- Whole-grain toast with almond butter
- Glass of calcium-fortified plant milk taken *with* meals

**Lunch**

- Lentil or chickpea salad with olive oil + lemon
- Grilled chicken or tofu (small portion: 75–100 g)
- Side of steamed broccoli or green beans

**Dinner**

- Baked salmon (100 g) or plant-protein bowl
- Quinoa, farro, or barley
- Roasted vegetables: zucchini, bell peppers, carrots

**Snacks**

- Bananas, melons, pears
- Plain yogurt or cheese with meals
- Unsalted nuts (moderation for oxalate; pistachios and macadamias preferred)

**Foods to Reduce (Not eliminate completely)**

- Red meat, processed meats, large animal-protein portions
- Sugar-sweetened beverages, pastries, high fructose intake
- Very high-oxalate foods *in excess*: spinach, almonds, rhubarb, beets, peanuts
- Vitamin C supplements >500 mg/day (increase oxalate)

**Fluids**

- Aim for ≥2.5 L/day urine output (≈3 L fluid intake/day) unless contraindicated.
- Emphasize water, lemon water, or citrate-containing drinks.

**4. Safety Warning**

You did not report comorbidities, but note:

- **If you have heart failure, CKD stage ≥3, or are on potassium-sparing medications**, increasing fruits/vegetables and potassium-rich foods should be done with medical supervision.
- If you develop edema or fluid restrictions are advised, your hydration target must be individualized.

**Bottom Line**

Your high urine calcium is **not sodium-driven**. The most effective nutritional levers at this point are:

1. **Reduce total animal protein load.**
2. **Increase alkali intake (fruits/vegetables).**
3. **Ensure adequate calcium WITH meals.**

**Std + QA:**

Below is **general medical information**, not a substitute for personal medical care. Kidney-stone prevention must be tailored to your labs, stone analysis, medications, and comorbidities—so please review any changes with your clinician.

**🧂 Your sodium is already low — so what else moves urinary calcium?**

For **calcium oxalate stone formers**, high urine calcium despite low sodium means you likely need **additional levers** beyond salt restriction. The most evidence-supported dietary changes for **reducing urine calcium and supersaturation** are:

**✅ 1. Increase dietary calcium (1,000–1,200 mg/day) — *with meals***

This surprises many patients.
When eaten **with meals**, dietary calcium binds oxalate in the gut → **less oxalate absorption → lower urinary oxalate → lower calcium oxalate supersaturation**, even if urinary calcium is somewhat high.

**Good sources (with meals):**

- Dairy: milk, yogurt, cheese
- Non-dairy: calcium-set tofu, fortified almond/soy milk, leafy greens (but avoid high-oxalate greens like spinach)

**Avoid calcium supplements** unless prescribed — they can raise urinary calcium when taken without food.

**✅ 2. Moderate (not eliminate) animal protein**

Animal protein increases urinary calcium, lowers citrate, and acidifies urine.

**Recommendations:**

- Aim for **0.8–1.0 g protein/kg/day** (~60–75 g/day for most 50-year-old men).
- Replace part of animal protein with **plant proteins** (beans, lentils, tofu not made with magnesium sulfate).
- Limit especially: **red meat**, poultry, fish in large quantities.

**✅ 3. Increase fruits & vegetables (alkali load)**

Alkali intake lowers urinary calcium by reducing bone resorption and increases urinary citrate (a strong inhibitor of stones).

Most effective choices:

- Citrus (lemons, oranges)
- Melons
- Bananas
- Potatoes, squash
- Leafy greens except **high-oxalate ones** (spinach, Swiss chard, beet greens)

If urinary citrate is low, diet alone may help significantly.

**✅ 4. Ensure adequate fluid intake (≥2.5 liters urine/day)**

This is the single strongest predictor of reduced recurrence.

Best drinks:

- Water
- Citrus-based drinks (lemon water, orange water)
  Avoid:
- Sugary sodas (raise stone risk)
- Excess cola (phosphoric acid increases stone risk)

**✅ 5. Limit added sugars & processed foods**

High–glycemic load foods increase urinary calcium and reduce calcium absorption efficiency.

Reduce:

- Sodas, sweet teas
- Desserts
- Highly refined carbs

**🚫 What *probably won’t* help given your numbers**

**Further sodium restriction** – once urine Na is around ~100 mmol/day, reducing it further yields **minimal additional reduction of urine calcium**.

**Oxalate elimination alone** – unless your urinary oxalate is high, cutting oxalate excessively does little and may worsen diet quality.

**⭐ Highest-yield changes specifically for *your pattern* (low sodium but high urine calcium)**

Based on metabolic studies, the next most effective interventions are:

**🔹 1. Increase fruits/vegetables (alkali → ↓ urine calcium)**

A higher dietary alkali load can decrease urinary calcium significantly even when sodium is already low.

**🔹 2. Moderation of animal protein**

Especially effective when sodium is already optimized.

**🔹 3. Ensure dietary calcium intake is normal-to-high, not low**

Low calcium diets paradoxically worsen stone risk.

**🔹 4. Fluids to achieve ≥2.5 L urine/day**

Universal benefit.

**🧪 If urine calcium remains high despite optimal diet**

Your clinician may consider:

- **Thiazide diuretics** (chlorthalidone, HCTZ, indapamide)
- **Potassium citrate** (if citrate is low or urine pH is low)

Both have strong evidence for reducing recurrence.

**SB + QB:**

Below is a precise metabolic interpretation based on guideline-supported physiology and your provided values.

**1. Metabolic Diagnosis**

You have **persistent hypercalciuria (8.0 mmol/day ≈ 320 mg/day)** **despite normal urinary sodium (100 mmol/day)**.
When sodium is not elevated, **hypercalciuria is often driven by:**

- **High animal protein intake → acid load → increased bone calcium release + reduced renal calcium reabsorption.**
- **High refined carbohydrate / sugar intake → increased calciuresis.**
- **Low dietary calcium → increased oxalate absorption → compensatory renal calcium excretion.**
- **Possible hypercalciuric phenotype ("renal leak" or "absorptive hypercalciuria") independent of diet.**

Thus the most likely drivers for you are:
**Protein-induced calciuria**, **low calcium intake**, or **high simple sugar load**, not sodium.

**2. Key Dietary Modifications (Top Priorities)**

**① Moderate animal protein (0.8–1.0 g/kg/day)**

- High protein → increased fixed acid load → lower citrate + increased urine calcium.
- Replace some animal protein with plant sources (beans, tofu, lentils).

**② Ensure normal calcium intake (1,000–1,200 mg/day)**

- Prevents increased oxalate absorption.
- Consume calcium *with* meals to bind dietary oxalate.

**③ Reduce simple sugars**

- High glycemic foods (sweets, pastries, sugary drinks) increase urinary calcium excretion independently of sodium.

**Secondary priorities (if still hypercalciuric):**

- Moderate oxalate intake paired with calcium at meals.
- Avoid excess vitamin C (> 500 mg/day).
- Evaluate for thiazide diuretic therapy if dietary treatment is insufficient (per EAU/CUA guidelines).

**3. Sample Menu Suggestions**

**Foods to Emphasize**

- **Protein:**
  - Lentil or chickpea stews, tofu stir-fries, grilled chicken/fish in moderate portions (90–120 g).
- **Calcium sources (with meals):**
  - Yogurt (unsweetened), milk or fortified plant milks, cheese in small portions.
- **Low-oxalate fruits/vegetables:**
  - Bananas, melon, cauliflower, cabbage, mushrooms, squash.
- **Alkali-rich foods to increase citrate:**
  - Citrus fruits, potatoes with skin, leafy greens (in moderation for oxalate).

**Foods to Reduce or Avoid**

- **High-protein excess:**
  - Large steaks, multiple daily servings of red meat, high-protein shakes.
- **High-sugar items:**
  - Sweetened beverages, pastries, chocolate bars, high-fructose snacks.
- **High-oxalate without calcium:**
  - Spinach, almonds, beets, rhubarb, dark chocolate eaten *without calcium sources*.
- **Vitamin C supplements** (>500 mg/day) — can increase oxalate.

**4. Safety Warning**

If you have **CKD, heart failure, or are on potassium-sparing medications**, speak with your clinician before significantly increasing intake of potassium-rich fruits and vegetables for citrate enhancement.

**Std + QB:**

Here are the **evidence-based factors** to focus on when urine sodium is not elevated but **hypercalciuria and recurrent calcium-oxalate stones** persist. I’ll keep this at a **general medical-information level**, not personal medical advice.

**✅ 1. Dietary Calcium Intake (Aim for Adequate, Not Low)**

If calcium intake is **too low**, the gut absorbs more oxalate → **higher urine oxalate** → more calcium-oxalate stones.

**What to do:**

- Eat **1,000–1,200 mg/day** of dietary calcium (from food, not supplements unless advised).
- Best sources: dairy, fortified plant milks, yogurt, leafy greens.
- Take calcium **with meals** to bind oxalate in the gut.

**Why this matters:**
Hypercalciuria is often worsened by the *body releasing calcium* rather than by intake. Restricting calcium makes stones worse.

**✅ 2. Reduce Animal Protein Intake**

Even with normal sodium, **animal protein is a major driver of hypercalciuria** because it:

- increases urinary calcium,
- lowers urinary citrate (a natural inhibitor of stones),
- acidifies urine.

**What to do:**

- Limit meat (especially red meat), fish, and poultry.
- Use more plant-based proteins (beans, soy, lentils).
- Keep total animal protein intake around **0.8 g/kg/day** unless instructed otherwise.

**✅ 3. Increase Fruits & Vegetables (Alkali Load)**

A higher dietary acid load causes bone and kidney calcium release → hypercalciuria.

**Helpful foods:**

- Fruits, vegetables, potatoes, legumes.
- These raise urinary citrate and reduce calcium excretion.

**Especially helpful for calcium-oxalate stone formers:**
Citrus: lemons, limes, oranges.

**✅ 4. Ensure Adequate Hydration**

Even if the underlying cause is hypercalciuria, **dilution** is the strongest single preventive factor.

**Goal:**

- Produce **>2–2.5 L of urine/day**
  This typically requires **2.5–3 L of fluid intake/day**, more in heat/exercise.

**✅ 5. Control Oxalate Intake — But Not Excessively**

High-oxalate foods increase risk *only* when taken with low calcium.

**High-oxalate foods to *moderate*, not eliminate:**

- Spinach, almonds, beets, rhubarb, chocolate, tea.
- Take calcium **with** moderate-oxalate foods to reduce absorption.

**✅ 6. Evaluate for Hidden Causes of Hypercalciuria**

If sodium is low but hypercalciuria persists, the common contributors include:

**A. High animal protein intake**

(As discussed, strong driver.)

**B. Low calcium intake**

Paradoxically causes *higher* urine oxalate.

**C. High vitamin D supplementation**

Increases calcium absorption.

**D. Primary hyperparathyroidism**

If urine calcium is ~8.0 mmol/day (~320 mg/day), biochemical evaluation should ensure:

- PTH level is normal
- Serum calcium is normal

**E. Renal calcium leak / idiopathic hypercalciuria**

This is common, and responds to:

- Thiazide-type diuretics
- Low sodium intake (which you're already doing)

**✅ 7. Consider Thiazide Diuretics (Medical Therapy)**

If diet modification alone does not lower urine calcium, physicians may use:

- **hydrochlorothiazide**,
- **chlorthalidone**, or
- **indapamide**

These reduce calcium excretion **independent** of sodium intake.

(But this is a physician-managed decision.)

**📌 Summary of What You Should Prioritize**

Since sodium intake is already low:

**Most impactful dietary targets for you:**

1. **Adequate dietary calcium (1,000–1,200 mg/day).**
2. **Reduce animal protein intake.**
3. **Increase fruits/vegetables → raise citrate.**
4. **Avoid excess vitamin D or calcium supplements unless supervised.**
5. **Hydration to dilute urine.**
6. **Moderate high-oxalate foods and pair them with calcium.**

These are the main non-sodium levers that influence both **hypercalciuria** and **calcium-oxalate stone formation**.

**Case27:**

**SA + QA:**

Below is a **personalized, guideline-based calcium oxalate stone prevention plan** tailored to a patient with *recurrent stones despite normal 24-hour urine values* (a common scenario known as **idiopathic stone formation**). Anxiety is also an important factor because stress physiology can meaningfully influence hydration, urinary pH, and dietary patterns.

**1. Metabolic Diagnosis**

**Recurrent idiopathic calcium oxalate nephrolithiasis with normal metabolic evaluation**
→ This pattern is consistent with **baseline normal risk markers but suboptimal lifestyle factors**, often including:

- Inadequate or inconsistent fluid intake (especially during stress/anxiety)
- High dietary oxalate without protective calcium pairing
- High sodium intake increasing calcium excretion despite normal test snapshots
- High animal protein affecting urine citrate and pH intermittently
- “Day-to-day variation” not captured in a single 24-hr urine test

Anxiety may contribute via:

- Dehydration during attacks of stress (sympathetic activation reduces thirst)
- Skipping meals → low calcium → higher oxalate absorption
- Increased caffeine intake or intermittent high-oxalate “comfort foods”

**2. Key Dietary Modifications (Top 3 Priorities)**

**① Achieve consistent, high urine volume (2.0–2.5 L/day output)**

Your metabolic panel being “normal” does NOT eliminate low urine volume on typical days.
**Target fluid intake: ~2.7–3.0 L/day**, adjusted upward in hot weather/exercise.

**Practical strategy:**
Use a 1-L bottle and ensure **three fills per day**, spacing them evenly (especially morning and early afternoon when stones tend to form).

**② Normalize sodium and animal protein intake**

Even with normal 24-hr urine calcium, **sodium and protein excess** can cause intermittent hypercalciuria.

- **Sodium goal: <2,000 mg/day** (EAU & CUA recommendation)
- **Protein goal: 0.8–1.0 g/kg/day**, with emphasis on plant proteins
  (Women often exceed animal protein without realizing it.)

**③ Ensure adequate dietary calcium with each meal (1,000–1,200 mg/day)**

NOT supplements—**dietary calcium**.
This binds oxalate in the gut and reduces stone risk even when oxalate levels look “normal.”

Examples: dairy or fortified plant milk **taken with meals**, not on an empty stomach.

**3. Sample Menu Suggestions**

**Foods to Prioritize**

**At Every Meal (calcium + low sodium + moderate protein)**

- ½ cup **Greek yogurt** or fortified soy milk **with meals**
- **Leafy greens** (low-oxalate options: kale, bok choy, romaine)
- **Plant proteins:** lentils, beans, tofu, edamame
- **Citrus fruits** (natural citrate): lemon water, oranges, clementines
- **Whole grains:** brown rice, oats, barley

**Low-Oxalate, Stone-Safe Snacks**

- Fresh fruit (apple, pear, melon)
- Cheese stick + crackers (low sodium)
- Hard-boiled egg
- Low-sodium popcorn

**Foods to Limit (not avoid)**

**High-Oxalate Foods**

Limit to small portions *paired with calcium*:

- Spinach
- Almonds/almond flour
- Beets, Swiss chard
- Dark chocolate/cacao
- Raspberries
- Sweet potatoes

**High-Sodium Foods**

- Restaurant & fast-food meals
- Packaged soups, ramen
- Deli meats
- Pickles, olives
- Salted snacks (chips, pretzels)

**Animal Protein**

Keep moderate:

- 3–4 oz portions of chicken/fish/meat; avoid >1 large portion per day.

**A Sample Day of Eating**

**Breakfast**

- Oatmeal cooked in fortified oat milk
- 1 serving berries (low-oxalate options: blueberries or strawberries)
- Lemon water

**Lunch**

- Quinoa bowl with chickpeas, roasted vegetables
- Side of yogurt or cheese (calcium pairing)

**Snack**

- Apple + handful of pumpkin seeds
- Herbal tea or water

**Dinner**

- Grilled salmon or tofu
- Brown rice
- Steamed broccoli or kale
- Citrus fruit for dessert

**Before Bed**

- 200–300 mL water (avoid excessive nighttime volume if it disrupts sleep)

**4. Safety Warning (related to anxiety)**

Your anxiety matters physiologically:

- Anxiety can **reduce fluid intake** for hours at a time → significant stone risk.
- Caffeine overuse (common in anxiety) increases urine calcium and lowers urine pH.
- Skipping meals decreases dietary calcium → **increases oxalate absorption**.

**Practical mental-health linked strategies:**

- Set **hydration alarms** during busy/anxious periods.
- If anxiety causes nausea or reduced intake, choose **hydration with electrolytes** (low-sodium options such as diluted fruit juice or coconut water).
- Avoid using caffeine as a coping strategy—limit to **≤1–2 cups/day**.

**Std + QA:**

Below is **general, educational guidance** about kidney-stone prevention. Because you mentioned recurrent calcium oxalate stones and anxiety, I’ll explain what is typically recommended *even when metabolic urine studies are normal.*
This is **not medical advice** and cannot replace a clinician who knows your full history.

**⭐ Why stones recur even with “normal” urine tests**

Many people with repeat calcium oxalate stones have **subclinical risks** that don’t show up on one 24-hr urine test—such as inconsistent hydration, dietary patterns, or genetic predisposition. Prevention focuses on *universal, evidence-based habits*.

**🥤 1. Hydration: the most powerful intervention**

Even in “normal” urine chemistries, low urine volume is a major driver of stones.

**Goal:**

- Produce **≥2.5 L of urine/day** → typically requires **3 L of fluid/day** (more if you exercise or live in a hot climate).
- Clear to pale-yellow urine is your best indicator.

**Tips for people with anxiety:**

- Set *gentle* hydration cues (a water bottle with time markers, phone reminders).
- Choose fluids throughout the day to avoid feeling overwhelmed.

Acceptable fluids: water, herbal tea, diluted lemonade, sparkling water.
Avoid: heavy soda intake and very strong iced teas (high oxalate).

**🥗 2. Calcium intake: *Do NOT* restrict it**

This surprises many stone formers.

**Why:** Normal dietary calcium binds oxalate in the gut and prevents absorption.

**Goal:** ~1,000–1,200 mg/day from food (NOT supplements unless prescribed).

Good choices:

- Dairy (milk, yogurt, cheese)
- Fortified almond/soy milks
- Leafy greens *except* high-oxalate ones (spinach, beet greens)

**Key pairing strategy:**
Eat calcium *with* your meals, especially higher-oxalate foods.

**🌱 3. Moderate oxalate—not eliminate it**

You don’t need a very low-oxalate diet, just moderation.

**Foods high in oxalate to limit (not ban):**

- Spinach, beets, Swiss chard
- Almonds and almond flour
- Rhubarb
- Buckwheat, bran cereals
- Dark chocolate (moderation is ok)
- Black tea in excess

**Better choices:**

- Kale, lettuce, broccoli
- Oats, quinoa, white rice
- Low-oxalate fruits (bananas, melons, grapes)

**⚖️ 4. Sodium reduction: critical even with normal tests**

Sodium increases urinary calcium.

**Goal:** <2,000 mg/day
(About one teaspoon of salt total from all foods.)

Practical tips:

- Choose “low-sodium” packaged foods.
- Limit restaurant meals.
- Cook with herbs, lemon, garlic instead of salt.

**🍗 5. Protein balance**

Excess animal protein increases stone risk by lowering citrate and increasing calcium.

**Goal:**

- ~0.8–1 g/kg/day for most people
- Emphasize **plant-based proteins** part of the time
- Don’t follow high-protein diets (keto, carnivore)

**🍋 6. Citrate: natural stone inhibitor**

Citrate binds calcium and reduces stone formation.

Ways to boost citrate naturally:

- **Lemon or lime juice** daily (e.g., 2–4 tbsp mixed into water)
- Eat plenty of fruits and vegetables
- If anxiety or GI issues make citrus uncomfortable, try potassium-rich fruits and vegetables (bananas, avocados, leafy greens).

Prescription potassium citrate is used when citrate is low — but your 24-hr urine was normal, so lifestyle citrate may be enough.

**🩺 7. Supplements: what to avoid or use cautiously**

- **Vitamin C supplements** → avoid doses >500 mg/day (convert to oxalate).
- **Turmeric / curcumin supplements** → can raise oxalate.
- **Calcium supplements** → only if ordered by a clinician; take with meals.
- **Vitamin D** → safe if monitored.
- **Magnesium** → sometimes beneficial but not typically needed unless deficient.

**😟 8. Anxiety connection & practical strategies**

Anxiety can worsen stone recurrence indirectly:

- Dehydration on stressful days
- Restrictive or erratic eating patterns
- Increased caffeine or tea consumption
- Avoidance of medical follow-up due to overwhelm

Helpful approaches:

- Pair hydration with grounding techniques (deep breathing while drinking).
- Use predictable meal patterns to keep calcium + oxalate balance steady.
- Replace high-oxalate, high-caffeine coping foods with gentler options.
- Discuss anxiety management with your clinician—treating anxiety improves preventive behaviors.

**🚦 9. When to follow up despite “normal” tests**

You should talk to your urologist or nephrologist if:

- Stones are recurring yearly or more often
- Stones grow >5 mm
- You’ve never had a **repeat** 24-hr urine (results can vary)
- You suspect GI issues (celiac, IBS, inflammatory bowel disease), since these alter oxalate absorption even with normal tests

**👍 Summary: Your core prevention plan**

**Daily habits:**

- Drink enough to produce 2.5 L urine/day
- Eat normal calcium (1,000–1,200 mg/day) with meals
- Moderate—not eliminate—oxalate
- Keep sodium <2,000 mg/day
- Limit animal protein; increase produce
- Add lemon/lime or citrate-rich foods
- Avoid high-dose vitamin C and turmeric supplements
- Manage anxiety with routines that support hydration and nutrition

**SB + QB:**

Below is a **stone-specialist, guideline-based answer** tailored for a patient with **recurrent calcium oxalate nephrolithiasis but normal metabolic evaluation** (a common and frustrating scenario).
Since you did **not** provide your exact 24-hour urine values, I will address this as **“idiopathic recurrent CaOx stone disease with a normal metabolic profile.”**

**1. Metabolic Diagnosis**

**Recurrent idiopathic calcium oxalate stone disease with no identifiable metabolic abnormality on routine 24-hour urine testing.**
This is common—up to **40%** of recurrent CaOx stone formers have “normal” 24-hour urine studies.
The clinical significance:

- Your “normal” does **not** mean “optimized.”
- Small deviations within the normal range can still allow crystallization.
- Many patients have *behavior-based* stone risk (fluid timing, sodium load, dietary pattern, bowel habits) that does not show up clearly on a single 24h urine test.

**2. Key Dietary Modifications (Top 3 Priorities)**

These apply **even when the metabolic evaluation is normal**, per EAU + CUA guidelines.

**(1) Increase fluid intake to achieve >2.5 L urine/day**

This is *the single strongest intervention* for recurrent CaOx stone prevention.

- This often requires drinking **3–3.5 L/day** depending on sweat and climate.
- Spread intake across the day and include a **large glass before bed**.

**(2) Reduce sodium intake to <2,000 mg/day**

Even with normal calciuria, sodium reduction:

- Lowers urinary calcium excretion by 20–25%.
- Improves citrate excretion modestly.
- Is a cornerstone intervention for “idiopathic” stone formers.

Practical sodium triggers:

- Restaurant meals
- Packaged snacks
- Bread, deli meats, canned soups
- Fast food (often >2,500 mg per meal)

**(3) Maintain normal dietary calcium (1,000–1,200 mg/day) *with meals***

Even when labs are normal, this remains protective and **prevents oxalate hyperabsorption**.
Calcium restriction is outdated and harmful in most CaOx stone patients.

Best choices:

- Dairy: yogurt, milk, kefir, cheese
- Non-dairy fortified alternatives
- Calcium with **oxalate-rich meals** (binds oxalate in the gut)

**3. Sample Menu Suggestions**

**✔️ Foods to Favor**

**Breakfast**

- Oatmeal with milk or fortified almond milk
- Greek yogurt + berries
- Whole grain toast with avocado (light salt)

**Lunch**

- Grilled chicken or tofu bowl with rice, squash, cucumber, carrot
- Homemade vinaigrette (no bottled high-sodium dressings)
- Low-sodium tortilla wrap with turkey + spinach

**Dinner**

- Salmon, cod, or lentils
- Roasted vegetables (zucchini, cauliflower, broccoli)
- Brown rice or quinoa
- Calcium-containing drink with the meal (milk/yogurt) if meal contains oxalate

**Snacks**

- Fresh fruit (except very high-oxalate: avoid dried figs, raspberries in excess)
- Cheese sticks or yogurt cups
- Unsalted nuts (small portions—almonds are high in oxalate, but okay in moderation *with* calcium-containing foods)

**🚫 Foods to Limit**

- Excessive **spinach**, **almonds**, **rhubarb**, **beets**, **sesame**, **soy flour**, **chocolate**
- High-sodium foods (frozen dinners, restaurant meals, chips, canned soup)
- High-dose vitamin C (>500 mg/day → converts to oxalate)
- Mega-protein diets (>1.3 g/kg/day)
- Tea > 4 cups/day if you are a strong tea drinker (oxalate load)

**4. Safety Warning**

Since you did not list comorbidities, these general cautions apply:

- If you have **heart failure, CKD, or are on fluid restrictions**, do **not** increase fluids beyond your provider’s instructions.
- If you take **loop diuretics** (e.g., furosemide), these raise urinary calcium—speak to your clinician.
- If you take **topiramate**, it lowers urinary citrate; therapy may require citrate supplementation.

**Next Steps (What I Recommend Clinically)**

These are the most important follow-up actions for *recurrent stones despite a normal workup*:

**1. Repeat 24-hour urine evaluation, ideally twice**

Stone risk can vary day-to-day. Studies show that **40–60% of “normal” first tests reveal abnormalities on repeat testing**, especially if diet was atypical that day.

**2. Verify stone composition at each event**

A shift from CaOx → CaP changes management dramatically.

**3. Consider potassium citrate even with “normal” citrate**

If stones persist, low-normal citrate (e.g., 400–500 mg/day) can still be insufficient.
This is an accepted approach for idiopathic CaOx recurrence.

**4. Perform a detailed dietary review**

Many hidden oxalate sources and sodium excesses are not obvious to patients.

**5. Evaluate bowel habits**

Chronic diarrhea, IBS, or fat malabsorption dramatically increase oxalate absorption (enteric hyperoxaluria), even when urine looks “normal-ish.”

**Std + QB:**

Here’s a clear, **evidence-based, non-medical-diagnosis** overview of what is typically recommended for someone with **recurrent calcium oxalate stones despite a “normal” metabolic evaluation.”**
This is general guidance only — it cannot replace personalized medical care.

**✅ 1. Most Important Next Steps**

**1) Review whether your evaluation was complete**

A “normal metabolic evaluation” usually refers to a **24-hour urine study plus blood tests**, but many people only receive partial testing. Ask your clinician whether the evaluation included:

- **24-hour urine volume**, calcium, oxalate, citrate, sodium, uric acid, pH
- **Serum calcium**, PTH (if needed), uric acid, bicarbonate
- **Stone analysis** (to confirm composition each time)

If any of these were missing or borderline, repeating or completing the study is reasonable.

**2) Verify the quality of the 24-hour urine collection**

Many “normal” tests are actually **under-collections** (low urine volume falsely lowers other values).
Signs a collection may not be accurate:

- Total urine volume < 1.5 L
- Very low creatinine excretion (suggests under-collection)

If your results were “normal” only because the collection was incomplete, stones can still form.

**3) Aim for the #1 proven prevention: *High urine volume***

Increasing fluid intake is the single strongest intervention for nearly all stone-formers.

- **Goal urine output: ≥ 2.5 liters/day**
- This usually requires **3 liters/day fluid intake**, more in hot climates or with exercise.

Even people with normal metabolic findings reduce recurrence sharply with this step alone.

**4) Review medications & supplements**

Some supplements and drugs increase stone risk:

- **Vitamin C > 500 mg/day** (raises urinary oxalate)
- **High-dose vitamin D + calcium supplements** (calcium *with meals* is usually fine; supplements on empty stomach raise urinary calcium)
- **Loop diuretics**, topiramate, high-dose PPIs, etc.

If you take any of these, discuss alternatives with your clinician.

**🍎 2. Most Important Diet Changes for Calcium Oxalate Stone Prevention**

Even with normal lab results, these diet measures significantly reduce stone formation.

**1) Drink enough fluids (the cornerstone)**

- Goal: clear or pale-yellow urine
- Spread fluids throughout the day
- Include water as the main beverage
- Limit cola and high-sugar drinks

**2) Maintain NORMAL dietary calcium (don’t restrict it!)**

Many people mistakenly lower calcium intake, which increases stone risk.

- **Goal: 1,000–1,200 mg/day**, from food
- Best sources: dairy, fortified plant milk, yogurt, cheese
- **Have calcium with meals**, especially if the meal contains oxalate → calcium binds oxalate in the gut and prevents absorption.

**3) Reduce high-oxalate foods *only if you consume them frequently***

You do NOT need to eliminate oxalate totally. Focus on moderation.

Highest-oxalate foods include:

- Spinach
- Almonds & nuts
- Beets
- Rhubarb
- Swiss chard
- Soy products
- Dark chocolate
- Wheat bran
- Black tea (in large amounts)
- Sweet potatoes

Tip: If you eat these foods, **pair them with a calcium source** (e.g., yogurt, cheese, fortified plant milk, or taking the food with a meal).

**4) Lower sodium intake**

Sodium increases urinary calcium.

- **Target: < 2,000 mg/day sodium**
- Avoid: processed foods, fast food, boxed snacks, canned soups
- Choose fresh ingredients when possible

This is one of the most effective diet changes for people whose labs look “normal.”

**5) Moderate animal protein**

High animal protein → more urinary calcium, more uric acid, lower citrate.

- Limit to **1–2 servings/day**
- Favor plant-based proteins or fish/chicken over red meat
- Avoid very high-protein diets (keto, carnivore-style eating)

**6) Increase fruits & vegetables (natural citrate sources)**

Citrate protects against stones.

- Emphasize: citrus (lemon, lime, orange), melons, leafy greens (other than spinach), bananas
- Lemon/lime water is helpful (but not a replacement for prescription potassium citrate if medically indicated)

**7) Avoid excessive sugar and cola beverages**

- Cola contains phosphoric acid → raises stone risk
- High-sugar diets increase urinary calcium

**🔍 3. Should you repeat testing or consider medication even with “normal” labs?**

Possibly — depending on your history.

Red flags that often justify **repeat 24-hour urine testing or referral to a stone specialist**:

- ≥ 3–4 stone episodes
- Bilateral stones
- Stones appearing despite lifestyle changes
- A “normal” study done years ago
- Stones forming rapidly (less than 1 year between episodes)

Even when labs look normal, some clinicians consider **low-dose thiazide therapy**, **potassium citrate**, or combination treatment for very recurrent calcium stones — **but only after proper evaluation.**

**🧘‍♂️ 4. Anxiety About Recurrence**

Recurrent stones are understandably stressful. The key thing to know is:

- Many people with “normal” initial testing find a modifiable factor on repeat testing.
- Even when all tests remain normal, **hydration + sodium restriction + normal calcium intake** reduces recurrence by 50–60%.

Feeling anxious does not mean you're missing something serious — this pattern is common, and manageable.

**📌 Summary: The Most Important Actions**

**Do immediately**

- Increase fluids to ≥ 2.5 L urine/day
- Maintain normal dietary calcium with meals
- Limit sodium
- Avoid high-dose vitamin C and unnecessary supplements
- Moderate animal protein

**Discuss with your clinician**

- Whether your previous evaluation was complete
- Whether a repeat 24-hour urine test is appropriate
- Review of medications & supplements
- Whether referral to a nephrologist/urologist specializing in stones is warranted

**Case28:**

**SA + QA:**

Below is your **personalized, guideline-based metabolic plan**.

**1. Metabolic Diagnosis**

Your urine pH of **5.3** indicates **markedly acidic urine**, which is the primary driver of **uric acid stone formation**.
In people with **type 2 diabetes**, insulin resistance impairs renal ammonium production, keeping urine persistently acidic—**the strongest risk factor for uric acid stones**.

So your metabolic diagnosis is:

**“Uric acid stone disease driven by persistently acidic urine related to insulin resistance.”**

**2. Key Dietary Modifications (Top Priorities)**

**1) Increase Alkali Intake to Raise Urine pH (Goal pH 6.2–6.8)**

Foods naturally rich in potassium alkali raise urine pH and citrate.
Best dietary sources include: vegetables, fruits, and legumes.

**2) Reduce Animal Protein Load**

Animal protein increases acid load → lowers urine pH → increases uric acid production.

Target:

- **≤0.8–1.0 g protein/kg/day**, with **more plant protein**.

**3) Reduce Fructose and Simple Carbohydrates (critical in diabetes)**

Fructose increases uric acid synthesis and worsens insulin resistance.
Avoid sweetened beverages, fruit juices, desserts, and excessive tropical fruits.

**3. Sample Menu Suggestions**

**Foods to Prioritize (Raise pH + Improve Diabetes)**

- **Vegetables**: spinach, kale, broccoli, cauliflower, zucchini, green beans.
- **Fruits (low-glycemic)**: berries, apples, pears, kiwi.
- **Plant proteins**: lentils, chickpeas, tofu, tempeh.
- **Whole grains**: quinoa, barley, steel-cut oats.
- **Alkalinizing beverages**: lemon water, mineral waters with bicarbonate (if tolerated).

**Foods to Limit or Avoid (Lower pH / Increase Uric Acid)**

- **Red meat & organ meats** (high purine load).
- **Shellfish** (high purine).
- **Sugary beverages / juices** (rapidly spike uric acid).
- **High-fructose items**: honey, agave, soda, sweet snacks.
- **High-fat meals** (worsen insulin resistance, indirectly lowering urine pH).

**Sample Daily Menu for Uric Acid Stone Prevention**

**Breakfast**

- Steel-cut oatmeal with blueberries & chia seeds
- Unsweetened green tea or lemon water

**Lunch**

- Large salad with mixed greens, chickpeas, olive oil, lemon
- Grilled chicken breast or tofu
- Sparkling alkaline water

**Snack**

- Handful of almonds
- 1 piece of low-glycemic fruit (apple, pear)

**Dinner**

- Baked salmon or lentils
- Roasted vegetables (cauliflower, asparagus, zucchini)
- Side of quinoa or barley

**Avoid for Dinner: Beef, shellfish, beer, or dessert → all worsen nighttime urine acidity.**

**4. Safety Warning**

Since you have **type 2 diabetes**:

- Choose fruits with **low glycemic load** and monitor glucose response.
- If you take **SGLT2 inhibitors**, hydration needs may increase—avoid dehydration.
- If you have **CKD**, high-potassium foods may require limits—check potassium levels with your clinician before making major dietary changes.

**Std + QA:**

Here’s guidance tailored to **uric acid stone prevention** *plus* your **type 2 diabetes** and **low urine pH (5.3)**. This is **general educational advice**, not a substitute for care from your clinician.

**✅ Key Goal: Raise Your Urine pH**

Uric acid stones form when urine is **too acidic**.
A pH of **5.3** is strongly associated with stone formation.
Diet can help move urine pH toward **6.0–6.5**, which greatly reduces risk.

**⭐ Most Effective Diet Changes**

**1. Increase Fruits & Vegetables (Especially Alkalinizing Ones)**

These raise urine pH naturally.

Prioritize:

- Citrus: **lemons, limes, oranges, grapefruit**
- Melons: **cantaloupe, honeydew, watermelon**
- Green vegetables: **spinach, kale, broccoli, bok choy**
- Other alkalinizing veggies: **cauliflower, zucchini, bell peppers, cucumbers**

**Why helpful for you:**
Citrus provides **natural citrate**, which increases urine pH and reduces stone formation *without raising blood sugar dramatically when eaten whole*.

**Diabetes tip:** Choose whole fruits, not juice, and pair fruit with protein to reduce glucose spikes.

**2. Reduce Intake of High-Purine Animal Proteins**

Purines increase uric acid production, lowering urine pH.

Limit:

- Red meat
- Organ meats (liver, kidney—very high purine)
- Shellfish (shrimp, crab, lobster)
- Sardines, anchovies

Better options:

- **Plant proteins**: beans, lentils, tofu
- **Low-purine proteins**: eggs, most fish (e.g., salmon, cod), white meat poultry in moderation

**Diabetes tip:** Beans and lentils improve insulin sensitivity.

**3. Limit Fructose and Added Sugars**

Fructose increases uric acid production and worsens diabetes.

Avoid or minimize:

- Soft drinks, especially **HFCS-containing sodas**
- Sweetened teas and sports drinks
- Processed desserts, pastries, candies
- Fruit juice (prefer whole fruits)

**4. Increase Fluid Intake**

Aim for enough fluid to produce **≥2–2.5 liters of urine per day**.

Best choices:

- Water
- Flavored water with lemon or lime
- Herbal teas (unsweetened)

Avoid:

- Sugary beverages
- Excess alcohol (especially beer—raises uric acid)

**5. Choose Better Carbohydrates**

Poorly controlled blood sugar increases uric acid.

Focus on:

- Whole grains: oats, quinoa, barley
- Non-starchy vegetables
- Beans and lentils
- Nuts and seeds

Limit:

- White bread, white rice, pasta
- Potatoes and large portions of starchy foods

**6. Reduce Sodium**

High sodium increases calcium excretion and can worsen stone risk.

Aim for **<2,300 mg/day** (or **<1,500 mg/day** for diabetes/hypertension).

Avoid:

- Fast food
- Cured meats
- Salty snacks
- Canned soups unless labeled low-sodium

**7. Moderate Alcohol**

Alcohol—especially **beer**—increases uric acid.

Safer choices (if you drink at all):

- Small amounts of wine
- Avoid binge drinking

**🌟 Optional but Often Very Effective**

**Daily alkalinizing beverages**

- 1–2 tablespoons of **lemon juice** in water
- Water infused with **baking soda is NOT recommended** without clinician approval due to sodium load.

**Note:** Many people with uric acid stones ultimately need **potassium citrate**, but only a clinician can prescribe this and monitor potassium levels.

**📌 Summary: Best Diet Strategy for You**

To reduce recurrence of **uric acid stones** while managing **type 2 diabetes**:

**Eat more:**

- Citrus fruits, melons, leafy greens, and vegetables
- Plant proteins (beans, lentils, tofu)
- Whole grains
- Water and citrus-infused water

**Eat less:**

- Red meat, organ meats, shellfish
- Sugary foods and beverages, especially HFCS
- White breads, refined carbs
- High-sodium foods
- Beer and excessive alcohol

**SB + QB:**

Below is a **personalized, guideline-based plan** integrating both **uric acid stone prevention** and **diabetes management**, following EAU/CUA dietary principles for urolithiasis.

**1. Metabolic Diagnosis**

The patient has **uric acid stone disease driven by persistently acidic urine (pH 5.3)**.
Low urine pH is the *primary* driver of uric acid stone formation and is typically associated with **insulin resistance and type 2 diabetes**, which impair renal ammonium production and acid excretion.

**2. Key Dietary Modifications (Top Priorities)**

**Priority 1 — Raise Urine pH via Alkali-Rich Diet (But Carb-Controlled)**

Focus on **low-glycemic, high-alkali fruits and vegetables** to increase urinary bicarbonate and citrate → this raises urine pH toward the target **6.0–6.5**.

**Priority 2 — Reduce Purine Load**

Limit **animal proteins**, especially high-purine sources that increase uric acid: red meat, organ meats, shellfish.

**Priority 3 — Optimize Carbohydrates for Diabetes + Stone Prevention**

Avoid high-sugar foods which:

- Worsen insulin resistance → lower urine pH
- Increase endogenous uric acid production
  Choose **slow-digesting, fiber-rich carbs** instead.

**3. Sample Menu Suggestions**

**Foods & Drinks to Focus On (Raise pH + Support Diabetes Control)**

**Alkali-rich vegetables (low-glycemic)**

- Spinach, kale, broccoli, cauliflower, zucchini, green beans
- Salads with olive oil + lemon (citrate-rich)

**Low-glycemic fruits (safe for diabetes, alkalinizing)**

- Berries (blueberries, raspberries, strawberries)
- Cherries
- Citrus (lemons, limes, grapefruit segments if allowed with meds)

**Plant-based proteins (alkali-producing)**

- Tofu, lentils, chickpeas, edamame (moderate portions for uric acid balance)

**Low-purine animal protein choices**

- Skinless poultry
- Eggs
- Low-fat dairy (yogurt, milk) — also protective for stones

**Hydration targets**

- Water
- Lemon-infused water
- Herbal teas
  (Aim for pale-yellow urine unless fluid restriction is medically indicated.)

**Foods & Drinks to Avoid or Minimize**

**High-purine foods (increase uric acid synthesis)**

- Red meat (beef, lamb, pork)
- Organ meats (liver, kidney, sweetbreads)
- Anchovies, sardines, shellfish
- Meat broths and gravies

**Foods that acidify urine**

- Large portions of animal protein
- Processed meats
- High-fat, low-vegetable diets

**Foods that worsen diabetes AND uric acid levels**

- Sugary drinks (soda, sweetened iced tea, energy drinks)
- Fruit juices
- Pastries, candy, refined carbs (white bread, white rice)

**Alcohol (strong acidifier)**

- Beer → highest risk (purines + acid load)
- Spirits also lower urine pH
- Wine is less problematic, but still best minimized

**Example Daily Menu (Balanced for Stones + Diabetes)**

**Breakfast**

- Greek yogurt topped with berries + chia seeds
- 1 slice whole-grain toast
- Water with lemon

**Lunch**

- Grilled chicken salad with kale, spinach, cucumbers, tomatoes
- Olive-oil + lemon dressing
- Sparkling water

**Snack**

- Small handful of almonds OR carrot sticks with hummus

**Dinner**

- Baked salmon or tofu
- Steamed broccoli + cauliflower
- Quinoa or barley (low-glycemic, alkalinizing)
- Herbal tea

**Foods to swap**

| **Instead of** | **Choose** |
| --- | --- |
| Red meat | Poultry, tofu, or fish in moderate portions |
| White rice | Quinoa, farro, or lentils |
| Fruit juice | Whole citrus or berries |
| Soda | Water with lemon or unsweetened tea |

**4. Safety Warning**

Since you have **diabetes**, be cautious with:

- High-sugar fruits (mango, pineapple, grapes)
- Fruit juices (cause glucose spikes + lower urine pH)
- Overuse of potassium-rich alkalizing foods or supplements **if you have kidney disease** — if CKD is present, potassium must be restricted.

If you take **SGLT2 inhibitors (e.g., Jardiance, Farxiga)**, monitor hydration more carefully to avoid dehydration.

**Std + QB:**

Below is **general educational guidance**, not a medical diagnosis or personalized medical plan. For kidney-stone prevention with **uric-acid stones**, **diabetes**, and **low urine pH (5.3)**, the core goals are:

1. **Raise urine pH** (more alkaline urine dissolves uric acid and prevents stones).
2. **Lower uric-acid load** from diet.
3. **Improve blood-sugar control** without worsening stone risk.

To help you visually, I’ve included images of recommended and avoided foods.

**🍋🫐 What to Focus On (Helps raise urine pH + friendly for diabetes)**

**1. Alkaline-forming fruits & vegetables**

These directly help raise urine pH.

- **Most vegetables**: spinach, kale, broccoli, cauliflower, cucumbers, zucchini, bell peppers
- **Low-sugar fruits**: berries, melon, peaches, apples, pears
- **Citrus fruits** (especially **lemon or lime**): despite acidic taste, they alkalinize urine.

**2. Plant-based proteins (instead of animal proteins)**

High animal protein lowers pH and increases uric acid.
Choose:

- Beans/lentils (in moderate portions if watching carbs)
- Tofu, tempeh
- Nuts & seeds (small portions for calories)

**3. Healthy drinks that raise pH**

- **Water** is essential: aim for steady hydration during the day.
- **Lemon water** or water flavored with citrus.
- **Herbal teas** (non-sweetened).
- **Carbonated water** (unsweetened) is fine.

**4. Low-glycemic whole grains (in moderate portions)**

Good for blood sugar and do not increase uric acid.
Examples: quinoa, barley, oats, buckwheat, whole-grain bread (in controlled portions).

**5. Low-fat dairy**

Can reduce stone risk and support diabetes control.

- Plain yogurt
- Milk
- Cottage cheese

**🍗🥩 What to Limit or Avoid (Raises uric acid or worsens diabetes)**

**1. High-purine animal proteins**

These significantly raise uric acid and acidify urine.
Avoid or sharply limit:

- **Red meat** (beef, lamb, pork)
- **Organ meats** (liver, kidney, heart)
- **Seafood high in purines**: anchovies, sardines, mussels, herring, trout, mackerel

**2. High-sugar foods and drinks**

These worsen diabetes AND increase uric acid production.
Avoid:

- Sugary beverages (soda, sweet tea, juices)
- Desserts, pastries, candies
- Foods with **high-fructose corn syrup** (HFCS)

**3. Large amounts of alcohol**

Alcohol—especially **beer**—raises uric acid and lowers urine pH.
If consuming alcohol, do so sparingly and avoid beer.

**4. Excess salt**

Salt increases calcium excretion and worsens metabolic control.
Avoid heavily processed foods, fast food, chips, canned soups with added salt.

**5. Very high-protein diets**

Even if low-carb, excess protein (especially animal protein) acidifies urine.
Moderate protein intake is key.

**🧂🫗 Additional habits that help both uric-acid stones & diabetes**

**Hydration strategy**

- Drink enough to produce at least **2–2.5 liters of urine per day**.
- Spread fluids throughout the day; dehydration concentrates uric acid.

**Weight management**

Losing weight gradually improves insulin resistance, lowers uric acid, and raises urine pH.

**Balance meals**

Pair carbohydrates with **fiber + protein + healthy fats** to prevent blood-sugar spikes.

**Consider potassium-rich foods**

They help alkalinize urine *and* support cardiovascular health (unless restricted due to kidney disease).
Examples: leafy greens, avocados, bananas (in moderation), potatoes, tomatoes.

**⭐ Putting it all together: A simple sample day**

**Breakfast:** Oatmeal with berries + a handful of nuts; lemon water
**Lunch:** Large vegetable salad with tofu or beans + olive oil vinaigrette
**Snack:** Greek yogurt or a small piece of fruit
**Dinner:** Grilled vegetables + moderate portion of quinoa or lentils
**Drinks:** Water, herbal tea, carbonated water

**Case29:**

**SA + QA:**

Below is a **patient-safe, guideline-aligned interpretation** without revealing internal reasoning steps.

**1. Metabolic Diagnosis**

Your 24-hour urine oxalate of **0.8 mmol/day** (~70 mg/day) is **elevated**, indicating **dietary hyperoxaluria**, which is common in vegan diets when intake of high-oxalate plants is not balanced with adequate calcium at meals.
Because you are vegan, the risk is **not due to dairy calcium restriction** but usually from:

- High intake of spinach, almonds, beets, chard, peanuts, soy products, or dark chocolate
- Insufficient calcium pairing with oxalate-containing meals (plant-based calcium sources)

You also have **calcium oxalate stone disease**, so controlling oxalate load and enhancing intestinal calcium binding are key.

**2. Key Dietary Modifications (Top Priorities)**

**1. Pair plant calcium with every moderate-oxalate meal (do NOT restrict calcium).**

- Aim for **500–600 mg/day of dietary calcium**, ideally **spread across meals**.
- Vegan calcium sources: calcium-set tofu, fortified soy/almond milk, fortified orange juice, sesame/tahini, chia seeds, kale, bok choy.

**2. Lower intake of the *very high oxalate* foods.**

You do **not** need to give up vegan eating, but rotating away from the highest-oxalate items reduces stone risk dramatically.

- Limit: spinach, Swiss chard, beet greens, almonds & almond flour, cashews, peanuts, soy nuts, rhubarb, beets, sweet potatoes, dark chocolate, black tea.

**3. Increase citrate and urinary volume through vegan-friendly sources.**

- Citrate-rich foods: lemon/lime juice, oranges, melons.
- Fluids: aim for **2.5–3 L/day**, water preferred.
- If you consume large amounts of plant protein, moderate portion sizes to reduce acid load (which lowers citrate).

**3. Sample Menu Suggestions**

**✔️ Low-oxalate vegan options (recommended)**

**Breakfast**

- Oatmeal with chia seeds, blueberries, fortified oat milk
- Lemon water on the side
- Calcium-fortified cereal with soy milk

**Lunch**

- Quinoa bowl with tofu (calcium-set), broccoli, carrots, zucchini, tahini dressing
- Sparkling water with lemon

**Dinner**

- Lentil stew with kale, mushrooms, and brown rice
- Side salad with romaine, cucumbers, avocado
- Calcium-fortified beverage

**Snacks**

- Pumpkin seeds, fresh fruit (melon, mango, berries), rice cakes with hummus

**❌ Foods to limit or rotate (very high oxalate)**

These are the biggest contributors to your elevated oxalate:

- Spinach, Swiss chard, beet greens
- Almonds, almond butter, almond milk, almond flour
- Soy nuts, large amounts of tofu NOT set with calcium
- Dark chocolate
- Rhubarb, beets, sweet potatoes
- Black tea (if consumed heavily)

You do **not** need to completely eliminate them—reducing frequency/portion sizes and pairing with calcium makes a major difference.

**4. Safety Warning**

Because you did not report CKD, heart failure, or potassium restrictions, the fluid and potassium-rich fruit/vegetable recommendations are **safe**.
If you have **bowel disease, malabsorption, or history of Roux-en-Y gastric bypass**, your oxalate management would differ—please let me know if any apply.

**Std + QA:**

Below is **general educational information**, not medical advice. Kidney-stone prevention should always be personalized with your clinician or dietitian—especially since you have known calcium oxalate stones and measured urine oxalate.

**🌱 Preventing Calcium Oxalate Stones on a Vegan Diet**

Your urine oxalate (**0.8 mmol/day ≈ ~70 mg/day**) is moderately elevated, but **you can stay vegan and still reduce stone risk**. The strategy is usually about *oxalate management, calcium timing, hydration, and sodium control*—not eliminating plant foods.

**1. 🍽️ Pair Plant Oxalate With Calcium at Meals**

In calcium oxalate stone formers, **dietary calcium lowers stone risk** by binding oxalate in the gut.

**Practical vegan calcium sources:**

Aim for **800–1,000 mg/day** from food + fortified products.

**Best options (lower oxalate):**

- Calcium-set tofu (look for *calcium sulfate*)
- Fortified soy, almond, or oat milk
- Bok choy, kale, napa cabbage
- Almond butter in small amounts (better tolerated when paired with calcium)

**Tip:**
Drink your fortified plant milk *with* higher-oxalate foods (beans, whole grains, nuts) so oxalate is bound in the gut before absorption.

**2. 🥗 Reduce Only *High-Oxalate* Plants — Not All**

You shouldn’t remove all oxalate-containing foods; just avoid the worst offenders.

**Most important foods to limit:**

- Spinach (extremely high)
- Swiss chard, beet greens
- Almonds & almond flour
- Cashews
- Peanuts
- Buckwheat
- Rhubarb
- Starfruit

**Foods that are vegan-friendly and lower in oxalate:**

- Lentils, split peas, most beans (except soy is moderate, peanuts high)
- Basmati or jasmine rice
- Quinoa (moderate—eat with calcium)
- Cauliflower, cabbage, mushrooms, peas
- Winter squash, zucchini
- Bananas, melon, pears, mango

You don’t need a low-oxalate diet—just a **targeted** one.

**3. 💧 Maximize Fluid Intake**

The strongest predictor of stone recurrence is **low urine volume**.

Goal:
**Urine output ≥ 2–2.5 L/day**
= usually **drink ~3 L/day**, more if sweating.

Tips:

- Carry a large water bottle
- Drink 250–500 mL before each meal
- Favor water, herbal teas, lemon water

**4. 🍋 Boost Citrate Intake (Alkali)**

Citrate inhibits calcium stones.

Good vegan sources:

- Lemon or lime juice (add to water daily)
- Oranges, mandarins
- Potatoes and other high-potassium vegetables

Target: juice of **½–1 lemon per day** in water (adjust for tolerance).

**5. 🧂 Reduce Sodium (Major for Calcium Stones)**

High sodium increases urinary calcium, raising stone risk.

Limit to **< 2,000 mg/day**:

- Minimize vegan processed foods (faux meats, soups, sauces)
- Cook with herbs, not salt
- Choose “low sodium” labeled products

**6. 🫘 Moderate Animal-Protein Equivalents**

You’re vegan already, so you avoid animal protein–induced acid load.
Still, **very high intake of plant protein powders or very high soy intake** can increase stone risk.

Guidelines:

- Avoid large amounts of **soy protein isolate** (protein shakes, mock meats)
- Whole soy foods (tofu, tempeh, soy milk) are fine

**7. 🥄 Consider Meal Timing + Food Prep**

- **Boiling high-oxalate vegetables** (not sautéing) reduces oxalate substantially.
- Soaking beans and lentils overnight reduces oxalate.
- Eat calcium-rich foods **with** each meal containing oxalate.

**👍 Example Vegan Stone-Smart Day**

**Breakfast:** Fortified soy milk + oats + chia (small amount) + fruit
**Lunch:** Lentil soup + whole-grain bread + side of steamed kale
**Snack:** Fruit + ½ cup calcium-set tofu or fortified yogurt alternative
**Dinner:** Tofu stir-fry with rice + bok choy + lemon water
**Avoid:** Spinach salad, almond-based snacks, high-sodium plant meats

**🎯 Summary: Key Steps for Your Case**

1. **Increase calcium at meals** (fortified drinks, calcium-set tofu).
2. **Limit only the highest-oxalate plants** (spinach, nuts, chard, buckwheat).
3. **Drink enough to produce 2–2.5 L urine/day.**
4. **Use lemon/lime regularly** to increase citrate.
5. **Keep sodium low.**
6. **Moderate soy protein isolates**, but whole soy is fine.

**SB + QB:**

Below is your tailored, guideline-based dietary plan for a **vegan calcium-oxalate stone former with high urinary oxalate (0.8 mmol/day ≈ 70 mg/day; elevated)**.
(Values referenced against EAU/CUA metabolic stone-prevention guidelines.)

**1. Metabolic Diagnosis**

**Primary abnormality: Hyperoxaluria** in the setting of a vegan diet likely driven by **high-oxalate plant foods**, **low dietary calcium at meals**, and possibly **excess vitamin C** or **gut-oxalate overabsorption**.

**2. Key Dietary Modifications (Top Priorities)**

**1) Reduce high-oxalate plant foods — not all plants, just the major offenders**

These foods can easily raise urine oxalate even in small servings.

**2) Add calcium *with meals* (NOT restrict it)**

For vegans, use **calcium-fortified foods** or **calcium citrate supplements (200–300 mg) with meals** to bind oxalate in the gut.
*This lowers oxalate absorption and is guideline-recommended.*

**3) Moderate nut & seed intake + diversify protein sources**

Many vegans rely heavily on nuts for protein/fat; this often drives oxalate higher. Replace with **soy/tofu, legumes, and whole grains**.

**3. Sample Menu Suggestions (Eat / Limit / Avoid)**

**A) Plant Foods to *Avoid or Strictly Limit* (High Oxalate)**

These are the biggest drivers of hyperoxaluria.

- **Spinach** (one of the highest oxalate foods)
- **Almonds, cashews, nut butters made from them**
- **Beets / beet greens**
- **Swiss chard**
- **Rhubarb**
- **Sweet potatoes**
- **Cocoa powder, dark chocolate**
- **Sesame seeds / tahini**
- **Buckwheat, amaranth (higher oxalate grains)**
- **Excess Vitamin C supplements (>200 mg/day)** — increases oxalate production

**Practical rule:** If it’s spinach-based, almond-based, or rhubarb-based → avoid.

**B) Plant Foods That Are *Moderate Oxalate* (OK in controlled portions)**

- Regular potatoes (not sweet potatoes)
- Soybeans, tempeh
- Lentils & chickpeas
- Quinoa
- Navy/Cannellini beans
- Raspberries
- Carrots
- Celery
- Whole-wheat products (1–2 servings/day fine)

**Tip:** Pair these foods with calcium at the same meal.

**C) Plant Foods That Are *Low Oxalate* (Keep + increase variety)**

**Low-oxalate vegetables**

- Kale, bok choy, broccoli, cauliflower
- Zucchini, cucumber, bell peppers
- Mushrooms
- Cabbage, lettuce, arugula
- Green beans, peas

**Low-oxalate fruits**

- Banana, mango, melon, grapes
- Apples, pears, blueberries
- Papaya (especially helpful due to low oxalate)

**Low-oxalate protein & carb sources**

- Tofu (especially **calcium-set**)
- Lentils/chickpeas (moderate but acceptable)
- Oats, barley, rice
- Seitan
- Fortified cereals

**D) How to Get Enough Calcium as a Vegan (Critical for Oxalate Binding)**

**Best options (aim ≥ 1000–1200 mg/day):**

- **Calcium-set tofu** (often 350–450 mg per serving)
- **Fortified almond, soy, oat, or pea milks** (300 mg per cup)
- **Fortified orange juice** (300 mg per cup)
- **Calcium supplements**: Calcium *citrate* 200–300 mg **with the two highest-oxalate meals** of the day.

**Guideline logic:** Calcium must be ingested **with** oxalate-containing foods to bind oxalate in the gut and prevent absorption.

**4. Example Daily Plant-Based Stone-Prevention Menu**

**Breakfast**

- Oatmeal with fortified soy milk
- Blueberries + banana
- 1 serving calcium-set tofu scramble with vegetables
  **Avoid:** spinach, almond milk, cocoa powder

**Lunch**

- Lentil soup
- Kale & red pepper salad
- Whole grain bread
- **Calcium source:** glass of fortified plant milk
  **Avoid:** beet salad, tahini dressing

**Snack**

- Fresh fruit, roasted chickpeas, or rice cakes
  **Avoid:** handful of almonds or almond butter

**Dinner**

- Stir-fry with tofu, broccoli, zucchini, mushrooms
- Rice or barley
- **Calcium citrate 200–300 mg if this meal includes moderate-oxalate foods**

**Dessert**

- Mango or melon
  **Avoid:** dark chocolate

**5. Safety Warning**

- If you take **vitamin C supplements**, keep total intake **<200 mg/day**, as higher doses significantly increase urinary oxalate.
- If you have **CKD, heart failure, or are on potassium-restricted diets**, please tell me—many vegan foods are potassium-rich and require adjusted recommendations.

**Std + QB:**

Below is **general educational information** about kidney-stone nutrition for people with **high urinary oxalate** on a **vegan diet**. It is **not personalized medical advice**; your own clinician or a renal dietitian should review any diet changes.

**🌱 Vegan Diet & High Urinary Oxalate**

**Goal:** Reduce oxalate absorption *and* keep enough calcium at meals so oxalate binds in the gut rather than in your kidneys.

Your value of **0.8 mmol/day urine oxalate (≈70 mg/day)** is above the usual target (<40–45 mg/day), so reducing dietary oxalate and pairing calcium with meals is a common strategy.

**✔️ Foods to Keep (Lower- and Moderate-Oxalate Plant Foods)**

These are typically safer staples:

**Lower-oxalate vegetables**

- Cauliflower, cabbage, bok choy
- Zucchini, cucumbers
- Mushrooms
- Lettuce, kale, broccoli (kale & broccoli are much lower in oxalate than spinach)

**Lower-oxalate fruits**

- Bananas
- Melons
- Mango
- Papaya
- Apples & pears
- Berries (blackberries moderately higher, but still ok in small portions)

**Protein sources**

- Tofu (especially calcium-set tofu — bonus calcium)
- Tempeh
- Seitan
- Most legumes in moderate portions (lentils, chickpeas, black beans are lower than soybeans)

**Grains**

- Quinoa
- Rice (white or brown—brown has some oxalate but still moderate)
- Barley, oats

These can stay as diet foundations.

**⚠️ Foods to Cut Back On (High-Oxalate Plant Foods)**

You don’t necessarily need to eliminate these, but reducing frequency and portion size often helps.

**Very high oxalate**

- **Spinach** (one of the highest—major driver of high urine oxalate)
- **Beets** and beet greens
- **Swiss chard**
- **Rhubarb**
- **Almonds & almond-based products** (almond milk, almond butter, almond flour)
- **Cashews**, peanuts to a lesser extent
- **Soybeans, soy nuts**, and certain soy products (moderate-to-high)
- **Buckwheat**, **teff**, amaranth
- **Potatoes (especially skins)**
- **Dark chocolate / cocoa**
- **Black tea** (switching partly to herbal teas helps)

**Moderately high**

- Sweet potatoes
- Raspberries
- Navy beans
- Tahini / sesame seeds

Often the biggest wins come from **reducing spinach, almonds, and beet greens**, which are extremely high.

**🧀 How to Get Calcium With Meals on a Vegan Diet**

(This is crucial—calcium binds oxalate in the intestines and prevents absorption.)

**⭐ Best vegan calcium sources to pair *with meals containing oxalate*:**

- **Calcium-set tofu (calcium sulfate)**
- **Calcium-fortified plant milks** (oat, soy, rice — avoid almond milk due to oxalate)
- **Calcium-fortified orange juice**
- **Calcium supplements taken *with* meals**, if your clinician agrees
  - Usually **calcium citrate 200–300 mg with meals** is used because it also increases citrate (a stone inhibitor), but ask your clinician first.

**Use calcium strategically**

Add one calcium source with *each* moderate/high-oxalate meal.
Examples:

- Lentil curry with a glass of fortified soy milk
- Stir-fry with calcium-set tofu
- Oatmeal with fortified oat milk
- Calcium supplement taken *only* with higher-oxalate meals (if advised by your doctor)

**💧 Other Helpful Habits**

**Hydration**

- Aim for enough fluid to produce **>2–2.5 L of urine/day** unless your doctor restricts fluids.

**Salt reduction**

- High sodium increases urinary calcium, raising stone risk.

**Don’t over-restrict healthy foods**

- Oxalate reduction doesn’t mean eliminating all nuts, beans, and greens—just the highest offenders.

**Citrate foods (stone-protective)**

- Lemon/lime juice in water
- Fruits and vegetables in general help maintain urine alkalinity and citrate

**📌 Sample Low-Oxalate Vegan Meal Pattern**

**Breakfast:** Oatmeal + fortified oat milk + banana
**Lunch:** Lentil & vegetable soup (no spinach) + whole-grain bread + glass of calcium-fortified juice
**Snack:** Carrot sticks + hummus
**Dinner:** Stir-fry with broccoli, tofu (calcium-set), rice
**Dessert:** Fruit or coconut yogurt

**Case30:**

**SA + QA:**

Below is a **simplified, patient-appropriate version** of your requested output structure, adapted to this limited dataset (no full 24-hour urine profile available). When you provide full labs, I will give the full guideline-level metabolic interpretation.

**1. Metabolic Diagnosis**

You are forming **calcium phosphate stones**, and you are on **Topiramate**, which commonly causes:

- **Chronic metabolic acidosis → Hypocitraturia (low citrate)**
- **Alkaline urine (high urine pH)**

Your urine **pH of 6.5** is elevated for stone prevention; calcium phosphate stones form easily when pH > 6.2.
So the working metabolic diagnosis is:

**“Calcium phosphate stone formation driven by topiramate-induced alkaline urine and likely hypocitraturia.”**

**2. Key Dietary Modifications (Top 3 Priorities)**

**1. Increase Citrate Intake Through Food (Not Supplements Yet)**

Topiramate lowers citrate, which normally stops stones from forming.

- Prioritize **citrate-rich, low-sugar** sources (lemons, limes).
- Avoid high-sugar juices (they worsen stone risk).

**2. Reduce Alkaline Load From Diet**

High urine pH increases calcium phosphate crystallization.
Reduce foods that push urine pH higher:

- Limit **very alkaline foods** in excess → spinach, kale, Swiss chard, beets, beet greens.
- Limit **plant-based milks with added calcium** (but keep normal dietary calcium!).

**3. Moderate Animal Protein + Keep Sodium Low**

Because both protein and sodium affect calcium excretion:

- Sodium target: **< 2,000 mg/day**
- Protein target: **0.8–1.0 g/kg/day**, avoid large single protein loads.

**3. Sample Menu Suggestions**

**✔ Foods that HELP**

- **Citrate sources:**
  - Water with freshly squeezed **lemon or lime**
  - Unsweetened lemonade made at home
- **Calcium from food (NOT supplements):**
  - Yogurt, kefir, cheese, milk (normal portions)
- **Lower-alkali vegetables:**
  - Zucchini, bell peppers, carrots, green beans
- **Proteins (moderate portions):**
  - Chicken, fish, eggs, tofu
- **Healthy grains:**
  - Oats, quinoa, whole wheat pasta

**✖ Foods to LIMIT for Calcium Phosphate Stone Formers**

- **Very alkaline greens:** spinach, Swiss chard, beet greens
- **High-sodium foods:** restaurant meals, chips, ramen, processed meats
- **Excessive plant milks** fortified with calcium (these raise dietary calcium load above natural amounts)
- **Large quantities of nuts** (high alkali load)
- **Baking soda or alkaline waters** (raise urine pH further)

**EXAMPLE DAY**

**Breakfast:**
Greek yogurt + berries; oatmeal with ground flax. Lemon water.

**Lunch:**
Grilled chicken, quinoa, roasted carrots and zucchini. Water with lemon.

**Snack:**
Apple + small handful of almonds.

**Dinner:**
Baked salmon, brown rice, steamed green beans. Herbal tea (not alkaline water).

**4. Safety Warning**

Topiramate can cause **metabolic acidosis** and **hypocitraturia**, increasing your stone risk.
Before using **potassium citrate supplements**, your clinician must check:

- Serum potassium
- Bicarbonate level
- Kidney function

Potassium citrate can **raise urine pH further**, which is dangerous for calcium phosphate stones unless carefully titrated.

**Std + QA:**

Here is **safe, evidence-based, non-personalized** guidance tailored to the *scenario you described*, with the reminder that you should review all changes with your clinician, especially because **topiramate can increase urine pH and the risk of calcium phosphate stones**.

**🥗 Diet considerations for calcium phosphate stones (especially with higher urine pH)**

Your situation has three key features:

1. **Calcium phosphate stones** (often form in **higher-pH urine**)
2. **Urine pH of 6.5**, which is on the alkaline side
3. **Topiramate use**, which raises urine pH and lowers citrate levels

Calcium phosphate stones are different from calcium oxalate stones, and some common “kidney stone tips” do *not* apply the same way. Here’s what makes dietary sense for your situation.

**✅ What to *prioritize***

**1. Keep a normal—not low—calcium intake**

Aim for **1,000–1,200 mg/day** from food (yogurt, milk, fortified plant milks, cheese).
Low calcium *increases* intestinal phosphate and oxalate absorption, which can worsen stones.

**Important:** Take calcium **with meals** so it binds dietary phosphate and oxalate in the gut.

**2. Increase dietary citrate**

Topiramate tends to **lower citrate**, which normally prevents stones.

Eat more citrate-rich foods:

- Lemons, limes, lemonade (low-sugar preferred)
- Oranges/mandarins
- Melons

This may help offset topiramate’s effect, though your clinician may consider potassium citrate if dietary measures aren’t enough.

**3. Drink enough fluid to produce >2–2.5 L of urine/day**

This is one of the strongest ways to reduce recurrence for any stone type.

Water is best; aiming for pale-yellow urine is a practical guide.

**⚠️ What to *limit or adjust* for calcium phosphate stones**

**1. Avoid excessive alkalinizing diets**

Your urine is already alkaline (pH 6.5), and **topiramate raises it further**, pushing calcium phosphate to crystallize.

Limit very alkaline-forming dietary patterns such as:

- Large volumes of green smoothies/juicing
- Very high intakes of spinach, kale, beet greens, Swiss chard
- Heavy reliance on “alkaline diets” or supplements (alkaline water, baking soda, etc.)

You don’t need to avoid these foods entirely—just avoid exaggerated amounts.

**2. Moderate sodium intake**

High sodium increases urinary calcium.

Aim for:

- **<2,300 mg/day sodium**, or lower if advised
- Avoid salty processed foods, fast foods, canned soups, chips, deli meats

**3. Avoid excessive animal protein**

Too much protein increases calcium excretion and lowers citrate.

Aim for:

- Moderate portions (e.g., 3–4 oz at meals)
- Include plant protein sources (beans, lentils, tofu) in a balanced way

**4. Be careful with supplements**

- **Avoid high-dose vitamin C (>500 mg/day)** → increases oxalate production.
- **Avoid calcium supplements unless your clinician recommends them**; food sources are preferred.
- **Avoid phosphate or alkaline mineral supplements** unless prescribed.

**🧠 Special consideration: Topiramate**

Topiramate:

- **Raises urine pH** (promotes calcium phosphate stones)
- **Lowers citrate** (reduces natural stone protection)

Diet alone cannot fully counteract these effects. Your clinician might:

- Adjust your topiramate dose
- Consider potassium citrate (careful, as it can raise pH further—must be monitored)
- Monitor 24-hour urine parameters after dietary changes

**🌟 Putting it together: A simple daily plan**

**Better choices**

- Water with lemon
- Yogurt or milk with meals
- Moderate protein: fish, poultry, tofu, beans
- Fruits: citrus, melons
- Vegetables in normal portions (not huge blended quantities)

**Limit**

- Salty snacks, processed foods
- Large quantities of leafy-green smoothies
- Excess red meat
- High-dose supplements
- “Alkaline” powders/drinks

**📌 Bottom line**

For someone with **calcium phosphate stones**, **pH 6.5**, and **topiramate use**, the most relevant dietary strategies are:

- Keep **normal calcium intake with meals**
- Boost **dietary citrate**
- **Restrict high sodium** and **moderate protein**
- Avoid **over-alkalinizing diets** that raise urine pH further
- Maintain **high fluid intake**
- Work with your clinician to monitor pH and citrate, given topiramate’s effects

**SB + QB:**

Below is a clear, guideline-aligned explanation tailored to calcium phosphate (CaP) stone formers on **topiramate**, which is known to alter urinary chemistry in a way that promotes CaP stones.

**1. Metabolic Diagnosis**

**Your elevated urine pH (6.5) is *very likely medication-related*, caused by topiramate-induced metabolic acidosis and renal bicarbonate loss.**
Topiramate acts as a **carbonic anhydrase inhibitor**, which:

- Raises urine pH (alkaline urine favors **calcium phosphate** crystallization).
- Lowers urine citrate (hypocitraturia), further increasing stone risk.
- May increase calcium excretion and supersaturation for CaP stones.

This constellation—**high pH + low citrate + CaP stones**—is classic for topiramate exposure.

**2. Key Dietary Modifications (Top 3 Priorities)**

These recommendations follow **EAU/CUA guidelines** for CaP stone prevention and metabolic acidosis correction:

**1. Reduce dietary alkali load**

You should **not** intentionally alkalinize your urine (unlike CaOx stone formers).
Avoid excess:

- Plant-based alkaline waters
- Baking soda
- High-alkali diets (very high fruit/vegetable smoothies, especially spinach/banana mixes)

**2. Limit sodium to <2 g/day (≈5 g salt/day)**

High sodium → higher urine calcium → more CaP supersaturation.
This is especially important if topiramate is already elevating pH.

**3. Maintain *normal* dietary calcium (1000–1200 mg/day) with meals**

Do **not** restrict calcium. Normal calcium intake helps bind dietary oxalate and stabilize bone metabolism.
Choose:

- Dairy with meals
- Calcium-fortified plant milks (unsweetened)

**3. Sample Menu Suggestions**

**Helpful foods for CaP stone formers on topiramate**

- **Protein:** Fish, poultry, eggs; moderate portions (≤0.8–1.0 g/kg/day)
- **Vegetables:** Neutral-acid load vegetables (green beans, zucchini, carrots, cabbage, cauliflower)
- **Fruits (in moderation):** Berries, apples, grapes (avoid very alkaline fruits in excess, e.g., bananas in smoothies)
- **Calcium sources:** Yogurt, kefir, cheddar, tofu with calcium sulfate
- **Hydration:** Plain water; no alkaline waters

**Foods/beverages to *limit***

- **High-alkali smoothies:** spinach + banana + almond milk
- **Alkalinized or “electrolyte” waters** (pH > 8)
- **Salt-heavy foods:** deli meats, canned soups, fast food
- **Large amounts of nuts/seeds** (alkali load + phosphorus)
- **Excess animal protein** (can worsen metabolic acidosis, though also lowers urine pH somewhat—balance is key)

**4. Safety / Medication Warning**

**Important**

Topiramate is a **well-documented cause of CaP stone formation**.
If you continue to form CaP stones or if your urine pH stays ≥6.5:

- **This cannot be solved with diet alone.**
- You should discuss with your neurologist whether:
  - A dose reduction is possible
  - An alternative medication exists
  - Potassium citrate is *contraindicated* for CaP stone formers with high pH (it raises pH further), so it must be used only with strict monitoring.

If you develop symptoms of **metabolic acidosis** (fatigue, deep/rapid breathing), that also warrants urgent re-evaluation of the medication.

**Std + QB:**

I can help explain how **topiramate**, **urine pH**, and **calcium phosphate stones** interact, and what is reasonable to address with **diet** versus with your **neurologist**.
(*This is general information, not personal medical advice.*)

**🔗 1. Is your elevated urine pH likely related to topiramate?**

In many cases, **yes**.

Topiramate is a carbonic anhydrase inhibitor. This class of medication:

- Causes **metabolic acidosis** (slightly lower blood pH)
- Leads the kidneys to excrete more bicarbonate
- Result: **urine becomes more alkaline** (higher pH)

A urine pH of **6.5** is consistent with what topiramate commonly produces.
This shift toward alkalinity increases the risk of **calcium phosphate stone formation**, particularly **brushite** and **apatite**, which grow more readily at pH > 6.2.

So the medication is **very plausibly a major contributor**.

**🔍 2. What you can address with diet**

Diet can help, but for **calcium phosphate** stones specifically, diet cannot fully counteract medication-driven alkalinization.

Useful dietary steps:

**✔️ Maintain normal calcium intake**

Paradoxically, **restricting calcium increases stone risk**. Target: ~1000–1200 mg/day from food.

**✔️ Lower sodium intake**

High sodium → increased urinary calcium.
Goal: **≤ 2 g sodium/day (≈ 5 g salt)**.

**✔️ Moderate animal protein**

Reduces acid load slightly and lowers urinary calcium, but does **not substantially lower urine pH** if the medication is driving it.

**✔️ Increase fluid intake**

Aim for urine output **> 2–2.5 L/day**.

**✔️ Avoid alkalinizing supplements**

- **Potassium citrate**: helps for calcium oxalate or uric acid stones but can *worsen* calcium phosphate risk by raising pH. Should only be used if your stone clinic specifically recommends it.

**⚠️ What diet CANNOT reliably do**

- **Lower urine pH** in a durable way when topiramate is the cause
  Dietary acidification (e.g., more animal protein) can lower pH a little but often causes other problems: more urinary calcium, more acid load, more stone risk overall. It is not an effective or recommended strategy for medication-induced alkaline urine.

**🧠 3. What to discuss with your neurologist**

Because diet can’t fully offset topiramate’s effect on urine pH, it is appropriate to **bring this up with the prescribing neurologist**. Topics to discuss:

**✔️ Is topiramate essential?**

Some people can switch to an alternative medication (e.g., for migraine or seizures) that **does not cause urine alkalinization**.

**✔️ Dose adjustments**

Sometimes lowering the dose reduces the bicarbonate-wasting effect.

**✔️ Monitoring**

Your neurologist may want:

- Serum bicarbonate levels
- Repeat urine chemistries
- Assessment of stone recurrence risk

**✔️ Coordination with a nephrologist or stone specialist**

When topiramate must be continued, a kidney stone specialist can help manage:

- Acid–base balance
- Urinary citrate
- Stone prevention strategies tailored to your labs

**🧭 Bottom line**

- **Yes, topiramate is a common and likely cause** of elevated urine pH and **calcium phosphate stone formation**.
- **Diet helps but cannot fully reverse the medication effect** on urine pH.
- It is **appropriate to discuss this with your neurologist**, especially if you have recurrent stones, to consider medication alternatives or adjustments.
